# Are Viruses Taxonomic Units? A Protein Domain and Loop-Centric Phylogenomic Assessment

**DOI:** 10.3390/v16071061

**Published:** 2024-06-30

**Authors:** Gustavo Caetano-Anollés

**Affiliations:** Evolutionary Bioinformatics Laboratory, Department of Crop Sciences, C. R. Woese Institute for Genomic Biology, University of Illinois, Urbana, IL 61801, USA; gca@illinois.edu

**Keywords:** holobionts, hologenomes, LUCA, origin of viruses, phylogenomic reconstruction, process, protein structure, reductive evolution, superkingdom, syncytins

## Abstract

Virus taxonomy uses a Linnaean-like subsumption hierarchy to classify viruses into taxonomic units at species and higher rank levels. Virus species are considered monophyletic groups of mobile genetic elements (MGEs) often delimited by the phylogenetic analysis of aligned genomic or metagenomic sequences. Taxonomic units are assumed to be independent organizational, functional and evolutionary units that follow a ‘natural history’ rationale. Here, I use phylogenomic and other arguments to show that viruses are not self-standing genetically-driven systems acting as evolutionary units. Instead, they are crucial components of holobionts, which are units of biological organization that dynamically integrate the genetics, epigenetic, physiological and functional properties of their co-evolving members. Remarkably, phylogenomic analyses show that viruses share protein domains and loops with cells throughout history via massive processes of reticulate evolution, helping spread evolutionary innovations across a wider taxonomic spectrum. Thus, viruses are not merely MGEs or microbes. Instead, their genomes and proteomes conduct cellularly integrated processes akin to those cataloged by the GO Consortium. This prompts the generation of compositional hierarchies that replace the ‘is-a-kind-of’ by a ‘is-a-part-of’ logic to better describe the mereology of integrated cellular and viral makeup. My analysis demands a new paradigm that integrates virus taxonomy into a modern evolutionarily centered taxonomy of organisms.

## 1. Introduction

The breakthrough discovery of the ‘microbe-mimicking’ virus (Mimivirus) by Jean-Michel Claverie and colleagues early this century [1] opened a new era for virology that rekindled theories of viral origin and evolution [2]. It also opened a Pandora’s box of surprises and questions. Mimiviruses are giant viruses that belong to the *Nucleocytoviricota* phylum, which is a group of viruses with large double-stranded DNA genomes [3]. While giant viruses lack many of the hallmarks of cellular life, including the ability to synthesize their own proteins, produce energy in the form of ATP for cellular activities, and reproduce by dividing, their large genomes resemble those of obligate parasitic bacteria, containing a primordial ‘core’ set of genes that is common to all cellular life. When transcribed, the encoded proteins organize the activities of infected cells, producing myriads of viral particles in specialized cellular structures (viral factories) that ensure the continuance of their life cycles [4,5]. Mimiviruses package their genomes into virions made of viral proteins [4]. Other giant viruses, including Mollivirus, Pithovirus and Pandoravirus, are protected by more complex cell-like envelopes [5]. Mimivirus has two internal lipid membranes: one lining the fibril-decorated icosahedral capsid and the other lining the nucleoid compartment that holds the genome and hundreds of proteins. While capsid layers cover lipid membranes that are often essential for infectivity, large viruses such as the amphora-shaped Pandoravirus have virions with at least one lipid layer lining a thick three-layer tegument, one of which is made of cellulose [6]. The large genomes of giant viruses are mosaics of genes, many of which are hallmarks of cellular organisms, including genes with roles in protein biosynthesis and metabolic functions. Examples include genomes encoding tRNAs, translation factors, aminoacyl-tRNA synthetases, in some cases complete sets of them [7], GMC-type oxidoreductases constituting the glycosylated fibrils of the capsid, and enzymes that protect the virion from oxidative stress [8]. Some viruses such as *Marseilleviridae* encode nucleosome-forming histones that help package genomes into virions (e.g., [9]). A virus-encoded transcription machinery (often confined to the nucleoid) that initiates the viral infection cycle includes an RNA polymerase. Enigmatically, smaller viruses (virophages) were found to infect some giant viruses and abort their life cycles [10], integrating in some cases into their genomes and therefore acting as double-stranded DNA episomes (transpovirons) capable of replicating independently of their hosts [11,12].

All of these features beg the question of whether viruses are independent units of organization, function and evolution. In fact, much of the viral machinery for entry, initiation, replication and exit appears fully integrated with the makeup of the host through, for example, membrane fusion, endocytosis and the production of specialized cellular functions [5]. In this regard, placing viral life cycles within a processual context may help assess their identity and origin. Here, I revisit the cellular origin of viruses, the phylogenomic data that support its validity, and the impact of viruses on holobionts and superkingdoms of cellular life (Archaea, Bacteria and Eukarya). I focus on two prior molecular states of proteins [13]: *structural domains*, the recognized structural, functional and evolutionary units of proteins, and *protein loops*, the architects of protein structure. The hypothesis of virus origins that emerges from structural phylogenomic exploration is firmly grounded in virus biology and benefits from concepts of information transfer, language, and molecular biocommunication [14]. The goal of this review is to address the fundamental question: Are viruses taxonomic units? A positive answer justifies half a century effort of virus classification [3]. A negative answer demands a new paradigm that integrates virus taxonomy into a more modern evolutionarily centered taxonomy of organisms [15].

Section 2 introduces the comparative and evolutionary genomic approaches that I here use to explore the evolutionary history of protein domains and loops. Section 3 shows results that describe how viruses spread molecular wealth in the proteomes of superkingdoms, facilitate horizontal transfer, and help microbes spread molecular innovations efficiently. Section 4 introduces the difficulties of taxonomic classification (co-evolution, reticulation, independent origins) and addresses the central taxonomy-relevant question of the study with phylogenomic data. Finally, Section 5 provides recommendations.

## 2. Methodological Approach

### 2.1. Structural Phylogenomic Reconstructions with Alignment-Free Methods

Phylogenomic reconstruction entails building phylogenetic trees (phylogenies) from data and models of evolutionary change, rooting the reconstructed trees and tracking change along their branches [16]. The protein structure is at least 3–10 times more evolutionarily conserved than the sequence and can be effectively used to uncover deep evolutionary history [17]. Because homology among structural domains forms the basis of many protein classification schemes, including the SCOP [18] and CATH [19] gold standards of protein taxonomy, we focused on identifying observable features of interest (characters) in the sequence, structure and function of domains that would hold significant evolutionary history and would comply with the ‘memory’ axiom of evolution [16]. Since 2003, we have been reconstructing history from genomic censuses of domains defined at different levels of SCOP and CATH classification in the proteomes of thousands of organisms and viruses, which is an effort that has been recently reviewed [20]. In the SCOP hierarchy, the fundamental unit of classification is the domain present in experimentally determined protein structures deposited in the PDB database. These domains are grouped into families, superfamilies, folds and classes. The evolutionary relatives of families and superfamilies are supported by the sequence and structural evidence of common evolutionary origin. Typically, families group domains with ≥30% sequence identity. Conversely, superfamilies group families that have little or no sequence identity but show strong structural and functional similarities. In contrast with families and superfamilies, the higher taxonomic ranks of folds and classes do not represent homologies, being solely based on structural similarities. Superfamilies are unified into folds when they share similar structural designs. Folds are unified into classes when they share similar secondary structure content and organization. SCOP development, which concluded in June 2009 with release 1.75, is currently followed by its extended and backward-compatible version, SCOPe [21,22]. SCOPe is curated with both manual and validated automated methods, taking advantage of the software and databases of the ASTRAL compendium [21]. Superfamilies are more evolutionarily conserved than families. Their conservation is demonstrated by the mapping of ~11 million sequences in 5080 proteomes to only ~2000 superfamilies detected at *E* < 0.0001 [23] and the fact that their conserved structural cores rarely evolve by convergent evolution [24].

**Figure 1:**
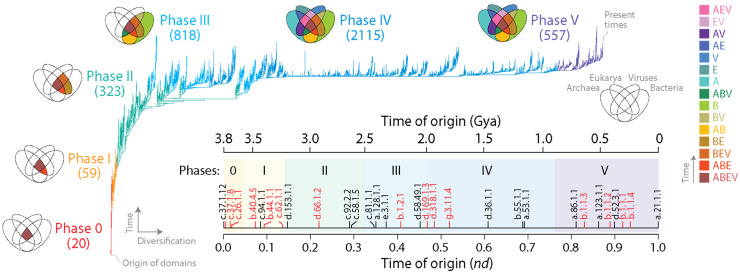
Phylogenomic tree of domains (ToD) defined at SCOP family level with branches colored according to the 6 evolutionary phases of the protein world. The ToD (2,083,556 steps; retention index = 0.704; g_1_ = 0.0004) describes the evolution of 3892 domains. Labels of leaves are not provided as they would not be legible. Venn diagrams describe the distribution of families among superkingdoms Archaea, Bacteria and Eukarya and viruses as new families accumulated in the branches of the tree and defined evolutionary phases. Numbers in parentheses represent new families appearing in each phase. Venn-group colors reflect the evolutionary chronology of Venn-group appearance. The inset shows an evolutionary chronology directly derived from the tree indexed with phases and illustrated with molecular clock (black) and landmark (red) markers identified with SCOP concise classification strings (*ccs*). Time of origin was expressed as the node distance (*nd*) or as billions of years ago (Gya). Clock markers: c.37.1.12, ABC transporter ATPase domain-like; c.94.1.1, Phosphate binding protein-like; d.153.1.1, Class II glutamine amidotransferases; c.92.2.2, TroA-like; c.58.1.5, Shikimate dehydrogenase-like; c.81.1.1, Formate dehydrogenase/DMSO reductase, domains 1–3; a.128.1.1, Isoprenyl diphosphate synthases; d.58.49.1, YajQ-like; d.36.1.1, Chalcone isomerase; b.55.1.1, Pleckstrin-homology domain; a.53.1.1, p53 tetramerization domain; a.86.1.1, Hemocyanin middle domain; a.123.1.1, Nuclear receptor ligand-binding domain; d.52.3.1, Prokaryotic type KH domain; a.21.1.1, HMG-box. Landmark markers: c.37.1.8, G-proteins; c.26.1.1, Class I aminoacyl-tRNA synthetases catalytic domain; b.40.4.5, Cold shock DNA-binding domain-like; b.44.1.1, EF-Tu/eEF-1alpha/elF2-gamma C-terminal domain; c.62.1.1, Integrin A(or I) domain; d.66.1.2, Ribosomal protein S4; b.1.2.1, Fibronectin type III; d.169.1.3, Invasin/intimin cell-adhesion fragment, C-terminal domain; d.318.1.1, SARS receptor-binding domain-like; g.3.11.4, Merozoite surface protein 1; b.1.1, Immunoglobulin superfamily.

Here, I reanalyze phylogenomic reconstructions that were based on the total count (abundance) of each of 3892 domain families that were present in the proteomes of 8127 organisms and viruses [25]. A total of 139 archaeal, 1734 bacterial, 210 eukaryal and 6044 viral reference-quality (RefSeq) proteomes were included in the analysis. Viral proteomes encompassed those from 85 archareoviruses, 2161 bacterioviruses and 4224 eukaryoviruses. The nucleo-cytoplasmic large DNA viruses belonging to the *Nucleocytoviricota* phylum (previously the *Megavirales* order) that includes giant viruses was represented by 119 proteomes. Figure 1 shows the single most parsimonious tree of domains (ToD) recovered using a previously published phylogenomic reconstruction [23]. File S1 contains the Newick tree in readable *nexus* format. The leaves of the tree (taxa) describe domains, while branches represent diversification events associated with the spread of structural innovations in proteomes. Raw abundance values were log-transformed, rescaled according to the abundance levels of each proteome, and binned into 24 phylogenetic character states. Normalization and rescaling protected against the effects of unequal proteome sizes and variances and ensured software compatibility. The resulting data matrix in *nexus* format was then used to reconstruct optimal trees using a maximum parsimony (MP) optimality criterion and a heuristic ‘search’ strategy in PAUP* [26]. The method involves optimizing the fit of data along the branches of trees while travelling through the tree solution space (via branch-swapping operations) and complying with a model of character state change (transformation series) defined by a fully ordered character state graph (CSG) of undirected ordered (Wagner) characters. These ordered multistate characters represent ‘serial homologies’ that are typical of many morphological characters but that are rare in the study of molecular sequences. Note that MP approximates maximum likelihood (ML) when phylogenetic characters evolve at different rates [27] and outperforms ML and other methods of phylogenetic inference under models appropriate for morphology [28] such as those used in our phylogenomic studies. Moreover, the claimed statistical inconsistency of MP is invalid and irrelevant for justifying the use of ML and Bayesian inference [29], especially when using multistate Wagner characters. In our searches, we only used characters that were phylogenetically informative (diagnostic), i.e., synapomorphies that complied with the ‘shared-and-derived’ tenet of phylogenetic systematics. Recovered optimal trees were visualized with Figtree ver. 1.4.4 [30], and the reliability of deep evolutionary relationships was assessed with 2000 bootstrap replicates. Finally, ToDs were rooted to establish the direction of evolutionary change using Lundberg optimization [31], which places the root at the most parsimonious location. The method invokes Weston’s generality criterion [32,33], which optimizes ancestral-derived homology relationships in nested patterns along branches of the trees (the ‘standard’ implementation) or makes use of a maximum or minimum state ancestor according to evolutionary considerations (the ‘ancestor’ implementation) [34]. Note that the generality criterion is a powerful ‘direct’ rooting approach that minimizes the number of assumptions and avoids resorting to an outgroup or a molecular clock model [16].

### 2.2. Evolutionary Chronologies

When rooted, ToDs describe the origin and evolution of domains. This is because rooting establishes an evolutionary order of branching, and the resulting branching patterns are significantly imbalanced. These properties allow estimation of a ‘time of origin’ for each structural domain (each leaf) (Figure 1, inset; Table S1). Such time estimates can be either relative or absolute. A node distance (*nd*) from the root to the leaves of the tree provides a relative measure of ancestry in a scale from 0 (oldest) to 1 (youngest) [35]. Alternatively, a clock of folds calibrated with biomarkers and geomarkers in billions of years (Gy) can link the geological record and domain evolution [36]. Ancestries allow to produce evolutionary timelines directly from the trees, revealing patterns of domain accretion and diversification over time. Note that times of origin of families derived from ToDs are ultimately dependent on a ‘profile’ distribution of both the occurrence and abundance of the families in proteomes. To illustrate, the most ancient family is the ‘ABC transporter ATPase domain-like’ (SCOP c.37.1.12). The family is both the most popular and the most widely represented in the proteomes of all superkingdoms and viruses. The families of the immunoglobulin superfamily (SCOP *concise classification string* b.1.1) that make up antibodies are also very abundant. Despite their very high abundance, they are not the most ancient families, because their high abundance is only restricted to selected lineages of eukaryotes (*nd* = 0.829–0.936; Figure 1). The contrasting examples show that both the abundance and spread of families in proteomes determine their individual position as leaves in the tree or as families in the timeline. Following almost two decades of phylogenomic experimentation, the relative positions of domains in evolutionary chronologies has been quite reproducible [20]. Similarly, chronological statements derived from phylogenomic reconstructions with structures defined using different approaches and different levels of abstraction have been congruently recovered. This suggests that the combination of the abundance and occurrence of structural domains in proteomes represents molecular features that are quite resistant or even benefit from non-vertical processes of gene exchange. Thus, increases in abundance by horizontal gene transfer (HGT) or decreases by loss or artificially by incomplete or biased sampling should not be considered significant factors. Perhaps this stems from negligible effects of non-vertical processes on the genomic abundance of ancient domain structures that are already highly abundant and, conversely, a limited effect of recently evolved structures that are necessarily present in proteomes with low count, as this would only affect few and very derived branches of the ToD.

The distribution of domain families belonging to Archaea, Bacteria, Eukarya and viruses defines a Venn diagram in which Venn groups describe sharing patterns between these supergroups (Figure 1). One expectation from both the profile distribution of family occurrence and abundance in the phylogenetic data matrix and compliance with Weston’s generality criterion is that the most ancient families should populate the deep branches of the ToD or the basal positions of the chronology, and that conversely, newer families should be located toward the crown of the tree or in derived positions of the timeline. The progression of evolutionary appearance of Venn groups, which was consistently recovered in four studies that included viruses [23,24,37,38], followed a specific temporal order that fulfilled that expectation. The Venn-group labels of Figure 1 are ordered (bottom to top) according to their appearance in the evolutionary timeline. This order delimited six evolutionary phases and two universal ancestors of life, which is the last universal common ancestor (LUCA) and the last universal cellular ancestor (LUCellA). Some implications of phases and ancestors were discussed in ref. [13]. Phase 0 (*nulla* in roman numerals; communal world) was only populated by universal families present in proteomes of cellular organisms and in many viruses. Phase I (rise of viral ancestors) was populated by universal families and by a minority of families common to all superkingdoms. Phase II (birth of archaeal ancestors) comprised four Venn groups (ABEV, ABE, BEV, and BE) and defined an ancestral stem line of cellular descent. Phase III (diversified Bacteria) harbored more than half of the Venn groups, all of them involving Bacteria. Finally, Phases IV and V introduced the rest of the Venn groups, including virus-specific families (VSFs) corresponding to Venn group V.

### 2.3. Loops: Architects of Protein Domains

Proteins are organized entities. Polypeptide chains fold into helical (mostly α-helices) or extended (β-strands) segments of regular structure mediated by hydrogen-bonding electrostatic interactions between carbonyl and amino groups of the main chain established at close or long distance range, respectively. Folding is a cooperative process of coalescence of these protein ‘secondary’ structures into higher-order arrangements, which also takes advantage of co-translational stabilization and packing. Returns of the polypeptide chain produce closed loops structures defined by helix, strand and turn segments with geometries captured by structural motifs known as *Smotifs* [39,40]. Examples of these ‘supersecondary’ elements include α-hairpins, β-hairpins and β-turns. Protein loop structures can be evolutionarily conserved and can serve as structural and functional units. Local sequence and structural similarities and sequence profiles driven by position-specific scoring matrices have identified non-combinable loops present in highly popular fold structures [41] and combinable ‘elementary functional loops’ (EFLs) that form active binding sites for binding cofactors [42] and enabling molecular functions [43]. EFLs grouped ABC transporters, aminoacyl-tRNA synthetases and helicases, methylases and methyltransferases, metal-binding proteins, transcriptional regulators, and cell surface proteins that were present in archaeal proteomes [44]. These groups likely represent ancient structural scaffolds. An evolutionary chronology of EFLs unfolding in a time series of bipartite networks of EFLs and domains confirmed this expectation [45]. It revealed that EFLs had origins in nucleotide-binding P-loop motifs of ABC transporters and that they were recruited to form domain structures in an active ongoing process. While non-combinable and combinable loops were typically 25–39 amino acid residue long, longer motifs involving longer sequence segments of up to 200 residues have been identified [46]. These ‘themes’ are highly reused and represent higher-order supersecondary structural elements.

Phylogenomic analysis can help establish evolutionary trajectories of the structural elements that are responsible for protein makeup [45]. In recent studies, we traced the evolution of *Smotifs* along timelines of structural domains [13,47,48]. We mapped loops sourced from ArchDB [49], which is a database that classifies loops based on geometry and conformation. Loop prototypes were defined using a Density Search (DS) algorithm that detects regions of feature space with a high density of loops around a centroid defined by loop length, conformation and geometry. Prototypes were filtered using mappings of prototypes to domain families at e-value <0.001. Because the libraries of both prototypes [50] and domains [51] are considered complete, the mapping of all possible *Smotifs* to domains makes evolutionary statements universal and powerful. Tracing loop prototypes along the chronology of domains revealed that out of 7110 identified prototypes, 5125 mapped exclusively to single-domain families of a similar time of origin. They were labeled ‘non-modular’ prototypes. The rest were recruited multiple times throughout the timeline to form a multitude of domain structures. The recruitment of the ‘modular’ prototypes was made explicit with time series of evolving bipartite networks of loops and domains, revealing two primordial waves of functional innovation involving founder ‘P-loop’ and ‘winged helix’ loop structures and an intricate tangle of loop combinations [47]. A focus on the more numerous non-modular prototypes avoided the need to untangle the complicating effect of evolutionary recruitments, enabling instead an evolutionary study of intrinsic disorder in protein loops [48]. Similarly, here, I focus on non-modular loop structures mapping to domains that are unique to cellular organisms and those that are shared with viruses. Because protein loops are stepping stones to the protein domain structure, exploring the evolution of loops and domains allows to study how these two related prior molecular states emerged in the protein world.

**Figure 2:**
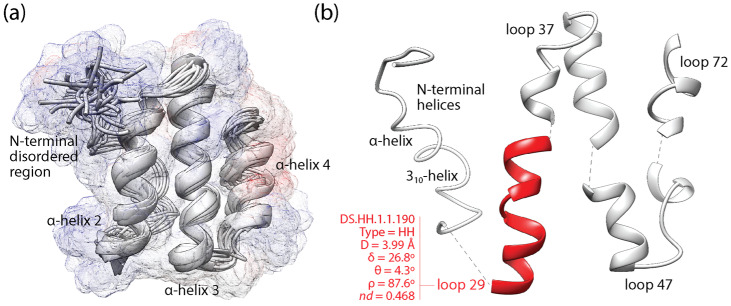
The loop structures of the non-structural protein 7 (NSP 7) of coronaviruses. (**a**) An alignment of the best 20 conformers of SARS-CoV NSP7 obtained by energy minimization from NMR screening (PDB entry 1YSY). The solution structure consists of an up–down–up ‘helical sheet’ composed of α-helices 2, 3 and 4, which packs on one of its sides an N-terminal region of loosely winded helices that together with α-helices 2 and 3 forms a 3-helix bundle. A semi-transparent surface representation embeds the cartoon structure of the backbone and is colored according to Coulombic electrostatic potentials (positive values in blue reflect positively charged surfaces). (**b**) Dissection of the fold structure into an N-terminal helical region and 4 loop structures, one of which (loop 29) matches a non-modular loop prototype (colored red) that embeds highly conserved and likely functional sites [52,53]. The loop prototype is indexed with type of bracing secondary structures (H stands for α-helix), the four ArchDB internal coordinates (distance between boundaries of aperiodic structure (D), hoist angle (δ), packing angle (θ), and meridian angle (ρ)) and time of origin (*nd*).

Figure 2 illustrates the mapping of loop prototypes to the domain structure of a VSF, the coronavirus NSP7-like family (SCOP a.8.9.1), which is exemplified with the NSP7 protein of the SARS-CoV virus [52]. The NSP7 fold embeds an ‘helical sheet’ of three α-helices, the first of which interacts with NSP8 through an ‘α-helical band’ needed for NSP12-driven RNA-dependent RNA polymerase activity of the viral replication/transcription complex [53]. This first helix of the helical sheet embeds the non-modular loop prototype DS.HH.1.1.190, which delimits a positively charged surface of interactions (blue bulge protruding from the start of α-helix 2; Figure 2a) and harbors highly conserved and likely functional sites [52] in its extended-compact loop structure (Figure 2b). This prototype is one of the 5125 non-modular prototypes examined in the mappings of this study.

## 3. Phylogenomic Analysis of Protein Structural Domains and Loops

### 3.1. Viruses Help Spread Domain Wealth in the Proteomes of Superkingdoms

**Figure 3:**
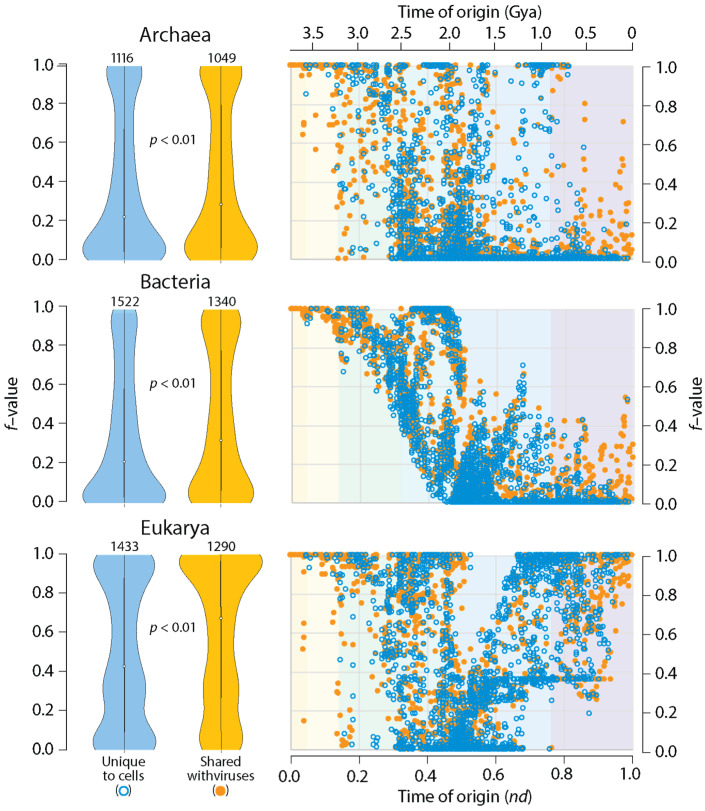
Structural domains shared with viruses spread more widely in the proteomes of Archaea, Bacteria and Eukarya. *Violin plots* (in the left) compare the spread (*f*-value) of domains in the proteomes of individual superkingdoms when domains are unique to cells (blue plots) or shared with viruses (orange plots). The *f*-value represents a distribution index that evaluates the number of species that uses a domain relative to the total number of species analyzed. Numbers in the top of violin plots represent the total number of domains involved in comparisons, all of which were statistically significant (Wilcoxon rank test, two-tailed, *p* < 0.01). *Chronologies* (in the right) compute individual *f*-values of domains and plots them along the timeline of domain families indexed with the six evolutionary phases (see Figure 1). Domains were defined at the SCOP family level.

A simple comparative proteomic exercise reveals that structural domains that are shared with viruses are more widely represented in the proteomes of organisms belonging to Archaea, Bacteria and Eukarya (Figure 3). A distribution index (*f*-value) that measures how many proteomes harbor individual domains, over all domains that are known, shows that viruses help spread the wealth of domains. This is an expected result. Two studies have already reported this finding [23,25]. However, I here extend the analysis by mapping *f*-values of domains unique to cells or shared with viruses along the domain chronology described in Figure 1 (Table S1). Remarkably, domains shared with viruses that appear late in the timeline had significantly higher *f*-values than cell-unique domains with similar times of origin. These were especially evident in plots for Archaea and Bacteria (Figure 3). It is noteworthy that the origin of these domains followed the appearance of 95 virus-specific domains in Phase IV at *nd* = 0.47–0.53. These VSF domains, which are absent in superkingdoms but are present in viruses (Venn group V), include domains defining capsid/coat proteins necessary for the formation of virions. Thus, viruses help spread domain wealth among a diversifying world of organisms, suggesting they are not ‘passive’ contributors to the structures and functions of the cellular world.

The chronologies of Figure 3 showcase patterns of evolutionary decrease, and then, increase in *f*-value distribution that are distinct in Archaea, Bacteria and Eukarya. These patterns were first described in a phylogenomic study of SCOP fold and superfamily domains [54] but were confirmed and found to be remarkably consistent in a number of subsequent studies that focused on domain combinations [55], SCOP domain families [56], and domains defined by the CATH classification [57]. Patterns of decrease reflected early episodes of domain loss (the so-called ‘loser trend’) in archaeal and eukaryal stem lines of descent and an increasingly restricted distribution of novel domains in emerging lineages (see [54] for an initial description). Because in the present study there were no efforts to exclude organisms with parasitic lifestyles, a ‘modern’ effect of reductive evolution was also expected to impact domain occurrence and abundance throughout the timeline. This was likely responsible for *f*-value decreases in ancient domains. Conversely, patterns of increase were induced by historical events of HGT, endosymbiosis, fusions, recruitments, and rearrangements, among many other factors [54,55,56,57]. The impact of viruses on domain wealth can now be added to this growing list of non-vertical processes of spread and exchange.

### 3.2. Viruses and the Evolutionary Primacy of Horizontal Transfer of Prior Molecular States

**Figure 4:**
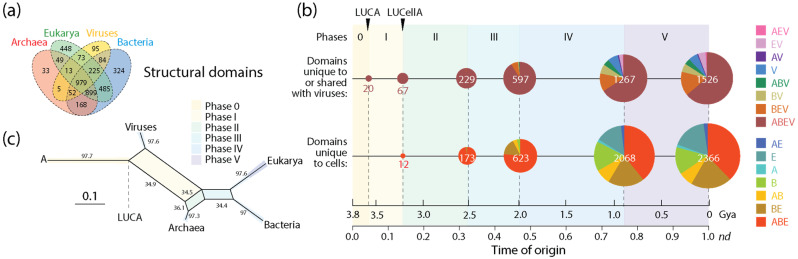
The evolution of protein structural domains that were unique to or shared with viruses and those that were unique to cells. (**a**) A four-set Venn diagram describes the distribution of domain families in the protein world [25]. (**b**) A chronology of domain families defines 6 timestamps of events delimiting evolutionary phases, with pie charts describing Venn-group distributions (colors indexed in the key) with sizes proportional to the number of domain families present at each evolutionary event. Actual domain numbers for each phase can be found in Figure 3 of ref. [20]. (**c**) Phylogenetic network describing the evolution of Archaea, Bacteria, Eukarya and viruses reconstructed directly from Venn-group domain distribution data using the NeighborNet algorithm and uncorrected-P distances [13]. Bootstrap support values (%) are given for individual edges following a bootstrap analysis with 2000 replicates. The splits of the network are shaded with colors describing evolutionary phases.

The number of domain families unique to or shared with viruses (1526) was significant in relation to the number of families unique to cells (2366). Tracing the accumulation of these sets and their corresponding Venn groups (Figure 4a) along the chronology of domains showed that such dichotomous distribution was both maintained throughout protein evolution and unfolded remarkable evolutionary patterns (Figure 4b). Venn groups ABEV and ABE defined LUCA and LUCellA during the first two evolutionary phases of the timeline. At the end of Phase II (birth of archaeal ancestors), 229 families shared with viruses and 173 families unique to cells delimited an ancestral stem line of cellular descent that dissected the primordial cellular world into ancestors of viruses (defined by the ABEV and BEV groups) and cells (defined by the ABE and BE groups) via ‘double-faced’ episodes of differential growth and/or reductive evolution affecting ancestors of viruses and Archaea. This trend, which continues to the present, manifests in an inflection point at the end of Phase III (diversified bacteria), in which the primacy of the set that was shared with viruses (597) reverses when compared to the set that was unique to cells (623). This global trend of differential growth is ultimately responsible for the limited proteomic repertoires of viruses (see plots of domain use and reuse in ref. [23]). Venn groups shared with viruses always appeared earlier than those that were unique to cells (e.g., BEV at the beginning of Phase II and BE at its end; see Figure 3 in ref. [20]), suggesting viruses played important roles in the diversification of superkingdoms throughout the timeline. Note, however, that the relationship between the number of domains in Venn groups that were unique to or shared with viruses and domains that were universal (the ABEV group) was significantly constrained in evolution when compared to the wide diversity of domains of Venn groups that are unique to cells. Perhaps this bias can be explained by viruses acting as ‘melting pots’ in which newly created and useful domains arising in viruses were being readily transferred to cells to enrich in particular the widely shared Venn groups (ABEV, ABE, BEV and BE). Note that Venn group distribution can be used as fruitful phylogenetic characters to construct phylogenetic networks describing the origin and evolution of Archaea, Bacteria, Eukarya and viruses. Figure 4c shows networks built using the NeighborNet algorithm that was rooted in an ancestral cell (A) ready to host prior molecular states of proteins (networks built in ref. [13]). The networks show the evolutionary progression of emergence of the stem lines of viruses and superkingdoms [13]. The significant reticulations arising after LUCA are noteworthy. They suggest that extensive horizontal exchange occurred during virus and organismal history.

**Figure 5:**
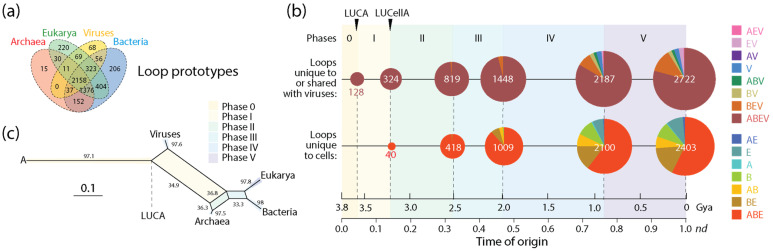
The evolution of protein loop prototypes that were unique to or shared with viruses and those that were unique to cells. (**a**) A four-set Venn diagram describes the distribution of non-modular prototypes in the protein world [48]. (**b**) A chronology of prototypes defines 6 timestamps of events delimiting evolutionary phases, with pie charts describing Venn-group distributions (colors indexed in the key) with sizes proportional to the number of prototypes present at each evolutionary event. Actual prototype numbers for each Venn group and phase can be found in Figure 4 of ref. [13]. (**c**) Phylogenetic network describing the evolution of Archaea, Bacteria, Eukarya and viruses reconstructed directly from Venn-group loop distribution data using the NeighborNet algorithm and uncorrected-P distances [13]. Bootstrap support values (%) are given for individual edges following a bootstrap analysis with 2000 replicates. The splits of the network are shaded with colors describing evolutionary phases.

A similar analysis of non-modular loop prototypes unique to or shared with viruses (2722) relative to the number of prototypes unique to cells (2403) was also revealing (Figure 5). The analysis supported the concept of viruses mediating the formation of structural domains from loops in protein history and in doing so spreading domain wealth in superkingdoms (observed in Figure 3). Remarkably, evolutionary trends in domain history (Figure 4b) were preserved in the loop history (Figure 5b), including an identical sequence of Venn-group appearances in the timeline. In fact, a phylogenetic network reconstructed from Venn-group distributions of loop structures (Figure 5c) was congruent with the phylogenetic network reconstructed from Venn-group distributions of domains (Figure 4c). Such congruency provides strong support to the evolutionary progression and sequential origin of ancestors of viruses, Archaea, Bacteria, and Eukarya, in that order. Within the confines of remarkably similar evolutionary trends, the numbers of emerging prototypes were substantially larger than those of domain building blocks in all Venn-group categories appearing between Phase 0 and the inflection point of Phase III, especially prototypes that originated earlier and/or were widely spread, such as those belonging to the universal ABEV and ABE sets (compare Figure 4 and Figure 5). The inflection point at the end of Phase III merits discussion since Phases IV and V are part of the ‘organismal diversification’ epoch of protein evolution and likely involve canonical organismal lineages rather than stem lines of descent [54,55,56,57]. The inflection point coincides with the inflection point clearly visible in *f*-value distribution plots that occur at about *nd*~0.5 (1.8 Gya) probably triggered by a massive rise of eukaryotic lineages (Figure 3). Note that loop prototypes and associated domains in the ABEV, ABE, BEV and BE categories originated continuously throughout the timeline, showing horizontal exchange processes were pervasive. The large number of widely shared prior molecular states probably reflects the origination and massive recruitment of loops and domains, which is a process that occurred throughout the timeline and is likely ongoing [13,45,47,58]. The number of loop prototypes involving viruses was also larger than those involving cells (Figure 5b). To illustrate, the percentage of prototypes belonging to the ABEV category in prototypes unique to or shared with viruses decreased from 100% in Phase 0 to 96.3% at end of Phase III and then to 79.2% in the extant protein world (Figure 4b). In turn, prototypes unique to cells belonging to the ABE category decreased from 100% in Phase 0 to 88.5% at the end of Phase III and then to 57.3% in present times. Clearly, sharing loops with viruses pushes them to become even more widely shared, especially during the last two evolutionary phases of the timeline.

A comparison of the numbers of loop prototypes and associated domains appearing in individual phases of the evolutionary timeline also highlighted the important role of viruses and recruitment in protein evolution (Table 1). The calculation of a ratio between the two prior molecular states for those that were unique to or shared with viruses and those that were unique to cells revealed a decreasing trend in ratios unfolding in time and in both categories. However, ratios of prior molecular states involving viruses were always larger than those that were unique to cells, again supporting the important role of viruses in the formation of domains by loop recruitment.

### 3.3. Microbial Supergroup-Specific Prior Molecular States Fail to Remain Persistent

**Figure 6:**
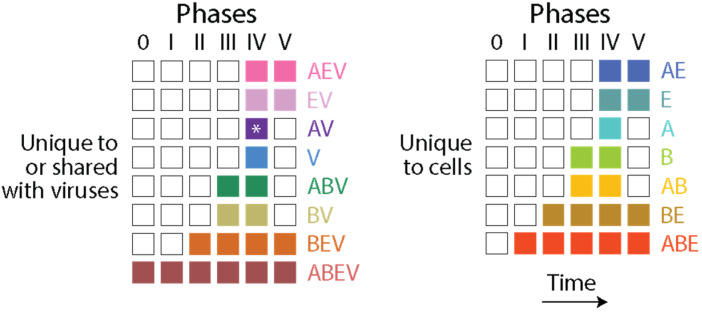
The evolutionary appearance of prior molecular states (loops and domains) in Venn groups describing their distribution among viruses and superkingdoms Archaea, Bacteria and Eukarya. Columns describe evolutionary phases and rows describe Venn groups. The asterisk indicates the Venn group was absent in the analysis of loop prototypes.

The *f*-value distribution plots of Figure 3 show that proteome evolution in Archaea and Bacteria (the microbial superkingdoms) materialized differently along the evolutionary timeline when compared to Eukarya. While the expected high *f*-values of early-originating domains decreased gradually to approximate or attain (by domain loss) values of 0 when stem lines of descent began to produce grades and/or diversified through the formation of lineages (*nd*~0.3–0.5), a ‘strong’ pattern of increase at later times was only evident in Eukarya. The increases in *f*-values occurring during the second half of domain history have been consistently recovered and explained in numerous studies as reflecting HGT events [54,55,56,57]. While many structural innovations were increasingly restricted to smaller sets of organisms making up smaller clades, episodes of domain gain and loss materialized across lineages [59,60], and horizontal events (e.g., domain recruitment [55,58]) were expected to offset the emergence and rise of superkingdom-specific domains in lineages that were massively emerging in the growing Tree of Life (ToL). I here contend that while the horizontal transfer of prior molecular states affected significantly the microbial superkingdoms, the effects were more limited in Eukarya. This conclusion may appear counterintuitive given the *f*-value distributions of Figure 3. However, tracing the presence of Venn groups involving viruses and unique to cells appearing in each of the six evolutionary phases of the chronologies of prior molecular states revealed microbial-specific domain and loop structures that were conspicuously absent in the last phase of protein evolution (Figure 6). Venn groups AV, BV, ABV, V, AB, B and A were clearly absent in Phase V. In contrast, Venn groups AEV, EV, AE and E, which involved Eukarya, were present in that phase. Thus, extensive HGT minimized the opportunity of domains and loops to remain confined to the individual lineages of microbial superkingdoms and viruses. Such transfer appears to represent an unrestricted global effect that may involve persistent interactions in holobionts (see discussion below).

## 4. Are Viruses Taxonomic Units?

In the previous section, I embarked on a phylogenomic exploration of structural domains and loops that make up the proteomes of viruses and the cellular world. My goal was to address a fundamental question: Are viruses taxonomic units? In this section, I first discuss the problems of virus taxonomy, especially the difficulties of considering viruses self-standing biological systems. I then dissect the processual essence of a virus, exploring what should be expected of its evolution. Finally, I assess the validity of the virus species concept given the phylogenomic data that were presented in the previous section.

### 4.1. Taxonomic Classification Is Hampered by Conceptual Difficulties

Taxonomies are classification schemes that are often hierarchical and have the ultimate purpose of assigning elementary entities to established groups known as ‘taxa’ (taxonomic units) at different ‘rank’ levels and according to their similarities and dissimilarities. Note that a taxonomic unit is often defined by features that are unique to that unit and would separate it from other units of the classification scheme. Modern taxonomies organize taxa in a pyramidal structure that follows a ‘subsumption’ (specification) hierarchy. Subsumption implies the logic form of nesting ‘is-a-kind-of’ relationships, which differs from the ‘is-a-part-of’ relationships of ‘compositional’ hierarchies that provide mereological descriptions [61].

In biology, taxonomy is a descriptive discipline devoted to naming, defining and classifying groups of organismal entities based on shared (and when possible shared-and-derived) characteristics. Virus taxonomy is an extension of Linnaean taxonomy that seeks to name and place viruses into a ‘conceptual’ taxonomic system similar to that used to classify organisms. Both Linnaean and virus taxonomies define a ‘species’ as the fundamental unit of classification as well as the lowest taxonomic rank and unit of biodiversity. Both taxonomies have recognized the necessity of making them *natural taxonomies*, i.e., robust taxonomies that reflect taxon evolution with high predictive and explanatory power [62]. Such recognition also stems from ontological, epistemological and methodological assumptions supporting the subsumption logic [61], which argues that taxa develop from earlier and simpler conditions (prior states) that are part of a developmental or evolutionary trajectory, are antecedent (perhaps ancestral), and follow a historical rationale. Defining species, however, has been a refractory proposition, and tracking and reconciling their evolution has been even more difficult [63]. It has been recognized that the canonical view of a species defined by processes of population variation and reproductive isolation [64] is difficult to apply to clonally propagating organisms and microbes and must be complemented with evolutionary history defined by ancestor-descendent relationships following forward-time monophyletic or backward-time coalescent groupings, ecology-defining niches and habitats, phenetic and cohesion criteria, constraints imposed by symbiosis, and gene flow among its members [65]. For example, patterns of recombination among population members of viral genera can help establish species boundaries for viral taxa [66]. While natural selection is certainly a long-term prerequisite for speciation, genome-wide divergence due to reduced gene flow in recombining populations or mutational divergence in clonal populations can be considered in balance with a gene ecology in which gene sets determine reproductive isolation or niche adaptation [65]. Species under some of these more integrated views become ‘separately evolving’ ancestor-descendent series with a capacity to exchange genetic information. Still, the definition of ‘species’ continues to represent a difficult problem.

We recently raised a number of important objections to virus taxonomy and Linnaean taxonomy in general that challenge the ‘species’ concept and the taxon-building enterprise [15]. These objections were grouped into three broad categories, which I will briefly describe: (i) taxon definition in light of co-evolution, (ii) impacts of reticulate evolution, and (iii) independent origins breaking up monophyletic relationships.

#### 4.1.1. Most Species Co-Evolve with Others

Most biological species are ‘holobionts’, which are organismal communities organized around individual hosts [67]. These communities behave as units of biological organization. The concept that was originally proposed by Meyer-Abich [68] more than half a century ago recognizes that organisms do not live and evolve in isolation. Instead, they form highly integrated systems that are constantly interacting with the biotic and abiotic environments that surrounds them. They exhibit synergistic phenotypes that impact their genetics, reproduction, physiology, anatomy and behavior [69,70]. Collective impacts on fitness poise coordinated co-evolution, making ‘hologenomes’ comprehensive and integrated gene systems. This challenges the traditional concepts of ‘individuality’ [71] and ‘organismality’ [72], and indirectly, the species concept of taxonomy [15].

All animals and plants are holobionts [73]. They host ‘microbiomes’, which are communities of symbiotic microbes living in close association with the complex multicellular make up of their hosts. These microbes are often highly diverse and include bacteria, archaea, algae, fungi, and protozoa in interaction with viruses. Their numbers often match the cell numbers of their hosts. For example, a typical 70 kg human harbors 3 × 10^13^ human cells and 3.8 × 10^13^ microbial cells weighing 0.2 kg [74]. The makeup of the microbiome is in constant flux and is the subject of extensive transfer of genetic information. For example, more than half of the genes in the genomes of the human microbiome have been transferred at different temporal scales [75]. This ‘genetic crosstalk’ moves microbial genes throughout the human body. This is not surprising. Phylogenetic analyses of sequence alignments of thousands of prokaryotic-eukaryotic clusters of homologs suggested recent HGT is a widespread and continuous process in prokaryotes but not in eukaryotes [76]. Recent studies, however, suggested HGT is more common than expected in eukaryotes. Hundreds of potential foreign genes expressed in primates, flies, nematodes and humans were identified, 145 of which were likely of bacterial origin, as well as others with origins in viruses and yeasts [77]. A more recent study identified 1467 HGT-associated regions of the human genome that were conserved with non-mammals (e.g., birds and fishes), mapped to all chromosomes, and involved 642 known horizontally transferred genes enriched in ion binding [78]. For decades, alignment-dependent methods have identified HGT from prokaryotes to many other eukaryotic species that was different from the well-documented endosymbiotic transfer involving mitochondrial and plastid genes. Reported HGT events included *Wolbachia* gene transfer to insect and nematode hosts, bacterial and fungal gene and bacteria-specific transposon transfer into the telomeres of rotifers, the transfer of fungal genes of carotenoid biosynthesis to aphids, the transfer of bacterial cellulose genes to nematodes, the transfer of bacterial and plant genes to arbuscular mycorrhizal fungi, the transfer of bacterial and archaeal genes to extremophile red algae, and the transfer of *Agrobaterium* tumor-inducing plasmids into plant cells, to name a few examples (reviewed in [79]). All of these events illustrate active HGT and in some cases co-evolution occurring between microbiomes and their hosts. Note, however, that the alignment-dependent methodology has shown numerous limitations. For example, two genome assembly studies of tardigrades, microfauna considered key to understanding the origins of Arthropoda, revealed that genes of bacterial origin represented in one case 16% of the tardigrade gene complement [80] and in another only up to 2% [81]; contamination was claimed as the culprit of such a difference. While the importance of HGT in eukaryotes remains controversial, hologenomes integrate all mechanisms of mutation (prokaryotic and eukaryotic) across many genomes, resulting in covariation and epistasis [73]. In fact, the human gut has been referred to as a ‘*melting pot of genetic exchange*’ [82], which is a statement that could well be extended to many types of holobiont interactions.

Viruses in the form of virions are considered the most abundant taxonomic species on the planet, especially in oceans [83] and freshwater [84], and they are also active and well represented in the microbiomes of holobionts, especially in animals and plants [73,85]. Viruses contribute to the genomic and functional diversity of holobionts, fostering bacterial diversity in microbiomes, hosting immunity mechanisms that prevent pathogenic states and mitigate cancer and other diseases, helping propagate useful traits and protecting from drought and cold, and facilitating processes of molecular innovation. They endogenize and transmit information vertically in host genomes from one generation to the next, sculpting them via retrovirus and transposon activities. Conversely, virus infections transmit and rearrange information horizontally in both microbiomes and hosts but also vertically by persisting in associated microbiomes. The fundamental evolutionary impact of holobiont-integrated viruses can be made evident with two well-known evolutionary leaps: the origin of placental mammals and eukaryogenesis. The mammalian placenta is a specialized organ that facilitates the retention of developing embryos within the reproductive tract of the mother, leading to the release of live offspring. Placentation, which allows the fusion of fetal membranes to the uterine mucosa for physiological exchange, evolved several times in vertebrate taxa 150–200 million years ago [86]. The culprits were repeated recruitments of fusogenic proteins of retroviral origin known as syncytins, which originally allowed the fusion of host cells for viral spreading [87]. Because placenta is the most rapidly evolving mammalian organ, the co-option of endogenous retrovirus-derived genes and gene control elements is likely important and ongoing. Their impact is underscored by the recruitment of syncytins across multiple animal lineages, including marsupials, bats and live-bearing reptiles (e.g., [88]). Remarkably, an analysis of close and distant structural neighbors of the ectodomain of human synsytin-1 revealed that the recruited retroviral fusion core was also recruited in other viruses and surprisingly in bacteria and eukaryotes (Appendix A). There is also growing evidence of viral eukaryogenesis, which is the origin of the eukaryotic cell nucleus from the endosymbiosis of a DNA virus and a prokaryote [89]. One striking example is the identification of nucleus-like structures in bacteria during viral infection [90]. These novel compartments, which resembled viral factories, separated viral DNA of the bacteriophage from the cytoplasm, were centered by a bipolar tubulin-based spindle, and segregated phage and bacterial proteins according to function. Thus, phages are evolving specialized nucleus-like structures to compartmentalize viral replication. These two striking examples of evolutionary leaps show that the evolution of hosts and viruses cannot be easily disentangled and can be crucially affected by the revolutionary appearance of molecular innovations.

#### 4.1.2. Evolution Is Reticulated

Phylogenomics has shown that closely and distantly related species of microbes, plants, insects and vertebrates exhibit reticulate phylogenies that cannot be described with standard phylogenetic trees [91]. Calling the ToL a ‘*rhizome*’ of life [92,93] or a ‘*tela vitae*’ [94] appears more appropriate. One consequence is clearly evident. The pyramidal structures of taxonomy cannot be approximated by evolutionary statements from phylogenetic reconstruction, as demanded by a ‘subsumption’ (specification) hierarchy.

The ‘horizontal’ movement of genetic information stored in nucleic acids between and within diverse organisms is a central process of life. HGT in prokaryotes is a recognized driving evolutionary force that is facilitated by the primary and well-known mechanisms of transformation, transduction and conjugation, which involve direct uptake, virus-mediated transmission, and the cell-to-cell transfer (often mediated by pili) of genetic materials, respectively [79]. Its ubiquity makes the definition of prokaryotic species fuzzy, if not impossible, and demands the construction of pangenomes—entire gene complements of clades rather than of individual species [95]. HGT in eukaryotes is more limited [75], but its presence is significant [96]. HGT, hybridization and introgression, incomplete lineage sorting, recombination, symbiogenesis, and the spread of transposons and gene transfer agents, among many other processes, make the evolution of eukaryotic species reticulated [96]. The separation of somatic tissue and the germ line in multicellular organisms, believed to be a barrier to HGT, seems to be porous to genetic transfer events, especially in unicellular or early stages of organismal development. HGT between prokaryotes and eukaryotes challenges the dogma that the process can only occur between closely related organisms [80]. In fact, there is growing knowledge of its impact across all branches of the ToL. Pangenomes offer the opportunity to capture the diversity of groups of organisms, and the approach has also been applied to eukaryotic organisms. For example, the human pangenome attempts to capture all variants and haplotypes of the human population [97]. Similarly, pangenomes attempt to replace the single reference genomes of crop plants and domestic animals for better insights into domestication, evolution and breeding [98]. They also offer a unique opportunity to capture the genetic diversity of holobionts.

Temporal scales are of importance when studying evolutionary processes and evaluating the validity of phylogeny-reflecting taxonomies. On short enough time scales, HGT and other forms of reticulate evolution are often considered of minimum relevance. In those circumstances, taxon evolution has been approximated with phylogenetic tree statements, and gene phylogenies have been considered acceptable approximations to species phylogenies [16,17]. However, two examples show that reticulation can significantly impact evolution at these short time scales. Whole genome comparisons revealed pervasive genomic mosaicism in phages that infect a single actinobacterial species [99,100]. Both studies uncovered a continuum of genetic diversity in phage populations. The existence of a highly diverse spectrum of relatedness and a constant state of change introduces serious difficulties to the taxonomic classification of bacteriophages. Similarly, recombination processes that are common in RNA viruses have significantly impacted the evolution of coronaviruses, as illustrated by the emergence of SARS-CoV-2 in Wuhan and of its many variants of concern [101]. In addition, the use of gene phylogenies is only valid if phylogenetic character independence is not violated by strong effects of molecular structure on sequence alignments or by the existence of strong historical heterogeneities imposed by the existence of prior molecular states in the sequences of the aligned genes [17]. On longer time scales, the effects of reticulations bring more significant challenges to both phylogenetic reconstruction and the evolutionary validity of higher taxonomic ranks.

Given that the pyramidal structure of taxonomy is incompatible with reticulate evolution, then a temporal directed graph could be used to define levels of biological organization and evolution that would act as ranks and would unify through child terms connected to multiple parents the reticulate evolution of species. This graph would be similar to the directed acyclic graphs of the Gene Ontology (GO) initiative that are used to describe a controlled vocabulary of gene functions [102]. However, building temporal directed graphs describing evolutionary reticulation requires reconstructing phylogenetic networks from sequence and phenotypic data, which is technically and computationally demanding [103,104]. Networks can be quickly generated from distance matrices with popular methods (e.g., NeighborNet [105] used in Figure 4 and Figure 5), but they are inaccurate and do not represent true phylogenetic histories. Alternatively, networks can be reconstructed from weighted triplets, quartets and sextets, which are more phylogenetically informative, using MP and local ML methods (e.g., Quartet-Net [106]) or directly from character data using search methods and optimality criteria (e.g., [107]). These methods are computationally inefficient with performance decreasing with increasing reticulation and number of taxa. They overestimate reticulations or cannot accommodate large numbers of them, especially in deep branches, and the presence of multiple origins compromises the technical feasibility of using phylogenetic relationships. Currently, all of these limitations challenge taxonomic classification and phylogenetic reconstruction.

#### 4.1.3. Independent Origins Break up Monophyletic Relationships

The centrality of monophyly (grouping a common ancestor and its descendants into a ‘clade’) and the rejection of paraphyly (grouping the ancestor and only some of the descendants) remain contested in taxonomy, yet both are tolerated or embraced [108]. However, adopting evolution as a guiding principle in classification implies dividing trees into nested sets of clades while disregarding taxonomic ranks (e.g., families, genera) or accepting both paraphyletic relations and ranks. The coexistence of monophyly and paraphyly is a reality linked to the horizontal and convergent vagaries of reticulate evolution and the impact of genotypic and phenotypic loss. So is polyphyly—an atypical grouping of mixed evolutionary origin that lacks an immediate common ancestor and is overwhelmingly rejected by taxonomists [108]. A ToL assembled by integrating thousands of phylogenies and describing the evolution of ~2.3 million taxa showed patchiness, conflicts, and uncertainties [109]. The evolutionary origins of archaea (including its monophyly), bacteria, early diverging microbial eukaryotes, fungi and animals remained contested or exhibited multiple conflicting resolutions. In this type of studies, instances of independent origin arising from reticulate evolution break up monophyletic relationships. In addition, many ingroup taxa have non-existent, unknown or distant outgroups that question the correct rooting of the trees and the likely origins of individual groups [16]. All of these difficulties complicate taxonomic classification, including the validity of many of its ranks.

The current megataxonomy of viruses embodies a 15-ranked classification system that unifies viruses into six realms and 10 kingdoms [3]. Realms do not share a common ancestor and are therefore polyphyletic. They are believed to have arisen independently in an ancient world of nucleic acid replicators. Their monophyletic nature has been also questioned on multiple grounds (reviewed in [15]). In addition, paraphyletic relationships are widespread in the phylogenetic analysis of viruses. In fact, a ToL that includes viruses reconstructed from structural domain counts in proteomes challenges the separate origin of realms [23]. The tree places viruses at its base as an ensemble of paraphyletic viral groups. Tracing realms and Baltimore classes onto its branches showed that both classification rationales failed to make monophyletic groups [15]. Instead, they appeared spread throughout basal branches as paraphyletic groupings [15].

### 4.2. Are Viruses Self-Standing or Fully-Integrated Biological Systems?

**Figure 7:**
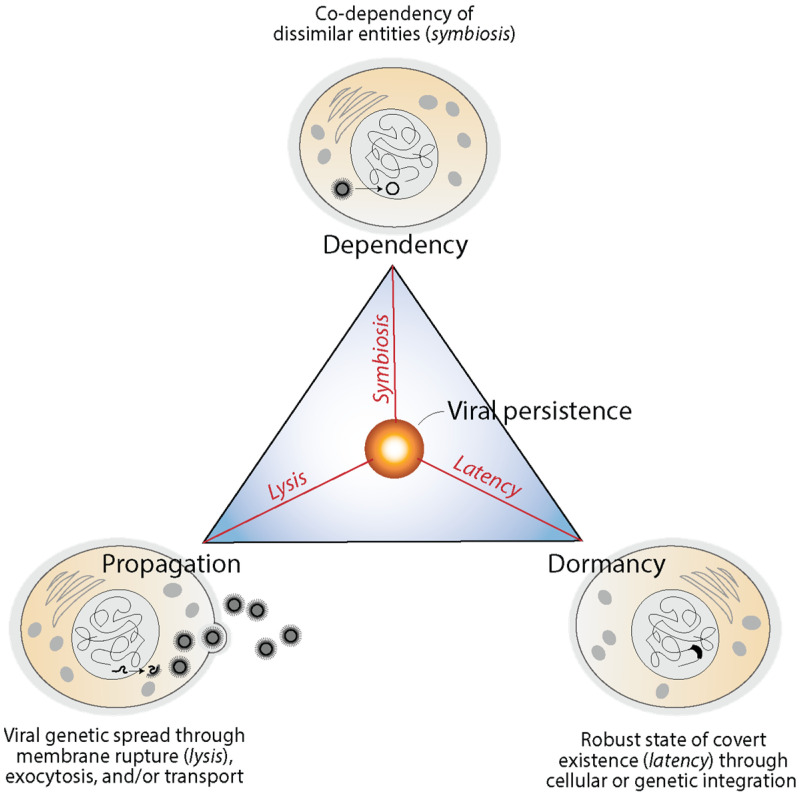
The triangle of viral persistence explains trade-offs between propagation, dependency and dormancy and modes of functional integration of viruses and hosts. Mechanisms are illustrated with influenza infections, herpesvirus latent infections and symbiogenic retroviral integration in human cells, respectively. Modified from [110].

Viruses enter into obligatory intracellular interactions with their hosts and are often endogenized and domesticated, impacting their long-term evolution. They enter into propagation, dependency, and dormancy modes mediated by lysis, symbiosis and latency, respectively [110]. These modes, which achieve different solution goals, have been impactful in the evolution of holobionts and can be illustrated with a triangle of viral persistence (Figure 7). Lysis (and other cellular mechanisms) favors the spread (propagation) of virus genetic material and opportunities for mutational innovation (flexibility through evolutionary plasticity). Spread can be mediated by the destruction of infected cells, budding via exocytosis, or cell-to-cell transport depending on viruses and the biological system. Symbiosis favors intimate association with the host (dependency) by fostering mutualism, commensalism, amensalism, and/or parasitism via economy-driven altruistic, cooperative, or antagonistic behaviors. Finally, latency favors cellular stasis (dormancy) through mechanisms of robustness, generally via episomes or the endogenization of genetic material. The triangle of virus persistence offers a morphospace of trade-offs between flexibility, economy and robustness that highlights how viruses are fully integrated with their hosts and impact their evolution [110].

Virus propagation, dependency and dormancy have tailored genomes in the course of evolution with viruses pushing reductive evolutionary tendencies and hosts enlarging and diversifying their genomic makeup. The impact of viruses in holobiont biology has been recently reviewed [73], including their role in symbiotic relationships [111]. Bacteriophages provide selective advantages when integrated into their bacterial hosts, regulating microbiome populations. They can also improve resistance to environmental stressors, enhance competition by favoring toxin production, provide new cellular functions (e.g., photosynthetic genes in cyanobacteria), produce bacterial biofilms, or enhance metabolic functions. Marine invertebrate viruses engage in symbiogenic interactions that for example foster HGT stability (e.g., chloroplast transfer from algae into sea slugs) or pathogenic control (e.g., coral holobionts). Plant viruses decrease the impact of abiotic stress (e.g., drought and cold tolerance) and biotic insults (e.g., from fungi or insects). Insect viruses enhance the development, fecundity and lifespan of their insect hosts. They enable parasitic interactions (e.g., polydnaviruses and parasitic wasps). Mammalian viruses moderate microbiome dynamics, fend against viral and non-viral diseases, activate innate immunity, protect brain function, and increase mammalian wellness.

In humans, the ‘viriobiota’ engages in parasitic, commensal or mutualistic interactions to establish a delicate balance between the health and disease of the human host [112]. Parasitic viruses causing acute (e.g., influenza virus), chronic (e.g., hepatitis B virus) or latent (e.g., herpesvirus) infections represent a minority compared to those that engage in commensal and mutualistic interactions. Commensal viruses are highly popular, replicate persistently but fail to cause disease (e.g., anelloviruses that live in tissues and blood). Similarly, mutualistic viruses such as bacteriophages regulate the population structure of the microbiome and indirectly impact the well-being of the host. Endogenous retroviruses that are present in the human genome are probably the best examples of impactful mutualistic interactions [111]. They infect germ cells, transmit vertically from parent to off-spring, are widely recycled, and are beneficial to the host [112]. About half of the sequence of the human genome contains transposable elements, of which 2–3% of that half represent DNA transposons and 42–43% comprise retroelements. This massive contribution of viruses to the human genomic makeup is perplexing but explained by physiological impacts, including fetal development and neuroprotection of the brain [112].

Two research examples using mice model systems illustrate the intimate interaction of viruses and their mammalian hosts. The *Herpesviridae* family infects almost all individuals in the human population and can engage in both propagation or latency strategies [113]. Remarkably, lifelong viral infections appear to help protect the host against bacterial infections. Reactivation studies of latent murine gammaherpesvirus 68 showed latency enabled the production of peritoneal macrophages that protected against infection by the intracellular parasite *Listeria monocytogenes* [114]. Similarly, the commensal ‘virome’ also plays an important role in maintaining the front line of defense of the mammalian gut, which is the intraepithelial lymphocytes that confront noxious microbes in the intestinal lumen. Commensal viruses activated an unconventional RIG-1–MAVs–IRF1 signaling pathway that induces cytokine IL-15 in dendritic cells to promote the biogenesis of intraepithelial lymphocytes [115]. These two studies show viruses are key regulators of innate immunity. In fact, viral infections trigger innate immunity signaling pathways that activate tumor suppressors (e.g., p53), suggesting suppressors may have evolved to regulate viral infections and not to control cancer [116]. Viral and cellular oncogenes are therefore integrated to impact host immune functions.

Finally, viruses rely on epigenetic programs for optimal functioning [117], mirroring mechanisms operating in cellular organisms [118]. DNA methylation, for example, is used as an epigenetic modification of DNA to regulate gene expression, silence transposon activity, impact imprinting and development, and modulate restriction–modification. Epigenetic changes such as DNA methylation, but also the modulation of cellular chromatin components, suppress gene activity of the Epstein–Barr virus in latently infected genes by unfolding different latency gene expression programs [119]. In fact, DNA methylation appears a central but diverse viral strategy. The viral genomes of iridoviruses and ascoviruses are highly methylated and encode their own methyltransferases, while the genomes of adenoviruses and polyomaviruses become methylated with latency [120]. These methylations are often site- and promoter-specific and reversible. DNA methylation is also widespread but uneven in giant viruses [121]. Remarkably, these viruses encode methyltransferases that are often evolutionarily conserved, functional, subjected to purifying selection, and transferred by gene exchange to bacteria, viruses and their eukaryotic hosts [121]. The methyltransferases of giant viruses work in conjunction with restriction endonucleases as self-defense or offensive restriction–modification systems against competing pathogens of amoebal hosts. Viral epigenetics is therefore tightly integrated with that of the host to establish propagation, dependency and dormancy modes in the complex holobiont environments.

### 4.3. Are Viruses Mobile Genetic Elements, Microbes or Cellularly Integrated Processes?

The International Committee of Taxonomy of Viruses (ICTV) creates species in accordance with the following definition: “A species is the lowest taxonomic level in the hierarchy approved by the ICTV. A species is a monophyletic group of mobile genetic elements (MGE) whose properties can be distinguished from those of other species by multiple criteria” [122]. These criteria include “natural and experimental host range, cell and tissue tropism, pathogenicity, vector specificity, antigenicity, and the degree of relatedness of their genomes or genes”. An MGE can be understood as a genetic unit that can move within a genome or be transferred from one replicating region (replicon) to another in a same or different organismal species. MGEs take the form of plasmids, transposons, retrotransposons, integrons, introns, viral genomes, and other genetic forms, some of which are dormancy and dependency modes of viral lifestyle and others are completely unrelated replicons. Such broad definition implies ICTV’s desire to broaden virus taxonomy. In doing so, however, the desire challenges the taxonomic unit or species concept of a virus by focusing on the genetic repository (or even the replication mode) instead of the virus life cycle and the associated physiologies. In addition, MGEs are also considered agents of ‘open source evolution’ [123]: ‘mechanisms’ that promote mutational change and enhance evolvability. Considering viruses as MGE-driven molecular mechanisms challenges the integrative aspect of virus biology and the feasibility of their taxonomy. Species defined within a natural classification system that embraces evolution must be both units of biological organization and units of evolution, not solely ‘mobile’ genetic units. They must be persistent entities.

The alternative view of treating viruses as microbial organisms is liberating. It enables researchers to embrace the traditional Linnaean classification scheme with all of its limitations. A microbe shares sets of homologous components (e.g., hallmark or core genes, phenotypes) better justifying phylogenetic and classification methodologies that help assign viruses to taxon membership. This approach, however, is fallacious for viruses. It disentangles the apparently ‘inert’ macromolecular structure and genetics of the virion from virus–host-integrated physiologies operating in viral life cycles (some illustrated in Figure 7), especially in light of holobiont evolution. In addition, a focus on the propagation/replication system that stresses viral genetic spread disregards intracellular parasitic, symbiotic or symbiogenic modes of viral life.

A more meaningful alternative that is in line with virus–host integration is to treat virus species and other taxa as ‘process’ abstractions. This avoids the ‘reification’ fallacy that is common in virology of treating ideas, concepts and properties as physical entities; a species “*cannot be purified by centrifugation, sequenced, visualized by electron microscopy or used to infect a host, since it is an imaginary entity of the mind and not a physical object*” [124]. A ‘processual’ view was first proposed by Burnet [125] in his analysis of the influenza virus: “*So we can catch a glimpse in broad outline of the process by which the new generation of virus particles emerges from the cell. If we look at the process from an even broader point of view, we can perhaps summarize it as a continuing alternation between two modes of life. A virus is not an individual organism in the ordinary sense of the term but something which could almost be called a stream of biological pattern. The pattern is carried from cell to cell by the relatively inert virus particles, but it takes on a new borrowed life from its host at each infection*” [125]. The view was fully endorsed by Lwoff [126], who understood viruses could not be ‘organisms’ or mobile ‘molecules’ but rather something in between. Indeed, as previously discussed, viruses are neither self-standing biological systems nor inanimate molecular (virion) structures (‘rogue’ nuisance elements) that escape from cells. Instead, they are dynamic entities that are better aligned with ontological definitions of biological process. In the GO classification [102], a *biological process* (GO:0008150) is ‘the execution of a genetically-encoded biological module or program’, a *viral process* (GO:0016032) is a ‘multi-organism process in which a virus is a participant’, a *viral life cycle* (GO:0019058) is ‘a set of processes which all viruses follow to ensure survival’, and a *response to virus* (GO:009615) is ‘any process that results in a change in state or activity of a cell or an organism (in terms of movement, secretion, enzyme production, gene expression, etc.) as a result of a stimulus from a virus’. While aspects of these definitions continue to be limited and inaccurate, they represent ontological approximations to a processual and integrative view of viral biology. Claverie has embraced this processual view of virology, and many other colleagues have followed [2,127,128]. They consider a virus a concept rather than a tangible entity. The problem is that if viruses are processes, virus taxonomy faces unsurmountable difficulties. In GO classification, *viral process* is sister to, for example, *metabolic process* (GO:0008152), *growth* (GO:0040007) and *locomotion* (GO:0040011), none of which can be used to build Linnaean-like subsumption taxonomies. Instead, they help build compositional hierarchies (e.g., metabolic pathways in KEGG [129]) that organize the complexity of cells and organisms to understand their evolution. In other words, a viral process is something that unfolds in cells and cannot be disentangled from its mereological underpinning. Thus, if viruses are processes, they cannot be considered taxonomic units.

### 4.4. A Phylogenomic-Centric Assessment

As previously discussed, any long-lasting virus-mediated interaction between organisms that leads to co-evolution in holobionts and microbiomes challenges taxon definition and virus classification. The integration of viruses into the cellular workings of their hosts via inter- or intra-molecular interactions mediating propagation, dependency or dormancy strengthens the concept of a virus being a biological process but diminishes the validity of a virus taxonomy. By the same token, any evolutionary process that generates reticulation complicates taxon definition. This includes the heterogeneous history of prior molecular states that make up gene products such as protein loops and domains. Reticulations impose independent origins, breaking up monophyletic relationships that form the basis of taxonomic ranks. A global evolutionary analysis of the protein world provides important clues about how viruses and cells have interfaced since the beginning of life [20]. Are they self-standing or fully integrated systems at the molecular level? My re-evaluation of previously published phylogenomic data [25,48] shows the protein repertoires of viruses and cells are fully integrated, further challenging the virus taxonomic unit.

A simple comparative proteomic exercise demonstrates viruses spread protein structural domain innovations throughout the cellular world in Archaea, Bacteria and Eukarya (Figure 3). Unfolding the comparative exercise along the chronology of domains dissects the beneficial role of viruses in evolution. Remarkably, the virus-delimited spread of domain innovations was particularly evident during the past 1.5 Gy of protein history once eukaryotes appeared in evolution and VSFs and the associated loop prototypes facilitated viral persistence. The conclusion that viruses are not ‘passive’ contributors to the structure and function of the protein world demands a better definition of viruses. Viruses are not simply mobile mechanisms that spread genetics, i.e., MGEs with self-referential goals. They also move cellular elements throughout the cellular world. This demonstrates cellular integration and the impact of viruses in cellular evolution.

A comparison of the numbers of domains and associated loop prototypes appearing in individual phases of the evolutionary timeline also highlights the important role of viral agents of reticulate evolution and recruitment in protein evolution (Figure 4, Figure 5 and Figure 6; Table 1). Out of all 3892 domain families that are present in 5080 proteomes from viruses and organisms, only 0.85%, 2.44%, 8.32% and 11.51% were specific to Archaea, Viruses, Bacteria and Eukarya, respectively (Figure 4a). Similarly, out of 5125 non-modular loop prototypes that combine to form domain structures, only 0.29%, 1.32%, 4.02% and 4.29% were specific to Archaea, Viruses, Bacteria and Eukarya, respectively (Figure 5a). These minorities plus the fact that 76.9% of domain families and 90.1% of loop prototypes are shared by more than one supergroup already highlights the primacy of recruitment of prior molecular states in evolution. What is unanticipated is the significant evolutionary role of viruses. A total of about 40% and 53% of domains and loops were found to be shared with viruses, respectively. This shows the substantial contribution that viruses make to the protein world and the biosphere. Unfolding the evolution of domains and loops along evolutionary chronologies shows the gradual complexification of that contribution, which started very early in Phase I with the rise of LUCellA and was always substantial (Figure 4b and Figure 5b). The ratio of prototypes to domains along the evolutionary phases of the timeline also revealed that prior molecular states shared with viruses were always larger than those that were unique to cells, especially during the first phases of the timeline (Table 1), again supporting the important role of viruses in enhancing domain formation by the recruitment of loops. Virus and cellular evolution are therefore completely integrated.

Finally, tracing the presence of Venn groups that involve viruses or are unique to cells along the evolutionary timeline showed that most domain and loop structures specific to viruses, Archaea, and Bacteria (but not eukaryotic specific counterparts) or shared between them appeared in Phase IV but were then conspicuously absent in the last phase of protein evolution (the last 0.8 Gy) (Figure 6). This unique evolutionary pattern is consistent with the extensive HGT processes that drive evolution in the microbial world, which would spread prior molecular states across supergroup boundaries. In contrast, the more constrained HGT effects affecting eukaryotic organisms, which host viruses and the microbiomes of emerging holobionts (expected to appear during Phases IV and V), help maintain domains and loops confined to the individual lineages of Eukarya. Venn groups AEV, EV, AE and E that appear in Phase V suggest viruses and archaea help spread eukaryotic innovations. The unrestricted global effect of HGT impacting the microbial superkingdoms and constraints imposed by the complexities of eukaryotic biology reveal the impact of persistent interactions in holobionts.

In summary, viruses appear central to life. They affect many if not all cellular lineages in all three superkingdoms. They share a substantial number of protein domains and loop structures with cells, and in the process, they help spread protein innovations across a wider taxonomic spectrum. Their proteomes are tightly integrated with those of cells at physiological and evolutionary levels with integration being supported by growing knowledge of how viral proteins impinge on their life cycles. The popular question of viruses being alive is moot, and the feasibility of a *natural* virus taxonomy is increasingly unlikely.

## 5. Conclusions and Recommendations

Virus species have been defined as monophyletic groups of MGEs according to the phylogenetic relatedness of their genomes or metagenomes and other intrinsic properties (e.g., host range, antigenicity, pathogenicity) [122]. Monophyletic membership is generally established by alignments of genomic or metagenomic sequences and subsequent phylogenetic analysis. This course of action and the sole use of metagenomic sequences has been criticized by many virologists. An early definition adopted by ICTV in 1991 [130], “*A virus species is a polythetic class of viruses that constitute a replicating lineage and occupy a particular ecological niche*”, has been considered better aligned with relational properties that arise from interactions with hosts and vectors [124]. These polythetic properties are not necessarily present in all members of a species but provide a more robust definition of species membership. The current definition of species (and higher ranks) is therefore limiting, as it focuses mostly on the genetic makeup of the virus and not the integrated complexities of the virion and virus life cycle. Objections and recommendations related to high-level ranks can be found elsewhere [15].

Here, I raise a number of much more important objections to the virus taxonomy enterprise. First, virus species are not monophyletic groups of MGEs. They are not members of a classification of genetic elements or replicons. They are under genetic and epigenetic control and co-evolve with other organisms, including hosts and microbiomes, because they are part of holobionts—units of biological organization. This reality introduces new forms of reticulation that further complicate the use of monophyly as a principle of taxonomic organization. Second, viruses are not self-standing biological systems acting as evolutionary units. They are fully integrated with microbial and cellular biology and are therefore the subject of processes of reticulate evolution, including the recruitment of prior molecular states. They also engage in propagation, dependency and dormancy strategies that impact holobiont evolution. Third, viruses are better described as cellularly integrated processes, which are akin to the many biological processes that are carefully cataloged by the GO Consortium. What makes these viral processes unique is their intermittent spatiotemporal manifestation; i.e., only a segment of holobionts may be affected by specific viral processes in space and at particular timeframes typical of physiology, development, population, and evolutionary dynamics. Considering viruses as processes makes the use of Linnaean-like subsumption hierarchies impossible, because processes are part of systems that must be catalogued with compositional hierarchies that may or may not necessarily use strict containment nesting rationales.

Given unsurmountable difficulties, viruses cannot be at present considered bona fide taxonomic units of a Linnaean-like classification. Other phylogeny-reflecting classification alternatives must be given full consideration. One solution for ICTV classification is to jumpstart collaborative links with the GO Consortium and/or other ontological initiatives that would help place the real complexity of viral processes within the framework of biological systems. These systems must be able to describe the molecular, organismal, and population complexities of the communities that make up the three known superkingdoms of life. I suggest developing compositional hierarchies cataloging viral makeup and initially appending them to a much more conservative ICTV taxonomy that includes only lower-level ranks and uses an operational rationale to name, define and classify viruses.

## Figures and Tables

**Table 1:** Comparison of domains and loop prototypes unique to or shared with viruses and unique to cells that were developing at each of the six phases of the evolutionary timeline. Ratios describe the numerical relationships between emerging prototypes and domains. Evolutionary phases correspond to timeframes described in the chronologies of Figure 4 and Figure 5.

	Evolutionary Phases						
		**0**	**I**	**II**	**III**	**IV**	**V**
**Unique to or shared with viruses**	Prototypes	128	196	495	629	879	535
	Domains	20	47	162	368	670	259
	**Ratio**	6.40	4.17	3.06	1.71	1.31	2.07
**Unique to cells**	Prototypes	0	40	378	656	1146	303
	Domains	0	12	161	450	1445	298
	**Ratio**	–	3.33	2.35	1.46	0.79	1.02

**Figure A1:**
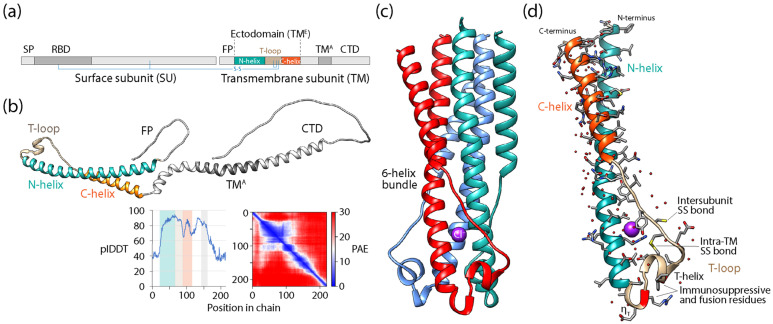
Structural model of the transmembrane ectodomain (TM^E^) of human syncytyn-1 in post-fusion conformation. (**a**) Diagram describing the organization of the syncytin-1 protein with its two subunits. Intra-subunit and inter-subunit disulfide bridges that bind chains together are indicated with blue lines. SP, signal peptide; RBD, receptor-binding domain; FP, fusion peptide; TM^E^, transmembrane ectodomain; TM^A^, transmembrane anchor; CTD, cytoplasmic terminal domain. (**b**) Top-ranking AlphaFold2 model of the transmembrane subunit (TM) generated with the ColabFold c.1.5.5. server [132] (pLDDT = 68.6). The inset shows plots of predicted IDDT (pIDDT) that measure accuracy (superposition-free local fit of all atoms) and predicted alignment error (PAE) that measures confidence in the relative position of residue pairs. (**c**) Structural model of the biological trimeric assembly of the post-fusion ectodomain (TM^E^) with its 6-helix bundle fold (PDB entry: 6RX1-A). (**d**) Protomer showing its three components, the N-helix, the T-loop and the C-helix.

**Figure A2:**
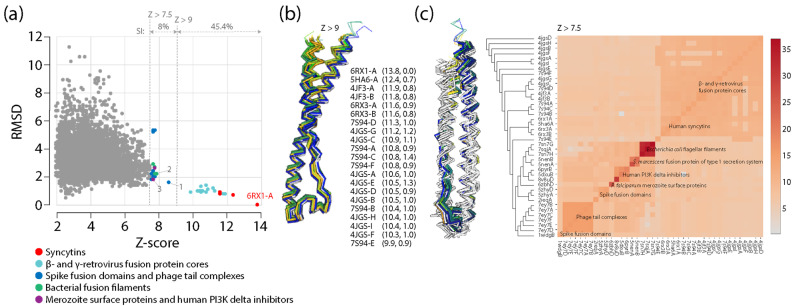
DALI structural neighborhood and best structural alignments to human synsytin-1 in pre-fusion conformation (PDB entry: 6RX1-A). (**a**) An RMSD versus Z-score plot illustrates the neighborhood of 4265 structures with Z ≥ 2 around the 6RX1-A query. The avg. sequence identities (SI) of close and distant structural homologs (identified with colored symbols in the plot) are given in percentages. Examples of distant neighbors: 1. Core structure of transmembrane fusion domain (2IEQ-A) of the spike protein of human coronavirus NK63 [134]; 2. Flagellar filament (7SN7-H) of an enteropathogenic *Escherichia coli* [135]; 3. Surface protein 1 (6ZBH-D) from *Plasmodium falciparum* merozoites [136]. (**b**) C-alpha traces describing the structural alignment of matches at Z-score > 9 show a tight structural fit between the query (light green) and corresponding PDB models (listed together with Z and RMSD values in parentheses). (**c**) C-alpha traces of a structural alignment of the 40 best ranked structures (Z > 7.5) and similarity matrix from an all-against-all comparison.

## Data Availability

The data and information supporting the findings of this study are either public or available within the article and its Supplementary Materials. The AlphaFold2 model presented in Figure A1 has been deposited in ModelArchive (https://www.modelarchive.org, accessed on 25 June 2024).

## Supplementary Materials

The following supporting information can be downloaded at https://www.mdpi.com/article/10.3390/v16071061/s1, Table S1: Times of origin and f-values for structural domain families. Table S2: DALI output file with summary statistics of structures matched to the syncytin-1 query (PDB entry 6RX1-A), Figure S1: Sequence and structural alignment of top-ranked structures (Z > 7.5). File S1: Tree file in *nexus* format encoding a phylogenomic tree of structural domains.

## Appendix A. The Structural Neighborhood of Human Syncytin-1

Syncytins are proteins that drive the fusion of cells in placenta formation [86]. They induce the fusion of peripheral blastocysts into a giant syncytium called syncytiotrophoblast that acts as a unique interface of exchange between maternal and fetal blood during pregnancy. Evolutionarily, syncitins are the products of multiple endogenization events that involved the *env* genes of retroviruses [87]. These genes encode transmembrane class I viral fusion proteins that fold in the endoplasmic reticulum, often trimerize and are activated by a proteolytic-activating step (Figure A1). This step involves a functional element of the hydrophobic structure known as the ‘fusion peptide’, which is located in the C-terminal transmembrane (TM) subunit and facilitates the fusogenic conformational change in the protein. The post-fusion ‘hairpin’ conformation of the TM subunit is known to exist in retroviruses and recently in human syncytins 1 and 2 [131]. The hairpin forms by bringing the N-terminal and C-terminal ends of the TM ectodomain (TM^E^) together, which is a feature that already emerges from the AlphaFold2 modeling of the TM subunit (Figure A1). Note that the hairpin causes the apposition and then merger of the two membranes during the fusion process.

Because structure is much more conserved than sequence [17], I explored the known structural space surrounding the functionally significant segment of syncytins to dissect close and distant structural homologs. Using the fully automated DALI server [133], I generated the structural neighborhood of the TM ectodomain (TM^E^) of human syncytin-1, which is the core fusogenic element of the protein [131]. The server aligned proteins structures non-hierarchically from a pool of 605,846 chains retrieved from PDB (computed 25 May 2024) producing a summary list of structural neighbors from a distance matrix (Table S2). The root-mean square deviation (RMSD) of rigid-body structural superpositions was used to describe three-dimensional (3D) structural similarities. RMSD scores were compared to length-dependent rescaling of the DALI-scores (open scale of structural similarity) in the form of a Z-score (Z), which describes length-rescaled distance matrix alignments that maximize one-to-one atomic correspondences between two structures with a weighted sum of similarities of intramolecular distances. Z provided an optimized measure of alignment length. Note that better structural matches receive lower RMSD scores and longer and better structural matches receive larger Z values. Figure A2a shows RMSD versus Z-score plots describing the structural neighborhood of syncytin-1 in post-fusion conformation (PDB entry: 6rx1_A). As expected, the 20 structural homologs that were closest to the query had RMSD values ranging from 0 to 1.4 and Z ranging from 9.9 to 13.8. These close structural neighbors had sequences and structures that matched those of *env*-encoded fusion protein cores from β- and γ-retroviruses. Figure A2b shows C-alpha traces of structurally aligned atomic models of the 20 closest homologs, revealing very close structural correspondences. Note that the closest homolog was syncytin-1 in post-fusion conformation (5HA6_A; Z = 12.4, RMSD = 0.7) and that syncytin-1 aligned more closely to the γ-retroviral envelope glycoprotein (4JF3_A and 4JF3_A; Z = 11.8–11.9, RMSD = 0.8) than to syncyin-2 (6RX3_A and 6RX3_B; Z = 11.6, RMSD = 0.8–0.9). Figure A2c extends the analysis to the 40 closest structural homologs (Z > 7.5), and Figure S1 shows the associated pairwise sequence and structural alignments without expanding gaps. Remarkably, the more distant structures belonged to fusion segments of spike fusion proteins and phage tail complexes of eukaryotic and bacterial viruses, bacterial fusion filaments and most importantly surface proteins of basal eukaryotes and human phosphoinositide 3-kinase (PI3K) inhibitors (see panel (a) in Figure A2). A similarity matrix from an all-against-all comparison and dendrogram reveals clearly defined structural groups corresponding to the different types of homologs (see panel (c) in Figure A2). These results strongly support the structural recruitment of ectodomain-like structures to perform functions linked to membrane fusion in a vast range of viruses, bacteria and eukaryotes.
