# Supplementary material for: Are Viruses Taxonomic Units? A Protein Domain and Loop-Centric Phylogenomic Assessment
Source: Viruses. 2024 Jun 30;16(7):1061. doi: 10.3390/v16071061 (PMC11281659; doi:10.3390/v16071061)
Supplement: Supplementary file 1 [file viruses-16-01061-s001.zip › Supplementary files/Figure S1.pdf]

viruses

MDPI

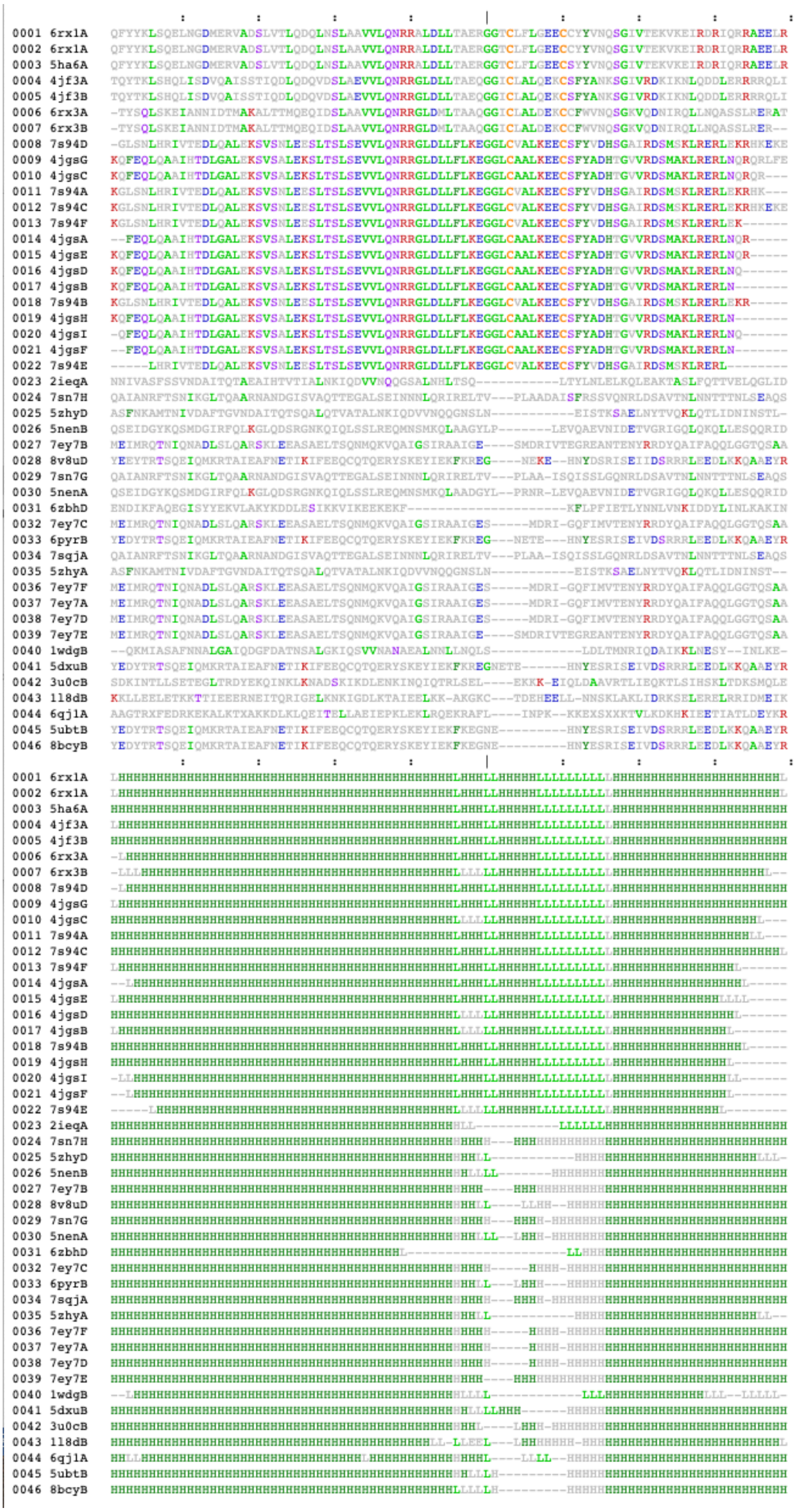

Figure S1. Sequence and structural alignment of top-ranked structures (Z > 7.5). Uppercase letters describe structurally equivalent positions with 6rx1A. Lowercase letters describe insertions. The most frequent amino acid type is colored in each column. DSSP secondary structure assignments: H/h, helix; E/e, strand; L/l: coil.
