# Supplementary material for: Are Viruses Taxonomic Units? A Protein Domain and Loop-Centric Phylogenomic Assessment
Source: Viruses. 2024 Jun 30;16(7):1061. doi: 10.3390/v16071061 (PMC11281659; doi:10.3390/v16071061)
Supplement: Supplementary file 1 [file viruses-16-01061-s001.zip › Supplementary files/Table S2.rtf]

# Table S1. DALI output file with summary statistics of structures matched to the SYNCYTIN-1 query (PDB entry 6rx1-A)# No:  Chain   Z    rmsd lali nres  %id PDB  Description   1:  6rx1-A 13.8  0.0   89    89  100   MOLECULE: SYNCYTIN-1;                                                   2:  5ha6-A 12.4  0.7   89    93   99   MOLECULE: SYNCYTIN-1;                                                   3:  4jf3-A 11.9  0.8   89    90   52   MOLECULE: ENVELOPE GLYCOPROTEIN;                                        4:  4jf3-B 11.8  0.8   89    92   52   MOLECULE: ENVELOPE GLYCOPROTEIN;                                        5:  6rx3-A 11.6  0.9   88    89   45   MOLECULE: SYNCYTIN-2;                                                   6:  6rx3-B 11.6  0.8   86    86   47   MOLECULE: SYNCYTIN-2;                                                   7:  7s94-D 11.3  1.0   88    90   38   MOLECULE: ENDOGENOUS RETROVIRUS GROUP S71 MEMBER 1 ENV POLY             8:  4jgs-G 11.2  1.2   89    90   35   MOLECULE: MLV-RELATED PROVIRAL ENV POLYPROTEIN;                         9:  4jgs-C 10.9  1.1   86    88   36   MOLECULE: MLV-RELATED PROVIRAL ENV POLYPROTEIN;                        10:  7s94-A 10.8  0.9   86    92   37   MOLECULE: ENDOGENOUS RETROVIRUS GROUP S71 MEMBER 1 ENV POLY            11:  7s94-C 10.8  1.4   89    97   37   MOLECULE: ENDOGENOUS RETROVIRUS GROUP S71 MEMBER 1 ENV POLY            12:  7s94-F 10.8  0.9   83    83   37   MOLECULE: ENDOGENOUS RETROVIRUS GROUP S71 MEMBER 1 ENV POLY            13:  4jgs-A 10.6  1.0   82    82   38   MOLECULE: MLV-RELATED PROVIRAL ENV POLYPROTEIN;                        14:  4jgs-E 10.5  1.3   84    84   37   MOLECULE: MLV-RELATED PROVIRAL ENV POLYPROTEIN;                        15:  4jgs-D 10.5  0.9   83    85   36   MOLECULE: MLV-RELATED PROVIRAL ENV POLYPROTEIN;                        16:  4jgs-B 10.5  1.0   82    82   37   MOLECULE: MLV-RELATED PROVIRAL ENV POLYPROTEIN;                        17:  7s94-B 10.4  1.0   84    89   38   MOLECULE: ENDOGENOUS RETROVIRUS GROUP S71 MEMBER 1 ENV POLY            18:  4jgs-H 10.4  1.0   82    84   37   MOLECULE: MLV-RELATED PROVIRAL ENV POLYPROTEIN;                        19:  4jgs-I 10.4  1.0   82    82   37   MOLECULE: MLV-RELATED PROVIRAL ENV POLYPROTEIN;                        20:  4jgs-F 10.3  1.0   80    80   38   MOLECULE: MLV-RELATED PROVIRAL ENV POLYPROTEIN;                        21:  7s94-E  9.9  0.9   76    76   41   MOLECULE: ENDOGENOUS RETROVIRUS GROUP S71 MEMBER 1 ENV POLY            22:  2ieq-A  8.6  1.6   78    88   18   MOLECULE: SPIKE GLYCOPROTEIN;                                          23:  7sn7-H  7.9  2.2   86   546    6   MOLECULE: FLAGELLIN;                                                   24:  5zhy-D  7.8  2.0   77    90   12   MOLECULE: SPIKE GLYCOPROTEIN, SPIKE GLYCOPROTEIN;                      25:  5nen-B  7.8  2.7   82   213    5   MOLECULE: LIPASE C;                                                    26:  7ey7-B  7.8  5.3   85   102    6   MOLECULE: TAIL FIBER PROTEIN;                                          27:  8v8u-D  7.8  2.6   83   279    8   MOLECULE: PHOSPHATIDYLINOSITOL 4,5-BISPHOSPHATE 3-KINASE CA            28:  7sn7-G  7.8  2.2   85   546    6   MOLECULE: FLAGELLIN;                                                   29:  5nen-A  7.7  2.9   86   266    5   MOLECULE: LIPASE C;                                                    30:  6zbh-D  7.7  1.8   68   201   12   MOLECULE: MEROZOITE SURFACE ANTIGENS;                                  31:  7ey7-C  7.7  5.2   83   102    6   MOLECULE: TAIL FIBER PROTEIN;                                          32:  6pyr-B  7.7  2.4   83   163    7   MOLECULE: PHOSPHATIDYLINOSITOL 4,5-BISPHOSPHATE 3-KINASE CA            33:  7sqj-A  7.7  2.2   85   546    6   MOLECULE: FLAGELLIN;                                                   34:  5zhy-A  7.7  2.0   76    90   11   MOLECULE: SPIKE GLYCOPROTEIN, SPIKE GLYCOPROTEIN;                      35:  7ey7-F  7.7  5.2   83   102    6   MOLECULE: TAIL FIBER PROTEIN;                                          36:  7ey7-A  7.7  5.2   83   102    6   MOLECULE: TAIL FIBER PROTEIN;                                          37:  7ey7-D  7.7  5.2   83   102    6   MOLECULE: TAIL FIBER PROTEIN;                                          38:  7ey7-E  7.7  5.3   85   102    6   MOLECULE: TAIL FIBER PROTEIN;                                          39:  1wdg-B  7.6  1.8   72    74   14   MOLECULE: E2 GLYCOPROTEIN;                                             40:  5dxu-B  7.6  2.2   83   169    7   MOLECULE: PHOSPHATIDYLINOSITOL 4,5-BISPHOSPHATE 3-KINASE CA            41:  3u0c-B  7.5  2.8   84   151    8   MOLECULE: INVASIN IPAB;                                                42:  1l8d-B  7.5  2.5   84   102   10   MOLECULE: DNA DOUBLE-STRAND BREAK REPAIR RAD50 ATPASE;                 43:  6qj1-A  7.5  2.6   83   406   10   MOLECULE: STRUCTURAL MAINTENANCE OF CHROMOSOMES PROTEIN,STR            44:  5ubt-B  7.5  2.0   80   163    8   MOLECULE: PHOSPHATIDYLINOSITOL 4,5-BISPHOSPHATE 3-KINASE CA            45:  8bcy-B  7.5  2.0   80   165    8   MOLECULE: PHOSPHATIDYLINOSITOL 4,5-BISPHOSPHATE 3-KINASE CA            46:  1l8d-A  7.4  2.5   83   103   12   MOLECULE: DNA DOUBLE-STRAND BREAK REPAIR RAD50 ATPASE;                 47:  8ub3-A  7.4  1.9   67   224    4   MOLECULE: DPHF7 FILAMENT;                                              48:  4ilo-A  7.4  2.5   84   236   12   MOLECULE: CT398;                                                       49:  3na7-A  7.4  2.8   80   237    6   MOLECULE: HP0958;                                                      50:  6vy1-f  7.4  3.1   82   121    9   MOLECULE: PREFOLDIN SUBUNIT ALPHA 2;                                   51:  8cvi-S  7.4  2.2   86   269    6   MOLECULE: FLAGELLIN;                                                   52:  8h37-J  7.4  2.7   83   168    7   MOLECULE: CULLIN-3;                                                    53:  7c53-A  7.4  2.9   80    93   19   MOLECULE: SPIKE PROTEIN S2',PAN-COVS INHIBITOR EK1;                    54:  6ocu-B  7.4  2.5   83   164    8   MOLECULE: PHOSPHATIDYLINOSITOL 4,5-BISPHOSPHATE 3-KINASE CA            55:  6njn-E  7.3  1.4   61   117    0   MOLECULE: GLUTAMATE RECEPTOR 1;                                        56:  4ilo-B  7.3  2.3   84   236   12   MOLECULE: CT398;                                                       57:  4lws-A  7.3  2.3   79   100    4   MOLECULE: UNCHARACTERIZED PROTEIN;                                     58:  8h37-H  7.3  2.8   83   168    7   MOLECULE: CULLIN-3;                                                    59:  6m1v-A  7.3  2.6   81    91   19   MOLECULE: SPIKE PROTEIN;                                               60:  4dci-B  7.2  3.0   78   148    9   MOLECULE: UNCHARACTERIZED PROTEIN;                                     61:  5tqy-A  7.2  2.1   81   578    7   MOLECULE: INHIBITOR OF NUCLEAR FACTOR KAPPA-B KINASE SUBUNI            62:  7p5m-B  7.2  2.4   79   394    6   MOLECULE: PROTEIN TWEETY HOMOLOG 2;                                    63:  2zdi-B  7.2  2.6   82   106    2   MOLECULE: PREFOLDIN SUBUNIT BETA;                                      64:  8ub3-M  7.2  1.5   65   224    6   MOLECULE: DPHF7 FILAMENT;                                              65:  8ub3-J  7.2  1.5   65   224    6   MOLECULE: DPHF7 FILAMENT;                                              66:  8ub3-E  7.2  1.5   65   224    6   MOLECULE: DPHF7 FILAMENT;                                              67:  8ub3-B  7.2  1.5   65   224    6   MOLECULE: DPHF7 FILAMENT;                                              68:  5xei-A  7.2  2.6   85   517    8   MOLECULE: CHROMOSOME PARTITION PROTEIN SMC;                            69:  6o35-B  7.2  4.3   86   100    8   MOLECULE: DE NOVO DESIGNED WSHC8;                                      70:  5itd-B  7.2  2.1   85   237    8   MOLECULE: PHOSPHATIDYLINOSITOL 4,5-BISPHOSPHATE 3-KINASE CA            71:  6b87-C  7.2  3.1   83   100    8   MOLECULE: TMHC2_E;                                                     72:  8h36-H  7.2  2.8   83   379    7   MOLECULE: E3 UBIQUITIN-PROTEIN LIGASE RBX1;                            73:  7nyy-B  7.2  2.1   85  1467    6   MOLECULE: CHROMOSOME PARTITION PROTEIN MUKB;                           74:  5dxh-B  7.2  2.2   84   121    8   MOLECULE: PHOSPHATIDYLINOSITOL 4,5-BISPHOSPHATE 3-KINASE CA            75:  8cye-L  7.2  2.7   83   269    8   MOLECULE: FLAGELLIN;                                                   76:  6u1s-A  7.1  3.6   82   310    7   MOLECULE: DE NOVO DESIGNED 16-HELIX TRANSMEMBRANE NANOPORE,            77:  8j2f-A  7.1  2.5   84   384    8   MOLECULE: SPHINGOMYELIN PHOSPHODIESTERASE 2;                           78:  4dci-C  7.1  3.4   78   148    9   MOLECULE: UNCHARACTERIZED PROTEIN;                                     79:  6dkm-H  7.1  2.1   67    72    7   MOLECULE: DHD131_A;                                                    80:  6b87-D  7.1  3.4   83   102   10   MOLECULE: TMHC2_E;                                                     81:  6njm-E  7.1  1.4   61   117    0   MOLECULE: GLUTAMATE RECEPTOR 3;                                        82:  3p8c-F  7.1  2.6   77   156    4   MOLECULE: CYTOPLASMIC FMR1-INTERACTING PROTEIN 1;                      83:  4n78-F  7.1  2.8   77   156    3   MOLECULE: CYTOPLASMIC FMR1-INTERACTING PROTEIN 1;                      84:  6u1s-B  7.1  3.7   81   310    7   MOLECULE: DE NOVO DESIGNED 16-HELIX TRANSMEMBRANE NANOPORE,            85:  6u1s-F  7.1  3.7   81   310    7   MOLECULE: DE NOVO DESIGNED 16-HELIX TRANSMEMBRANE NANOPORE,            86:  6g6w-B  7.1  2.8   85   153    4   MOLECULE: PHOSPHATIDYLINOSITOL 4,5-BISPHOSPHATE 3-KINASE CA            87:  6u1s-D  7.1  3.8   80   310    9   MOLECULE: DE NOVO DESIGNED 16-HELIX TRANSMEMBRANE NANOPORE,            88:  6u1s-H  7.1  3.8   80   310    9   MOLECULE: DE NOVO DESIGNED 16-HELIX TRANSMEMBRANE NANOPORE,            89:  8j2f-B  7.1  2.5   84   384    8   MOLECULE: SPHINGOMYELIN PHOSPHODIESTERASE 2;                           90:  5dxh-E  7.1  2.1   84   125    8   MOLECULE: PHOSPHATIDYLINOSITOL 4,5-BISPHOSPHATE 3-KINASE CA            91:  8cvi-a  7.1  2.6   83   268    8   MOLECULE: FLAGELLIN;                                                   92:  6u1s-L  7.1  3.8   80   310    9   MOLECULE: DE NOVO DESIGNED 16-HELIX TRANSMEMBRANE NANOPORE,            93:  6u1s-J  7.1  3.7   81   310    7   MOLECULE: DE NOVO DESIGNED 16-HELIX TRANSMEMBRANE NANOPORE,            94:  6o35-C  7.1  4.2   86   100    8   MOLECULE: DE NOVO DESIGNED WSHC8;                                      95:  6oco-B  7.1  2.9   86   170    3   MOLECULE: PHOSPHATIDYLINOSITOL 4,5-BISPHOSPHATE 3-KINASE CA            96:  6o35-A  7.1  4.3   86   101    8   MOLECULE: DE NOVO DESIGNED WSHC8;                                      97:  6u1s-N  7.1  3.7   81   310    7   MOLECULE: DE NOVO DESIGNED 16-HELIX TRANSMEMBRANE NANOPORE,            98:  8w9b-B  7.1  2.5   82   276    7   MOLECULE: PHOSPHATIDYLINOSITOL 4,5-BISPHOSPHATE 3-KINASE CA            99:  5ha6-B  7.0  1.8   72    79    6   MOLECULE: SYNCYTIN-1;                                                 100:  6dkm-D  7.0  1.8   67    79    6   MOLECULE: DHD131_A;                                                   101:  5j0l-A  7.0  5.3   78   130   15   MOLECULE: DESIGNED PROTEIN 3L6HC2_2;                                  102:  3fx7-B  7.0  2.1   73    87   10   MOLECULE: PUTATIVE UNCHARACTERIZED PROTEIN;                           103:  2ieq-B  7.0  2.6   78    89    6   MOLECULE: SPIKE GLYCOPROTEIN;                                         104:  6vy1-A  7.0  2.8   78   121   14   MOLECULE: PREFOLDIN SUBUNIT ALPHA 2;                                  105:  6z6o-C  7.0  2.7   85   548    1   MOLECULE: HISTONE DEACETYLASE HDA1;                                   106:  2ieq-C  7.0  2.7   78    88    6   MOLECULE: SPIKE GLYCOPROTEIN;                                         107:  6o35-D  7.0  4.2   85    99    8   MOLECULE: DE NOVO DESIGNED WSHC8;                                     108:  7uh4-D  7.0  4.5   86   110   13   MOLECULE: LXG-ASSOCIATED ALPHA-HELICAL PROTEIN D2;                    109:  6wnr-J  7.0  3.5   69    77    9   MOLECULE: ATP SYNTHASE SUBUNIT DELTA;                                 110:  7c53-E  7.0  2.4   79    93   19   MOLECULE: SPIKE PROTEIN S2',PAN-COVS INHIBITOR EK1;                   111:  7c53-B  7.0  2.9   78    93   19   MOLECULE: SPIKE PROTEIN S2',PAN-COVS INHIBITOR EK1;                   112:  6vy1-B  7.0  2.7   78   121   14   MOLECULE: PREFOLDIN SUBUNIT ALPHA 2;                                  113:  6dkm-B  7.0  1.8   66    74    6   MOLECULE: DHD131_A;                                                   114:  7lq1-B  7.0  2.9   86   169    3   MOLECULE: PHOSPHATIDYLINOSITOL 4,5-BISPHOSPHATE 3-KINASE CA           115:  6u28-D  7.0  2.8   77   144    5   MOLECULE: NON-STRUCTURAL PROTEIN 1;                                   116:  6vy1-E  7.0  2.7   78   121   14   MOLECULE: PREFOLDIN SUBUNIT ALPHA 2;                                  117:  6vy1-G  7.0  2.8   82   121    9   MOLECULE: PREFOLDIN SUBUNIT ALPHA 2;                                  118:  5xgi-B  7.0  2.8   82   277    5   MOLECULE: PHOSPHATIDYLINOSITOL 4,5-BISPHOSPHATE 3-KINASE CA           119:  7c53-D  7.0  2.1   74    89   20   MOLECULE: SPIKE PROTEIN S2',PAN-COVS INHIBITOR EK1;                   120:  5izs-D  6.9  3.8   73    82    4   MOLECULE: DESIGNED PROTEIN 5L6HC3_1;                                  121:  7zr1-D  6.9  2.2   83   780    7   MOLECULE: DOUBLE-STRAND BREAK REPAIR PROTEIN;                         122:  6b87-B  6.9  3.1   79    96   11   MOLECULE: TMHC2_E;                                                    123:  1cxz-B  6.9  1.5   66    86    6   MOLECULE: PROTEIN (HIS-TAGGED TRANSFORMING PROTEIN RHOA(0-            124:  5ip0-A  6.9  3.9   82   107    7   MOLECULE: PHA GRANULE-ASSOCIATED PROTEIN;                             125:  8a1g-C  6.9  1.9   81   184    7   MOLECULE: SORTING NEXIN-1;                                            126:  4ry2-A  6.9  4.5   80   701    4   MOLECULE: ABC-TYPE BACTERIOCIN TRANSPORTER;                           127:  8px9-B  6.9  4.1   82   567    9   MOLECULE: MICROCIN-J25 EXPORT ATP-BINDING/PERMEASE PROTEIN            128:  3fav-C  6.9  2.5   79    90   10   MOLECULE: ESAT-6-LIKE PROTEIN ESXB;                                   129:  5ayh-A  6.9  2.7   86   254    3   MOLECULE: CYTOPLASMIC DYNEIN 1 HEAVY CHAIN 1;                         130:  6ois-C  6.9  2.7   84   330    8   MOLECULE: PROTEIN RDM1;                                               131:  5ip0-I  6.9  3.9   82   107    7   MOLECULE: PHA GRANULE-ASSOCIATED PROTEIN;                             132:  7uh4-B  6.9  4.3   83   109   12   MOLECULE: LXG-ASSOCIATED ALPHA-HELICAL PROTEIN D2;                    133:  7c53-F  6.9  2.1   76    92   20   MOLECULE: SPIKE PROTEIN S2',PAN-COVS INHIBITOR EK1;                   134:  6vy1-b  6.9  2.7   77   121   14   MOLECULE: PREFOLDIN SUBUNIT ALPHA 2;                                  135:  6rx3-C  6.8  2.3   84    99   12   MOLECULE: SYNCYTIN-2;                                                 136:  8cqn-B  6.8  2.9   79   187    9   MOLECULE: LIPOPROTEIN, PUTATIVE;                                      137:  7nyx-A  6.8  2.2   82  1467    6   MOLECULE: CHROMOSOME PARTITION PROTEIN MUKB;                          138:  1x04-A  6.8  1.6   76   200   14   MOLECULE: SH3-CONTAINING GRB2-LIKE PROTEIN 2;                         139:  5ip0-M  6.8  4.0   82   107    7   MOLECULE: PHA GRANULE-ASSOCIATED PROTEIN;                             140:  3r84-E  6.8  1.4   62    73    6   MOLECULE: MEDIATOR OF RNA POLYMERASE II TRANSCRIPTION SUBUN           141:  5j9u-D  6.8  4.2   84   120    2   MOLECULE: HISTONE ACETYLTRANSFERASE ESA1;                             142:  6ucb-H  6.8  3.5   67   134    6   MOLECULE: GLUTAMATE RECEPTOR 2;                                       143:  5j9t-H  6.8  4.1   80   119    1   MOLECULE: HISTONE ACETYLTRANSFERASE ESA1;                             144:  6vy1-g  6.8  2.9   78   121   14   MOLECULE: PREFOLDIN SUBUNIT ALPHA 2;                                  145:  3g67-A  6.8  2.1   83   213    5   MOLECULE: METHYL-ACCEPTING CHEMOTAXIS PROTEIN;                        146:  6oqr-M  6.8  3.5   69    77    9   MOLECULE: ATP SYNTHASE SUBUNIT DELTA;                                 147:  5fd9-A  6.8  1.9   73   125    7   MOLECULE: VACUOLAR-SORTING PROTEIN SNF7;                              148:  6djl-B  6.8  2.8   86   221    7   MOLECULE: RAS-RELATED PROTEIN RAB-11A;                                149:  7v5c-A  6.8  4.5   80   572    5   MOLECULE: ABC-TYPE OLIGOPEPTIDE TRANSPORTER ABCB9;                    150:  7jis-B  6.8  2.8   86   163    3   MOLECULE: PHOSPHATIDYLINOSITOL 4,5-BISPHOSPHATE 3-KINASE CA           151:  6vy1-e  6.8  2.8   78   121   14   MOLECULE: PREFOLDIN SUBUNIT ALPHA 2;                                  152:  6vy1-C  6.8  2.8   78   121   14   MOLECULE: PREFOLDIN SUBUNIT ALPHA 2;                                  153:  6vy1-d  6.8  2.7   78   121   14   MOLECULE: PREFOLDIN SUBUNIT ALPHA 2;                                  154:  6vy1-F  6.8  2.8   78   121   14   MOLECULE: PREFOLDIN SUBUNIT ALPHA 2;                                  155:  5j0l-B  6.8  5.2   75   120   15   MOLECULE: DESIGNED PROTEIN 3L6HC2_2;                                  156:  2i1k-A  6.7  2.0   72   545   11   MOLECULE: MOESIN;                                                     157:  6zff-A  6.7  2.8   83   126   11   MOLECULE: DNA DOUBLE-STRAND BREAK REPAIR RAD50 ATPASE;                158:  6vhf-A  6.7  2.7   87   140    7   MOLECULE: PHD-TYPE DOMAIN-CONTAINING PROTEIN;                         159:  6oit-F  6.7  3.2   81   342    2   MOLECULE: PROTEIN RDM1;                                               160:  6jfk-A  6.7  3.3   82   428    7   MOLECULE: MITOFUSIN-2,CDNA FLJ57997, HIGHLY SIMILAR TO TRAN           161:  3n27-B  6.7  1.6   66    79   11   MOLECULE: FUSION GLYCOPROTEIN F0, LINKER, FUSION GLYCOPROTE           162:  3r84-W  6.7  1.4   62    73    6   MOLECULE: MEDIATOR OF RNA POLYMERASE II TRANSCRIPTION SUBUN           163:  6b87-A  6.7  3.1   81   100    9   MOLECULE: TMHC2_E;                                                    164:  5wkq-B  6.7  2.5   81   157    6   MOLECULE: INVASIN IPAB;                                               165:  6yvv-B  6.7  2.9   74   480   12   MOLECULE: STRUCTURAL MAINTENANCE OF CHROMOSOMES PROTEIN 2,S           166:  6wnr-P  6.7  3.4   70    77    9   MOLECULE: ATP SYNTHASE SUBUNIT DELTA;                                 167:  6oqs-I  6.7  3.5   69    77    9   MOLECULE: ATP SYNTHASE SUBUNIT DELTA;                                 168:  6vwk-L  6.7  3.6   69    77    9   MOLECULE: ATP SYNTHASE SUBUNIT C;                                     169:  6wa9-N  6.7  3.0   75    86   16   MOLECULE: LOW CALCIUM RESPONSE LOCUS PROTEIN D;                       170:  5t8n-A  6.7  2.0   73   125    7   MOLECULE: VACUOLAR-SORTING PROTEIN SNF7;                              171:  6vy1-D  6.7  2.7   78   121   14   MOLECULE: PREFOLDIN SUBUNIT ALPHA 2;                                  172:  6pyu-B  6.7  2.7   82   160    4   MOLECULE: PHOSPHATIDYLINOSITOL 4,5-BISPHOSPHATE 3-KINASE CA           173:  6d05-F  6.7  3.2   84   466    8   MOLECULE: TRANSFERRIN RECEPTOR PROTEIN 1;                             174:  5izs-F  6.7  3.8   71    80    4   MOLECULE: DESIGNED PROTEIN 5L6HC3_1;                                  175:  3fx7-A  6.7  2.2   73    84   10   MOLECULE: PUTATIVE UNCHARACTERIZED PROTEIN;                           176:  1wnc-B  6.7  2.7   76    81   20   MOLECULE: E2 GLYCOPROTEIN;                                            177:  8h37-I  6.7  2.8   83   168    7   MOLECULE: CULLIN-3;                                                   178:  8idd-E  6.7  2.5   84   159   10   MOLECULE: CELL DIVISION ATP-BINDING PROTEIN FTSE;                     179:  6d05-E  6.7  3.2   84   466    8   MOLECULE: TRANSFERRIN RECEPTOR PROTEIN 1;                             180:  6x80-H  6.7  2.6   83   574    7   MOLECULE: FLAGELLIN A;                                                181:  5ip0-D  6.6  3.6   78   108    6   MOLECULE: PHA GRANULE-ASSOCIATED PROTEIN;                             182:  8e4g-a  6.6  4.7   85   127    7   MOLECULE: PORTAL PROTEIN;                                             183:  7z1n-O  6.6  5.1   77   570    6   MOLECULE: DNA-DIRECTED RNA POLYMERASE III SUBUNIT RPC1;               184:  1wdf-B  6.6  2.6   80    93    9   MOLECULE: E2 GLYCOPROTEIN;                                            185:  3r84-O  6.6  1.4   62    76    6   MOLECULE: MEDIATOR OF RNA POLYMERASE II TRANSCRIPTION SUBUN           186:  8tzk-D  6.6  2.9   73   304    7   MOLECULE: CELL DIVISION ATP-BINDING PROTEIN FTSE;                     187:  5z7b-A  6.6  3.5   68   196   10   MOLECULE: PADR FAMILY TRANSCRIPTIONAL REGULATOR;                      188:  1fzc-A  6.6  2.5   67    74   10   MOLECULE: FIBRIN;                                                     189:  1ebo-A  6.6  2.5   79   112   14   MOLECULE: EBOLA VIRUS ENVELOPE PROTEIN CHIMERA CONSISTING             190:  2y3a-B  6.6  2.6   82   236    7   MOLECULE: PHOSPHATIDYLINOSITOL-4,5-BISPHOSPHATE 3-KINASE CA           191:  6oqt-Q  6.6  3.5   69    77    9   MOLECULE: ATP SYNTHASE SUBUNIT DELTA;                                 192:  5j10-B  6.6  2.2   63    70    6   MOLECULE: PEPTIDE DESIGN 2L4HC2_24;                                   193:  6lxt-E  6.6  1.6   78   114   14   MOLECULE: 2019-NCOV S2 SUBUNIT,2019-NCOV S2 SUBUNIT;                  194:  8v8i-B  6.6  2.0   84   212    8   MOLECULE: PHOSPHATIDYLINOSITOL 4,5-BISPHOSPHATE 3-KINASE CA           195:  3ze5-A  6.6  3.0   67   115    3   MOLECULE: DIACYLGLYCEROL KINASE;                                      196:  4ry2-B  6.6  4.6   80   699    5   MOLECULE: ABC-TYPE BACTERIOCIN TRANSPORTER;                           197:  6s1k-L  6.6  2.5   80   102    9   MOLECULE: CHEMOTAXIS PROTEIN CHEA;                                    198:  6el1-R  6.6  3.3   77   332   12   MOLECULE: YAXA;                                                       199:  6z6h-I  6.6  3.0   81   541    2   MOLECULE: HISTONE DEACETYLASE HDA1;                                   200:  6vy1-a  6.6  2.7   76   120   14   MOLECULE: PREFOLDIN SUBUNIT ALPHA 2;                                  201:  6zff-B  6.6  2.9   85   124   11   MOLECULE: DNA DOUBLE-STRAND BREAK REPAIR RAD50 ATPASE;                202:  6z6h-C  6.6  3.0   81   541    2   MOLECULE: HISTONE DEACETYLASE HDA1;                                   203:  6x80-L  6.6  2.8   84   574    7   MOLECULE: FLAGELLIN A;                                                204:  8h37-G  6.6  2.4   83   168    4   MOLECULE: CULLIN-3;                                                   205:  5ip0-L  6.6  3.6   78   108    6   MOLECULE: PHA GRANULE-ASSOCIATED PROTEIN;                             206:  3hr0-A  6.5  2.1   75   250    7   MOLECULE: COG4;                                                       207:  6x80-C  6.5  2.3   77   574    8   MOLECULE: FLAGELLIN A;                                                208:  3r84-I  6.5  1.5   62    80    5   MOLECULE: MEDIATOR OF RNA POLYMERASE II TRANSCRIPTION SUBUN           209:  5ip0-P  6.5  4.5   83   108    7   MOLECULE: PHA GRANULE-ASSOCIATED PROTEIN;                             210:  3r84-M  6.5  1.4   62    80    6   MOLECULE: MEDIATOR OF RNA POLYMERASE II TRANSCRIPTION SUBUN           211:  3r84-U  6.5  1.4   62    81    5   MOLECULE: MEDIATOR OF RNA POLYMERASE II TRANSCRIPTION SUBUN           212:  3r84-G  6.5  1.4   62    79    6   MOLECULE: MEDIATOR OF RNA POLYMERASE II TRANSCRIPTION SUBUN           213:  3r84-C  6.5  1.4   62    80    6   MOLECULE: MEDIATOR OF RNA POLYMERASE II TRANSCRIPTION SUBUN           214:  5kpj-A  6.5  2.5   80  1177    5   MOLECULE: MULTIDRUG RESISTANCE PROTEIN 1A;                            215:  7p3r-D  6.5  2.4   82   341    7   MOLECULE: MAKA TETRAMER;                                              216:  8h7e-A  6.5  2.4   79    91   13   MOLECULE: DE NOVO FERRIC ENTEROBACTIN ESTERASE SYN-F4;                217:  1qr9-A  6.5  2.1   63    68   11   MOLECULE: GP41 ENVELOPE PROTEIN;                                      218:  2hpc-D  6.5  2.5   67    71   10   MOLECULE: FIBRINOGEN ALPHA CHAIN;                                     219:  7uh4-A  6.5  4.4   86   110   13   MOLECULE: LXG-ASSOCIATED ALPHA-HELICAL PROTEIN D2;                    220:  8gmh-C  6.5  2.1   85   138    2   MOLECULE: LXG DOMAIN-CONTAINING PROTEIN;                              221:  7sn7-a  6.5  2.2   86   546    6   MOLECULE: FLAGELLIN;                                                  222:  4pl0-B  6.5  4.4   83   576   10   MOLECULE: MICROCIN-J25 EXPORT ATP-BINDING/PERMEASE PROTEIN            223:  4afl-B  6.5  3.4   77    98   12   MOLECULE: INHIBITOR OF GROWTH PROTEIN 4;                              224:  3ze5-B  6.5  3.0   67   116    3   MOLECULE: DIACYLGLYCEROL KINASE;                                      225:  5jpq-R  6.5  1.7   67   332    0   MOLECULE: WD40 DOMAIN PROTEINS;                                       226:  6jy0-M  6.5  2.4   83   410    8   MOLECULE: FLAGELLIN;                                                  227:  6jy0-W  6.5  2.4   83   410    8   MOLECULE: FLAGELLIN;                                                  228:  6dkm-F  6.5  1.5   59    67    7   MOLECULE: DHD131_A;                                                   229:  5j0k-A  6.5  1.9   65    74    5   MOLECULE: DESIGNED PROTEIN 2L4HC2_23;                                 230:  7use-E  6.5  2.4   71   155    0   MOLECULE: CYTOPLASMIC FMR1-INTERACTING PROTEIN 1;                     231:  8cye-Q  6.5  2.7   78   269    6   MOLECULE: FLAGELLIN;                                                  232:  6rwb-C  6.5  3.0   80  1873    6   MOLECULE: TOXIN,TOXIN COMPLEX SUBUNIT TCAB,PUTATIVE TOXIN S           233:  6eu0-O  6.5  5.3   80   550    8   MOLECULE: DNA-DIRECTED RNA POLYMERASE III SUBUNIT RPC1;               234:  4xvn-F  6.5  2.8   71   137    8   MOLECULE: SMALL TERMINASE;                                            235:  8dzz-A  6.5  1.9   76  2363    8   MOLECULE: DYNEIN HEAVY CHAIN, CYTOPLASMIC;                            236:  5izs-E  6.5  4.1   75    81    5   MOLECULE: DESIGNED PROTEIN 5L6HC3_1;                                  237:  6jy0-L  6.5  2.4   83   410    8   MOLECULE: FLAGELLIN;                                                  238:  7uh4-C  6.5  4.4   86   110   13   MOLECULE: LXG-ASSOCIATED ALPHA-HELICAL PROTEIN D2;                    239:  5zhy-E  6.5  2.4   77    88    5   MOLECULE: SPIKE GLYCOPROTEIN, SPIKE GLYCOPROTEIN;                     240:  5izs-C  6.5  3.6   72    82    4   MOLECULE: DESIGNED PROTEIN 5L6HC3_1;                                  241:  1wnc-F  6.5  2.0   71    75   21   MOLECULE: E2 GLYCOPROTEIN;                                            242:  6jy0-O  6.5  2.4   83   410    8   MOLECULE: FLAGELLIN;                                                  243:  6jy0-J  6.5  2.4   83   410    8   MOLECULE: FLAGELLIN;                                                  244:  6d03-E  6.4  3.2   81   466   12   MOLECULE: TRANSFERRIN RECEPTOR PROTEIN 1;                             245:  6dlm-A  6.4  1.8   64    74    9   MOLECULE: DHD127_A;                                                   246:  6rwb-A  6.4  3.0   80  1873    6   MOLECULE: TOXIN,TOXIN COMPLEX SUBUNIT TCAB,PUTATIVE TOXIN S           247:  7zoq-A  6.4  3.4   71   812    7   MOLECULE: TPR-CHAT;                                                   248:  5nug-A  6.4  1.5   70  2920    3   MOLECULE: CYTOPLASMIC DYNEIN 1 HEAVY CHAIN 1;                         249:  3etx-B  6.4  2.2   71   107    4   MOLECULE: ADHESIN A;                                                  250:  3r84-A  6.4  1.4   62    81    6   MOLECULE: MEDIATOR OF RNA POLYMERASE II TRANSCRIPTION SUBUN           251:  6el1-E  6.4  2.5   80   351    8   MOLECULE: YAXA;                                                       252:  3r84-S  6.4  1.4   62    81    6   MOLECULE: MEDIATOR OF RNA POLYMERASE II TRANSCRIPTION SUBUN           253:  3r84-K  6.4  1.3   62    80    6   MOLECULE: MEDIATOR OF RNA POLYMERASE II TRANSCRIPTION SUBUN           254:  3r84-Q  6.4  1.4   62    80    6   MOLECULE: MEDIATOR OF RNA POLYMERASE II TRANSCRIPTION SUBUN           255:  7b2l-I  6.4  2.0   64   247   11   MOLECULE: ENTH DOMAIN OF EPSIN ENT1;                                  256:  3tul-B  6.4  2.5   83   133    6   MOLECULE: CELL INVASION PROTEIN SIPB;                                 257:  7b2l-D  6.4  2.0   64   247   11   MOLECULE: ENTH DOMAIN OF EPSIN ENT1;                                  258:  7b2l-S  6.4  2.0   64   247   11   MOLECULE: ENTH DOMAIN OF EPSIN ENT1;                                  259:  7b2l-N  6.4  2.0   64   247   11   MOLECULE: ENTH DOMAIN OF EPSIN ENT1;                                  260:  8cwy-X  6.4  1.9   65    74    3   MOLECULE: T32-15-1;                                                   261:  1fze-D  6.4  2.4   69    80   12   MOLECULE: FIBRINOGEN;                                                 262:  1fzc-D  6.4  2.5   67    74   10   MOLECULE: FIBRIN;                                                     263:  1ebo-D  6.4  2.5   78   112   14   MOLECULE: EBOLA VIRUS ENVELOPE PROTEIN CHIMERA CONSISTING             264:  8cwy-J  6.4  1.9   65    74    3   MOLECULE: T32-15-1;                                                   265:  8pmq-9  6.4  4.7   82   412   18   MOLECULE: E3 UBIQUITIN-PROTEIN LIGASE RMD5;                           266:  8idc-E  6.4  2.6   85   300   12   MOLECULE: CELL DIVISION ATP-BINDING PROTEIN FTSE;                     267:  4bpd-F  6.4  2.9   66    92    3   MOLECULE: DIACYLGLYCEROL KINASE;                                      268:  6ejq-B  6.4  2.6   71   141    7   MOLECULE: TERMINASE SMALL SUBUNIT;                                    269:  4dk0-A  6.4  2.4   79   324   11   MOLECULE: PUTATIVE MACA;                                              270:  5lki-A  6.4  2.5   81  1427    5   MOLECULE: TCDA1;                                                      271:  3hhm-B  6.4  1.9   80   247    8   MOLECULE: PHOSPHATIDYLINOSITOL-4,5-BISPHOSPHATE 3-KINASE              272:  3mtt-A  6.4  3.3   84   163    5   MOLECULE: PHOSPHATIDYLINOSITOL 3-KINASE REGULATORY SUBUNIT            273:  3lay-D  6.4  4.1   69    78   10   MOLECULE: ZINC RESISTANCE-ASSOCIATED PROTEIN;                         274:  5j9q-H  6.4  4.0   79   120    1   MOLECULE: HISTONE ACETYLTRANSFERASE ESA1;                             275:  8cwy-R  6.4  1.9   65    74    3   MOLECULE: T32-15-1;                                                   276:  6nr8-5  6.4  3.0   77   127   13   MOLECULE: PREFOLDIN SUBUNIT 1;                                        277:  6ejq-A  6.4  2.8   71   138    6   MOLECULE: TERMINASE SMALL SUBUNIT;                                    278:  4xvn-D  6.4  3.0   72   139    7   MOLECULE: SMALL TERMINASE;                                            279:  1wnc-C  6.4  2.4   76    78   20   MOLECULE: E2 GLYCOPROTEIN;                                            280:  6vy1-c  6.4  2.8   78   121   14   MOLECULE: PREFOLDIN SUBUNIT ALPHA 2;                                  281:  6rwb-B  6.4  3.0   80  1873    6   MOLECULE: TOXIN,TOXIN COMPLEX SUBUNIT TCAB,PUTATIVE TOXIN S           282:  4j7k-D  6.4  2.7   77    84    9   MOLECULE: SECRETED PROTEIN ESXB;                                      283:  4j41-A  6.4  2.8   80    89    9   MOLECULE: SECRETED PROTEIN ESXB;                                      284:  3hr0-B  6.4  2.3   74   249    7   MOLECULE: COG4;                                                       285:  4j41-D  6.4  2.7   77    84    9   MOLECULE: SECRETED PROTEIN ESXB;                                      286:  8cye-I  6.4  2.9   86   269    3   MOLECULE: FLAGELLIN;                                                  287:  4j11-D  6.4  3.0   79    86    9   MOLECULE: SECRETED PROTEIN ESXB;                                      288:  6rwb-D  6.4  3.0   80  1873    6   MOLECULE: TOXIN,TOXIN COMPLEX SUBUNIT TCAB,PUTATIVE TOXIN S           289:  5zhy-C  6.4  2.5   78    89    5   MOLECULE: SPIKE GLYCOPROTEIN, SPIKE GLYCOPROTEIN;                     290:  5da9-A  6.3  2.6   75   433    5   MOLECULE: PUTATIVE UNCHARACTERIZED PROTEIN,PUTATIVE UNCHARA           291:  8h36-G  6.3  2.5   83   379    5   MOLECULE: E3 UBIQUITIN-PROTEIN LIGASE RBX1;                           292:  4j41-B  6.3  2.5   77    86    9   MOLECULE: SECRETED PROTEIN ESXB;                                      293:  3fd9-A  6.3  3.1   70   240   11   MOLECULE: UNCHARACTERIZED PROTEIN;                                    294:  6el1-D  6.3  2.5   80   351    8   MOLECULE: YAXA;                                                       295:  6el1-C  6.3  2.5   80   351    8   MOLECULE: YAXA;                                                       296:  6el1-B  6.3  2.5   80   351    8   MOLECULE: YAXA;                                                       297:  6el1-A  6.3  2.5   80   351    8   MOLECULE: YAXA;                                                       298:  8cwy-L  6.3  1.9   65    74    3   MOLECULE: T32-15-1;                                                   299:  6ree-F  6.3  3.5   67    74    1   MOLECULE: ASA-10: POLYTOMELLA F-ATP SYNTHASE ASSOCIATED SUB           300:  7vrc-A  6.3  2.9   71   107   14   MOLECULE: TRANSCRIPTION REGULATORY PROTEIN SNF11;                     301:  6njn-G  6.3  1.4   61   117    0   MOLECULE: GLUTAMATE RECEPTOR 1;                                       302:  2hod-J  6.3  2.6   67    79   10   MOLECULE: FIBRINOGEN ALPHA CHAIN;                                     303:  6hk5-B  6.3  2.1   60    66    5   MOLECULE: COOJ;                                                       304:  6oqu-S  6.3  3.7   68    77    9   MOLECULE: ATP SYNTHASE SUBUNIT DELTA;                                 305:  6rer-H  6.3  3.6   67    74    1   MOLECULE: MITOCHONDRIAL ATP SYNTHASE SUBUNIT C;                       306:  6ree-H  6.3  3.6   67    74    1   MOLECULE: ASA-10: POLYTOMELLA F-ATP SYNTHASE ASSOCIATED SUB           307:  6wvt-L  6.3  1.7   61   168    8   MOLECULE: ACTIN, ALPHA SKELETAL MUSCLE;                               308:  7utj-H  6.3  1.7   60   156    8   MOLECULE: ACTIN, ALPHA SKELETAL MUSCLE;                               309:  6wvt-N  6.3  1.7   61   168    8   MOLECULE: ACTIN, ALPHA SKELETAL MUSCLE;                               310:  1fxk-B  6.3  3.4   75   109    7   MOLECULE: PREFOLDIN;                                                  311:  7d76-R  6.3  2.3   60   265   10   MOLECULE: GUANINE NUCLEOTIDE-BINDING PROTEIN G(O) SUBUNIT A           312:  3aei-A  6.3  3.7   75    94   11   MOLECULE: PREFOLDIN BETA SUBUNIT 2;                                   313:  7p3r-A  6.3  1.9   81   328    7   MOLECULE: MAKA TETRAMER;                                              314:  5fd7-A  6.3  2.1   71   123    8   MOLECULE: VACUOLAR-SORTING PROTEIN SNF7;                              315:  3hiz-B  6.3  2.0   82   234    6   MOLECULE: PHOSPHATIDYLINOSITOL-4,5-BISPHOSPHATE 3-KINASE              316:  6ref-F  6.3  3.5   67    74    1   MOLECULE: ASA-10: POLYTOMELLA F-ATP SYNTHASE ASSOCIATED SUB           317:  7o3y-E  6.3  2.7   80   215    8   MOLECULE: PROTEIN SLL0617;                                            318:  7tz7-B  6.3  3.3   81   266    7   MOLECULE: PHOSPHATIDYLINOSITOL 4,5-BISPHOSPHATE 3-KINASE CA           319:  4j7k-A  6.3  2.3   76    90    9   MOLECULE: SECRETED PROTEIN ESXB;                                      320:  8cvi-M  6.3  2.1   85   269    6   MOLECULE: FLAGELLIN;                                                  321:  5sw8-B  6.3  2.2   85   201    5   MOLECULE: PHOSPHATIDYLINOSITOL 4,5-BISPHOSPHATE 3-KINASE CA           322:  5ip0-N  6.3  4.0   76    88   12   MOLECULE: PHA GRANULE-ASSOCIATED PROTEIN;                             323:  6rwb-E  6.3  3.0   80  1873    6   MOLECULE: TOXIN,TOXIN COMPLEX SUBUNIT TCAB,PUTATIVE TOXIN S           324:  6ois-E  6.3  2.4   80   329    5   MOLECULE: PROTEIN RDM1;                                               325:  4j10-A  6.3  2.2   76    88    9   MOLECULE: SECRETED PROTEIN ESXB;                                      326:  5zuv-C  6.3  2.4   77   119   17   MOLECULE: SPIKE GLYCOPROTEIN,INHIBITOR EK1;                           327:  4j11-B  6.3  2.7   80    93   10   MOLECULE: SECRETED PROTEIN ESXB;                                      328:  6ejq-C  6.3  2.7   70   137    6   MOLECULE: TERMINASE SMALL SUBUNIT;                                    329:  8v8j-B  6.3  2.6   75   239    3   MOLECULE: PHOSPHATIDYLINOSITOL 4,5-BISPHOSPHATE 3-KINASE CA           330:  7nyy-A  6.3  2.9   83  1467   10   MOLECULE: CHROMOSOME PARTITION PROTEIN MUKB;                          331:  7nz0-A  6.3  4.1   81  1467   10   MOLECULE: CHROMOSOME PARTITION PROTEIN MUKB;                          332:  3gwk-E  6.3  3.6   79    97    6   MOLECULE: PUTATIVE UNCHARACTERIZED PROTEIN SAG1039;                   333:  7usd-E  6.3  2.8   74   155    0   MOLECULE: CYTOPLASMIC FMR1-INTERACTING PROTEIN 1;                     334:  6ejq-F  6.3  2.8   70   139    9   MOLECULE: TERMINASE SMALL SUBUNIT;                                    335:  4mu6-A  6.2  2.2   83   274   10   MOLECULE: KINECTIN 1 (KINESIN RECEPTOR);                              336:  3tul-A  6.2  2.8   80   136    5   MOLECULE: CELL INVASION PROTEIN SIPB;                                 337:  6yvu-A  6.2  2.8   80  1127    3   MOLECULE: STRUCTURAL MAINTENANCE OF CHROMOSOMES PROTEIN 2,S           338:  5j2l-A  6.2  2.6   67    76    4   MOLECULE: PROTEIN DESIGN 2L4HC2_11;                                   339:  5nnv-D  6.2  2.6   82   252    9   MOLECULE: CHROMOSOME PARTITION PROTEIN SMC,CHROMOSOME PARTI           340:  6zsi-D  6.2  2.3   71   133    7   MOLECULE: RAS-RELATED PROTEIN RAB-8A;                                 341:  8pmq-2  6.2  3.0   81   355    7   MOLECULE: E3 UBIQUITIN-PROTEIN LIGASE RMD5;                           342:  5jhf-F  6.2  3.7   84   405    6   MOLECULE: KLTH0D11660P;                                               343:  6jy0-A  6.2  2.4   82   410    2   MOLECULE: FLAGELLIN;                                                  344:  4dci-D  6.2  3.5   79   145   13   MOLECULE: UNCHARACTERIZED PROTEIN;                                    345:  5ebz-D  6.2  2.4   84   655    7   MOLECULE: INHIBITOR OF NUCLEAR FACTOR KAPPA-B KINASE SUBUNI           346:  5jdo-B  6.2  2.1   67   245   10   MOLECULE: HAPTOGLOBIN-HAEMOGLOBIN RECEPTOR;                           347:  8eav-E  6.2  4.1   71   159    0   MOLECULE: YAR027W OR YAR028W;                                         348:  7sn7-I  6.2  2.8   85   546    4   MOLECULE: FLAGELLIN;                                                  349:  5ip0-B  6.2  4.5   78    88   12   MOLECULE: PHA GRANULE-ASSOCIATED PROTEIN;                             350:  6vme-H  6.2  1.9   60    76    8   MOLECULE: TUMOR SUSCEPTIBILITY GENE 101 PROTEIN;                      351:  1fzb-D  6.2  2.5   68    81   10   MOLECULE: FIBRINOGEN;                                                 352:  1fzb-A  6.2  2.5   68    81   10   MOLECULE: FIBRINOGEN;                                                 353:  2hpc-J  6.2  2.5   67    79   10   MOLECULE: FIBRINOGEN ALPHA CHAIN;                                     354:  7upo-C  6.2  2.9   67    76    4   MOLECULE: DHT03 PROTEIN A;                                            355:  7upq-I  6.2  2.9   67    76    4   MOLECULE: DHT03 PROTEIN A;                                            356:  6nrb-2  6.2  3.3   84   103    7   MOLECULE: T-COMPLEX PROTEIN 1 SUBUNIT ALPHA;                          357:  2gd5-D  6.2  2.2   78   162    9   MOLECULE: CHARGED MULTIVESICULAR BODY PROTEIN 3;                      358:  2vkz-B  6.2  4.9   66  1614    9   MOLECULE: FATTY ACID SYNTHASE SUBUNIT ALPHA;                          359:  4afl-A  6.2  4.1   80   102   11   MOLECULE: INHIBITOR OF GROWTH PROTEIN 4;                              360:  6wvt-Q  6.2  1.7   59   168    8   MOLECULE: ACTIN, ALPHA SKELETAL MUSCLE;                               361:  4afl-E  6.2  3.7   77   101   12   MOLECULE: INHIBITOR OF GROWTH PROTEIN 4;                              362:  4afl-F  6.2  3.1   76    91   11   MOLECULE: INHIBITOR OF GROWTH PROTEIN 4;                              363:  4afl-C  6.2  3.0   77   102    9   MOLECULE: INHIBITOR OF GROWTH PROTEIN 4;                              364:  6rer-I  6.2  3.6   67    74    6   MOLECULE: MITOCHONDRIAL ATP SYNTHASE SUBUNIT C;                       365:  6ref-D  6.2  3.5   67    74    6   MOLECULE: ASA-10: POLYTOMELLA F-ATP SYNTHASE ASSOCIATED SUB           366:  3aei-B  6.2  3.9   75    94   11   MOLECULE: PREFOLDIN BETA SUBUNIT 2;                                   367:  4zwq-G  6.2  5.7   85   132    8   MOLECULE: RECOMBINATION PROTEIN UVSY;                                 368:  5sxb-B  6.2  2.3   82   212    6   MOLECULE: PHOSPHATIDYLINOSITOL 4,5-BISPHOSPHATE 3-KINASE CA           369:  3udc-A  6.2  4.1   68   267    6   MOLECULE: SMALL-CONDUCTANCE MECHANOSENSITIVE CHANNEL, C-TER           370:  5izs-A  6.2  3.4   68    77    4   MOLECULE: DESIGNED PROTEIN 5L6HC3_1;                                  371:  3udc-F  6.2  4.1   68   267    6   MOLECULE: SMALL-CONDUCTANCE MECHANOSENSITIVE CHANNEL, C-TER           372:  3udc-E  6.2  4.1   68   267    6   MOLECULE: SMALL-CONDUCTANCE MECHANOSENSITIVE CHANNEL, C-TER           373:  5izs-B  6.2  3.4   68    77    4   MOLECULE: DESIGNED PROTEIN 5L6HC3_1;                                  374:  8cye-N  6.2  2.7   78   269    6   MOLECULE: FLAGELLIN;                                                  375:  8pqz-A  6.2  2.7   73  2892   12   MOLECULE: CYTOPLASMIC DYNEIN 1 HEAVY CHAIN 1;                         376:  5sxi-B  6.2  2.2   85   209    5   MOLECULE: PHOSPHATIDYLINOSITOL 4,5-BISPHOSPHATE 3-KINASE CA           377:  4j7j-B  6.2  2.9   78    87    9   MOLECULE: SECRETED PROTEIN ESXB;                                      378:  5ip0-F  6.2  4.2   75    88   12   MOLECULE: PHA GRANULE-ASSOCIATED PROTEIN;                             379:  6oit-C  6.2  2.4   81   334    5   MOLECULE: PROTEIN RDM1;                                               380:  8tgd-D  6.2  2.4   86   245    5   MOLECULE: PHOSPHATIDYLINOSITOL 4,5-BISPHOSPHATE 3-KINASE CA           381:  3a5x-A  6.2  2.5   85   494    1   MOLECULE: FLAGELLIN;                                                  382:  8tgd-B  6.2  2.3   85   245    5   MOLECULE: PHOSPHATIDYLINOSITOL 4,5-BISPHOSPHATE 3-KINASE CA           383:  7upq-F  6.1  3.0   68    77    7   MOLECULE: DHT03 PROTEIN A;                                            384:  7cbc-A  6.1  4.4   82   319    4   MOLECULE: DE NOVO DESIGNED SWITCH PROTEIN CAGING A HEMAGGLU           385:  8qfc-C  6.1  2.8   83   380   13   MOLECULE: 60S RIBOSOMAL PROTEIN L10A;                                 386:  4dci-G  6.1  3.5   79   143   13   MOLECULE: UNCHARACTERIZED PROTEIN;                                    387:  4ck0-A  6.1  3.0   66   114   11   MOLECULE: DIACYLGLYCEROL KINASE;                                      388:  5ip0-H  6.1  4.5   84   108    6   MOLECULE: PHA GRANULE-ASSOCIATED PROTEIN;                             389:  6ynw-M  6.1  3.4   64    75    2   MOLECULE: SUBUNIT C;                                                  390:  6ynw-J  6.1  3.4   64    75    2   MOLECULE: SUBUNIT C;                                                  391:  6ynw-O  6.1  3.3   64    75    2   MOLECULE: SUBUNIT C;                                                  392:  6ynw-L  6.1  3.4   64    75    2   MOLECULE: SUBUNIT C;                                                  393:  6ynw-I  6.1  3.4   64    75    2   MOLECULE: SUBUNIT C;                                                  394:  6ynw-Q  6.1  3.3   64    75    2   MOLECULE: SUBUNIT C;                                                  395:  6ynw-K  6.1  3.4   64    75    2   MOLECULE: SUBUNIT C;                                                  396:  8bri-D  6.1  3.5   82   243    6   MOLECULE: CHEMOTAXIS PROTEIN POMA;                                    397:  2oyh-A  6.1  2.5   62    64   11   MOLECULE: FIBRINOGEN ALPHA CHAIN;                                     398:  6v1i-I  6.1  2.7   71   137    6   MOLECULE: SMALL TERMINASE PROTEIN;                                    399:  6c14-B  6.1  3.1   62   164    8   MOLECULE: PROTOCADHERIN-15;                                           400:  5j73-A  6.1  1.7   60    75   15   MOLECULE: PROTEIN DESIGN 2L4HC2_9;                                    401:  7uw5-A  6.1  2.8   66   723    5   MOLECULE: MECHANOSENSITIVE CHANNEL MSCK;                              402:  4uos-A  6.1  4.8   77   188    6   MOLECULE: DESIGNED HELICAL BUNDLE;                                    403:  1n86-D  6.1  2.5   67    74   10   MOLECULE: FIBRIN ALPHA/ALPHA-E CHAIN;                                 404:  4a55-B  6.1  2.2   85   141    5   MOLECULE: PHOSPHATIDYLINOSITOL-4,5-BISPHOSPHATE 3-KINASE CA           405:  8gl3-A  6.1  2.9   80   189   10   MOLECULE: PRLB-519;                                                   406:  7jw1-e  6.1  2.3   83   235   10   MOLECULE: CAPSID PROTEINS;                                            407:  7mpe-A  6.1  4.1   78  1428   13   MOLECULE: METAL RESISTANCE PROTEIN YCF1;                              408:  5bw9-g  6.1  2.3   82   141   10   MOLECULE: V-TYPE PROTON ATPASE CATALYTIC SUBUNIT A;                   409:  1skv-A  6.1  1.9   61    64    2   MOLECULE: HYPOTHETICAL 7.5 KDA PROTEIN;                               410:  1fze-A  6.1  2.7   74    81    4   MOLECULE: FIBRINOGEN;                                                 411:  7o3x-E  6.1  2.8   80   215    8   MOLECULE: PROTEIN SLL0617;                                            412:  4uxw-A  6.1  3.0   66   114   11   MOLECULE: DIACYLGLYCEROL KINASE;                                      413:  6v1i-H  6.1  2.7   71   137    6   MOLECULE: SMALL TERMINASE PROTEIN;                                    414:  4w4l-A  6.1  2.4   69    84    7   MOLECULE: PE FAMILY PROTEIN PE25;                                     415:  3teq-B  6.1  4.1   83   101   10   MOLECULE: STROMAL INTERACTION MOLECULE 1;                             416:  1kmi-Z  6.1  2.3   83   177   12   MOLECULE: CHEMOTAXIS PROTEIN CHEY;                                    417:  3t9n-D  6.1  4.6   66   265    8   MOLECULE: SMALL-CONDUCTANCE MECHANOSENSITIVE CHANNEL;                 418:  4afl-D  6.1  3.0   75    95   11   MOLECULE: INHIBITOR OF GROWTH PROTEIN 4;                              419:  6wvt-X  6.1  1.7   59   168    8   MOLECULE: ACTIN, ALPHA SKELETAL MUSCLE;                               420:  3t9n-C  6.1  4.6   66   264    8   MOLECULE: SMALL-CONDUCTANCE MECHANOSENSITIVE CHANNEL;                 421:  3t9n-G  6.1  4.6   66   264    8   MOLECULE: SMALL-CONDUCTANCE MECHANOSENSITIVE CHANNEL;                 422:  6wvt-O  6.1  1.7   59   168    8   MOLECULE: ACTIN, ALPHA SKELETAL MUSCLE;                               423:  3t9n-E  6.1  4.6   66   264    8   MOLECULE: SMALL-CONDUCTANCE MECHANOSENSITIVE CHANNEL;                 424:  3t9n-B  6.1  4.6   66   264    8   MOLECULE: SMALL-CONDUCTANCE MECHANOSENSITIVE CHANNEL;                 425:  3t9n-F  6.1  4.6   66   264    8   MOLECULE: SMALL-CONDUCTANCE MECHANOSENSITIVE CHANNEL;                 426:  4dci-A  6.1  4.0   78   147    9   MOLECULE: UNCHARACTERIZED PROTEIN;                                    427:  7upo-B  6.1  3.4   70    78   10   MOLECULE: DHT03 PROTEIN A;                                            428:  5sx8-B  6.1  2.3   82   240    6   MOLECULE: PHOSPHATIDYLINOSITOL 4,5-BISPHOSPHATE 3-KINASE CA           429:  5u1d-A  6.1  4.9   76   561    9   MOLECULE: ANTIGEN PEPTIDE TRANSPORTER 1;                              430:  3udc-C  6.1  4.1   68   267    6   MOLECULE: SMALL-CONDUCTANCE MECHANOSENSITIVE CHANNEL, C-TER           431:  6ejq-H  6.1  2.1   68   137    7   MOLECULE: TERMINASE SMALL SUBUNIT;                                    432:  3o9o-A  6.1  2.4   76    92    5   MOLECULE: UNCHARACTERIZED PROTEIN GBS1074;                            433:  8cye-K  6.1  2.7   83   269    5   MOLECULE: FLAGELLIN;                                                  434:  8cye-V  6.1  2.9   82   269   12   MOLECULE: FLAGELLIN;                                                  435:  4j11-C  6.1  2.2   73    91    8   MOLECULE: SECRETED PROTEIN ESXB;                                      436:  8v8j-D  6.1  3.1   86   239    5   MOLECULE: PHOSPHATIDYLINOSITOL 4,5-BISPHOSPHATE 3-KINASE CA           437:  8cvi-Z  6.1  2.8   82   268   11   MOLECULE: FLAGELLIN;                                                  438:  4j7k-B  6.1  2.8   82    93    9   MOLECULE: SECRETED PROTEIN ESXB;                                      439:  5j0l-E  6.1  4.6   69   111   16   MOLECULE: DESIGNED PROTEIN 3L6HC2_2;                                  440:  3gvm-B  6.1  2.5   77    94    6   MOLECULE: PUTATIVE UNCHARACTERIZED PROTEIN SAG1039;                   441:  3gvm-A  6.1  3.5   80    96    6   MOLECULE: PUTATIVE UNCHARACTERIZED PROTEIN SAG1039;                   442:  8tdu-D  6.1  2.3   85   237    5   MOLECULE: PHOSPHATIDYLINOSITOL 4,5-BISPHOSPHATE 3-KINASE CA           443:  1wnc-D  6.1  2.1   74    77   22   MOLECULE: E2 GLYCOPROTEIN;                                            444:  8tu6-B  6.1  2.5   85   252    5   MOLECULE: PHOSPHATIDYLINOSITOL 4,5-BISPHOSPHATE 3-KINASE CA           445:  4j10-B  6.1  2.4   74    82    9   MOLECULE: SECRETED PROTEIN ESXB;                                      446:  8tdu-B  6.1  2.3   85   227    5   MOLECULE: PHOSPHATIDYLINOSITOL 4,5-BISPHOSPHATE 3-KINASE CA           447:  3gvm-D  6.1  2.5   77    94    6   MOLECULE: PUTATIVE UNCHARACTERIZED PROTEIN SAG1039;                   448:  5m6u-B  6.1  3.2   83   164    5   MOLECULE: PHOSPHATIDYLINOSITOL 4,5-BISPHOSPHATE 3-KINASE CA           449:  3zbh-D  6.1  2.7   77    93   10   MOLECULE: ESXA;                                                       450:  4j7j-A  6.1  2.4   75    87    9   MOLECULE: SECRETED PROTEIN ESXB;                                      451:  1mg1-A  6.0  2.6   81   450   14   MOLECULE: PROTEIN (HTLV-1 GP21 ECTODOMAIN/MALTOSE-BINDING P           452:  2zdi-C  6.0  3.0   83   148   16   MOLECULE: PREFOLDIN SUBUNIT BETA;                                     453:  5j10-A  6.0  2.6   62    70    6   MOLECULE: PEPTIDE DESIGN 2L4HC2_24;                                   454:  5los-A  6.0  2.2   78    89    8   MOLECULE: PIIN_05872;                                                 455:  5gox-A  6.0  3.3   76   181    7   MOLECULE: DNA REPAIR PROTEIN RAD50;                                   456:  5z7b-B  6.0  4.2   76   197    7   MOLECULE: PADR FAMILY TRANSCRIPTIONAL REGULATOR;                      457:  7aqc-R  6.0  3.1   77   558    8   MOLECULE: 23S RIBOSOMAL RNA;                                          458:  4v1f-A  6.0  4.4   78    86    4   MOLECULE: F0F1 ATP SYNTHASE SUBUNIT C;                                459:  1fxk-C  6.0  3.0   84   133    6   MOLECULE: PREFOLDIN;                                                  460:  2g38-A  6.0  2.7   68    77    7   MOLECULE: PE FAMILY PROTEIN;                                          461:  3etw-A  6.0  3.0   78   109    9   MOLECULE: ADHESIN A;                                                  462:  2x0l-A  6.0  3.2   78   670    5   MOLECULE: LYSINE-SPECIFIC HISTONE DEMETHYLASE 1;                      463:  8dk2-C  6.0  2.1   81   723   10   MOLECULE: JETA;                                                       464:  6ynw-P  6.0  3.4   64    75    2   MOLECULE: SUBUNIT C;                                                  465:  6ynw-N  6.0  3.4   64    75    2   MOLECULE: SUBUNIT C;                                                  466:  7p5c-A  6.0  3.8   81   392    1   MOLECULE: PROTEIN TWEETY HOMOLOG 3;                                   467:  6cno-C  6.0  1.9   68   360    3   MOLECULE: INTERMEDIATE CONDUCTANCE CALCIUM-ACTIVATED POTASS           468:  4bt9-B  6.0  4.0   81   238    5   MOLECULE: PROLYL 4-HYDROXYLASE SUBUNIT ALPHA-1;                       469:  5d57-F  6.0  2.8   67    88   10   MOLECULE: DIACYLGLYCEROL KINASE;                                      470:  4kum-A  6.0  3.0   78   665    4   MOLECULE: LYSINE-SPECIFIC HISTONE DEMETHYLASE 1A;                     471:  1yd8-G  6.0  1.5   55    93   16   MOLECULE: UBIQUIN;                                                    472:  1fza-A  6.0  2.5   69    85   12   MOLECULE: FIBRINOGEN;                                                 473:  8j0h-H  6.0  2.1   59   129   14   MOLECULE: UNCHARACTERIZED PROTEIN C4H3.06;                            474:  1fza-D  6.0  2.5   69    85   12   MOLECULE: FIBRINOGEN;                                                 475:  6c14-D  6.0  3.4   63   164    8   MOLECULE: PROTOCADHERIN-15;                                           476:  6dma-B  6.0  2.1   63    73   11   MOLECULE: DHD15_CLOSED_A;                                             477:  8fbn-A  6.0  2.2   64   226    3   MOLECULE: KWOCA_73;                                                   478:  7p3n-Q  6.0  4.1   73    80    7   MOLECULE: ATP SYNTHASE SUBUNIT ALPHA;                                 479:  8akr-C  6.0  2.5   81   196    6   MOLECULE: CHLOROPLAST MEMBRANE-ASSOCIATED 30 KD PROTEIN;              480:  8akr-K  6.0  2.3   82   196    7   MOLECULE: CHLOROPLAST MEMBRANE-ASSOCIATED 30 KD PROTEIN;              481:  6wnq-O  6.0  3.5   67    77    7   MOLECULE: ATP SYNTHASE SUBUNIT DELTA;                                 482:  8akr-e  6.0  2.7   81   196    7   MOLECULE: CHLOROPLAST MEMBRANE-ASSOCIATED 30 KD PROTEIN;              483:  6o84-A  6.0  4.4   71   415    7   MOLECULE: LOC100127796 PROTEIN,LOC100127796 PROTEIN,OTOP3,            484:  3q4h-D  6.0  3.2   73    83   10   MOLECULE: PE FAMILY PROTEIN;                                          485:  3lss-B  6.0  3.5   82   468    4   MOLECULE: SERYL-TRNA SYNTHETASE;                                      486:  7p3w-Q  6.0  4.0   72    79    7   MOLECULE: ATP SYNTHASE SUBUNIT ALPHA;                                 487:  7jna-A  6.0  5.0   74   278    7   MOLECULE: PROTON-ACTIVATED CHLORIDE CHANNEL;                          488:  7jna-C  6.0  5.0   74   278    7   MOLECULE: PROTON-ACTIVATED CHLORIDE CHANNEL;                          489:  5j0i-A  6.0  2.9   67    73   13   MOLECULE: DESIGNED PROTEIN 2L6HC3_12;                                 490:  5t4o-U  6.0  3.5   69    75    9   MOLECULE: ATP SYNTHASE SUBUNIT ALPHA;                                 491:  5t4o-O  6.0  3.2   68    75    9   MOLECULE: ATP SYNTHASE SUBUNIT ALPHA;                                 492:  4i0x-D  6.0  2.3   68    83   15   MOLECULE: ESAT-6-LIKE PROTEIN MAB_3112;                               493:  7v5d-A  6.0  4.5   79   570    5   MOLECULE: ABC-TYPE OLIGOPEPTIDE TRANSPORTER ABCB9;                    494:  3gvm-C  6.0  3.5   80    97    6   MOLECULE: PUTATIVE UNCHARACTERIZED PROTEIN SAG1039;                   495:  6v1i-B  6.0  2.4   68   137    7   MOLECULE: SMALL TERMINASE PROTEIN;                                    496:  6v1i-E  6.0  2.4   68   137    7   MOLECULE: SMALL TERMINASE PROTEIN;                                    497:  4xvn-B  6.0  2.5   69   123    4   MOLECULE: SMALL TERMINASE;                                            498:  3gwk-C  6.0  3.6   80    98    6   MOLECULE: PUTATIVE UNCHARACTERIZED PROTEIN SAG1039;                   499:  6ejq-D  6.0  3.2   70   134    7   MOLECULE: TERMINASE SMALL SUBUNIT;                                    500:  8v8h-B  6.0  2.4   85   239    5   MOLECULE: PHOSPHATIDYLINOSITOL 4,5-BISPHOSPHATE 3-KINASE CA           501:  5sx9-B  6.0  2.2   85   239    5   MOLECULE: PHOSPHATIDYLINOSITOL 4,5-BISPHOSPHATE 3-KINASE CA           502:  6v1i-C  6.0  2.4   68   137    7   MOLECULE: SMALL TERMINASE PROTEIN;                                    503:  6v1i-D  6.0  2.4   68   137    7   MOLECULE: SMALL TERMINASE PROTEIN;                                    504:  5zhy-F  6.0  2.4   74    89    7   MOLECULE: SPIKE GLYCOPROTEIN, SPIKE GLYCOPROTEIN;                     505:  8cye-G  6.0  2.7   83   269    5   MOLECULE: FLAGELLIN;                                                  506:  6nct-B  6.0  2.1   84   225    5   MOLECULE: PHOSPHATIDYLINOSITOL 4,5-BISPHOSPHATE 3-KINASE CA           507:  1wnc-E  6.0  1.8   69    74   22   MOLECULE: E2 GLYCOPROTEIN;                                            508:  5sxf-B  6.0  2.3   85   183    5   MOLECULE: PHOSPHATIDYLINOSITOL 4,5-BISPHOSPHATE 3-KINASE CA           509:  3o9o-B  6.0  3.4   79    96    6   MOLECULE: UNCHARACTERIZED PROTEIN GBS1074;                            510:  4j11-A  6.0  2.4   74    87    9   MOLECULE: SECRETED PROTEIN ESXB;                                      511:  6v1i-G  6.0  2.4   68   137    7   MOLECULE: SMALL TERMINASE PROTEIN;                                    512:  6v1i-A  6.0  2.4   68   137    7   MOLECULE: SMALL TERMINASE PROTEIN;                                    513:  6v1i-F  6.0  2.4   68   137    7   MOLECULE: SMALL TERMINASE PROTEIN;                                    514:  3zbh-B  6.0  3.1   77    92    9   MOLECULE: ESXA;                                                       515:  8cvi-W  6.0  2.8   78   269    6   MOLECULE: FLAGELLIN;                                                  516:  5sxe-B  6.0  2.3   85   225    5   MOLECULE: PHOSPHATIDYLINOSITOL 4,5-BISPHOSPHATE 3-KINASE CA           517:  8ek4-B  5.9  5.6   83   135    7   MOLECULE: ICE-BINDING PROTEIN TIP-99A;                                518:  8c5v-I  5.9  2.8   82   516    6   MOLECULE: CHEMOTAXIS PROTEIN CHEA;                                    519:  6zbj-B  5.9  3.0   76   501    4   MOLECULE: PRECURSOR OF THE MAJOR MEROZOITE SURFACE ANTIGENS           520:  5h69-A  5.9  2.7   76   252    5   MOLECULE: CHROMOSOME PARTITION PROTEIN SMC;                           521:  8pm6-A  5.9  1.7   69   872   12   MOLECULE: BILE SALT EXPORT PUMP;                                      522:  7yqh-A  5.9  2.6   85  1069    6   MOLECULE: STRUCTURAL MAINTENANCE OF CHROMOSOMES PROTEIN 5;            523:  6vz1-A  5.9  5.6   76   411    7   MOLECULE: DIACYLGLYCEROL O-ACYLTRANSFERASE 1;                         524:  8ibh-A  5.9  2.2   64    75    9   MOLECULE: CENTROSOMAL PROTEIN OF 57 KDA;                              525:  5vo5-A  5.9  2.6   75   179    4   MOLECULE: COILED-COIL AND C2 DOMAIN-CONTAINING PROTEIN 1-LI           526:  5n6x-B  5.9  2.3   66   400    6   MOLECULE: WIPA;                                                       527:  3etz-A  5.9  3.0   77   106    9   MOLECULE: ADHESIN A;                                                  528:  3pwx-A  5.9  3.3   80   224   10   MOLECULE: PUTATIVE FLAGELLAR HOOK-ASSOCIATED PROTEIN;                 529:  3etx-C  5.9  3.0   79   106    9   MOLECULE: ADHESIN A;                                                  530:  4gzr-D  5.9  2.8   62    80    8   MOLECULE: ESAT-6-LIKE PROTEIN 6;                                      531:  6eyd-D  5.9  4.0   75  1241    8   MOLECULE: DNA-DIRECTED RNA POLYMERASE SUBUNIT ALPHA;                  532:  3tul-C  5.9  2.4   77   130    6   MOLECULE: CELL INVASION PROTEIN SIPB;                                 533:  4l1b-B  5.9  2.2   84   277    8   MOLECULE: PHOSPHATIDYLINOSITOL 4,5-BISPHOSPHATE 3-KINASE CA           534:  5a7d-O  5.9  2.2   59   288   10   MOLECULE: PINS;                                                       535:  3fpp-B  5.9  2.9   79   267    6   MOLECULE: MACROLIDE-SPECIFIC EFFLUX PROTEIN MACA;                     536:  3k9a-A  5.9  1.9   60    82   10   MOLECULE: HIV GLYCOPROTEIN GP41;                                      537:  6xj1-B  5.9  2.5   74   277   11   MOLECULE: CELL DIVISION CONTROL PROTEIN 15;                           538:  3zx6-A  5.9  2.2   83   303    4   MOLECULE: HAMP, METHYL-ACCEPTING CHEMOTAXIS PROTEIN I;                539:  2yq8-B  5.9  4.2   83   226    2   MOLECULE: PROLYL 4-HYDROXYLASE SUBUNIT ALPHA-1;                       540:  8ga6-A  5.9  2.7   64   476    6   MOLECULE: THR6;                                                       541:  6rrc-C  5.9  1.7   67   331   13   MOLECULE: COHESIN SUBUNIT SA-1;                                       542:  3p30-A  5.9  3.3   73    84    7   MOLECULE: HIV-1 GP41;                                                 543:  6vme-C  5.9  1.9   59    67    7   MOLECULE: TUMOR SUSCEPTIBILITY GENE 101 PROTEIN;                      544:  1fzf-A  5.9  2.3   64    67    9   MOLECULE: FIBRINOGEN;                                                 545:  1fzg-A  5.9  2.6   63    66   11   MOLECULE: FIBRINOGEN;                                                 546:  1rf0-A  5.9  2.5   62    64    5   MOLECULE: FIBRINOGEN ALPHA/ALPHA-E CHAIN;                             547:  2ebo-A  5.9  2.9   73    74   16   MOLECULE: EBOLA VIRUS ENVELOPE GLYCOPROTEIN;                          548:  2hpc-G  5.9  2.8   67    74   10   MOLECULE: FIBRINOGEN ALPHA CHAIN;                                     549:  7o3z-B  5.9  2.9   81   215    5   MOLECULE: PROTEIN SLL0617;                                            550:  8hpo-C  5.9  6.5   82   138   11   MOLECULE: TRANSCRIPTIONAL REGULATORY PROTEIN UME1;                    551:  7o3v-A  5.9  3.8   85   198    7   MOLECULE: TRWJ PROTEIN;                                               552:  6dkm-A  5.9  3.0   69    77    1   MOLECULE: DHD131_A;                                                   553:  6ynw-H  5.9  3.3   66    75    6   MOLECULE: SUBUNIT C;                                                  554:  7p3w-P  5.9  4.5   72    80    8   MOLECULE: ATP SYNTHASE SUBUNIT ALPHA;                                 555:  7p2y-L  5.9  4.6   73    80    7   MOLECULE: ATP SYNTHASE SUBUNIT ALPHA;                                 556:  2x2v-L  5.9  3.0   63    69    8   MOLECULE: ATP SYNTHASE SUBUNIT C;                                     557:  6v9z-A  5.9  4.7   78   715    9   MOLECULE: ABC-TYPE BACTERIOCIN TRANSPORTER;                           558:  3teq-C  5.9  4.4   82   101    9   MOLECULE: STROMAL INTERACTION MOLECULE 1;                             559:  6csv-A  5.9  4.7   76    90    5   MOLECULE: CENTROSOMAL PROTEIN OF 63 KDA,CENTROSOMAL PROTEIN           560:  5uhb-D  5.9  4.6   78  1265    9   MOLECULE: DNA-DIRECTED RNA POLYMERASE SUBUNIT ALPHA;                  561:  8t3s-R  5.9  2.6   64   258    5   MOLECULE: GUANINE NUCLEOTIDE-BINDING PROTEIN G(Q) SUBUNIT A           562:  7p2y-O  5.9  4.0   73    80    7   MOLECULE: ATP SYNTHASE SUBUNIT ALPHA;                                 563:  4v1g-B  5.9  4.6   77    86    4   MOLECULE: F0F1 ATP SYNTHASE SUBUNIT C;                                564:  6xns-D  5.9  3.0   76   330    4   MOLECULE: C3_CROWN-05;                                                565:  7o40-B  5.9  2.7   81   215    9   MOLECULE: PROTEIN SLL0617;                                            566:  5swt-B  5.9  2.3   82   249    6   MOLECULE: PHOSPHATIDYLINOSITOL 4,5-BISPHOSPHATE 3-KINASE CA           567:  7upq-C  5.9  2.9   67    76    7   MOLECULE: DHT03 PROTEIN A;                                            568:  4uvb-A  5.9  3.0   77   666    4   MOLECULE: LYSINE-SPECIFIC HISTONE DEMETHYLASE 1A;                     569:  8sbc-B  5.9  2.9   82   264    9   MOLECULE: PHOSPHATIDYLINOSITOL 4,5-BISPHOSPHATE 3-KINASE CA           570:  5zhy-B  5.9  2.9   77    89    8   MOLECULE: SPIKE GLYCOPROTEIN, SPIKE GLYCOPROTEIN;                     571:  6ejq-I  5.9  2.8   69    85    7   MOLECULE: TERMINASE SMALL SUBUNIT;                                    572:  6wtu-J  5.9  4.6   71   295    7   MOLECULE: RETICULOCYTE BINDING PROTEIN 2B;                            573:  8cye-R  5.9  2.8   83   269    5   MOLECULE: FLAGELLIN;                                                  574:  6oit-E  5.9  2.9   79   336    9   MOLECULE: PROTEIN RDM1;                                               575:  7pg6-B  5.9  2.3   85   266    5   MOLECULE: PHOSPHATIDYLINOSITOL 4,5-BISPHOSPHATE 3-KINASE CA           576:  5fi4-B  5.9  2.3   85   249    5   MOLECULE: PHOSPHATIDYLINOSITOL 4,5-BISPHOSPHATE 3-KINASE CA           577:  8v8h-D  5.9  2.4   84   239    5   MOLECULE: PHOSPHATIDYLINOSITOL 4,5-BISPHOSPHATE 3-KINASE CA           578:  8cye-H  5.9  2.7   84   269    8   MOLECULE: FLAGELLIN;                                                  579:  7sn7-L  5.9  3.2   81   546   11   MOLECULE: FLAGELLIN;                                                  580:  8tsd-B  5.9  2.5   84   270    4   MOLECULE: PHOSPHATIDYLINOSITOL 4,5-BISPHOSPHATE 3-KINASE CA           581:  3zbh-F  5.9  2.3   75    93    9   MOLECULE: ESXA;                                                       582:  8e00-A  5.9  2.7   82  2363    9   MOLECULE: DYNEIN HEAVY CHAIN, CYTOPLASMIC;                            583:  2vrz-A  5.8  2.9   74    98    7   MOLECULE: VIRULENCE FACTOR ESXA;                                      584:  5nik-D  5.8  2.8   79   340   10   MOLECULE: OUTER MEMBRANE PROTEIN TOLC;                                585:  5h9c-A  5.8  2.7   72    78   11   MOLECULE: ENVELOPE GLYCOPROTEIN GP95;                                 586:  5ofb-B  5.8  2.9   72   541    3   MOLECULE: MORC FAMILY CW-TYPE ZINC FINGER PROTEIN 2;                  587:  4dci-E  5.8  3.4   79   147   13   MOLECULE: UNCHARACTERIZED PROTEIN;                                    588:  2x2v-K  5.8  3.0   62    69    5   MOLECULE: ATP SYNTHASE SUBUNIT C;                                     589:  4cbk-E  5.8  3.2   64    69    5   MOLECULE: ATP SYNTHASE SUBUNIT C;                                     590:  4cbk-B  5.8  3.0   62    69    5   MOLECULE: ATP SYNTHASE SUBUNIT C;                                     591:  4cbk-G  5.8  3.0   62    69    5   MOLECULE: ATP SYNTHASE SUBUNIT C;                                     592:  2x2v-C  5.8  2.9   62    69    5   MOLECULE: ATP SYNTHASE SUBUNIT C;                                     593:  4cbk-I  5.8  3.0   62    69    5   MOLECULE: ATP SYNTHASE SUBUNIT C;                                     594:  4mtx-C  5.8  2.0   59    97    7   MOLECULE: ETHYLENE RESPONSE SENSOR 1;                                 595:  3etx-A  5.8  3.1   79   106    9   MOLECULE: ADHESIN A;                                                  596:  6x80-M  5.8  2.4   85   574    0   MOLECULE: FLAGELLIN A;                                                597:  4g2k-A  5.8  2.3   77   110   12   MOLECULE: GENERAL CONTROL PROTEIN GCN4, ENVELOPE GLYCOPROTE           598:  4pd3-B  5.8  2.9   71   930   11   MOLECULE: NONMUSCLE MYOSIN HEAVY CHAIN B, ALPHA-ACTININ A C           599:  4uxz-F  5.8  3.4   67    95    3   MOLECULE: DIACYLGLYCEROL KINASE-DELTA 7;                              600:  7p2y-K  5.8  4.0   73    80    7   MOLECULE: ATP SYNTHASE SUBUNIT ALPHA;                                 601:  5f5p-G  5.8  4.6   83   180    6   MOLECULE: PROTEIN SHROOM2;                                            602:  6jyi-A  5.8  2.7   62   174    5   MOLECULE: TRANSCRIPTIONAL REPRESSOR PADR;                             603:  2x2v-A  5.8  2.9   62    69    5   MOLECULE: ATP SYNTHASE SUBUNIT C;                                     604:  3bvh-A  5.8  2.6   60    62   12   MOLECULE: FIBRINOGEN ALPHA CHAIN;                                     605:  6quz-D  5.8  2.2   69   574   13   MOLECULE: ABC TRANSPORTER, ATP-BINDING PROTEIN;                       606:  7p2y-H  5.8  3.6   69    79    7   MOLECULE: ATP SYNTHASE SUBUNIT ALPHA;                                 607:  5nfd-A  5.8  1.9   63    75   11   MOLECULE: KINESIN-LIKE PROTEIN KIF21A;                                608:  1f23-B  5.8  2.3   64    72    8   MOLECULE: TRANSMEMBRANE GLYCOPROTEIN;                                 609:  2hod-G  5.8  2.7   67    74   10   MOLECULE: FIBRINOGEN ALPHA CHAIN;                                     610:  5ee7-A  5.8  2.1   59   416    5   MOLECULE: GLUCAGON RECEPTOR,ENDOLYSIN,GLUCAGON RECEPTOR;              611:  1rf1-A  5.8  2.6   62    64    5   MOLECULE: FIBRINOGEN ALPHA/ALPHA-E CHAIN;                             612:  6l48-A  5.8  3.1   65   397    5   MOLECULE: STEROL O-ACYLTRANSFERASE 1;                                 613:  5n77-A  5.8  2.0   74   257    7   MOLECULE: MAGNESIUM TRANSPORT PROTEIN CORA;                           614:  8sqm-A  5.8  5.5   82  1398   13   MOLECULE: METAL RESISTANCE PROTEIN YCF1;                              615:  4xh3-A  5.8  2.5   64   214   11   MOLECULE: ACTIN-BINDING PROTEIN ANILLIN;                              616:  1df5-A  5.8  2.4   64    68   11   MOLECULE: HIV-1 ENVELOPE GLYCOPROTEIN GP41;                           617:  7enj-V  5.8  2.8   61   130    7   MOLECULE: MEDIATOR OF RNA POLYMERASE II TRANSCRIPTION SUBUN           618:  3vbb-B  5.8  2.7   76   454    7   MOLECULE: SERYL-TRNA SYNTHETASE, CYTOPLASMIC;                         619:  4cbk-M  5.8  3.2   64    69    5   MOLECULE: ATP SYNTHASE SUBUNIT C;                                     620:  4cbk-D  5.8  3.0   63    69    8   MOLECULE: ATP SYNTHASE SUBUNIT C;                                     621:  3udc-B  5.8  4.5   66   267    8   MOLECULE: SMALL-CONDUCTANCE MECHANOSENSITIVE CHANNEL, C-TER           622:  1yce-A  5.8  5.0   80    89    4   MOLECULE: SUBUNIT C;                                                  623:  1yce-a  5.8  5.0   80    89    3   MOLECULE: SUBUNIT C;                                                  624:  4fi5-A  5.8  2.4   63    71    6   MOLECULE: NUCLEOPROTEIN;                                              625:  8hio-A  5.8  2.2   61   567   10   MOLECULE: RNA (56-MER);                                               626:  6reu-I  5.8  3.6   67    74    6   MOLECULE: MITOCHONDRIAL ATP SYNTHASE SUBUNIT C;                       627:  5vi5-D  5.8  5.0   80  1238    8   MOLECULE: DNA (49-MER);                                               628:  5hkv-V  5.8  1.4   54    65   13   MOLECULE: 23S RIBOSOMAL RNA;                                          629:  5lki-E  5.8  2.7   81  1427    9   MOLECULE: TCDA1;                                                      630:  7kal-E  5.8  2.3   64   172    5   MOLECULE: PROTEIN TRANSPORT CHANNEL SEC61 COMPLEX, ALPHA SU           631:  2x2v-M  5.8  2.9   62    69    5   MOLECULE: ATP SYNTHASE SUBUNIT C;                                     632:  7c53-C  5.8  2.5   72    91    7   MOLECULE: SPIKE PROTEIN S2',PAN-COVS INHIBITOR EK1;                   633:  4zop-B  5.8  2.4   85   240    5   MOLECULE: PHOSPHATIDYLINOSITOL 4,5-BISPHOSPHATE 3-KINASE CA           634:  8ts8-B  5.8  2.5   82   262    6   MOLECULE: PHOSPHATIDYLINOSITOL 4,5-BISPHOSPHATE 3-KINASE CA           635:  4j7k-C  5.8  2.3   73    91   10   MOLECULE: SECRETED PROTEIN ESXB;                                      636:  8tsa-B  5.8  3.1   85   272    6   MOLECULE: PHOSPHATIDYLINOSITOL 4,5-BISPHOSPHATE 3-KINASE CA           637:  3zbh-H  5.8  2.8   76    92   11   MOLECULE: ESXA;                                                       638:  3zbh-E  5.8  2.6   75    91   11   MOLECULE: ESXA;                                                       639:  5uk8-B  5.8  2.4   85   244    5   MOLECULE: PHOSPHATIDYLINOSITOL 4,5-BISPHOSPHATE 3-KINASE CA           640:  3zbh-A  5.8  2.2   74    90    9   MOLECULE: ESXA;                                                       641:  8pqw-A  5.8  3.1   86  2892   10   MOLECULE: CYTOPLASMIC DYNEIN 1 HEAVY CHAIN 1;                         642:  1wnc-A  5.8  1.9   64    69   23   MOLECULE: E2 GLYCOPROTEIN;                                            643:  5sxa-B  5.8  3.1   78   218    9   MOLECULE: PHOSPHATIDYLINOSITOL 4,5-BISPHOSPHATE 3-KINASE CA           644:  6djl-E  5.7  3.4   82   226    7   MOLECULE: RAS-RELATED PROTEIN RAB-11A;                                645:  7o3x-B  5.7  2.8   74   215   12   MOLECULE: PROTEIN SLL0617;                                            646:  6m9t-A  5.7  3.7   68   444    6   MOLECULE: PROSTAGLANDIN E2 RECEPTOR EP3 SUBTYPE, ENDOLYSIN            647:  8i3j-A  5.7  2.4   71   277   11   MOLECULE: DYNEIN AXONEMAL HEAVY CHAIN 1;                              648:  6zh3-A  5.7  3.3   83   170    7   MOLECULE: VACUOLAR PROTEIN-SORTING-ASSOCIATED PROTEIN 24;             649:  3mxz-A  5.7  2.4   75   107    5   MOLECULE: TUBULIN-SPECIFIC CHAPERONE A;                               650:  5c21-A  5.7  2.7   84   267    7   MOLECULE: CHROMOSOMAL HEMOLYSIN D;                                    651:  2efl-A  5.7  2.2   77   281    8   MOLECULE: FORMIN-BINDING PROTEIN 1;                                   652:  5xg2-A  5.7  2.7   85   237    4   MOLECULE: CHROMOSOME PARTITION PROTEIN SMC;                           653:  3ogi-B  5.7  2.9   70    89    3   MOLECULE: PUTATIVE ESAT-6-LIKE PROTEIN 6;                             654:  6nr8-4  5.7  2.4   82   104    6   MOLECULE: PREFOLDIN SUBUNIT 1;                                        655:  8tzk-E  5.7  2.9   83   101    4   MOLECULE: CELL DIVISION ATP-BINDING PROTEIN FTSE;                     656:  4tko-B  5.7  3.5   82   326    5   MOLECULE: EMRA;                                                       657:  2p01-A  5.7  3.0   79   323    9   MOLECULE: ALPHA-2-MACROGLOBULIN RECEPTOR-ASSOCIATED                   658:  8pjn-b  5.7  4.0   83   296    6   MOLECULE: E3 UBIQUITIN-PROTEIN TRANSFERASE RMND5A;                    659:  4cbk-A  5.7  2.9   62    69    5   MOLECULE: ATP SYNTHASE SUBUNIT C;                                     660:  3ety-A  5.7  3.1   76   104    9   MOLECULE: ADHESIN A;                                                  661:  4cbk-J  5.7  3.0   62    69    5   MOLECULE: ATP SYNTHASE SUBUNIT C;                                     662:  5fij-W  5.7  2.9   67   217    4   MOLECULE: ATP SYNTHASE SUBUNIT ALPHA, MITOCHONDRIAL;                  663:  4cbk-K  5.7  3.0   62    69    5   MOLECULE: ATP SYNTHASE SUBUNIT C;                                     664:  6d04-E  5.7  3.9   79   466   16   MOLECULE: TRANSFERRIN RECEPTOR PROTEIN 1;                             665:  4n21-B  5.7  2.5   80   123   10   MOLECULE: GP2 ECTODOMAIN;                                             666:  4mrr-A  5.7  4.5   82   601   12   MOLECULE: ABC TRANSPORTER RELATED PROTEIN;                            667:  2x2v-H  5.7  3.0   62    69    5   MOLECULE: ATP SYNTHASE SUBUNIT C;                                     668:  7p3w-S  5.7  4.0   73    80    7   MOLECULE: ATP SYNTHASE SUBUNIT ALPHA;                                 669:  4uxx-A  5.7  3.1   66   114   11   MOLECULE: DIACYLGLYCEROL KINASE;                                      670:  4jpr-A  5.7  2.7   71    76   10   MOLECULE: ASLV FUSION TM;                                             671:  4nkg-D  5.7  1.8   58    65    9   MOLECULE: E3 UBIQUITIN-PROTEIN LIGASE SSPH1;                          672:  2dw4-A  5.7  3.1   80   634   10   MOLECULE: LYSINE-SPECIFIC HISTONE DEMETHYLASE 1;                      673:  5uh7-A  5.7  5.1   80   131    9   MOLECULE: DNA-DIRECTED RNA POLYMERASE SUBUNIT BETA';                  674:  6qum-G  5.7  3.1   81   207   15   MOLECULE: V-TYPE ATP SYNTHASE ALPHA CHAIN;                            675:  3h32-A  5.7  2.7   66    74   11   MOLECULE: FIBRINOGEN ALPHA CHAIN;                                     676:  7p3n-J  5.7  4.4   72    80    8   MOLECULE: ATP SYNTHASE SUBUNIT ALPHA;                                 677:  2hpc-A  5.7  2.7   66    74    9   MOLECULE: FIBRINOGEN ALPHA CHAIN;                                     678:  8akr-0  5.7  2.8   81   196    7   MOLECULE: CHLOROPLAST MEMBRANE-ASSOCIATED 30 KD PROTEIN;              679:  8akr-I  5.7  2.8   81   196    7   MOLECULE: CHLOROPLAST MEMBRANE-ASSOCIATED 30 KD PROTEIN;              680:  8akr-p  5.7  2.9   81   196    7   MOLECULE: CHLOROPLAST MEMBRANE-ASSOCIATED 30 KD PROTEIN;              681:  6jo5-B  5.7  2.4   67   732    9   MOLECULE: PHOTOSYSTEM I P700 CHLOROPHYLL A APOPROTEIN A1;             682:  5j0j-B  5.7  2.0   63    69    8   MOLECULE: DESIGNED PROTEIN 2L6HC3_6;                                  683:  3cp1-A  5.7  2.2   65    73    5   MOLECULE: TRANSMEMBRANE PROTEIN;                                      684:  2xaq-A  5.7  3.0   79   666    4   MOLECULE: LYSINE-SPECIFIC HISTONE DEMETHYLASE 1;                      685:  7mq3-A  5.7  1.3   49    83    4   MOLECULE: COPPER-SENSING TRANSCRIPTIONAL REPRESSOR CSOR;              686:  4cbk-L  5.7  3.2   64    69    5   MOLECULE: ATP SYNTHASE SUBUNIT C;                                     687:  3t9n-A  5.7  4.6   66   266    8   MOLECULE: SMALL-CONDUCTANCE MECHANOSENSITIVE CHANNEL;                 688:  1ykh-B  5.7  1.6   56   114    4   MOLECULE: RNA POLYMERASE II MEDIATOR COMPLEX PROTEIN MED7;            689:  6dlm-B  5.7  2.8   64    72   13   MOLECULE: DHD127_A;                                                   690:  7uuw-L  5.7  1.7   60   159    7   MOLECULE: ACTIN, ALPHA SKELETAL MUSCLE;                               691:  6oqs-R  5.7  3.6   66    76    6   MOLECULE: ATP SYNTHASE SUBUNIT DELTA;                                 692:  6r10-U  5.7  3.8   64    73    8   MOLECULE: V-TYPE ATP SYNTHASE ALPHA CHAIN;                            693:  5u0s-V  5.7  2.6   66   118    9   MOLECULE: MEDIATOR COMPLEX SUBUNIT 6;                                 694:  6s1k-F  5.7  2.8   78   102   12   MOLECULE: CHEMOTAXIS PROTEIN CHEA;                                    695:  8sbj-B  5.7  2.5   85   263    5   MOLECULE: PHOSPHATIDYLINOSITOL 4,5-BISPHOSPHATE 3-KINASE CA           696:  8cvi-V  5.7  2.1   83   269    5   MOLECULE: FLAGELLIN;                                                  697:  8cvi-K  5.7  2.1   83   269    5   MOLECULE: FLAGELLIN;                                                  698:  3zbh-G  5.7  2.5   75    93    9   MOLECULE: ESXA;                                                       699:  5ul1-B  5.7  2.5   85   257    5   MOLECULE: PHOSPHATIDYLINOSITOL 4,5-BISPHOSPHATE 3-KINASE CA           700:  8tsc-B  5.7  2.6   84   270    5   MOLECULE: PHOSPHATIDYLINOSITOL 4,5-BISPHOSPHATE 3-KINASE CA           701:  7nyz-B  5.7  2.5   82  1467    5   MOLECULE: CHROMOSOME PARTITION PROTEIN MUKB;                          702:  5ukj-B  5.7  2.5   85   257    5   MOLECULE: PHOSPHATIDYLINOSITOL 4,5-BISPHOSPHATE 3-KINASE CA           703:  7myo-B  5.7  2.6   85   286    6   MOLECULE: PHOSPHATIDYLINOSITOL 3-KINASE REGULATORY SUBUNIT            704:  8cpz-B  5.6  3.1   80  2122    9   MOLECULE: TCDA1;                                                      705:  2zqm-A  5.6  2.9   81   114    4   MOLECULE: PREFOLDIN BETA SUBUNIT 1;                                   706:  4gfq-A  5.6  4.1   72   186   13   MOLECULE: RIBOSOME-RECYCLING FACTOR;                                  707:  7emf-K  5.6  2.0   62   112    5   MOLECULE: MEDIATOR OF RNA POLYMERASE II TRANSCRIPTION SUBUN           708:  7a6h-O  5.6  3.7   74   512    7   MOLECULE: DNA-DIRECTED RNA POLYMERASE III SUBUNIT RPC1;               709:  7eeb-L  5.6  5.6   76   597    7   MOLECULE: ENHANCED GREEN FLUORESCENT PROTEIN,CATION CHANNEL           710:  6c48-D  5.6  3.8   75    87    5   MOLECULE: PROTEIN LIN-9 HOMOLOG;                                      711:  8ga7-A  5.6  3.9   73   503    7   MOLECULE: THR5;                                                       712:  7qlr-A  5.6  2.8   73   603    8   MOLECULE: CDHS1_22 PUTATIVE TAIL FIBER PROTEIN;                       713:  4kqt-A  5.6  4.7   82   169    7   MOLECULE: PUTATIVE OUTER MEMBRANE CHAPERONE (OMPH-LIKE);              714:  5ew5-A  5.6  2.3   84   490    4   MOLECULE: COLICIN-E9;                                                 715:  8ap9-G  5.6  2.3   71   279   10   MOLECULE: ATP SYNTHASE GAMMA SUBUNIT;                                 716:  3qwe-A  5.6  2.2   70   260    7   MOLECULE: GEM-INTERACTING PROTEIN;                                    717:  6rw8-B  5.6  2.6   79  2337    5   MOLECULE: A COMPONENT OF INSECTICIDAL TOXIN COMPLEX (TC);             718:  4cbk-H  5.6  3.0   62    69    5   MOLECULE: ATP SYNTHASE SUBUNIT C;                                     719:  2gts-A  5.6  2.4   68    77    9   MOLECULE: HYPOTHETICAL PROTEIN HP0062;                                720:  8f6h-B  5.6  3.7   72   271    6   MOLECULE: CADMIUM AND ZINC EFFLUX PUMP FIEF;                          721:  2b5u-A  5.6  2.6   79   470    9   MOLECULE: COLICIN E3;                                                 722:  7ek1-A  5.6  3.1   69   336    4   MOLECULE: BESTROPHIN-LIKE PROTEIN;                                    723:  2oex-B  5.6  5.9   83   341    5   MOLECULE: PROGRAMMED CELL DEATH 6-INTERACTING PROTEIN;                724:  4biz-D  5.6  4.8   72   214    7   MOLECULE: SENSOR PROTEIN CPXA;                                        725:  4n21-A  5.6  2.6   80   123   10   MOLECULE: GP2 ECTODOMAIN;                                             726:  4n23-A  5.6  2.6   81   123   11   MOLECULE: GP2 ECTODOMAIN;                                             727:  7p3w-O  5.6  4.6   72    80    7   MOLECULE: ATP SYNTHASE SUBUNIT ALPHA;                                 728:  3q4h-A  5.6  2.8   73    90    8   MOLECULE: PE FAMILY PROTEIN;                                          729:  7qha-A  5.6  1.8   56   153    9   MOLECULE: PUTATIVE TRAP-TYPE C4-DICARBOXYLATE TRANSPORT SYS           730:  7o3y-B  5.6  2.6   81   215    9   MOLECULE: PROTEIN SLL0617;                                            731:  3u0c-A  5.6  2.8   81   151    6   MOLECULE: INVASIN IPAB;                                               732:  8y3x-E  5.6  3.1   80   111   10   MOLECULE: CELL DIVISION ATP-BINDING PROTEIN FTSE;                     733:  5l0g-B  5.6  2.7   67   172   13   MOLECULE: VINCULIN;                                                   734:  6j54-K  5.6  4.2   66    72    2   MOLECULE: ATP SYNTHASE PERIPHERAL STALK-MEMBRANE SUBUNIT B;           735:  3msv-A  5.6  2.9   61   352    8   MOLECULE: NUCLEAR IMPORT ADAPTOR, NRO1;                               736:  6lqo-I  5.6  3.0   68    88    4   MOLECULE: CYTOPLASMIC ENVELOPMENT PROTEIN 1;                          737:  8d06-C  5.6  2.8   61    67    3   MOLECULE: HALC3_104;                                                  738:  6cnj-A  5.6  2.9   59   370   10   MOLECULE: NEURONAL ACETYLCHOLINE RECEPTOR SUBUNIT ALPHA-4;            739:  3h32-D  5.6  2.7   66    74   11   MOLECULE: FIBRINOGEN ALPHA CHAIN;                                     740:  8akr-J  5.6  2.9   81   196    7   MOLECULE: CHLOROPLAST MEMBRANE-ASSOCIATED 30 KD PROTEIN;              741:  8akr-o  5.6  2.9   81   196    7   MOLECULE: CHLOROPLAST MEMBRANE-ASSOCIATED 30 KD PROTEIN;              742:  3jrt-A  5.6  2.8   66   166    2   MOLECULE: INTEGRON CASSETTE PROTEIN VPC_CASS2;                        743:  6tdx-O  5.6  3.2   69    81    3   MOLECULE: ATP SYNTHASE F1 SUBUNIT GAMMA;                              744:  6v6d-A  5.6  2.9   67   209    9   MOLECULE: PANNEXIN-1;                                                 745:  8d06-K  5.6  2.7   60    66    3   MOLECULE: HALC3_104;                                                  746:  1gax-B  5.6  2.8   63   863   13   MOLECULE: TRNA(VAL);                                                  747:  7o3y-C  5.6  2.6   81   215    9   MOLECULE: PROTEIN SLL0617;                                            748:  5d56-E  5.6  3.2   66    89    3   MOLECULE: DIACYLGLYCEROL KINASE;                                      749:  6res-B  5.6  3.7   67    74    6   MOLECULE: ASA-10: POLYTOMELLA F-ATP SYNTHASE ASSOCIATED SUB           750:  6reu-B  5.6  3.7   67    74    6   MOLECULE: MITOCHONDRIAL ATP SYNTHASE SUBUNIT C;                       751:  6ret-B  5.6  3.7   67    74    6   MOLECULE: ASA-10: POLYTOMELLA F-ATP SYNTHASE ASSOCIATED SUB           752:  8soi-A  5.6  3.1   85   505    8   MOLECULE: RB1-INDUCIBLE COILED-COIL PROTEIN 1;                        753:  2axt-z  5.6  2.0   58    62    5   MOLECULE: PHOTOSYSTEM Q(B) PROTEIN;                                   754:  5odw-C  5.6  2.7   69   195   12   MOLECULE: FERRIPYOVERDINE RECEPTOR;                                   755:  4s0f-B  5.6  3.2   74   565    4   MOLECULE: ABC-TYPE BACTERIOCIN TRANSPORTER;                           756:  4whe-A  5.6  2.7   82   137   12   MOLECULE: PHAGE SHOCK PROTEIN A;                                      757:  8akr-d  5.6  2.8   81   196    7   MOLECULE: CHLOROPLAST MEMBRANE-ASSOCIATED 30 KD PROTEIN;              758:  5un6-E  5.6  4.0   78    89    5   MOLECULE: FRIZZLED-8;                                                 759:  7p3n-K  5.6  4.1   72    79    7   MOLECULE: ATP SYNTHASE SUBUNIT ALPHA;                                 760:  1re3-A  5.6  2.0   58    60   12   MOLECULE: FIBRINOGEN ALPHA/ALPHA-E CHAIN;                             761:  8v8i-D  5.6  2.8   83   222    4   MOLECULE: PHOSPHATIDYLINOSITOL 4,5-BISPHOSPHATE 3-KINASE CA           762:  5sxk-B  5.6  2.8   76   257    8   MOLECULE: PHOSPHATIDYLINOSITOL 4,5-BISPHOSPHATE 3-KINASE CA           763:  8ts7-B  5.6  2.5   83   266    6   MOLECULE: PHOSPHATIDYLINOSITOL 4,5-BISPHOSPHATE 3-KINASE CA           764:  3wuq-A  5.6  2.6   70   263    7   MOLECULE: CYTOPLASMIC DYNEIN 1 HEAVY CHAIN 1;                         765:  5j0k-B  5.6  2.7   65    73   14   MOLECULE: DESIGNED PROTEIN 2L4HC2_23;                                 766:  7sn7-O  5.6  2.1   83   546    5   MOLECULE: FLAGELLIN;                                                  767:  8cvi-D  5.6  2.9   78   268    6   MOLECULE: FLAGELLIN;                                                  768:  6vld-B  5.5  2.9   63   470    6   MOLECULE: ALPHA-(1,6)-FUCOSYLTRANSFERASE;                             769:  8w20-A  5.5  2.4   82   732    7   MOLECULE: SECRETED PROTEIN;                                           770:  8gjg-A  5.5  1.8   61   162    8   MOLECULE: GLUC_A04_0005 BINDER;                                       771:  2v0o-A  5.5  2.2   74   273    7   MOLECULE: FCH DOMAIN ONLY PROTEIN 2;                                  772:  7p5j-A  5.5  4.1   82   399    7   MOLECULE: PROTEIN TWEETY HOMOLOG 1;                                   773:  3av0-B  5.5  5.4   72   365    6   MOLECULE: DNA DOUBLE-STRAND BREAK REPAIR PROTEIN MRE11;               774:  7p2y-J  5.5  5.0   75    80    7   MOLECULE: ATP SYNTHASE SUBUNIT ALPHA;                                 775:  6y1y-B  5.5  3.0   85   128    8   MOLECULE: CHEA;                                                       776:  7qcd-B  5.5  3.4   82   998    7   MOLECULE: STRUCTURAL MAINTENANCE OF CHROMOSOMES PROTEIN 5;            777:  8eav-H  5.5  4.8   75   159    0   MOLECULE: YAR027W OR YAR028W;                                         778:  6dfk-K  5.5  3.0   79   223    5   MOLECULE: SUBUNIT OF PROTEASEOME ACTIVATOR COMPLEX,PUTATIVE           779:  7o3v-J  5.5  4.4   84   260    7   MOLECULE: TRWJ PROTEIN;                                               780:  4n23-B  5.5  2.5   77   122   10   MOLECULE: GP2 ECTODOMAIN;                                             781:  4n23-C  5.5  2.5   80   124   10   MOLECULE: GP2 ECTODOMAIN;                                             782:  6b2z-J  5.5  3.3   65    75    6   MOLECULE: ATP SYNTHASE SUBUNIT C, MITOCHONDRIAL;                      783:  6x80-K  5.5  3.0   81   574    7   MOLECULE: FLAGELLIN A;                                                784:  5d56-F  5.5  3.3   66    85    3   MOLECULE: DIACYLGLYCEROL KINASE;                                      785:  7sn7-B  5.5  2.9   85   546    5   MOLECULE: FLAGELLIN;                                                  786:  5d6i-B  5.5  3.0   67   116   10   MOLECULE: DIACYLGLYCEROL KINASE;                                      787:  2ffd-A  5.5  2.8   64    65    5   MOLECULE: FIBRINOGEN ALPHA/ALPHA-E CHAIN;                             788:  2ke4-A  5.5  3.4   76    98    4   MOLECULE: CDC42-INTERACTING PROTEIN 4;                                789:  4hga-A  5.5  2.2   58   207   12   MOLECULE: DEATH DOMAIN-ASSOCIATED PROTEIN 6;                          790:  6vwk-S  5.5  4.0   68    77    6   MOLECULE: ATP SYNTHASE SUBUNIT C;                                     791:  2ch7-A  5.5  2.4   85   309   12   MOLECULE: METHYL-ACCEPTING CHEMOTAXIS PROTEIN;                        792:  8fn2-a  5.5  2.0   54    65   11   MOLECULE: 23S RIBOSOMAL RNA;                                          793:  7upo-A  5.5  2.8   65    74   11   MOLECULE: DHT03 PROTEIN A;                                            794:  1e1q-G  5.5  2.8   76   122    5   MOLECULE: BOVINE MITOCHONDRIAL F1-ATPASE;                             795:  7xp6-R  5.5  2.2   64   283   11   MOLECULE: GUANINE NUCLEOTIDE-BINDING PROTEIN G(T) SUBUNIT A           796:  1ebo-E  5.5  2.5   78   111   14   MOLECULE: EBOLA VIRUS ENVELOPE PROTEIN CHIMERA CONSISTING             797:  1ebo-C  5.5  2.4   77   111   16   MOLECULE: EBOLA VIRUS ENVELOPE PROTEIN CHIMERA CONSISTING             798:  5hfm-A  5.5  2.6   70    77   14   MOLECULE: ENVELOPE GLYCOPROTEIN GP160,GP41 CHR REGION;                799:  2rld-B  5.5  1.8   53   112    4   MOLECULE: UNCHARACTERIZED PROTEIN;                                    800:  4cg4-A  5.5  3.5   82   376    4   MOLECULE: PYRIN;                                                      801:  8i03-H  5.5  6.7   82   126    5   MOLECULE: PAIRED AMPHIPATHIC HELIX PROTEIN PST1;                      802:  8akr-T  5.5  2.9   81   196    7   MOLECULE: CHLOROPLAST MEMBRANE-ASSOCIATED 30 KD PROTEIN;              803:  8akr-7  5.5  2.5   81   196    6   MOLECULE: CHLOROPLAST MEMBRANE-ASSOCIATED 30 KD PROTEIN;              804:  6pe4-Q  5.5  2.1   63   369    8   MOLECULE: V-TYPE PROTON ATPASE SUBUNIT A, VACUOLAR ISOFORM;           805:  6xxv-C  5.5  5.2   69   111    4   MOLECULE: ANTIBODY C57, HEAVY CHAIN;                                  806:  7p3w-L  5.5  4.6   71    79    7   MOLECULE: ATP SYNTHASE SUBUNIT ALPHA;                                 807:  7tmr-M  5.5  2.7   82   214   10   MOLECULE: H(+)-TRANSPORTING TWO-SECTOR ATPASE;                        808:  1fft-I  5.5  2.4   63   109    0   MOLECULE: UBIQUINOL OXIDASE;                                          809:  8akr-D  5.5  2.5   81   196    6   MOLECULE: CHLOROPLAST MEMBRANE-ASSOCIATED 30 KD PROTEIN;              810:  4igg-B  5.5  2.4   63   771    6   MOLECULE: CATENIN ALPHA-1;                                            811:  8akr-m  5.5  3.1   82   196    9   MOLECULE: CHLOROPLAST MEMBRANE-ASSOCIATED 30 KD PROTEIN;              812:  6dma-D  5.5  2.5   62    68    8   MOLECULE: DHD15_CLOSED_A;                                             813:  2xnd-J  5.5  3.5   65    72    2   MOLECULE: ATP SYNTHASE SUBUNIT ALPHA, MITOCHONDRIAL;                  814:  3t9o-B  5.5  2.0   58   120    2   MOLECULE: DIGUANYLATE CYCLASE DGCZ;                                   815:  6oqu-M  5.5  4.1   69    77    7   MOLECULE: ATP SYNTHASE SUBUNIT DELTA;                                 816:  5buo-A  5.5  3.8   73   185    5   MOLECULE: AMYLOID BETA A4 PROTEIN;                                    817:  6ref-I  5.5  3.6   65    74    9   MOLECULE: ASA-10: POLYTOMELLA F-ATP SYNTHASE ASSOCIATED SUB           818:  6oqr-O  5.5  3.8   67    77    6   MOLECULE: ATP SYNTHASE SUBUNIT DELTA;                                 819:  6oqu-L  5.5  3.9   67    77    6   MOLECULE: ATP SYNTHASE SUBUNIT DELTA;                                 820:  5t4p-T  5.5  3.7   68    75    9   MOLECULE: ATP SYNTHASE SUBUNIT ALPHA;                                 821:  3udc-D  5.5  4.1   68   267    6   MOLECULE: SMALL-CONDUCTANCE MECHANOSENSITIVE CHANNEL, C-TER           822:  8cye-E  5.5  2.1   83   269    5   MOLECULE: FLAGELLIN;                                                  823:  5ip0-J  5.5  4.3   76    88    8   MOLECULE: PHA GRANULE-ASSOCIATED PROTEIN;                             824:  8cvi-b  5.5  2.0   81   268    5   MOLECULE: FLAGELLIN;                                                  825:  8cye-M  5.5  2.0   81   269    5   MOLECULE: FLAGELLIN;                                                  826:  4j41-E  5.5  2.7   70    81   14   MOLECULE: SECRETED PROTEIN ESXB;                                      827:  5t8f-B  5.5  2.7   86   169    5   MOLECULE: PHOSPHATIDYLINOSITOL 4,5-BISPHOSPHATE 3-KINASE CA           828:  3zbh-C  5.5  2.2   73    90   10   MOLECULE: ESXA;                                                       829:  5xgj-B  5.5  2.6   83   278    6   MOLECULE: PHOSPHATIDYLINOSITOL 4,5-BISPHOSPHATE 3-KINASE CA           830:  6cnn-A  5.4  2.8   71   360   10   MOLECULE: INTERMEDIATE CONDUCTANCE CALCIUM-ACTIVATED POTASS           831:  8sg4-A  5.4  4.3   79  1469   14   MOLECULE: METAL RESISTANCE PROTEIN YCF1;                              832:  6lqo-L  5.4  3.3   70    94    9   MOLECULE: CYTOPLASMIC ENVELOPMENT PROTEIN 1;                          833:  2pms-C  5.4  2.9   66   109    8   MOLECULE: LACTOTRANSFERRIN;                                           834:  7nb6-A  5.4  5.2   81   340   11   MOLECULE: AI-2 TRANSPORT PROTEIN TQSA;                                835:  6ird-B  5.4  4.3   80   254    6   MOLECULE: 1-PHOSPHATIDYLINOSITOL 4,5-BISPHOSPHATE PHOSPHODI           836:  8h2j-A  5.4  7.1   74    92   11   MOLECULE: P26;                                                        837:  6fze-A  5.4  4.2   73   209   12   MOLECULE: PUTATIVE SURFACE PROTEIN;                                   838:  4abx-A  5.4  2.3   67   167    7   MOLECULE: DNA REPAIR PROTEIN RECN;                                    839:  6z6o-D  5.4  2.3   85   542   14   MOLECULE: HISTONE DEACETYLASE HDA1;                                   840:  5szj-B  5.4  2.8   72   145    4   MOLECULE: RAS-RELATED PROTEIN RAB-10;                                 841:  3k66-A  5.4  3.9   76   217   11   MOLECULE: BETA-AMYLOID-LIKE PROTEIN;                                  842:  3q7g-A  5.4  3.9   71   203    8   MOLECULE: AMYLOID-LIKE PROTEIN 1;                                     843:  5n5e-W  5.4  2.1   57    97   11   MOLECULE: PFC_05175;                                                  844:  7nb6-B  5.4  4.6   77   340    8   MOLECULE: AI-2 TRANSPORT PROTEIN TQSA;                                845:  2iw5-A  5.4  2.7   79   666    6   MOLECULE: LYSINE-SPECIFIC HISTONE DEMETHYLASE 1;                      846:  5jsb-F  5.4  4.5   74   116   12   MOLECULE: INDUCED MYELOID LEUKEMIA CELL DIFFERENTIATION PRO           847:  6tms-E  5.4  2.7   62    69    3   MOLECULE: A NOVEL DESIGNED PORE PROTEIN;                              848:  7o3v-G  5.4  4.4   84   261    7   MOLECULE: TRWJ PROTEIN;                                               849:  5o71-A  5.4  2.4   72   474   10   MOLECULE: UBIQUITIN CARBOXYL-TERMINAL HYDROLASE 25;                   850:  6ire-A  5.4  5.9   80   223    3   MOLECULE: 1-PHOSPHATIDYLINOSITOL 4,5-BISPHOSPHATE PHOSPHODI           851:  8afz-B  5.4  3.3   83   376    2   MOLECULE: SORTING NEXIN-1;                                            852:  6f38-R  5.4  1.8   63    87    0   MOLECULE: ARP1 ACTIN RELATED PROTEIN 1 HOMOLOG A;                     853:  5gy2-C  5.4  2.5   79   169    6   MOLECULE: TLR5B PROTEIN,VARIABLE LYMPHOCYTE RECEPTOR B;               854:  6x80-G  5.4  2.7   82   574    9   MOLECULE: FLAGELLIN A;                                                855:  6x80-N  5.4  2.8   85   574    7   MOLECULE: FLAGELLIN A;                                                856:  6vjy-B  5.4  3.1   70   280    6   MOLECULE: ERAD-ASSOCIATED E3 UBIQUITIN-PROTEIN LIGASE HRD1;           857:  6zbl-D  5.4  2.3   85   498    8   MOLECULE: PRECURSOR OF THE MAJOR MEROZOITE SURFACE ANTIGENS           858:  4mt8-A  5.4  1.7   54   100   13   MOLECULE: ETHYLENE RESPONSE SENSOR 1;                                 859:  5sva-U  5.4  1.8   53   156   11   MOLECULE: DNA-DIRECTED RNA POLYMERASE II SUBUNIT RPB1;                860:  8akr-z  5.4  2.8   82   196    7   MOLECULE: CHLOROPLAST MEMBRANE-ASSOCIATED 30 KD PROTEIN;              861:  5lgn-A  5.4  2.9   77   670    6   MOLECULE: LYSINE-SPECIFIC HISTONE DEMETHYLASE 1A;                     862:  6tqj-A  5.4  3.6   70    81    6   MOLECULE: ATP SYNTHASE SUBUNIT C, CHLOROPLASTIC;                      863:  6kop-D  5.4  5.6   78  1263   12   MOLECULE: DNA-DIRECTED RNA POLYMERASE SUBUNIT ALPHA;                  864:  8d06-B  5.4  2.9   61    67    3   MOLECULE: HALC3_104;                                                  865:  7k3h-A  5.4  1.5   57   104    7   MOLECULE: NETWORK HALLUCINATED PROTEIN 0217;                          866:  3mq1-B  5.4  2.7   63   101   16   MOLECULE: MITE ALLERGEN DER P 5;                                      867:  5b1n-A  5.4  2.7   56    59   13   MOLECULE: OSMOLARITY SENSOR PROTEIN ENVZ;                             868:  6ek9-A  5.4  2.3   58   121    9   MOLECULE: CYTOSOLIC COPPER STORAGE PROTEIN;                           869:  2hod-A  5.4  2.9   66    74    9   MOLECULE: FIBRINOGEN ALPHA CHAIN;                                     870:  6tej-A  5.4  1.9   58   561   10   MOLECULE: DRUG ABC TRANSPORTER ATP-BINDING PROTEIN;                   871:  1i5x-A  5.4  2.3   58    61   12   MOLECULE: TRANSMEMBRANE GLYCOPROTEIN (GP41);                          872:  3ajw-A  5.4  2.6   83   134    7   MOLECULE: FLAGELLAR FLIJ PROTEIN;                                     873:  6th1-R  5.4  6.2   76   360   12   MOLECULE: IMMEDIATE EARLY PROTEIN 1;                                  874:  7tmp-M  5.4  2.5   66   214    5   MOLECULE: H(+)-TRANSPORTING TWO-SECTOR ATPASE;                        875:  8akr-h  5.4  2.5   81   196    6   MOLECULE: CHLOROPLAST MEMBRANE-ASSOCIATED 30 KD PROTEIN;              876:  4zwt-E  5.4  5.0   82   126    4   MOLECULE: RECOMBINATION PROTEIN UVSY;                                 877:  6rz8-A  5.4  2.7   68   366    7   MOLECULE: CYSTEINYL LEUKOTRIENE RECEPTOR 2,SOLUBLE CYTOCHRO           878:  3lay-E  5.4  5.0   70    78    9   MOLECULE: ZINC RESISTANCE-ASSOCIATED PROTEIN;                         879:  8h39-B  5.4  4.0   69    90   10   MOLECULE: P26;                                                        880:  6n9h-A  5.4  3.0   69    75    6   MOLECULE: AMANTADINE-BINDING PROTEIN;                                 881:  1fft-D  5.4  2.4   62   109    0   MOLECULE: UBIQUINOL OXIDASE;                                          882:  6h2x-A  5.4  3.4   81   352    5   MOLECULE: CHROMOSOME PARTITION PROTEIN MUKB,CHROMOSOME PART           883:  3teq-D  5.4  4.5   82   100    6   MOLECULE: STROMAL INTERACTION MOLECULE 1;                             884:  7utj-K  5.4  1.7   60   156    8   MOLECULE: ACTIN, ALPHA SKELETAL MUSCLE;                               885:  4brb-F  5.4  2.9   66    92    3   MOLECULE: DIACYLGLYCEROL KINASE;                                      886:  7utj-G  5.4  1.7   60   156    8   MOLECULE: ACTIN, ALPHA SKELETAL MUSCLE;                               887:  7utj-I  5.4  1.7   60   156    8   MOLECULE: ACTIN, ALPHA SKELETAL MUSCLE;                               888:  3udc-G  5.4  4.5   66   267    8   MOLECULE: SMALL-CONDUCTANCE MECHANOSENSITIVE CHANNEL, C-TER           889:  7utj-Z  5.4  1.7   60   156    8   MOLECULE: ACTIN, ALPHA SKELETAL MUSCLE;                               890:  7utj-L  5.4  1.7   60   156    8   MOLECULE: ACTIN, ALPHA SKELETAL MUSCLE;                               891:  6iac-A  5.4  2.8   70   301    7   MOLECULE: PORTAL PROTEIN;                                             892:  1jch-A  5.4  2.2   82   468   12   MOLECULE: COLICIN E3;                                                 893:  2p22-C  5.4  2.5   60   186   10   MOLECULE: SUPPRESSOR PROTEIN STP22 OF TEMPERATURE-SENSITIVE           894:  6oqs-J  5.4  4.1   70    77    7   MOLECULE: ATP SYNTHASE SUBUNIT DELTA;                                 895:  6vyl-E  5.4  4.6   60   256    7   MOLECULE: MECHANOSENSITIVE CHANNEL MSCS;                              896:  3nyl-A  5.4  3.8   76   196    5   MOLECULE: AMYLOID BETA (A4) PROTEIN (PEPTIDASE NEXIN-II, AL           897:  2ic6-B  5.4  2.5   67    76   13   MOLECULE: NUCLEOCAPSID PROTEIN;                                       898:  5vou-H  5.4  2.9   63   175    5   MOLECULE: GLUTAMATE RECEPTOR 2;                                       899:  4n21-C  5.4  2.0   75   124    9   MOLECULE: GP2 ECTODOMAIN;                                             900:  6res-F  5.4  3.5   65    74    9   MOLECULE: ASA-10: POLYTOMELLA F-ATP SYNTHASE ASSOCIATED SUB           901:  4jio-A  5.4  4.2   82   310    5   MOLECULE: BRO1;                                                       902:  8h39-A  5.4  6.7   73    92   10   MOLECULE: P26;                                                        903:  5mmi-Z  5.4  1.7   55   101   13   MOLECULE: 50S RIBOSOMAL PROTEIN L31;                                  904:  6pqv-Q  5.4  4.1   70    77    7   MOLECULE: ATP SYNTHASE SUBUNIT DELTA;                                 905:  3onx-A  5.4  3.7   76   125   14   MOLECULE: BUD SITE SELECTION PROTEIN 6;                               906:  5xgh-B  5.4  2.6   85   277    6   MOLECULE: PHOSPHATIDYLINOSITOL 4,5-BISPHOSPHATE 3-KINASE CA           907:  8cvi-T  5.4  3.1   76   269    5   MOLECULE: FLAGELLIN;                                                  908:  5wjt-a  5.4  3.1   78   302   12   MOLECULE: FLAGELLIN;                                                  909:  4j41-C  5.4  2.7   68    81   15   MOLECULE: SECRETED PROTEIN ESXB;                                      910:  8dd8-B  5.4  2.5   85   261    5   MOLECULE: PHOSPHATIDYLINOSITOL 4,5-BISPHOSPHATE 3-KINASE CA           911:  4j41-F  5.4  2.5   66    80   15   MOLECULE: SECRETED PROTEIN ESXB;                                      912:  7sn7-F  5.4  2.2   83   546    5   MOLECULE: FLAGELLIN;                                                  913:  3k8v-A  5.4  2.4   82   323    6   MOLECULE: FLAGELLIN HOMOLOG;                                          914:  3k8w-A  5.4  2.3   85   296    5   MOLECULE: FLAGELLIN HOMOLOG;                                          915:  4aki-A  5.4  3.3   73  2650   10   MOLECULE: GLUTATHIONE S-TRANSFERASE CLASS-MU 26 KDA ISOZYME           916:  4mh6-A  5.3  2.3   82   159   10   MOLECULE: PUTATIVE TYPE III SECRETION PROTEIN YSCO;                   917:  6bog-A  5.3  3.7   63   967    2   MOLECULE: RNA POLYMERASE-ASSOCIATED PROTEIN RAPA;                     918:  5wkq-A  5.3  3.7   83   166    7   MOLECULE: INVASIN IPAB;                                               919:  6nr8-1  5.3  3.0   82   107    6   MOLECULE: PREFOLDIN SUBUNIT 1;                                        920:  7tve-D  5.3  3.9   78   445   10   MOLECULE: DNA (68-MER);                                               921:  6zbj-A  5.3  2.2   71   666    7   MOLECULE: PRECURSOR OF THE MAJOR MEROZOITE SURFACE ANTIGENS           922:  4kxr-A  5.3  2.9   71    85    7   MOLECULE: PE25;                                                       923:  6xi6-A  5.3  3.5   78   269    5   MOLECULE: HELICAL FUSION DESIGN;                                      924:  6em5-b  5.3  3.8   79   421    4   MOLECULE: 5.8S RIBOSOMAL RNA;                                         925:  5c1f-A  5.3  2.5   73   298    7   MOLECULE: SEPTATION PROTEIN IMP2;                                     926:  6pwn-A  5.3  4.2   67   280    1   MOLECULE: SMALL-CONDUCTANCE MECHANOSENSITIVE CHANNEL;                 927:  7nb6-C  5.3  4.4   77   340    8   MOLECULE: AI-2 TRANSPORT PROTEIN TQSA;                                928:  5j0l-F  5.3  3.9   64   107    8   MOLECULE: DESIGNED PROTEIN 3L6HC2_2;                                  929:  8i03-F  5.3  2.6   76   228   11   MOLECULE: PAIRED AMPHIPATHIC HELIX PROTEIN PST1;                      930:  8cr1-C  5.3  6.9   82   266    4   MOLECULE: ATPASE ASNA1;                                               931:  6nuw-F  5.3  5.2   59   190    8   MOLECULE: INNER KINETOCHORE SUBUNIT IML3;                             932:  1grj-A  5.3  2.9   63   151    5   MOLECULE: GREA PROTEIN;                                               933:  3bvo-A  5.3  2.3   58   197   12   MOLECULE: CO-CHAPERONE PROTEIN HSCB, MITOCHONDRIAL PRECURSO           934:  6msr-A  5.3  2.5   66    76    5   MOLECULE: PRO-2.5;                                                    935:  4cqi-A  5.3  2.4   71   107    6   MOLECULE: TUBULIN-BINDING COFACTOR A;                                 936:  3ghg-D  5.3  3.0   77   174    6   MOLECULE: FIBRINOGEN ALPHA CHAIN;                                     937:  5hfm-C  5.3  2.6   67    77   13   MOLECULE: ENVELOPE GLYCOPROTEIN GP160,GP41 CHR REGION;                938:  3p8l-A  5.3  1.3   54   283    6   MOLECULE: GERANYLTRANSTRANSFERASE;                                    939:  6x80-P  5.3  2.9   85   574    7   MOLECULE: FLAGELLIN A;                                                940:  4v1o-Z  5.3  1.6   57    88    4   MOLECULE: DNA-DIRECTED RNA POLYMERASE II SUBUNIT RPB1;                941:  6oqv-L  5.3  3.8   67    77    6   MOLECULE: ATP SYNTHASE SUBUNIT DELTA;                                 942:  5un6-G  5.3  4.0   76    87    5   MOLECULE: FRIZZLED-8;                                                 943:  6jqa-A  5.3  2.9   75    85    5   MOLECULE: PHYTOPLASMAL EFFECTOR CAUSING PHYLLODY 1;                   944:  2rld-C  5.3  2.0   54   116   11   MOLECULE: UNCHARACTERIZED PROTEIN;                                    945:  8h2j-F  5.3  6.2   72    91   10   MOLECULE: P26;                                                        946:  5idj-A  5.3  2.6   66   242    9   MOLECULE: HISTIDINE KINASE;                                           947:  8c5v-R  5.3  1.8   78   516    9   MOLECULE: CHEMOTAXIS PROTEIN CHEA;                                    948:  4kp4-A  5.3  3.3   65   219    8   MOLECULE: OSMOLARITY SENSOR PROTEIN ENVZ, HISTIDINE KINASE;           949:  1i5y-A  5.3  2.3   58    61   12   MOLECULE: TRANSMEMBRANE GLYCOPROTEIN (GP41);                          950:  6n1l-A  5.3  2.3   71   141   11   MOLECULE: FIBRONECTIN-BINDING PROTEIN BBK32;                          951:  6wjv-A  5.3  3.3   68  1951    1   MOLECULE: DNA POLYMERASE EPSILON CATALYTIC SUBUNIT A;                 952:  6hk5-F  5.3  2.5   58    61   12   MOLECULE: COOJ;                                                       953:  6vwk-M  5.3  4.8   70    77   14   MOLECULE: ATP SYNTHASE SUBUNIT C;                                     954:  5knd-G  5.3  2.7   80   178   13   MOLECULE: V-TYPE SODIUM ATPASE CATALYTIC SUBUNIT A;                   955:  3frt-A  5.3  2.5   75   141    8   MOLECULE: CHARGED MULTIVESICULAR BODY PROTEIN 3;                      956:  3frt-B  5.3  2.2   75   142    5   MOLECULE: CHARGED MULTIVESICULAR BODY PROTEIN 3;                      957:  3frv-A  5.3  2.3   75   133    7   MOLECULE: CHARGED MULTIVESICULAR BODY PROTEIN 3;                      958:  2gd5-C  5.3  2.3   75   142    5   MOLECULE: CHARGED MULTIVESICULAR BODY PROTEIN 3;                      959:  4hwf-A  5.3  2.5   55    87    9   MOLECULE: BAG FAMILY MOLECULAR CHAPERONE REGULATOR 3;                 960:  8sq0-B  5.3  5.0   79  1402   13   MOLECULE: METAL RESISTANCE PROTEIN YCF1;                              961:  6tms-C  5.3  2.2   63    69    8   MOLECULE: A NOVEL DESIGNED PORE PROTEIN;                              962:  1skv-B  5.3  1.9   55    61    2   MOLECULE: HYPOTHETICAL 7.5 KDA PROTEIN;                               963:  4oe8-C  5.3  2.0   52    87    8   MOLECULE: INTERLEUKIN-12 SUBUNIT BETA;                                964:  4zwt-K  5.3  4.9   83   128    8   MOLECULE: RECOMBINATION PROTEIN UVSY;                                 965:  7nyw-B  5.3  3.4   80   858   14   MOLECULE: CHROMOSOME PARTITION PROTEIN MUKB;                          966:  4zws-E  5.3  2.7   70    96    7   MOLECULE: RECOMBINATION PROTEIN UVSY;                                 967:  5krw-A  5.3  4.0   80   155    6   MOLECULE: FLAGELLAR PROTEIN FLIT,FLAGELLAR HOOK-ASSOCIATED            968:  4okv-E  5.3  4.1   61    65   11   MOLECULE: HEAVY CHAIN OF 8H7 MAB;                                     969:  1ykh-A  5.3  2.3   55    95    5   MOLECULE: RNA POLYMERASE II MEDIATOR COMPLEX PROTEIN MED7;            970:  6dma-A  5.3  2.2   63    71   13   MOLECULE: DHD15_CLOSED_A;                                             971:  5jqz-A  5.3  2.4   64    75    9   MOLECULE: DE NOVO DESIGNED HOMOTETRAMER;                              972:  6dnf-A  5.3  4.6   67   225    9   MOLECULE: MITOCHONDRIAL CALCIUM UNIPORTER MCU;                        973:  1or3-A  5.3  4.5   70   136    6   MOLECULE: PROTEIN (APOLIPOPROTEIN E);                                 974:  5iuy-C  5.3  2.0   80   451   13   MOLECULE: MULTIDRUG EFFLUX OUTER MEMBRANE PROTEIN OPRN;               975:  5hfm-E  5.3  2.6   67    77   13   MOLECULE: ENVELOPE GLYCOPROTEIN GP160,GP41 CHR REGION;                976:  8h2j-C  5.3  6.2   72    91   10   MOLECULE: P26;                                                        977:  8h2j-D  5.3  4.0   69    91   10   MOLECULE: P26;                                                        978:  6s1k-G  5.3  1.8   64   102    9   MOLECULE: CHEMOTAXIS PROTEIN CHEA;                                    979:  4akh-A  5.3  4.1   76  2650    9   MOLECULE: GLUTATHIONE S-TRANSFERASE CLASS-MU 26 KDA ISOZYME           980:  4akg-A  5.3  4.1   76  2650    9   MOLECULE: GLUTATHIONE S-TRANSFERASE CLASS-MU 26 KDA ISOZYME           981:  5wjt-g  5.3  4.0   81   302    5   MOLECULE: FLAGELLIN;                                                  982:  5zuv-B  5.3  3.1   82   129    6   MOLECULE: SPIKE GLYCOPROTEIN,INHIBITOR EK1;                           983:  5zuv-A  5.3  2.8   82   130    5   MOLECULE: SPIKE GLYCOPROTEIN,INHIBITOR EK1;                           984:  7zol-A  5.3  2.6   67   150    3   MOLECULE: CAS7-11;                                                    985:  7nyz-A  5.3  2.7   84  1467   11   MOLECULE: CHROMOSOME PARTITION PROTEIN MUKB;                          986:  6z6o-O  5.3  2.9   82   548    7   MOLECULE: HISTONE DEACETYLASE HDA1;                                   987:  3n27-C  5.3  2.1   59    70    5   MOLECULE: FUSION GLYCOPROTEIN F0, LINKER, FUSION GLYCOPROTE           988:  7sn7-K  5.3  2.1   83   546    5   MOLECULE: FLAGELLIN;                                                  989:  7nyx-B  5.3  3.2   82  1467   13   MOLECULE: CHROMOSOME PARTITION PROTEIN MUKB;                          990:  6w1s-Q  5.2  2.9   73   131    5   MOLECULE: MEDIATOR OF RNA POLYMERASE II TRANSCRIPTION SUBUN           991:  6qd6-G  5.2  3.0   73   469    8   MOLECULE: MB-CHOPQ-NB207,OUTER MEMBRANE PROTEIN,MB-CHOPQ-NB           992:  6yvd-D  5.2  2.9   77   414    9   MOLECULE: CONDENSIN COMPLEX SUBUNIT 2;                                993:  6swg-B  5.2  4.5   62    74    8   MOLECULE: PERIPHILIN-1;                                               994:  4gzu-A  5.2  2.5   65   436    9   MOLECULE: FERM, RHOGEF AND PLECKSTRIN DOMAIN-CONTAINING PRO           995:  3uum-B  5.2  2.5   77   123   10   MOLECULE: UTROPHIN;                                                   996:  8hf3-A  5.2  2.7   67   295    3   MOLECULE: PALMITOYLTRANSFERASE ZDHHC9;                                997:  6xns-B  5.2  2.4   65   333   11   MOLECULE: C3_CROWN-05;                                                998:  1lrz-A  5.2  3.2   64   400    9   MOLECULE: FACTOR ESSENTIAL FOR EXPRESSION OF METHICILLIN              999:  7d5i-A  5.2  3.4   73   443   10   MOLECULE: CYTOCHROME D UBIQUINOL OXIDASE SUBUNIT 1;                  1000:  6nr8-6  5.2  2.6   73   102    8   MOLECULE: PREFOLDIN SUBUNIT 1;                                       1001:  7vv6-R  5.2  3.6   70   271   10   MOLECULE: MAS-RELATED G-PROTEIN COUPLED RECEPTOR MEMBER X2;          1002:  3etz-B  5.2  3.2   82   115   13   MOLECULE: ADHESIN A;                                                 1003:  6v22-E  5.2  3.5   76   199    5   MOLECULE: CALCIUM-ACTIVATED POTASSIUM CHANNEL SUBUNIT ALPHA          1004:  5i6r-A  5.2  2.4   76   419    9   MOLECULE: SLIT-ROBO RHO GTPASE-ACTIVATING PROTEIN 2;                 1005:  6nf4-A  5.2  3.2   64   421    6   MOLECULE: OTOPETRIN1;                                                1006:  8ifg-A  5.2  3.5   80   879    8   MOLECULE: RBAP48-RELATED WD40 REPEAT-CONTAINING PROTEIN PRW          1007:  7nhr-A  5.2  2.5   59   663   10   MOLECULE: PUTATIVE TRANSMEMBRANE PROTEIN WZC;                        1008:  6rw6-B  5.2  3.0   83  2485    7   MOLECULE: TCDA1;                                                     1009:  6grj-A  5.2  2.6   74   338   11   MOLECULE: AHLB;                                                      1010:  8cqo-B  5.2  2.8   77   174    5   MOLECULE: LIPOPROTEIN, PUTATIVE;                                     1011:  7b5m-H  5.2  2.9   63   226    6   MOLECULE: CULLIN-1;                                                  1012:  6rld-B  5.2  5.2   66   265    3   MOLECULE: SMALL-CONDUCTANCE MECHANOSENSITIVE CHANNEL;                1013:  6rld-D  5.2  5.2   66   265    3   MOLECULE: SMALL-CONDUCTANCE MECHANOSENSITIVE CHANNEL;                1014:  5uac-C  5.2  3.8   68  1342    9   MOLECULE: DNA-DIRECTED RNA POLYMERASE SUBUNIT ALPHA;                 1015:  5uah-I  5.2  5.3   76  1340    7   MOLECULE: DNA-DIRECTED RNA POLYMERASE SUBUNIT ALPHA;                 1016:  6rld-G  5.2  5.2   66   265    3   MOLECULE: SMALL-CONDUCTANCE MECHANOSENSITIVE CHANNEL;                1017:  6rld-E  5.2  5.2   66   265    3   MOLECULE: SMALL-CONDUCTANCE MECHANOSENSITIVE CHANNEL;                1018:  6cnn-B  5.2  2.6   75   360    8   MOLECULE: INTERMEDIATE CONDUCTANCE CALCIUM-ACTIVATED POTASS          1019:  4uig-A  5.2  2.6   60    91    3   MOLECULE: COPPER SENSITIVE OPERON REPRESSOR;                         1020:  8fck-D  5.2  3.4   60   666    3   MOLECULE: HAUS AUGMIN-LIKE COMPLEX SUBUNIT 1;                        1021:  4ynv-A  5.2  2.1   61   283   10   MOLECULE: ACL4;                                                      1022:  5da9-B  5.2  1.6   49   400    2   MOLECULE: PUTATIVE UNCHARACTERIZED PROTEIN,PUTATIVE UNCHARA          1023:  4q66-B  5.2  1.9   54   507    2   MOLECULE: CHS5P;                                                     1024:  3lu0-C  5.2  5.0   74  1335    4   MOLECULE: DNA-DIRECTED RNA POLYMERASE SUBUNIT ALPHA;                 1025:  6wm4-G  5.2  2.6   83   213    4   MOLECULE: V-TYPE PROTON ATPASE 116 KDA SUBUNIT A ISOFORM 1;          1026:  5ukv-B  5.2  2.7   62    74    5   MOLECULE: ATP-BINDING PROTEIN;                                       1027:  5g04-I  5.2  3.0   77   730    5   MOLECULE: ANAPHASE-PROMOTING COMPLEX SUBUNIT 1;                      1028:  7use-B  5.2  6.3   73  1099    8   MOLECULE: CYTOPLASMIC FMR1-INTERACTING PROTEIN 1;                    1029:  4abn-B  5.2  3.8   60   426    8   MOLECULE: TETRATRICOPEPTIDE REPEAT PROTEIN 5;                        1030:  5are-W  5.2  2.9   73   217   10   MOLECULE: ATP SYNTHASE SUBUNIT ALPHA, MITOCHONDRIAL;                 1031:  5uhk-B  5.2  1.8   55   120    5   MOLECULE: O-GLCNACASE TIM-BARREL DOMAIN;                             1032:  5z51-B  5.2  2.8   59    71    3   MOLECULE: DNA PRIMASE;                                               1033:  8cqn-A  5.2  2.7   80   185    9   MOLECULE: LIPOPROTEIN, PUTATIVE;                                     1034:  8akr-y  5.2  2.8   82   196    7   MOLECULE: CHLOROPLAST MEMBRANE-ASSOCIATED 30 KD PROTEIN;             1035:  4btb-A  5.2  4.4   75   231    3   MOLECULE: PROLYL 4-HYDROXYLASE SUBUNIT ALPHA-1;                      1036:  4wpc-B  5.2  2.0   79   288    5   MOLECULE: RHO GTPASE-ACTIVATING PROTEIN RGD1;                        1037:  8ioi-A  5.2  3.2   66   190   12   MOLECULE: PADR FAMILY TRANSCRIPTIONAL REGULATOR;                     1038:  5n5e-A  5.2  2.1   58    98   10   MOLECULE: PFC_05175;                                                 1039:  5zmw-A  5.2  5.4   69  1000    9   MOLECULE: SARCOPLASMIC/ENDOPLASMIC RETICULUM CALCIUM ATPASE          1040:  6aye-C  5.2  3.4   80   476   10   MOLECULE: MUCOLIPIN-3;                                               1041:  1c99-A  5.2  5.1   70    79    7   MOLECULE: PROTEOLIPID F1FO OF ATP SYNTHASE;                          1042:  2gd5-B  5.2  2.3   75   156    7   MOLECULE: CHARGED MULTIVESICULAR BODY PROTEIN 3;                     1043:  5kay-B  5.2  2.7   79   200    6   MOLECULE: SPELTER;                                                   1044:  2rkh-A  5.2  2.6   57   164    7   MOLECULE: PUTATIVE APHA-LIKE TRANSCRIPTION FACTOR;                   1045:  6hs5-A  5.2  3.0   64   249    8   MOLECULE: TSSA;                                                      1046:  5hfm-F  5.2  2.6   66    75   11   MOLECULE: ENVELOPE GLYCOPROTEIN GP160,GP41 CHR REGION;               1047:  6csv-B  5.2  4.6   73    87   11   MOLECULE: CENTROSOMAL PROTEIN OF 63 KDA,CENTROSOMAL PROTEIN          1048:  2iub-F  5.2  3.0   76   342   12   MOLECULE: DIVALENT CATION TRANSPORT-RELATED PROTEIN;                 1049:  8akr-r  5.2  2.5   84   196   13   MOLECULE: CHLOROPLAST MEMBRANE-ASSOCIATED 30 KD PROTEIN;             1050:  4nsm-A  5.2  2.4   61    71    8   MOLECULE: COLLAGEN-LIKE PROTEIN SCLB;                                1051:  1wu0-A  5.2  3.3   66    72    3   MOLECULE: ATP SYNTHASE C CHAIN;                                      1052:  5tcx-A  5.2  7.9   81   208    2   MOLECULE: CD81 ANTIGEN;                                              1053:  8h2x-A  5.2  3.5   68    92   10   MOLECULE: P26;                                                       1054:  4gwq-A  5.2  1.9   63   112    6   MOLECULE: MEDIATOR OF RNA POLYMERASE II TRANSCRIPTION SUBUN          1055:  3ofn-Y  5.2  2.4   76   115    9   MOLECULE: ATP SYNTHASE SUBUNIT ALPHA;                                1056:  4ux3-A  5.2  3.3   83   436    8   MOLECULE: STRUCTURAL MAINTENANCE OF CHROMOSOMES PROTEIN 3;           1057:  5nl6-B  5.2  2.3   71   229    6   MOLECULE: CALPONIN DOMAIN FAMILY PROTEIN;                            1058:  1fnt-c  5.2  3.8   72   198    3   MOLECULE: PROTEASOME COMPONENT C7-ALPHA;                             1059:  3l9f-A  5.2  2.6   62   170   11   MOLECULE: PUTATIVE UNCHARACTERIZED PROTEIN SMU.1604C;                1060:  1c0v-A  5.2  2.7   69    79    4   MOLECULE: PROTEIN (F1FO ATPASE SUBUNIT C);                           1061:  5ew5-D  5.2  2.7   80   490    8   MOLECULE: COLICIN-E9;                                                1062:  7f0k-B  5.2  4.7   68    77    9   MOLECULE: KINETOPLASTID MEMBRANE PROTEIN KMP-11;                     1063:  6res-I  5.2  3.5   65    74    9   MOLECULE: ASA-10: POLYTOMELLA F-ATP SYNTHASE ASSOCIATED SUB          1064:  6oqt-O  5.2  4.2   69    77    7   MOLECULE: ATP SYNTHASE SUBUNIT DELTA;                                1065:  5ofr-B  5.2  3.1   70   576    7   MOLECULE: MICROCIN-J25 EXPORT ATP-BINDING/PERMEASE PROTEIN           1066:  3n27-A  5.2  2.3   63    73    6   MOLECULE: FUSION GLYCOPROTEIN F0, LINKER, FUSION GLYCOPROTE          1067:  8gmh-E  5.2  2.8   82   137    6   MOLECULE: LXG DOMAIN-CONTAINING PROTEIN;                             1068:  6jy0-T  5.2  3.5   76   410   11   MOLECULE: FLAGELLIN;                                                 1069:  8cvi-I  5.2  3.5   76   269   12   MOLECULE: FLAGELLIN;                                                 1070:  6f40-O  5.2  4.2   73   534    7   MOLECULE: DNA-DIRECTED RNA POLYMERASE III SUBUNIT RPC1;              1071:  6ejq-G  5.2  3.0   67   132    9   MOLECULE: TERMINASE SMALL SUBUNIT;                                   1072:  5ofr-A  5.2  3.0   74   571    7   MOLECULE: MICROCIN-J25 EXPORT ATP-BINDING/PERMEASE PROTEIN           1073:  6jy0-E  5.2  3.5   80   410   10   MOLECULE: FLAGELLIN;                                                 1074:  7sn7-D  5.2  3.2   79   546    6   MOLECULE: FLAGELLIN;                                                 1075:  8ilv-B  5.2  2.9   86   265    8   MOLECULE: PHOSPHATIDYLINOSITOL 4,5-BISPHOSPHATE 3-KINASE CA          1076:  7tch-A  5.1  4.7   81   645    7   MOLECULE: BACITRACIN EXPORT PERMEASE PROTEIN BCEB;                   1077:  4wpe-A  5.1  2.3   73   275    4   MOLECULE: CYTOKINESIS PROTEIN 2;                                     1078:  8rd2-B  5.1  3.0   81   400    9   MOLECULE: INVARIANT SURFACE GLYCOPROTEIN;                            1079:  4dyl-A  5.1  3.4   81   376    7   MOLECULE: TYROSINE-PROTEIN KINASE FES/FPS;                           1080:  5me8-A  5.1  2.7   74   107   11   MOLECULE: INHIBITOR OF GROWTH PROTEIN 5;                             1081:  8q72-A  5.1  2.2   70   750    9   MOLECULE: JETC;                                                      1082:  6ogd-E  5.1  3.0   82  1078    7   MOLECULE: TOXIN SUBUNIT YENA1;                                       1083:  7usd-A  5.1  3.7   74  1185    8   MOLECULE: CYTOPLASMIC FMR1-INTERACTING PROTEIN 1;                    1084:  5frg-A  5.1  3.0   75   104    3   MOLECULE: FORMIN-BINDING PROTEIN 1-LIKE;                             1085:  7syf-A  5.1  2.3   69  1391   10   MOLECULE: PHOSPHATIDYLINOSITOL 3,4,5-TRISPHOSPHATE-DEPENDEN          1086:  6w1s-Y  5.1  2.6   68   132   10   MOLECULE: MEDIATOR OF RNA POLYMERASE II TRANSCRIPTION SUBUN          1087:  1wyy-B  5.1  2.7   78   124    8   MOLECULE: E2 GLYCOPROTEIN;                                           1088:  4dci-H  5.1  3.4   77   143   13   MOLECULE: UNCHARACTERIZED PROTEIN;                                   1089:  5f7t-L  5.1  2.6   68   133   13   MOLECULE: TRIPARTITE MOTIF-CONTAINING PROTEIN 5,SERINE--TRN          1090:  5ebz-I  5.1  2.5   84   655    7   MOLECULE: INHIBITOR OF NUCLEAR FACTOR KAPPA-B KINASE SUBUNI          1091:  5ebz-J  5.1  2.5   84   655    7   MOLECULE: INHIBITOR OF NUCLEAR FACTOR KAPPA-B KINASE SUBUNI          1092:  3zx6-B  5.1  2.1   83   303    8   MOLECULE: HAMP, METHYL-ACCEPTING CHEMOTAXIS PROTEIN I;               1093:  7tac-C  5.1  3.9   78   209    5   MOLECULE: REGULATORY PROTEIN NPR1;                                   1094:  6tms-J  5.1  2.8   65    69    3   MOLECULE: A NOVEL DESIGNED PORE PROTEIN;                             1095:  6el1-J  5.1  2.9   73   351    3   MOLECULE: YAXA;                                                      1096:  6el1-G  5.1  2.9   73   351    3   MOLECULE: YAXA;                                                      1097:  4cjz-C  5.1  3.0   63   104   11   MOLECULE: DIACYLGLYCEROL KINASE;                                     1098:  6el1-F  5.1  2.9   73   351    3   MOLECULE: YAXA;                                                      1099:  8cqr-B  5.1  3.4   62   103    6   MOLECULE: NINJURIN-1;                                                1100:  4rsj-A  5.1  2.7   84   270    2   MOLECULE: CHROMOSOME PARTITION PROTEIN SMC;                          1101:  5gar-U  5.1  4.5   67    79    9   MOLECULE: V-TYPE ATP SYNTHASE ALPHA CHAIN;                           1102:  3zo6-M  5.1  3.2   61    68    5   MOLECULE: ATP SYNTHASE ROTOR RING;                                   1103:  6xky-R  5.1  2.5   82   271    9   MOLECULE: FLAGELLIN;                                                 1104:  5szi-B  5.1  2.2   70   140    7   MOLECULE: RAS-RELATED PROTEIN RAB-8A;                                1105:  6pqv-O  5.1  4.1   69    77    7   MOLECULE: ATP SYNTHASE SUBUNIT DELTA;                                1106:  2r9i-A  5.1  2.0   56    72    9   MOLECULE: PUTATIVE PHAGE CAPSID PROTEIN;                             1107:  4kxr-B  5.1  3.1   75   174    7   MOLECULE: PE25;                                                      1108:  5nvu-A  5.1  4.0   80  3169    0   MOLECULE: DYNEIN MOTOR DOMAIN;                                       1109:  6oqs-O  5.1  3.7   66    77    8   MOLECULE: ATP SYNTHASE SUBUNIT DELTA;                                1110:  6o1q-A  5.1  1.8   63   119    6   MOLECULE: NEPHROCYSTIN-1;                                            1111:  2rbd-A  5.1  2.4   59   159    2   MOLECULE: BH2358 PROTEIN;                                            1112:  8fvt-A  5.1  2.0   61    96    7   MOLECULE: 3HB12;                                                     1113:  8pda-A  5.1  6.4   66   973    3   MOLECULE: ERAD-ASSOCIATED E3 UBIQUITIN-PROTEIN LIGASE DOA10          1114:  8j7v-B  5.1  4.3   61   280    7   MOLECULE: ZINC TRANSPORTER 7;                                        1115:  5ziy-A  5.1  2.8   83   195   14   MOLECULE: FLAGELLAR HOOK-ASSOCIATED PROTEIN 3;                       1116:  6nr8-2  5.1  4.5   81   103    7   MOLECULE: PREFOLDIN SUBUNIT 1;                                       1117:  3d19-A  5.1  3.3   62   264   10   MOLECULE: CONSERVED METALLOPROTEIN;                                  1118:  1skv-C  5.1  2.0   56    58    0   MOLECULE: HYPOTHETICAL 7.5 KDA PROTEIN;                              1119:  6nrd-4  5.1  2.5   76   104    8   MOLECULE: T-COMPLEX PROTEIN 1 SUBUNIT ALPHA;                         1120:  4zwt-D  5.1  5.0   83   128   14   MOLECULE: RECOMBINATION PROTEIN UVSY;                                1121:  5kmg-P  5.1  2.4   66   128    8   MOLECULE: TUBULIN ALPHA-1B CHAIN;                                    1122:  6wnq-I  5.1  4.2   69    77    7   MOLECULE: ATP SYNTHASE SUBUNIT DELTA;                                1123:  1dd5-A  5.1  4.6   75   184    5   MOLECULE: RIBOSOME RECYCLING FACTOR;                                 1124:  7n5e-F  5.1  7.5   74   310    9   MOLECULE: MECHANOSENSITIVE ION CHANNEL FLYCATCHER1;                  1125:  4zws-G  5.1  2.7   68    96   10   MOLECULE: RECOMBINATION PROTEIN UVSY;                                1126:  4zwt-M  5.1  4.7   77   123   16   MOLECULE: RECOMBINATION PROTEIN UVSY;                                1127:  6o5b-G  5.1  9.1   80   250   13   MOLECULE: CALCIUM UNIPORTER PROTEIN, MITOCHONDRIAL;                  1128:  1set-A  5.1  3.2   68   421   16   MOLECULE: SERYL-TRNA SYNTHETASE;                                     1129:  5t4q-R  5.1  3.6   69    75    9   MOLECULE: ATP SYNTHASE SUBUNIT ALPHA;                                1130:  1nnq-A  5.1  2.2   58   170    2   MOLECULE: RUBRERYTHRIN;                                              1131:  6z6p-N  5.1  2.7   82   541   10   MOLECULE: HISTONE DEACETYLASE HDA1;                                  1132:  5sxc-B  5.1  2.6   83   208    8   MOLECULE: PHOSPHATIDYLINOSITOL 4,5-BISPHOSPHATE 3-KINASE CA          1133:  6wno-A  5.1  2.6   70   290    7   MOLECULE: 243244 FAB HEAVY CHAIN;                                    1134:  5maw-D  5.1  2.6   69   260    7   MOLECULE: FLAGELLIN;                                                 1135:  6jy0-H  5.1  3.5   76   410   11   MOLECULE: FLAGELLIN;                                                 1136:  6u28-C  5.1  2.4   72   129    4   MOLECULE: NON-STRUCTURAL PROTEIN 1;                                  1137:  1wyy-A  5.1  2.7   78   126    8   MOLECULE: E2 GLYCOPROTEIN;                                           1138:  8dzz-D  5.1  3.9   79  2363   10   MOLECULE: DYNEIN HEAVY CHAIN, CYTOPLASMIC;                           1139:  6bpe-D  5.1  2.7   71   302    7   MOLECULE: RETICULOCYTE BINDING PROTEIN 2, PUTATIVE;                  1140:  5eg1-A  5.1  3.2   72   571    4   MOLECULE: MICROCIN-J25 EXPORT ATP-BINDING/PERMEASE PROTEIN           1141:  8dcp-B  5.1  2.7   83   276    8   MOLECULE: PHOSPHATIDYLINOSITOL 4,5-BISPHOSPHATE 3-KINASE CA          1142:  8v8v-D  5.1  3.2   82   271    9   MOLECULE: PHOSPHATIDYLINOSITOL 4,5-BISPHOSPHATE 3-KINASE CA          1143:  6z6f-C  5.1  2.7   78   541    6   MOLECULE: HISTONE DEACETYLASE HDA1;                                  1144:  5sxj-B  5.1  2.9   83   216    7   MOLECULE: PHOSPHATIDYLINOSITOL 4,5-BISPHOSPHATE 3-KINASE CA          1145:  5n9j-D  5.0  2.6   63   135    6   MOLECULE: MEDIATOR OF RNA POLYMERASE II TRANSCRIPTION SUBUN          1146:  6s2e-A  5.0  3.2   66  1006    2   MOLECULE: DNA POLYMERASE EPSILON CATALYTIC SUBUNIT A;                1147:  4nqi-D  5.0  4.7   84   232    5   MOLECULE: SH3 DOMAIN-CONTAINING PROTEIN;                             1148:  3rkg-A  5.0  3.4   78   258    8   MOLECULE: MAGNESIUM TRANSPORTER MRS2, MITOCHONDRIAL;                 1149:  8i6s-E  5.0  2.7   70    77    9   MOLECULE: CELL DIVISION PROTEIN FTSX;                                1150:  6tmh-H  5.0  3.2   62    71    8   MOLECULE: INHIBITOR OF F1;                                           1151:  7mi1-A  5.0  3.8   82  2628   12   MOLECULE: CHIMERA PROTEIN OF DYNEIN AND ENDOLYSIN;                   1152:  3zcj-A  5.0  3.9   80   205    4   MOLECULE: CAGL;                                                      1153:  4lws-B  5.0  2.9   73    88   10   MOLECULE: UNCHARACTERIZED PROTEIN;                                   1154:  1cii-A  5.0  2.5   82   602    5   MOLECULE: COLICIN IA;                                                1155:  6zz6-B  5.0  2.9   81   423   11   MOLECULE: STRUCTURAL MAINTENANCE OF CHROMOSOMES PROTEIN 1,S          1156:  8gyz-B  5.0  4.2   73   215   10   MOLECULE: TRANSCRIPTION FACTOR TGA7;                                 1157:  5fj8-O  5.0  3.9   73   539    5   MOLECULE: DNA-DIRECTED RNA POLYMERASE III SUBUNIT RPC1;              1158:  5uhe-D  5.0  4.9   80  1266    9   MOLECULE: DNA-DIRECTED RNA POLYMERASE SUBUNIT ALPHA;                 1159:  6tms-H  5.0  2.4   63    69    5   MOLECULE: A NOVEL DESIGNED PORE PROTEIN;                             1160:  6tms-A  5.0  2.5   63    69    5   MOLECULE: A NOVEL DESIGNED PORE PROTEIN;                             1161:  6tut-O  5.0  4.7   78   551    9   MOLECULE: DNA-DIRECTED RNA POLYMERASE III SUBUNIT RPC1;              1162:  7s5c-J  5.0  1.8   53    59    9   MOLECULE: ENCB;                                                      1163:  5xsj-L  5.0  3.1   62   122    2   MOLECULE: PERIPLASMIC BINDING PROTEIN/LACI TRANSCRIPTIONAL           1164:  8bws-O  5.0  4.6   78   568    8   MOLECULE: DNA-DIRECTED RNA POLYMERASE III SUBUNIT RPC1;              1165:  5n5f-B  5.0  3.0   60    92   12   MOLECULE: ENCAPSULATED FERRITIN;                                     1166:  7qcd-A  5.0  3.4   79  1028    8   MOLECULE: STRUCTURAL MAINTENANCE OF CHROMOSOMES PROTEIN 5;           1167:  5jsb-D  5.0  4.3   69   116   10   MOLECULE: INDUCED MYELOID LEUKEMIA CELL DIFFERENTIATION PRO          1168:  5iwr-A  5.0  5.3   70   596    1   MOLECULE: TRANSIENT RECEPTOR POTENTIAL CATION CHANNEL SUBFA          1169:  2y3g-D  5.0  3.7   69   108    9   MOLECULE: NICKEL AND COBALT RESISTANCE PROTEIN CNRR;                 1170:  5ziy-B  5.0  2.2   77   173    8   MOLECULE: FLAGELLAR HOOK-ASSOCIATED PROTEIN 3;                       1171:  4uy8-Y  5.0  2.2   54    63   15   MOLECULE: 50S RIBOSOMAL PROTEIN L32;                                 1172:  4bne-A  5.0  2.3   70   290    7   MOLECULE: PROTEIN KINASE C AND CASEIN KINASE SUBSTRATE IN N          1173:  5iit-C  5.0  2.1   72   357    6   MOLECULE: VACUOLAR TRANSPORTER CHAPERONE 4,CORE HISTONE MAC          1174:  3tul-D  5.0  2.2   76   122    7   MOLECULE: CELL INVASION PROTEIN SIPB;                                1175:  5dac-A  5.0  2.9   73   432    4   MOLECULE: PUTATIVE UNCHARACTERIZED PROTEIN,PUTATIVE UNCHARA          1176:  6elu-D  5.0  2.2   63   161    6   MOLECULE: SERUM RESISTANCE ASSOCIATED; VSG PROTEIN;                  1177:  2xcs-B  5.0  5.5   71   672   13   MOLECULE: DNA GYRASE SUBUNIT B, DNA GYRASE SUBUNIT A;                1178:  1wlx-A  5.0  4.0   77   129   13   MOLECULE: ALPHA-ACTININ 4;                                           1179:  7ni5-A  5.0  2.4   71  2791   11   MOLECULE: SERINE-PROTEIN KINASE ATM;                                 1180:  1yvl-B  5.0  2.9   82   653   12   MOLECULE: SIGNAL TRANSDUCER AND ACTIVATOR OF TRANSCRIPTION           1181:  6dlc-A  5.0  2.2   60   105   13   MOLECULE: DESIGNED PROTEIN DHD1:234_A;                               1182:  6wlz-G  5.0  2.4   86   213   15   MOLECULE: V-TYPE PROTON ATPASE CATALYTIC SUBUNIT A;                  1183:  5lcy-B  5.0  2.4   57    89    5   MOLECULE: FRMR;                                                      1184:  7o3x-C  5.0  2.7   80   215    9   MOLECULE: PROTEIN SLL0617;                                           1185:  3ehf-B  5.0  2.6   60   104    8   MOLECULE: SENSOR KINASE (YOCF PROTEIN);                              1186:  7jtk-C  5.0  2.8   83   427    6   MOLECULE: FLAGELLAR RADIAL SPOKE PROTEIN 1;                          1187:  4yn0-B  5.0  5.1   77   202    5   MOLECULE: TUMOR NECROSIS FACTOR RECEPTOR SUPERFAMILY MEMBER          1188:  7usc-E  5.0  2.7   74   153    4   MOLECULE: CYTOPLASMIC FMR1-INTERACTING PROTEIN 1;                    1189:  8eja-A  5.0  3.1   64   113    9   MOLECULE: ABAK;                                                      1190:  2dq3-B  5.0  3.6   76   425   12   MOLECULE: SERYL-TRNA SYNTHETASE;                                     1191:  7tao-O  5.0  3.8   61    69    5   MOLECULE: V-TYPE PROTON ATPASE SUBUNIT C';                           1192:  6gpg-A  5.0  4.2   60   651    5   MOLECULE: RNA (5'-R(*CP*GP*AP*CP*GP*CP*UP*AP*GP*CP*GP*UP*CP          1193:  2eul-A  5.0  2.6   63   156    5   MOLECULE: ANTI-CLEAVAGE ANTI-GREA TRANSCRIPTION FACTOR GFH1          1194:  3g6b-B  5.0  2.9   72   212   10   MOLECULE: METHYL-ACCEPTING CHEMOTAXIS PROTEIN;                       1195:  5h6i-A  5.0  2.6   56   215    9   MOLECULE: PROTEIN B;                                                 1196:  6roh-C  5.0  3.1   67   337   10   MOLECULE: PROBABLE PHOSPHOLIPID-TRANSPORTING ATPASE DRS2;            1197:  5y5x-O  5.0  5.3   70    75    9   MOLECULE: V-TYPE ATP SYNTHASE ALPHA CHAIN;                           1198:  6ek4-B  5.0  2.8   82   313    7   MOLECULE: PAXB;                                                      1199:  6y5q-9  5.0  2.3   58   420    9   MOLECULE: PHD FINGER-LIKE DOMAIN-CONTAINING PROTEIN 5A;              1200:  6zbf-C  5.0  3.1   81   285   12   MOLECULE: MEROZOITE SURFACE ANTIGENS;                                1201:  2wcd-C  5.0  3.3   84   285    6   MOLECULE: HEMOLYSIN E, CHROMOSOMAL;                                  1202:  6zbd-C  5.0  3.1   81   285   12   MOLECULE: MEROZOITE SURFACE ANTIGENS;                                1203:  8bri-C  5.0  3.4   81   251    7   MOLECULE: CHEMOTAXIS PROTEIN POMA;                                   1204:  8dt0-A  5.0  2.6   65   140    9   MOLECULE: SCAFFOLDING PROTEIN FUNCTIONAL SITES;                      1205:  6sny-A  5.0  3.9   68   107   15   MOLECULE: SYNTHETIC EPCR BINDING PROTEIN;                            1206:  6rwy-M  5.0  3.0   62    73   15   MOLECULE: INNER ROD PROTEIN;                                         1207:  6t5a-B  5.0  4.4   63    96    5   MOLECULE: TEGUMENT PROTEIN UL51;                                     1208:  4zws-F  5.0  2.5   68    93   10   MOLECULE: RECOMBINATION PROTEIN UVSY;                                1209:  4zwt-J  5.0  5.1   82   135   15   MOLECULE: RECOMBINATION PROTEIN UVSY;                                1210:  8ieq-C  5.0  2.9   61   278    2   MOLECULE: PROBABLE G-PROTEIN COUPLED RECEPTOR 156;                   1211:  8iep-C  5.0  2.9   61   278    2   MOLECULE: PROBABLE G-PROTEIN COUPLED RECEPTOR 156;                   1212:  8olt-L  5.0  2.3   54   606    4   MOLECULE: NADH-UBIQUINONE OXIDOREDUCTASE CHAIN 3;                    1213:  3lay-J  5.0  6.1   69    81   12   MOLECULE: ZINC RESISTANCE-ASSOCIATED PROTEIN;                        1214:  6xf2-A  5.0  3.3   72   118   14   MOLECULE: NESPRIN-1;                                                 1215:  6rwy-P  5.0  3.2   65    73   14   MOLECULE: INNER ROD PROTEIN;                                         1216:  6dmp-B  5.0  2.7   67    82    6   MOLECULE: DESIGNED ORTHOGONAL PROTEIN DHD13_XAAA_A;                  1217:  3vr4-G  5.0  4.5   82   181   10   MOLECULE: V-TYPE SODIUM ATPASE CATALYTIC SUBUNIT A;                  1218:  8v2d-G  5.0  2.5   57   307    9   MOLECULE: O43_129 COMPONENT B;                                       1219:  5vot-G  5.0  3.2   64   169    5   MOLECULE: GLUTAMATE RECEPTOR 2;                                      1220:  7n5e-G  5.0  7.1   75   310    9   MOLECULE: MECHANOSENSITIVE ION CHANNEL FLYCATCHER1;                  1221:  7upp-C  5.0  1.8   52    75    8   MOLECULE: DHT03 PROTEIN A;                                           1222:  6ox7-D  5.0  2.8   77   144   10   MOLECULE: NON-STRUCTURAL PROTEIN 1;                                  1223:  8cvi-O  5.0  2.9   78   268    6   MOLECULE: FLAGELLIN;                                                 1224:  4ovu-B  5.0  2.6   83   268    8   MOLECULE: PHOSPHATIDYLINOSITOL 4,5-BISPHOSPHATE 3-KINASE CA          1225:  4waf-B  5.0  2.6   83   250    8   MOLECULE: PHOSPHATIDYLINOSITOL 4,5-BISPHOSPHATE 3-KINASE CA          1226:  4jps-B  5.0  2.6   83   227    8   MOLECULE: PHOSPHATIDYLINOSITOL 4,5-BISPHOSPHATE 3-KINASE CA          1227:  5swg-B  5.0  2.6   83   255    8   MOLECULE: PHOSPHATIDYLINOSITOL 4,5-BISPHOSPHATE 3-KINASE CA          1228:  6wtv-G  5.0  2.1   67   295    3   MOLECULE: RETICULOCYTE BINDING PROTEIN 2B;                           1229:  4xvn-C  5.0  2.8   64   129    5   MOLECULE: SMALL TERMINASE;                                           1230:  8cvi-G  5.0  5.0   77   268   12   MOLECULE: FLAGELLIN;                                                 1231:  4xvn-A  5.0  2.8   64   130    5   MOLECULE: SMALL TERMINASE;                                           1232:  6jy0-B  5.0  3.5   76   410   11   MOLECULE: FLAGELLIN;                                                 1233:  8cvi-E  5.0  5.6   81   268   10   MOLECULE: FLAGELLIN;                                                 1234:  7rch-C  5.0  2.5   71   129    4   MOLECULE: NON-STRUCTURAL PROTEIN 1;                                  1235:  6jy0-C  5.0  3.5   76   410   11   MOLECULE: FLAGELLIN;                                                 1236:  6xt4-A  4.9  2.2   59   235    7   MOLECULE: 1BH_69;                                                    1237:  7uic-c  4.9  3.2   74   110    5   MOLECULE: MEDIATOR OF RNA POLYMERASE II TRANSCRIPTION SUBUN          1238:  8th8-J  4.9  3.5   70   372    7   MOLECULE: DYNEIN REGULATORY COMPLEX PROTEIN 1/2 N-TERMINAL           1239:  8hr5-A  4.9  3.0   65   466    6   MOLECULE: TRANSPOSASE;                                               1240:  8eki-B  4.9  3.6   66    85    6   MOLECULE: PROTEIN TRANSPORT PROTEIN SEC20;                           1241:  8fmv-D  4.9  3.5   75   665    3   MOLECULE: PROBABLE MULTIDRUG RESISTANCE ABC TRANSPORTER ATP          1242:  1u2m-C  4.9  3.9   69   143   14   MOLECULE: HISTONE-LIKE PROTEIN HLP-1;                                1243:  4p1n-B  4.9  3.4   61   207    2   MOLECULE: ATG1 TMIT;                                                 1244:  6jqa-B  4.9  3.8   74    88    4   MOLECULE: PHYTOPLASMAL EFFECTOR CAUSING PHYLLODY 1;                  1245:  4cgk-A  4.9  4.3   72   351    6   MOLECULE: SECRETED 45 KDA PROTEIN;                                   1246:  1yf2-A  4.9  4.4   75   425    7   MOLECULE: TYPE I RESTRICTION-MODIFICATION ENZYME, S SUBUNIT          1247:  6njp-G  4.9  2.2   59    64    5   MOLECULE: TRANSLOCATOR ESCN;                                         1248:  5ebz-G  4.9  2.5   84   655    7   MOLECULE: INHIBITOR OF NUCLEAR FACTOR KAPPA-B KINASE SUBUNI          1249:  8hcq-R  4.9  5.5   72   303    6   MOLECULE: GUANINE NUCLEOTIDE-BINDING PROTEIN G(Q) SUBUNIT A          1250:  5twv-B  4.9  2.8   79  1375    8   MOLECULE: ATP-SENSITIVE INWARD RECTIFIER POTASSIUM CHANNEL           1251:  7ny0-A  4.9  2.9   61   121    7   MOLECULE: BOSKAR4;                                                   1252:  5jsb-J  4.9  3.9   71   116    8   MOLECULE: INDUCED MYELOID LEUKEMIA CELL DIFFERENTIATION PRO          1253:  5k47-D  4.9  7.4   80   484    4   MOLECULE: POLYCYSTIN-2;                                              1254:  2dq3-A  4.9  3.0   79   425    8   MOLECULE: SERYL-TRNA SYNTHETASE;                                     1255:  7enj-H  4.9  3.1   64   181    5   MOLECULE: MEDIATOR OF RNA POLYMERASE II TRANSCRIPTION SUBUN          1256:  3fxd-B  4.9  2.0   51    58    8   MOLECULE: PROTEIN ICMQ;                                              1257:  6x80-F  4.9  2.9   83   574   11   MOLECULE: FLAGELLIN A;                                               1258:  4n6h-A  4.9  2.8   75   408    8   MOLECULE: SOLUBLE CYTOCHROME B562, DELTA-TYPE OPIOID RECEPT          1259:  5jsb-H  4.9  3.7   70   116    6   MOLECULE: INDUCED MYELOID LEUKEMIA CELL DIFFERENTIATION PRO          1260:  3pla-A  4.9  2.8   75   375    9   MOLECULE: PRE MRNA SPLICING PROTEIN;                                 1261:  5jsb-B  4.9  3.7   70   116    6   MOLECULE: INDUCED MYELOID LEUKEMIA CELL DIFFERENTIATION PRO          1262:  5t4p-Q  4.9  3.8   66    75    8   MOLECULE: ATP SYNTHASE SUBUNIT ALPHA;                                1263:  8tdl-A  4.9  5.3   62   364    5   MOLECULE: MECHANOSENSITIVE ION CHANNEL PROTEIN 10;                   1264:  7y6b-A  4.9  2.6   57    72    2   MOLECULE: ESCE/YSCE/SSAE FAMILY TYPE III SECRETION SYSTEM N          1265:  7khw-R  4.9  3.3   72   174    7   MOLECULE: TRANSLOCON ESPA;                                           1266:  4ayt-A  4.9  2.8   70   565    3   MOLECULE: ATP-BINDING CASSETTE SUB-FAMILY B MEMBER 10 MITOC          1267:  6zbg-A  4.9  2.2   64   562    9   MOLECULE: MEROZOITE SURFACE ANTIGENS;                                1268:  6pza-C  4.9  3.2   66  1422    6   MOLECULE: ATP-BINDING CASSETTE SUB-FAMILY C MEMBER 8;                1269:  5z51-A  4.9  2.9   62   161    3   MOLECULE: DNA PRIMASE;                                               1270:  1a36-A  4.9  3.1   65   544   12   MOLECULE: DNA (5'-                                                   1271:  6res-D  4.9  4.3   67    74    9   MOLECULE: ASA-10: POLYTOMELLA F-ATP SYNTHASE ASSOCIATED SUB          1272:  6nrd-5  4.9  3.2   79   127   13   MOLECULE: T-COMPLEX PROTEIN 1 SUBUNIT ALPHA;                         1273:  3kbu-B  4.9  3.0   76   298    5   MOLECULE: SPECTRIN BETA CHAIN, ERYTHROCYTE;                          1274:  4gwp-A  4.9  2.8   66   112    9   MOLECULE: MEDIATOR OF RNA POLYMERASE II TRANSCRIPTION SUBUN          1275:  5x11-B  4.9  2.1   58   179   10   MOLECULE: DNA (28-MER);                                              1276:  6reu-C  4.9  3.5   58    74    2   MOLECULE: MITOCHONDRIAL ATP SYNTHASE SUBUNIT C;                      1277:  1qu7-B  4.9  2.6   82   221    4   MOLECULE: METHYL-ACCEPTING CHEMOTAXIS PROTEIN I;                     1278:  6nrb-1  4.9  4.5   80   107    9   MOLECULE: T-COMPLEX PROTEIN 1 SUBUNIT ALPHA;                         1279:  6pw5-B  4.9  6.2   75   782    3   MOLECULE: TRP-LIKE ION CHANNEL;                                      1280:  5uaq-I  4.9  3.8   67  1340    7   MOLECULE: DNA-DIRECTED RNA POLYMERASE SUBUNIT ALPHA;                 1281:  1few-A  4.9  3.0   81   173    2   MOLECULE: SECOND MITOCHONDRIA-DERIVED ACTIVATOR OF CASPASES          1282:  5y06-A  4.9  3.4   77   235    6   MOLECULE: MSMEG_4306;                                                1283:  4zwr-C  4.9  5.2   85   137   14   MOLECULE: RECOMBINATION PROTEIN UVSY;                                1284:  8j0h-A  4.9  2.8   64   134    3   MOLECULE: UNCHARACTERIZED PROTEIN C4H3.06;                           1285:  3k29-A  4.9  2.7   81   162    9   MOLECULE: PUTATIVE UNCHARACTERIZED PROTEIN;                          1286:  8f6e-A  4.9  4.0   73   282    5   MOLECULE: CADMIUM AND ZINC EFFLUX PUMP FIEF;                         1287:  2gd5-A  4.9  2.2   74   142    5   MOLECULE: CHARGED MULTIVESICULAR BODY PROTEIN 3;                     1288:  6tut-A  4.9  6.6   65  1390    8   MOLECULE: DNA-DIRECTED RNA POLYMERASE III SUBUNIT RPC1;              1289:  8j0n-D  4.9  2.7   60   156    3   MOLECULE: ER MEMBRANE PROTEIN COMPLEX SUBUNIT 1;                     1290:  7rmy-A  4.9  2.5   60   266    5   MOLECULE: DE NOVO DESIGNED TUNABLE HOMODIMER, D_3-337;               1291:  7wu7-3  4.9  3.2   73   116   10   MOLECULE: PREFOLDIN SUBUNIT 1;                                       1292:  7khw-H  4.9  3.4   73   174    7   MOLECULE: TRANSLOCON ESPA;                                           1293:  3k6c-D  4.9  2.4   60    92    8   MOLECULE: UNCHARACTERIZED PROTEIN NE0167;                            1294:  2v0x-B  4.9  2.7   81   205    9   MOLECULE: LAMINA-ASSOCIATED POLYPEPTIDE 2 ISOFORMS                   1295:  6vbu-2  4.9  3.1   83   659    2   MOLECULE: BARDET-BIEDL SYNDROME 18 PROTEIN;                          1296:  4pl0-A  4.9  4.4   80   571    8   MOLECULE: MICROCIN-J25 EXPORT ATP-BINDING/PERMEASE PROTEIN           1297:  6zsi-C  4.9  2.5   68   130    9   MOLECULE: RAS-RELATED PROTEIN RAB-8A;                                1298:  1f23-A  4.9  2.9   65    74   12   MOLECULE: TRANSMEMBRANE GLYCOPROTEIN;                                1299:  1jq0-A  4.9  2.6   63    71   10   MOLECULE: GP41 ENVELOPE PROTEIN;                                     1300:  4fyg-A  4.9  5.6   72   743    6   MOLECULE: SIDF, INHIBITOR OF GROWTH FAMILY, MEMBER 3;                1301:  5k47-B  4.9  7.1   79   484    6   MOLECULE: POLYCYSTIN-2;                                              1302:  8ddj-B  4.9  3.4   63   280    3   MOLECULE: MECHANOSENSITIVE CHANNEL MSCS;                             1303:  4h63-V  4.9  2.9   72   117    6   MOLECULE: MEDIATOR OF RNA POLYMERASE II TRANSCRIPTION SUBUN          1304:  5u96-A  4.9  1.8   48    50   10   MOLECULE: PUTATIVE INTEGRASE;                                        1305:  2f42-A  4.9  4.0   63   138    5   MOLECULE: STIP1 HOMOLOGY AND U-BOX CONTAINING PROTEIN 1;             1306:  6o84-B  4.9  3.0   63   415   13   MOLECULE: LOC100127796 PROTEIN,LOC100127796 PROTEIN,OTOP3,           1307:  5lnx-D  4.9  2.0   66   373    8   MOLECULE: ACYL-COA DEHYDROGENASE;                                    1308:  6o30-A  4.9  4.4   77   575    6   MOLECULE: LIPID A EXPORT ATP-BINDING/PERMEASE PROTEIN MSBA;          1309:  7jh5-A  4.9  3.1   64   285    8   MOLECULE: CO-LOCKR: DE NOVO DESIGNED PROTEIN SWITCH;                 1310:  5swp-B  4.9  2.6   83   258    8   MOLECULE: PHOSPHATIDYLINOSITOL 4,5-BISPHOSPHATE 3-KINASE CA          1311:  5k7v-B  4.9  2.7   56   279    9   MOLECULE: DESIGNED PROTEIN HR00C3;                                   1312:  5sxd-B  4.9  2.6   82   244    9   MOLECULE: PHOSPHATIDYLINOSITOL 4,5-BISPHOSPHATE 3-KINASE CA          1313:  2rd0-B  4.9  2.9   83   139    8   MOLECULE: PHOSPHATIDYLINOSITOL-4,5-BISPHOSPHATE 3-KINASE CA          1314:  8dd4-B  4.9  3.1   83   274    7   MOLECULE: PHOSPHATIDYLINOSITOL 4,5-BISPHOSPHATE 3-KINASE CA          1315:  7myn-B  4.9  2.6   84   276    7   MOLECULE: PHOSPHATIDYLINOSITOL 4,5-BISPHOSPHATE 3-KINASE CA          1316:  6bpa-A  4.9  2.0   67   297    3   MOLECULE: RETICULOCYTE BINDING PROTEIN 2, PUTATIVE;                  1317:  8cvi-L  4.9  3.6   76   269   13   MOLECULE: FLAGELLIN;                                                 1318:  6f44-O  4.9  4.3   73   534    8   MOLECULE: DNA-DIRECTED RNA POLYMERASE III SUBUNIT RPC1;              1319:  6z6o-K  4.9  2.7   84   548    4   MOLECULE: HISTONE DEACETYLASE HDA1;                                  1320:  5gnt-A  4.9  3.3   66   378    9   MOLECULE: MITOFUSIN-1;                                               1321:  5swr-B  4.9  2.5   82   252    9   MOLECULE: PHOSPHATIDYLINOSITOL 4,5-BISPHOSPHATE 3-KINASE CA          1322:  6r1j-J  4.8  2.4   77   264   13   MOLECULE: UNCHARACTERIZED PROTEIN;                                   1323:  7emf-H  4.8  3.4   64   181    9   MOLECULE: MEDIATOR OF RNA POLYMERASE II TRANSCRIPTION SUBUN          1324:  6bfi-B  4.8  4.8   71   806   11   MOLECULE: VIN1;                                                      1325:  4i0x-F  4.8  2.8   68    85    9   MOLECULE: ESAT-6-LIKE PROTEIN MAB_3112;                              1326:  7tve-E  4.8  3.6   82   461    9   MOLECULE: DNA (68-MER);                                              1327:  7jqe-A  4.8  4.1   76   357    8   MOLECULE: ESAT-6/WXG100 SECRETION SYSTEM PROTEIN;                    1328:  1sc7-A  4.8  2.6   68   567    4   MOLECULE: 5'-D(*AP*AP*AP*AP*AP*GP*AP*CP*TP*T)-3';                    1329:  6btm-C  4.8  4.1   67   457   12   MOLECULE: ALTERNATIVE COMPLEX III SUBUNIT A;                         1330:  6ek4-A  4.8  3.4   79   342    9   MOLECULE: PAXB;                                                      1331:  5xu0-B  4.8  2.6   69   231    6   MOLECULE: MEMBRANE-FUSION PROTEIN;                                   1332:  8b6l-O  4.8  2.2   67   580    7   MOLECULE: PROTEIN TRANSPORT PROTEIN SEC61 SUBUNIT ALPHA ISO          1333:  6q0x-A  4.8  3.1   76   373    5   MOLECULE: SORTING NEXIN MVP1;                                        1334:  2lw1-A  4.8  3.8   72    85   10   MOLECULE: ABC TRANSPORTER ATP-BINDING PROTEIN UUP;                   1335:  3caz-B  4.8  3.2   80   210    6   MOLECULE: BAR PROTEIN;                                               1336:  6lum-G  4.8  6.2   67   123    4   MOLECULE: SUCCINATE DEHYDROGENASE SUBUNIT C;                         1337:  4dci-F  4.8  3.4   79   147   13   MOLECULE: UNCHARACTERIZED PROTEIN;                                   1338:  8kg9-A  4.8  2.2   56   200    5   MOLECULE: DNA REPLICATION LICENSING FACTOR MCM2;                     1339:  3lnm-D  4.8  8.9   80   318    4   MOLECULE: VOLTAGE-GATED POTASSIUM CHANNEL SUBUNIT BETA-2;            1340:  6ch2-E  4.8  3.5   76   147    5   MOLECULE: FLAGELLAR BIOSYNTHESIS PROTEIN FLHA;                       1341:  8fih-A  4.8  2.4   67   105   13   MOLECULE: 3HB05;                                                     1342:  7uw5-F  4.8  2.4   63   723    3   MOLECULE: MECHANOSENSITIVE CHANNEL MSCK;                             1343:  4oyd-B  4.8  3.6   71   117    6   MOLECULE: APOPTOSIS REGULATOR BHRF1;                                 1344:  3a8p-A  4.8  2.2   65   232    6   MOLECULE: T-LYMPHOMA INVASION AND METASTASIS-INDUCING                1345:  2c2a-A  4.8  3.9   71   240    7   MOLECULE: SENSOR HISTIDINE KINASE;                                   1346:  7uw5-C  4.8  2.4   63   723    3   MOLECULE: MECHANOSENSITIVE CHANNEL MSCK;                             1347:  3ehf-C  4.8  2.0   52    57   10   MOLECULE: SENSOR KINASE (YOCF PROTEIN);                              1348:  5dac-B  4.8  3.0   75   432    5   MOLECULE: PUTATIVE UNCHARACTERIZED PROTEIN,PUTATIVE UNCHARA          1349:  7sn7-A  4.8  2.8   85   546    4   MOLECULE: FLAGELLIN;                                                 1350:  1zw0-A  4.8  2.3   56    65    7   MOLECULE: TYPE III SECRETION PROTEIN;                                1351:  5gar-X  4.8  4.0   68    79    7   MOLECULE: V-TYPE ATP SYNTHASE ALPHA CHAIN;                           1352:  6hk5-H  4.8  1.8   53    60    4   MOLECULE: COOJ;                                                      1353:  6tj1-A  4.8  2.8   61    71    5   MOLECULE: DE NOVO DESIGNED WSHC6;                                    1354:  4zwq-A  4.8  5.3   86   136   14   MOLECULE: RECOMBINATION PROTEIN UVSY;                                1355:  7nkh-G  4.8  3.0   65    89    9   MOLECULE: ATP SYNTHASE SUBUNIT ALPHA;                                1356:  5fl7-Q  4.8  3.9   67    76    3   MOLECULE: ATP SYNTHASE SUBUNIT ALPHA;                                1357:  7asm-W  4.8  2.1   55    66    4   MOLECULE: 50S RIBOSOMAL PROTEIN L19;                                 1358:  6d80-H  4.8  6.3   68    80    0   MOLECULE: SAPOSIN A;                                                 1359:  8a1g-A  4.8  2.0   82   218   15   MOLECULE: SORTING NEXIN-1;                                           1360:  6dma-C  4.8  2.4   65    73   11   MOLECULE: DHD15_CLOSED_A;                                            1361:  6nd1-E  4.8  2.6   62   139    8   MOLECULE: PROTEIN TRANSPORT PROTEIN SEC61;                           1362:  7khw-a  4.8  3.9   78   174    5   MOLECULE: TRANSLOCON ESPA;                                           1363:  5yti-A  4.8  2.2   82   330    5   MOLECULE: FLAGELLAR HOOK ASSOCIATED PROTEIN TYPE 3 FLGL;             1364:  3kbt-B  4.8  2.9   74   294    5   MOLECULE: SPECTRIN BETA CHAIN, ERYTHROCYTE;                          1365:  1ivs-A  4.8  2.9   63   863   10   MOLECULE: TRNA (VAL);                                                1366:  1cun-A  4.8  2.1   63   213   14   MOLECULE: PROTEIN (ALPHA SPECTRIN);                                  1367:  6vme-K  4.8  2.2   57    65    4   MOLECULE: TUMOR SUSCEPTIBILITY GENE 101 PROTEIN;                     1368:  7x8v-A  4.8  2.7   75   185    7   MOLECULE: OS01G0156300 PROTEIN;                                      1369:  7dms-A  4.8  2.3   51    94   12   MOLECULE: FE(II)-BINDING EFFECTOR;                                   1370:  3gnl-A  4.8  3.2   53   234    9   MOLECULE: UNCHARACTERIZED PROTEIN, DUF633, LMOF2365_1472;            1371:  6d6v-A  4.8  2.7   58   980    5   MOLECULE: TELOMERASE REVERSE TRANSCRIPTASE;                          1372:  7khw-t  4.8  3.4   73   174    7   MOLECULE: TRANSLOCON ESPA;                                           1373:  5ijh-A  4.8  3.1   82   181    5   MOLECULE: XENOTROPIC AND POLYTROPIC RETROVIRUS RECEPTOR 1;           1374:  3abu-A  4.8  2.7   66   644   11   MOLECULE: LYSINE-SPECIFIC HISTONE DEMETHYLASE 1;                     1375:  2z4e-D  4.8  2.0   52    54   10   MOLECULE: FIBRINOGEN ALPHA CHAIN;                                    1376:  1wr6-A  4.8  1.8   56    90   16   MOLECULE: ADP-RIBOSYLATION FACTOR BINDING PROTEIN GGA3;              1377:  5mkk-A  4.8  4.2   82   593    5   MOLECULE: MULTIDRUG RESISTANCE ABC TRANSPORTER ATP-BINDING           1378:  5n9j-Z  4.8  3.0   67   119    4   MOLECULE: MEDIATOR OF RNA POLYMERASE II TRANSCRIPTION SUBUN          1379:  7ag9-B  4.8  3.5   84   384    7   MOLECULE: KAR9;                                                      1380:  8rhn-A  4.8  4.2   80   198    9   MOLECULE: ATPASE FAMILY GENE 2 PROTEIN HOMOLOG A;                    1381:  2wcd-P  4.8  3.2   84   285    6   MOLECULE: HEMOLYSIN E, CHROMOSOMAL;                                  1382:  7f3t-B  4.8  4.1   58   330    9   MOLECULE: TRANSMEMBRANE PROTEIN 120A;                                1383:  6d80-G  4.8  7.3   71    80    0   MOLECULE: SAPOSIN A;                                                 1384:  6cno-D  4.8  2.3   72   360    4   MOLECULE: INTERMEDIATE CONDUCTANCE CALCIUM-ACTIVATED POTASS          1385:  6z0c-A  4.8  2.8   80   199   15   MOLECULE: MAQUETTE-3;                                                1386:  1t7s-A  4.8  2.9   68   129    9   MOLECULE: BAG-1 COCHAPERONE;                                         1387:  1tqq-A  4.8  3.6   62   428    8   MOLECULE: OUTER MEMBRANE PROTEIN TOLC;                               1388:  4rh7-A  4.8  2.7   85  3005   14   MOLECULE: GREEN FLUORESCENT PROTEIN/CYTOPLASMIC DYNEIN 2 HE          1389:  2oer-A  4.8  3.2   59   179    5   MOLECULE: PROBABLE TRANSCRIPTIONAL REGULATOR;                        1390:  8ieq-D  4.8  2.9   62   279    2   MOLECULE: PROBABLE G-PROTEIN COUPLED RECEPTOR 156;                   1391:  1i4d-A  4.8  3.8   79   188   10   MOLECULE: ARFAPTIN 2;                                                1392:  8iep-D  4.8  2.9   62   279    2   MOLECULE: PROBABLE G-PROTEIN COUPLED RECEPTOR 156;                   1393:  2z4y-B  4.8  2.8   62   282    6   MOLECULE: GERANYLGERANYL PYROPHOSPHATE SYNTHETASE;                   1394:  6ah0-w  4.8  2.9   62   443   13   MOLECULE: U5SNRNA;                                                   1395:  5tpt-B  4.8  4.7   70   181   10   MOLECULE: AMYLOID-LIKE PROTEIN 2;                                    1396:  3qf4-B  4.8  4.2   79   588    8   MOLECULE: ABC TRANSPORTER, ATP-BINDING PROTEIN;                      1397:  6b2z-4  4.8  3.7   66    75    6   MOLECULE: ATP SYNTHASE SUBUNIT C, MITOCHONDRIAL;                     1398:  6n3q-E  4.8  3.4   68   135   12   MOLECULE: PROTEIN TRANSPORT PROTEIN SEC61;                           1399:  7nvr-c  4.8  1.9   60   109    7   MOLECULE: TFIIH BASAL TRANSCRIPTION FACTOR COMPLEX HELICASE          1400:  8v2d-A  4.8  2.7   57   307    9   MOLECULE: O43_129 COMPONENT B;                                       1401:  2axt-Z  4.8  2.7   58    62    2   MOLECULE: PHOTOSYSTEM Q(B) PROTEIN;                                  1402:  5lm2-B  4.8  3.8   82   340    7   MOLECULE: TYROSINE-PROTEIN PHOSPHATASE NON-RECEPTOR TYPE 23          1403:  2vv5-A  4.8  4.3   61   256    7   MOLECULE: SMALL-CONDUCTANCE MECHANOSENSITIVE CHANNEL;                1404:  8ddj-C  4.8  3.7   64   280    3   MOLECULE: MECHANOSENSITIVE CHANNEL MSCS;                             1405:  6cnm-C  4.8  2.9   75   359    9   MOLECULE: INTERMEDIATE CONDUCTANCE CALCIUM-ACTIVATED POTASS          1406:  3nvo-A  4.8  2.8   71   250    7   MOLECULE: ZINC TRANSPORT PROTEIN ZNTB;                               1407:  5eiu-D  4.8  2.5   68   132   13   MOLECULE: TRIM PROTEIN-E3 LIGASE CHIMERA;                            1408:  6lqo-J  4.8  4.8   72    93    8   MOLECULE: CYTOPLASMIC ENVELOPMENT PROTEIN 1;                         1409:  5aww-G  4.8  4.7   63    75   10   MOLECULE: PROTEIN TRANSLOCASE SUBUNIT SECY;                          1410:  6lqo-K  4.8  4.9   70    81    9   MOLECULE: CYTOPLASMIC ENVELOPMENT PROTEIN 1;                         1411:  8ddj-E  4.8  3.6   64   280    3   MOLECULE: MECHANOSENSITIVE CHANNEL MSCS;                             1412:  6a2j-A  4.8  2.9   62   309    6   MOLECULE: HEME A SYNTHASE;                                           1413:  6xjx-C  4.8  8.6   81   151   14   MOLECULE: CALCIUM UNIPORTER PROTEIN, MITOCHONDRIAL;                  1414:  8fdu-A  4.8  2.7   67  1001    9   MOLECULE: CYTOPLASMIC DYNEIN 1 HEAVY CHAIN 1;                        1415:  8cvi-X  4.8  5.0   77   268   12   MOLECULE: FLAGELLIN;                                                 1416:  7zr1-C  4.8  3.6   82   779    2   MOLECULE: DOUBLE-STRAND BREAK REPAIR PROTEIN;                        1417:  6jy0-U  4.8  3.9   73   410   10   MOLECULE: FLAGELLIN;                                                 1418:  7rch-D  4.8  3.1   81   144    4   MOLECULE: NON-STRUCTURAL PROTEIN 1;                                  1419:  8cye-A  4.8  3.5   70   269    7   MOLECULE: FLAGELLIN;                                                 1420:  6f41-O  4.8  4.3   73   534    8   MOLECULE: DNA-DIRECTED RNA POLYMERASE III SUBUNIT RPC1;              1421:  2v1y-B  4.8  3.2   83   170    8   MOLECULE: PHOSPHATIDYLINOSITOL-4,5-BISPHOSPHATE 3-KINASE CA          1422:  5swo-B  4.8  2.6   83   267    8   MOLECULE: PHOSPHATIDYLINOSITOL 4,5-BISPHOSPHATE 3-KINASE CA          1423:  6jfl-B  4.8  3.3   67   413    9   MOLECULE: MITOFUSIN-2,CDNA FLJ57997, HIGHLY SIMILAR TO TRAN          1424:  6jy0-K  4.8  3.9   73   410   10   MOLECULE: FLAGELLIN;                                                 1425:  5go4-A  4.8  2.0   71   414    3   MOLECULE: MITOFUSIN-1;                                               1426:  6jy0-N  4.8  3.9   73   410   10   MOLECULE: FLAGELLIN;                                                 1427:  8cye-T  4.8  2.8   85   269    4   MOLECULE: FLAGELLIN;                                                 1428:  5ukv-A  4.7  4.2   65    74    3   MOLECULE: ATP-BINDING PROTEIN;                                       1429:  6upn-A  4.7  3.5   72   245    8   MOLECULE: ENDOPHILIN-B1;                                             1430:  8th8-H  4.7  2.1   65   820   11   MOLECULE: DYNEIN REGULATORY COMPLEX PROTEIN 1/2 N-TERMINAL           1431:  7kzn-B  4.7  3.0   61   683   16   MOLECULE: HEAVY CHAIN ALPHA;                                         1432:  8ipr-C  4.7  2.8   70   515   11   MOLECULE: COMPONENT LINKED WITH THE ASSEMBLY OF CYTOCHROME'          1433:  6m3q-F  4.7  3.1   78   301    3   MOLECULE: ANKYRIN-2;                                                 1434:  7xad-C  4.7  3.1   65    96   14   MOLECULE: PROGRAMMED CELL DEATH 1 LIGAND 1;                          1435:  4i0x-K  4.7  3.3   68    77    9   MOLECULE: ESAT-6-LIKE PROTEIN MAB_3112;                              1436:  6xkw-n  4.7  3.0   72   471   10   MOLECULE: CYTOCHROME C OXIDASE, CBB3-TYPE, SUBUNIT I;                1437:  3hai-A  4.7  2.6   78   294    9   MOLECULE: HUMAN PACSIN1 F-BAR;                                       1438:  5ijj-B  4.7  3.6   78   157    8   MOLECULE: SPX DOMAIN;                                                1439:  3edv-A  4.7  3.0   79   322    8   MOLECULE: SPECTRIN BETA CHAIN, BRAIN 1;                              1440:  6qum-N  4.7  3.6   78   649   10   MOLECULE: V-TYPE ATP SYNTHASE ALPHA CHAIN;                           1441:  2qup-A  4.7  3.9   59   119    7   MOLECULE: BH1478 PROTEIN;                                            1442:  5xfs-A  4.7  2.7   69    78   13   MOLECULE: PE FAMILY PROTEIN PE8;                                     1443:  5vj4-A  4.7  3.1   60   275    3   MOLECULE: UNCHARACTERIZED PROTEIN;                                   1444:  7pgg-A  4.7  4.2   68   147    4   MOLECULE: ION TRANSPORT PROTEIN;                                     1445:  5l4k-V  4.7  5.8   82   293    2   MOLECULE: 26S PROTEASOME NON-ATPASE REGULATORY SUBUNIT 4;            1446:  6ln2-A  4.7  3.3   66   437    5   MOLECULE: GLUCAGON-LIKE PEPTIDE 1 RECEPTOR,RUBREDOXIN,GLUCA          1447:  4okv-F  4.7  3.9   61    66    5   MOLECULE: HEAVY CHAIN OF 8H7 MAB;                                    1448:  7xdi-A  4.7  4.1   62    69    5   MOLECULE: VP1;                                                       1449:  4k0d-B  4.7  3.5   76   145    4   MOLECULE: PERIPLASMIC SENSOR HYBRID HISTIDINE KINASE;                1450:  4gcz-B  4.7  2.7   63   378    5   MOLECULE: BLUE-LIGHT PHOTORECEPTOR, SENSOR PROTEIN FIXL;             1451:  4f4c-A  4.7  2.4   65  1250    3   MOLECULE: MULTIDRUG RESISTANCE PROTEIN PGP-1;                        1452:  1i49-A  4.7  4.1   81   201    4   MOLECULE: ARFAPTIN 2;                                                1453:  5ebz-F  4.7  2.5   84   655    7   MOLECULE: INHIBITOR OF NUCLEAR FACTOR KAPPA-B KINASE SUBUNI          1454:  5jeq-A  4.7  2.9   83   227    7   MOLECULE: NITRATE/NITRITE SENSOR PROTEIN NARQ;                       1455:  5n5e-F  4.7  2.1   57    97   11   MOLECULE: PFC_05175;                                                 1456:  5n5e-T  4.7  2.1   57    97   11   MOLECULE: PFC_05175;                                                 1457:  6zbe-C  4.7  2.5   70   285    9   MOLECULE: MEROZOITE SURFACE ANTIGENS;                                1458:  2ymb-D  4.7  2.4   52   231   13   MOLECULE: MIT DOMAIN-CONTAINING PROTEIN 1;                           1459:  7uw5-G  4.7  2.4   64   723    5   MOLECULE: MECHANOSENSITIVE CHANNEL MSCK;                             1460:  3lhp-S  4.7  4.8   71   116    6   MOLECULE: FV 4E10 HEAVY CHAIN;                                       1461:  8q7n-L  4.7  4.0   72   373   10   MOLECULE: U5 SNRNA;                                                  1462:  1y69-8  4.7  4.8   69   113    4   MOLECULE: 23S RIBOSOMAL RNA;                                         1463:  4h5y-A  4.7  3.7   59   528    7   MOLECULE: LIDA PROTEIN, SUBSTRATE OF THE DOT/ICM SYSTEM;             1464:  5f5p-A  4.7  3.3   81   181   10   MOLECULE: PROTEIN SHROOM2;                                           1465:  7usx-A  4.7  4.1   66   605    6   MOLECULE: TRANSMEMBRANE CHANNEL-LIKE PROTEIN 1;                      1466:  6xr2-D  4.7  1.6   53   167   15   MOLECULE: DTOR_3X57R;                                                1467:  6x80-O  4.7  2.8   84   574    5   MOLECULE: FLAGELLIN A;                                               1468:  6wlw-0  4.7  3.1   71   204    1   MOLECULE: V-TYPE PROTON ATPASE 21 KDA PROTEOLIPID SUBUNIT;           1469:  7die-C  4.7  2.1   60   164    7   MOLECULE: FERRITIN;                                                  1470:  6vyl-C  4.7  4.6   60   256    2   MOLECULE: MECHANOSENSITIVE CHANNEL MSCS;                             1471:  5yq7-M  4.7  4.8   72   305    4   MOLECULE: BETA SUBUNIT OF LIGHT-HARVESTING 1;                        1472:  3edu-A  4.7  2.5   68   191    7   MOLECULE: SPECTRIN BETA CHAIN, ERYTHROCYTE;                          1473:  4zh2-C  4.7  5.0   74  1340    3   MOLECULE: DNA-DIRECTED RNA POLYMERASE SUBUNIT ALPHA;                 1474:  6uz8-A  4.7  5.8   71   734    3   MOLECULE: SHORT TRANSIENT RECEPTOR POTENTIAL CHANNEL 6;              1475:  5hkx-A  4.7  4.4   63   374    3   MOLECULE: E3 UBIQUITIN-PROTEIN LIGASE CBL;                           1476:  6nr9-3  4.7  4.5   80   132    3   MOLECULE: T-COMPLEX PROTEIN 1 SUBUNIT ALPHA;                         1477:  5vox-b  4.7  3.3   77   627    8   MOLECULE: V-TYPE PROTON ATPASE CATALYTIC SUBUNIT A,V-TYPE P          1478:  2a01-A  4.7  5.2   80   243    4   MOLECULE: APOLIPOPROTEIN A-I;                                        1479:  8ctj-A  4.7  5.0   74   401    5   MOLECULE: TRANSMEMBRANE PROTEIN 87A;                                 1480:  5t4p-O  4.7  3.1   64    75    6   MOLECULE: ATP SYNTHASE SUBUNIT ALPHA;                                1481:  8ddj-A  4.7  3.4   63   280    3   MOLECULE: MECHANOSENSITIVE CHANNEL MSCS;                             1482:  1ez3-A  4.7  2.2   67   124    7   MOLECULE: SYNTAXIN-1A;                                               1483:  5d80-G  4.7  2.4   85   223    8   MOLECULE: V-TYPE PROTON ATPASE CATALYTIC SUBUNIT A;                  1484:  8ec3-A  4.7  4.0   70   155    6   MOLECULE: FIBRONECTIN-BINDING PROTEIN;                               1485:  6wc4-B  4.7  2.2   55    87    0   MOLECULE: PROTEIN TRANSPORT PROTEIN SEC39;                           1486:  6m97-A  4.7  2.7   84   208    6   MOLECULE: CHIMERA PROTEIN OF HIGH AFFINITY COPPER UPTAKE PR          1487:  6g7o-A  4.7  3.2   53   350   11   MOLECULE: ALKALINE CERAMIDASE 3,SOLUBLE CYTOCHROME B562;             1488:  8esv-B  4.7  7.1   78   255    5   MOLECULE: DISINTEGRIN AND METALLOPROTEINASE DOMAIN-CONTAINI          1489:  6khi-D  4.7  4.1   56   504    4   MOLECULE: NAD(P)H-QUINONE OXIDOREDUCTASE SUBUNIT 1;                  1490:  6h4j-A  4.7  3.1   84   488    8   MOLECULE: UBIQUITIN CARBOXYL-TERMINAL HYDROLASE 25;                  1491:  2q83-A  4.7  3.6   55   332    4   MOLECULE: YTAA PROTEIN;                                              1492:  6tej-B  4.7  4.2   83   585    7   MOLECULE: DRUG ABC TRANSPORTER ATP-BINDING PROTEIN;                  1493:  6vm0-A  4.7  4.6   74   363    8   MOLECULE: GLYCINE RECEPTOR SUBUNIT ALPHAZ1;                          1494:  7tmw-R  4.7  3.4   74   499    8   MOLECULE: RELAXIN RECEPTOR 1, GUANINE NUCLEOTIDE-BINDING PR          1495:  2wcd-D  4.7  2.5   85   285   11   MOLECULE: HEMOLYSIN E, CHROMOSOMAL;                                  1496:  1eh1-A  4.7  5.3   74   185    8   MOLECULE: RIBOSOME RECYCLING FACTOR;                                 1497:  4j2c-A  4.7  2.1   61   108    8   MOLECULE: SYNTAXIN-6;                                                1498:  7uuw-G  4.7  1.7   60   159    7   MOLECULE: ACTIN, ALPHA SKELETAL MUSCLE;                              1499:  5aji-C  4.7  4.5   62   255    8   MOLECULE: SMALL-CONDUCTANCE MECHANOSENSITIVE CHANNEL;                1500:  7uuw-H  4.7  1.7   60   159    7   MOLECULE: ACTIN, ALPHA SKELETAL MUSCLE;                              1501:  7uuw-Z  4.7  1.7   60   159    7   MOLECULE: ACTIN, ALPHA SKELETAL MUSCLE;                              1502:  7uuw-I  4.7  1.7   60   159    7   MOLECULE: ACTIN, ALPHA SKELETAL MUSCLE;                              1503:  7uuw-K  4.7  1.7   60   159    7   MOLECULE: ACTIN, ALPHA SKELETAL MUSCLE;                              1504:  6b2z-M  4.7  3.5   75   249    5   MOLECULE: ATP SYNTHASE SUBUNIT C, MITOCHONDRIAL;                     1505:  2y3g-A  4.7  3.5   69   109    4   MOLECULE: NICKEL AND COBALT RESISTANCE PROTEIN CNRR;                 1506:  8v2d-P  4.7  2.7   57   307    9   MOLECULE: O43_129 COMPONENT B;                                       1507:  3vbb-A  4.7  3.7   78   451    8   MOLECULE: SERYL-TRNA SYNTHETASE, CYTOPLASMIC;                        1508:  3ogi-C  4.7  3.0   52    56   12   MOLECULE: PUTATIVE ESAT-6-LIKE PROTEIN 6;                            1509:  6hk5-E  4.7  2.6   56    64    2   MOLECULE: COOJ;                                                      1510:  4x5u-A  4.7  3.1   78   205    3   MOLECULE: CAG PATHOGENICITY ISLAND PROTEIN (CAG18);                  1511:  6nr9-5  4.7  2.9   81   127   14   MOLECULE: T-COMPLEX PROTEIN 1 SUBUNIT ALPHA;                         1512:  7aqo-C  4.7  2.2   59   227    8   MOLECULE: THO COMPLEX SUBUNIT 2;                                     1513:  8ils-B  4.7  2.7   83   276    8   MOLECULE: PHOSPHATIDYLINOSITOL 4,5-BISPHOSPHATE 3-KINASE CA          1514:  8v8u-B  4.7  2.8   83   279    8   MOLECULE: PHOSPHATIDYLINOSITOL 4,5-BISPHOSPHATE 3-KINASE CA          1515:  4ovv-B  4.7  2.7   83   241    8   MOLECULE: PHOSPHATIDYLINOSITOL 4,5-BISPHOSPHATE 3-KINASE CA          1516:  7cbc-B  4.7  2.9   65   315    8   MOLECULE: DE NOVO DESIGNED SWITCH PROTEIN CAGING A HEMAGGLU          1517:  8am0-B  4.7  2.8   82   255    9   MOLECULE: PHOSPHATIDYLINOSITOL 4,5-BISPHOSPHATE 3-KINASE CA          1518:  7pg5-B  4.7  3.0   83   266    8   MOLECULE: PHOSPHATIDYLINOSITOL 4,5-BISPHOSPHATE 3-KINASE CA          1519:  6jy0-R  4.7  3.8   73   410    8   MOLECULE: FLAGELLIN;                                                 1520:  6ejq-E  4.7  4.2   69   103    7   MOLECULE: TERMINASE SMALL SUBUNIT;                                   1521:  6eu3-O  4.7  4.3   73   537    7   MOLECULE: DNA-DIRECTED RNA POLYMERASE III SUBUNIT RPC1;              1522:  8cye-B  4.7  3.5   70   269    4   MOLECULE: FLAGELLIN;                                                 1523:  6woz-A  4.7  2.0   67   296    3   MOLECULE: RETICULOCYTE BINDING PROTEIN 2B;                           1524:  6eo1-A  4.6  7.4   75   355   11   MOLECULE: CYCLIC NUCLEOTIDE-GATED POTASSIUM CHANNEL MLL3241          1525:  6xf1-A  4.6  3.1   77   230    9   MOLECULE: NESPRIN-2;                                                 1526:  8gji-A  4.6  2.2   62   167    8   MOLECULE: GCG BINDER;                                                1527:  3aq0-C  4.6  3.2   58   327    2   MOLECULE: GERANYL DIPHOSPHATE SYNTHASE;                              1528:  6dcf-D  4.6  5.2   79  1177    8   MOLECULE: RNA POLYMERASE-BINDING PROTEIN RBPA;                       1529:  7xc2-A  4.6  3.3   58   858   16   MOLECULE: CNL9;                                                      1530:  4m0m-A  4.6  3.7   63   721    6   MOLECULE: PUTATIVE UNCHARACTERIZED PROTEIN;                          1531:  8eas-A  4.6  3.8   75   130    8   MOLECULE: VACUOLAR ATPASE ASSEMBLY PROTEIN VMA22;                    1532:  8auc-B  4.6  2.7   84   493    4   MOLECULE: CELL WALL-ASSOCIATED HYDROLASES (INVASION-ASSOCIA          1533:  6lo8-D  4.6  4.8   62   119   10   MOLECULE: MITOCHONDRIAL IMPORT INNER MEMBRANE TRANSLOCASE S          1534:  6zls-A  4.6  2.9   71   292   10   MOLECULE: HISTIDINE KINASE;                                          1535:  1izl-B  4.6  3.9   72   378    6   MOLECULE: PHOTOSYSTEM II: SUBUNIT PSBA;                              1536:  5yke-B  4.6  3.3   81   845    5   MOLECULE: ATP-SENSITIVE INWARD RECTIFIER POTASSIUM CHANNEL           1537:  7mi6-A  4.6  2.8   54  2419    6   MOLECULE: FUSION PROTEIN OF DYNEIN AND ENDOLYSIN;                    1538:  6uo8-B  4.6  2.9   63   696    5   MOLECULE: GAMMA-AMINOBUTYRIC ACID TYPE B RECEPTOR SUBUNIT 1          1539:  8otx-A  4.6  2.7   66  1054    9   MOLECULE: SPERM-SPECIFIC SODIUM PROTON EXCHANGER;                    1540:  6ahx-A  4.6  3.0   52    60    0   MOLECULE: PUTATIVE CYTOSOLIC PROTEIN;                                1541:  1zw0-D  4.6  2.1   55    66   15   MOLECULE: TYPE III SECRETION PROTEIN;                                1542:  8eg3-A  4.6  3.9   62   554    3   MOLECULE: STERYL-SULFATASE;                                          1543:  6hgc-A  4.6  3.1   72   304   10   MOLECULE: UBIQUITIN CARBOXYL-TERMINAL HYDROLASE CALYPSO,UBI          1544:  4drf-A  4.6  3.4   69   408   13   MOLECULE: METALLOPHOSPHOESTERASE;                                    1545:  5eke-C  4.6  3.1   72   307    6   MOLECULE: UNCHARACTERIZED GLYCOSYLTRANSFERASE SLL0501;               1546:  7eew-A  4.6  3.4   75   611    4   MOLECULE: TYPE I RESTRICTION-MODIFICATION SYSTEM METHYLTRAN          1547:  5fl7-M  4.6  3.8   66    75    3   MOLECULE: ATP SYNTHASE SUBUNIT ALPHA;                                1548:  6h9x-A  4.6  4.3   75   428    9   MOLECULE: SERINE--TRNA LIGASE;                                       1549:  5ebz-K  4.6  2.4   84   655    7   MOLECULE: INHIBITOR OF NUCLEAR FACTOR KAPPA-B KINASE SUBUNI          1550:  6tye-D  4.6  5.1   79  1262    8   MOLECULE: RNA POLYMERASE SIGMA FACTOR;                               1551:  2x2v-F  4.6  2.9   58    69    2   MOLECULE: ATP SYNTHASE SUBUNIT C;                                    1552:  7ag9-A  4.6  3.0   68   375    4   MOLECULE: KAR9;                                                      1553:  6elu-A  4.6  2.2   69   193    4   MOLECULE: SERUM RESISTANCE ASSOCIATED; VSG PROTEIN;                  1554:  4ijj-B  4.6  3.0   74   130    8   MOLECULE: PUTATIVE C4-TYPE ZINC FINGER PROTEIN, DKSA/TRAR F          1555:  2y3g-B  4.6  3.8   70   110    9   MOLECULE: NICKEL AND COBALT RESISTANCE PROTEIN CNRR;                 1556:  2y3g-C  4.6  3.5   69   109    9   MOLECULE: NICKEL AND COBALT RESISTANCE PROTEIN CNRR;                 1557:  1eiy-A  4.6  3.3   71   345    8   MOLECULE: TRNA(PHE);                                                 1558:  2y3d-A  4.6  4.9   74   111    8   MOLECULE: NICKEL AND COBALT RESISTANCE PROTEIN CNRR;                 1559:  2y3h-D  4.6  3.6   70   107    7   MOLECULE: NICKEL AND COBALT RESISTANCE PROTEIN CNRR;                 1560:  5tj5-H  4.6  4.3   68   150    7   MOLECULE: V-TYPE PROTON ATPASE SUBUNIT A;                            1561:  2y3h-B  4.6  3.8   70   109    7   MOLECULE: NICKEL AND COBALT RESISTANCE PROTEIN CNRR;                 1562:  3ehf-D  4.6  3.1   63   181   11   MOLECULE: SENSOR KINASE (YOCF PROTEIN);                              1563:  2y3h-C  4.6  3.5   69   108    7   MOLECULE: NICKEL AND COBALT RESISTANCE PROTEIN CNRR;                 1564:  3epv-A  4.6  3.5   69   109    9   MOLECULE: NICKEL AND COBALT RESISTANCE PROTEIN CNRR;                 1565:  6vyl-G  4.6  4.6   61   256    2   MOLECULE: MECHANOSENSITIVE CHANNEL MSCS;                             1566:  6vyl-F  4.6  4.6   61   256    2   MOLECULE: MECHANOSENSITIVE CHANNEL MSCS;                             1567:  3zq6-D  4.6  2.9   65   307    8   MOLECULE: PUTATIVE ARSENICAL PUMP-DRIVING ATPASE;                    1568:  7zkp-6  4.6  2.2   49   184    8   MOLECULE: SUBUNIT NIMM OF NADH:UBIQUINONE OXIDOREDUCTASE (C          1569:  1n73-A  4.6  3.2   65    91    5   MOLECULE: FIBRIN ALPHA-1 CHAIN;                                      1570:  4di0-B  4.6  2.2   58   140    3   MOLECULE: RUBRERYTHRIN;                                              1571:  3ze4-C  4.6  3.4   64    92   13   MOLECULE: DIACYLGLYCEROL KINASE;                                     1572:  6vyl-D  4.6  4.6   61   256    2   MOLECULE: MECHANOSENSITIVE CHANNEL MSCS;                             1573:  7rkx-R  4.6  3.2   64   275   11   MOLECULE: GUANINE NUCLEOTIDE-BINDING PROTEIN G(I) SUBUNIT A          1574:  5k3j-B  4.6  2.5   59   659    7   MOLECULE: ACYL-COENZYME A OXIDASE;                                   1575:  6vyl-B  4.6  4.6   61   256    2   MOLECULE: MECHANOSENSITIVE CHANNEL MSCS;                             1576:  8eav-F  4.6  3.7   74   159    0   MOLECULE: YAR027W OR YAR028W;                                        1577:  4hks-A  4.6  3.0   68   165    7   MOLECULE: CALCIUM RELEASE-ACTIVATED CALCIUM CHANNEL PROTEIN          1578:  7wkk-A  4.6  1.8   63  1684    8   MOLECULE: MGC83295 PROTEIN;                                          1579:  8jzr-B  4.6  4.6   72   456    7   MOLECULE: LYSOSOMAL TRANSPORTER,ALFA TAG;                            1580:  8cws-B  4.6  1.8   49    55   10   MOLECULE: F4132-2 CHAIN A;                                           1581:  6prk-A  4.6  9.5   83   115    6   MOLECULE: RICF;                                                      1582:  7d6d-A  4.6  5.8   83   380    7   MOLECULE: SORTING NEXIN-1;                                           1583:  3e1i-A  4.6  2.4   55    57   15   MOLECULE: FIBRINOGEN ALPHA CHAIN;                                    1584:  1rkc-A  4.6  2.8   68   258    7   MOLECULE: VINCULIN;                                                  1585:  2p22-A  4.6 48.9   63   168   14   MOLECULE: SUPPRESSOR PROTEIN STP22 OF TEMPERATURE-SENSITIVE          1586:  6ixv-B  4.6  2.5   82   226    7   MOLECULE: SH3 DOMAIN-BINDING PROTEIN 5;                              1587:  6v7v-A  4.6  2.7   56    62   13   MOLECULE: QUORUM SENSING ANTI-ACTIVATOR AQS1;                        1588:  7s5c-G  4.6  2.4   54    62    7   MOLECULE: ENCB;                                                      1589:  6foc-G  4.6  2.2   84   193    8   MOLECULE: ATP SYNTHASE SUBUNIT ALPHA,ATP SYNTHASE SUBUNIT A          1590:  5t58-A  4.6  5.9   73   233    4   MOLECULE: KLLA0F02343P;                                              1591:  7c7s-A  4.6  5.0   61   685    8   MOLECULE: GAMMA-AMINOBUTYRIC ACID TYPE B RECEPTOR SUBUNIT 1          1592:  3plt-A  4.6  5.0   86   214    8   MOLECULE: SPHINGOLIPID LONG CHAIN BASE-RESPONSIVE PROTEIN L          1593:  7emf-0  4.6  2.4   60   267    5   MOLECULE: MEDIATOR OF RNA POLYMERASE II TRANSCRIPTION SUBUN          1594:  4gzr-C  4.6  3.5   56    61    2   MOLECULE: ESAT-6-LIKE PROTEIN 6;                                     1595:  6zbc-C  4.6  5.4   81   285    4   MOLECULE: MEROZOITE SURFACE ANTIGENS;                                1596:  8q0e-B  4.6  3.1   77   241   13   MOLECULE: VARIANT SURFACE GLYCOPROTEIN 545;                          1597:  5xlr-B  4.6  3.4   64  1022    6   MOLECULE: SPIKE GLYCOPROTEIN;                                        1598:  4rkm-B  4.6  2.8   53   660    6   MOLECULE: MCCA;                                                      1599:  8sql-A  4.6  5.3   81  1396   14   MOLECULE: METAL RESISTANCE PROTEIN YCF1;                             1600:  1s5l-Z  4.6  2.4   55    58    5   MOLECULE: PHOTOSYSTEM Q(B) PROTEIN;                                  1601:  6nrc-6  4.6  3.4   77   102    9   MOLECULE: PREFOLDIN SUBUNIT 1;                                       1602:  8ij9-C  4.6  2.4   62    71    5   MOLECULE: RAS-RELATED PROTEIN RAB-6B;                                1603:  5aji-F  4.6  4.5   62   256    8   MOLECULE: SMALL-CONDUCTANCE MECHANOSENSITIVE CHANNEL;                1604:  3l4q-C  4.6  3.2   84   163   11   MOLECULE: NON-STRUCTURAL PROTEIN 1;                                  1605:  4k2p-A  4.6  2.4   67   238    7   MOLECULE: T-LYMPHOMA INVASION AND METASTASIS-INDUCING PROTE          1606:  3ibp-A  4.6  3.3   78   283    9   MOLECULE: CHROMOSOME PARTITION PROTEIN MUKB;                         1607:  6rwy-U  4.6  3.6   63    73   14   MOLECULE: INNER ROD PROTEIN;                                         1608:  5jsn-B  4.6  5.7   75   115    4   MOLECULE: APOPTOSIS REGULATOR BCL-2;                                 1609:  3auy-A  4.6  3.2   67   366    9   MOLECULE: DNA DOUBLE-STRAND BREAK REPAIR RAD50 ATPASE;               1610:  6z16-f  4.6  2.7   51    88    2   MOLECULE: MULTISUBUNIT NA+/H+ ANTIPORTER, A SUBUNIT;                 1611:  7o3x-D  4.6  2.9   81   215    9   MOLECULE: PROTEIN SLL0617;                                           1612:  1set-B  4.6  3.4   71   421    6   MOLECULE: SERYL-TRNA SYNTHETASE;                                     1613:  6k7x-A  4.6  8.3   80   273   13   MOLECULE: CALCIUM UNIPORTER PROTEIN, MITOCHONDRIAL;                  1614:  5xu0-A  4.6  3.0   72   227    7   MOLECULE: MEMBRANE-FUSION PROTEIN;                                   1615:  6uuj-K  4.6  2.3   58   163   10   MOLECULE: PE FAMILY IMMUNOMODULATOR PE5;                             1616:  6sih-B  4.6  3.1   83   488   10   MOLECULE: FLAGELLAR HOOK-ASSOCIATED PROTEIN 2;                       1617:  8ipr-D  4.6  3.8   55   504    5   MOLECULE: COMPONENT LINKED WITH THE ASSEMBLY OF CYTOCHROME'          1618:  7mgm-A  4.6  3.8   70  2274   11   MOLECULE: DYNEIN AAA3-WALKERB MUTANT (E2488Q);                       1619:  8cye-D  4.6  3.8   79   269   11   MOLECULE: FLAGELLIN;                                                 1620:  8ilr-B  4.6  2.8   83   276    8   MOLECULE: PHOSPHATIDYLINOSITOL 4,5-BISPHOSPHATE 3-KINASE CA          1621:  6jy0-Q  4.6  3.9   72   410   10   MOLECULE: FLAGELLIN;                                                 1622:  8cye-S  4.6  5.2   79   269   10   MOLECULE: FLAGELLIN;                                                 1623:  6jy0-S  4.6  3.9   73   410   10   MOLECULE: FLAGELLIN;                                                 1624:  6jy0-F  4.6  3.9   73   410   10   MOLECULE: FLAGELLIN;                                                 1625:  7sn7-b  4.6  3.3   73   546    5   MOLECULE: FLAGELLIN;                                                 1626:  8cye-F  4.6  3.9   79   269   10   MOLECULE: FLAGELLIN;                                                 1627:  5j0l-C  4.6  4.9   70   118   16   MOLECULE: DESIGNED PROTEIN 3L6HC2_2;                                 1628:  6jy0-P  4.6  3.9   73   410   10   MOLECULE: FLAGELLIN;                                                 1629:  6bpc-A  4.6  2.1   67   296    3   MOLECULE: RETICULOCYTE BINDING PROTEIN 2, PUTATIVE;                  1630:  8cye-C  4.6  4.0   78   269   10   MOLECULE: FLAGELLIN;                                                 1631:  6bpc-D  4.6  4.4   78   286    6   MOLECULE: RETICULOCYTE BINDING PROTEIN 2, PUTATIVE;                  1632:  6vfh-B  4.6  4.3   57   277    9   MOLECULE: T33_DN10A;                                                 1633:  8cye-P  4.6  3.7   72   269    6   MOLECULE: FLAGELLIN;                                                 1634:  7sn7-N  4.6  3.6   71   546    7   MOLECULE: FLAGELLIN;                                                 1635:  6ox7-C  4.6  2.7   71   129   10   MOLECULE: NON-STRUCTURAL PROTEIN 1;                                  1636:  5n9y-A  4.5  4.1   76   327    8   MOLECULE: ZINC TRANSPORT PROTEIN ZNTB;                               1637:  2ajq-A  4.5  8.0   70   704    6   MOLECULE: DNA PRIMER;                                                1638:  4ykn-A  4.5  2.4   72  1204    3   MOLECULE: PHOSPHATIDYLINOSITOL 3-KINASE REGULATORY SUBUNIT           1639:  5odw-D  4.5  2.5   70   198   11   MOLECULE: FERRIPYOVERDINE RECEPTOR;                                  1640:  4ad8-A  4.5  4.2   77   452    8   MOLECULE: DNA REPAIR PROTEIN RECN;                                   1641:  8odu-C  4.5  4.7   65   232    2   MOLECULE: ATPASE GET3;                                               1642:  8ik3-A  4.5  3.3   61   328   11   MOLECULE: STIMULATOR OF INTERFERON GENES PROTEIN,IMMUNE PRO          1643:  6uuj-G  4.5  3.1   63    74   11   MOLECULE: PE FAMILY IMMUNOMODULATOR PE5;                             1644:  7d3e-A  4.5  5.0   79  1380    6   MOLECULE: DUAL OXIDASE 1;                                            1645:  8ay2-A  4.5  3.4   74   400    5   MOLECULE: EXOCYST COMPLEX COMPONENT 4;                               1646:  7wb4-E  4.5  4.6   80  1363    8   MOLECULE: OUTER NUP133;                                              1647:  2yev-C  4.5  2.0   52    64    6   MOLECULE: CYTOCHROME C OXIDASE POLYPEPTIDE I+III;                    1648:  2fb5-A  4.5  3.6   66   204    5   MOLECULE: HYPOTHETICAL MEMBRANE SPANNING PROTEIN;                    1649:  4b6x-A  4.5  2.7   60    69    3   MOLECULE: AVIRULENCE PROTEIN;                                        1650:  5jdo-A  4.5  4.1   74   249    8   MOLECULE: HAPTOGLOBIN-HAEMOGLOBIN RECEPTOR;                          1651:  4tn3-B  4.5  3.8   78   362    8   MOLECULE: TRIM5/CYCLOPHILIN A FUSION PROTEIN/T4 LYSOZYME CH          1652:  7upp-A  4.5  2.7   57   312    5   MOLECULE: DHT03 PROTEIN A;                                           1653:  7oqz-A  4.5  2.9   61   237    3   MOLECULE: TRANSMEMBRANE PROTEIN 45A;                                 1654:  7bin-g  4.5  2.9   64   260   17   MOLECULE: FLAGELLAR BIOSYNTHETIC PROTEIN FLIP;                       1655:  8xej-X  4.5  2.8   66   358    8   MOLECULE: ISOFORM 2 OF BASIGIN;                                      1656:  1g8x-A  4.5  3.0   74  1009    5   MOLECULE: MYOSIN II HEAVY CHAIN FUSED TO ALPHA-ACTININ 3;            1657:  3ter-B  4.5  4.4   77   121    6   MOLECULE: MAMMALIAN STROMAL INTERACTION MOLECULE-1;                  1658:  7v2w-J  4.5  3.1   71   236    8   MOLECULE: THO COMPLEX SUBUNIT HPR1;                                  1659:  6ewy-A  4.5  2.7   82   201    5   MOLECULE: PEPTIDOGLYCAN ENDOPEPTIDASE RIPA;                          1660:  5lbm-C  4.5  2.7   59    90    7   MOLECULE: TRANSCRIPTIONAL REPRESSOR FRMR;                            1661:  8gmh-D  4.5  3.7   68    78    7   MOLECULE: LXG DOMAIN-CONTAINING PROTEIN;                             1662:  7pgi-D  4.5  4.6   74   147    5   MOLECULE: ION TRANSPORT PROTEIN;                                     1663:  6jfm-A  4.5  2.8   81   419    9   MOLECULE: MITOFUSIN-2,MITOFUSIN-2;                                   1664:  4iwb-A  4.5  2.7   57   165    5   MOLECULE: FLIC, FLIS CHIMERA;                                        1665:  2y3h-A  4.5  4.4   71   110    7   MOLECULE: NICKEL AND COBALT RESISTANCE PROTEIN CNRR;                 1666:  2fji-1  4.5  3.2   67   399    7   MOLECULE: EXOCYST COMPLEX COMPONENT SEC6;                            1667:  6b2z-a  4.5  3.6   71   249    6   MOLECULE: ATP SYNTHASE SUBUNIT C, MITOCHONDRIAL;                     1668:  7ytj-C  4.5  3.1   62   102   10   MOLECULE: VACUOLAR TRANSPORTER CHAPERONE 4;                          1669:  1s94-B  4.5  2.0   65   118    8   MOLECULE: S-SYNTAXIN;                                                1670:  7jg5-a  4.5  3.9   68   207    4   MOLECULE: ATP SYNTHASE SUBUNIT ALPHA;                                1671:  7by1-A  4.5  2.6   50   287   10   MOLECULE: HISTONE ACETYLTRANSFERASE KAT2A;                           1672:  2xy5-A  4.5  5.6   70   581    7   MOLECULE: DNA POLYMERASE I;                                          1673:  6xky-C  4.5  2.4   82   271    7   MOLECULE: FLAGELLIN;                                                 1674:  6xky-K  4.5  2.4   82   271    7   MOLECULE: FLAGELLIN;                                                 1675:  6xky-E  4.5  2.4   82   271    7   MOLECULE: FLAGELLIN;                                                 1676:  6xky-J  4.5  2.4   82   271    7   MOLECULE: FLAGELLIN;                                                 1677:  6xky-D  4.5  2.4   82   271    7   MOLECULE: FLAGELLIN;                                                 1678:  6xky-I  4.5  2.4   82   271    7   MOLECULE: FLAGELLIN;                                                 1679:  6xky-T  4.5  2.4   82   271    7   MOLECULE: FLAGELLIN;                                                 1680:  5l0g-C  4.5  3.1   70   171    4   MOLECULE: VINCULIN;                                                  1681:  6xky-M  4.5  2.4   82   271    7   MOLECULE: FLAGELLIN;                                                 1682:  6xky-G  4.5  2.4   82   271    7   MOLECULE: FLAGELLIN;                                                 1683:  6xky-S  4.5  2.4   82   271    7   MOLECULE: FLAGELLIN;                                                 1684:  6xky-H  4.5  2.4   82   271    7   MOLECULE: FLAGELLIN;                                                 1685:  6xky-O  4.5  2.4   82   271    7   MOLECULE: FLAGELLIN;                                                 1686:  6xky-F  4.5  2.4   82   271    7   MOLECULE: FLAGELLIN;                                                 1687:  6xky-L  4.5  2.4   85   271    7   MOLECULE: FLAGELLIN;                                                 1688:  5lqy-N  4.5  3.3   62    72    8   MOLECULE: ATP SYNTHASE SUBUNIT F;                                    1689:  2d4x-A  4.5  2.9   84   214    5   MOLECULE: FLAGELLAR HOOK-ASSOCIATED PROTEIN 3;                       1690:  5fwp-E  4.5  2.7   76   259    9   MOLECULE: HEAT SHOCK PROTEIN HSP 90 BETA;                            1691:  8jwd-A  4.5  2.9   61   123   11   MOLECULE: HISTIDINE KINASE;                                          1692:  8q5h-A  4.5  5.7   71   205    8   MOLECULE: KINETOCHORE PROTEIN SPC24;                                 1693:  3r84-J  4.5  3.1   65    79    5   MOLECULE: MEDIATOR OF RNA POLYMERASE II TRANSCRIPTION SUBUN          1694:  6irb-B  4.5  5.3   80   207    3   MOLECULE: 1-PHOSPHATIDYLINOSITOL 4,5-BISPHOSPHATE PHOSPHODI          1695:  8fih-C  4.5  2.7   63   105    6   MOLECULE: 3HB05;                                                     1696:  1u4q-A  4.5  2.8   75   318    4   MOLECULE: SPECTRIN ALPHA CHAIN, BRAIN;                               1697:  4p1n-A  4.5  3.3   73   190    7   MOLECULE: ATG1 TMIT;                                                 1698:  6ux2-A  4.5  2.1   60   619   17   MOLECULE: SIGNAL TRANSDUCER AND ACTIVATOR OF TRANSCRIPTION           1699:  7yzp-C  4.5  4.1   81   448    6   MOLECULE: DNA HAIRPIN (59-MER);                                      1700:  6sl1-A  4.5  3.4   76  2651    8   MOLECULE: SERINE/THREONINE-PROTEIN KINASE TEL1;                      1701:  1upg-A  4.5  4.1   66    90   11   MOLECULE: TRANSCRIPTIONAL REPRESSOR TRAM;                            1702:  8adl-A  4.5  2.2   53   633    6   MOLECULE: MTC5 ISOFORM 1;                                            1703:  1i4t-B  4.5  4.4   82   179    9   MOLECULE: ARFAPTIN 2;                                                1704:  3stq-A  4.5  2.4   55    86    2   MOLECULE: PUTATIVE UNCHARACTERIZED PROTEIN;                          1705:  6rlb-A  4.5  2.8   65   969    9   MOLECULE: O6-ALKYLGUANINE-DNA ALKYLTRANSFERASE MUTANT,DYNC2          1706:  6v6c-L  4.5  2.6   52   497   10   MOLECULE: GAMMA-TUBULIN COMPLEX COMPONENT 6;                         1707:  6kac-B  4.5  5.5   77   490    3   MOLECULE: PHOTOSYSTEM II PROTEIN D1;                                 1708:  5uh9-D  4.5  5.1   78  1265    6   MOLECULE: DNA-DIRECTED RNA POLYMERASE SUBUNIT ALPHA;                 1709:  1b3q-B  4.5  2.6   59   370    5   MOLECULE: PROTEIN (CHEMOTAXIS PROTEIN CHEA);                         1710:  2g38-B  4.5  2.7   73   173    8   MOLECULE: PE FAMILY PROTEIN;                                         1711:  5vlr-B  4.5  3.0   82   170    6   MOLECULE: PHOSPHATIDYLINOSITOL 4,5-BISPHOSPHATE 3-KINASE CA          1712:  5xbj-A  4.5  3.2   85   500   11   MOLECULE: FLAGELLAR HOOK-ASSOCIATED PROTEIN FLGK;                    1713:  2yfb-B  4.5  3.4   80   238    8   MOLECULE: METHYL-ACCEPTING CHEMOTAXIS TRANSDUCER;                    1714:  8c5v-L  4.5  1.9   78   516   10   MOLECULE: CHEMOTAXIS PROTEIN CHEA;                                   1715:  8cqr-A  4.5  3.0   62   103   10   MOLECULE: NINJURIN-1;                                                1716:  2a79-B  4.5  8.8   77   259    6   MOLECULE: VOLTAGE-GATED POTASSIUM CHANNEL BETA-2 SUBUNIT;            1717:  2pih-A  4.5  9.0   78   123    5   MOLECULE: PROTEIN YMCA;                                              1718:  3qne-A  4.5  2.6   74   441    7   MOLECULE: SERYL-TRNA SYNTHETASE, CYTOPLASMIC;                        1719:  4avm-A  4.5  3.1   86   230    5   MOLECULE: BRIDGING INTEGRATOR 2;                                     1720:  3l4q-D  4.5  3.0   82   163   10   MOLECULE: NON-STRUCTURAL PROTEIN 1;                                  1721:  3b60-A  4.5  5.2   82   572   11   MOLECULE: LIPID A EXPORT ATP-BINDING/PERMEASE PROTEIN MSBA;          1722:  1y2o-A  4.5  2.5   85   248    7   MOLECULE: BAI1-ASSOCIATED PROTEIN 2 ISOFORM 1;                       1723:  6h2f-A  4.5  2.6   68   331    7   MOLECULE: AHLB;                                                      1724:  7jh6-A  4.5  3.4   77   174   12   MOLECULE: TWO-DOMAIN DI-ZN(II) AND PORPHYRIN-BINDING PROTEI          1725:  4m70-H  4.5  2.5   62   111   10   MOLECULE: RX PROTEIN;                                                1726:  6z16-F  4.5  2.7   51    88    2   MOLECULE: MULTISUBUNIT NA+/H+ ANTIPORTER, A SUBUNIT;                 1727:  8soi-B  4.5  2.7   83   515   12   MOLECULE: RB1-INDUCIBLE COILED-COIL PROTEIN 1;                       1728:  2jj1-G  4.5  2.6   79   167    5   MOLECULE: ATP SYNTHASE SUBUNIT ALPHA HEART ISOFORM;                  1729:  5ik2-G  4.5  2.9   84   285    7   MOLECULE: ATP SYNTHASE SUBUNIT ALPHA;                                1730:  5ip0-G  4.5  3.4   62    90    6   MOLECULE: PHA GRANULE-ASSOCIATED PROTEIN;                            1731:  8tsb-B  4.5  3.1   83   270    8   MOLECULE: PHOSPHATIDYLINOSITOL 4,5-BISPHOSPHATE 3-KINASE CA          1732:  8cye-U  4.5  3.5   70   269    4   MOLECULE: FLAGELLIN;                                                 1733:  6cnc-O  4.5  3.6   66   539    6   MOLECULE: DNA-DIRECTED RNA POLYMERASE III SUBUNIT RPC1;              1734:  8igq-E  4.5  2.6   81   126    7   MOLECULE: PROBABLE ENDOPEPTIDASE MT2245;                             1735:  8cvi-g  4.5  3.7   70   269    7   MOLECULE: FLAGELLIN;                                                 1736:  6wty-J  4.5  2.5   70   289    7   MOLECULE: RETICULOCYTE BINDING PROTEIN 2B;                           1737:  6x80-I  4.5  3.6   69   574    9   MOLECULE: FLAGELLIN A;                                               1738:  5yew-B  4.5  4.1   67   387    6   MOLECULE: MITOFUSIN-1,MITOFUSIN-1 FUSION PROTEIN;                    1739:  8cvi-Y  4.5  2.8   67   268    6   MOLECULE: FLAGELLIN;                                                 1740:  8dcx-B  4.5  3.3   82   236    9   MOLECULE: PHOSPHATIDYLINOSITOL 4,5-BISPHOSPHATE 3-KINASE CA          1741:  4cti-D  4.4  2.7   65   222    9   MOLECULE: OSMOLARITY SENSOR PROTEIN ENVZ, AF1503;                    1742:  2c9l-Y  4.4  3.5   58    63    5   MOLECULE: BZLF1 TRANS-ACTIVATOR PROTEIN;                             1743:  3cqx-D  4.4  3.3   63    84   13   MOLECULE: HEAT SHOCK COGNATE 71 KDA PROTEIN;                         1744:  1wa8-A  4.4  3.2   69    99   10   MOLECULE: ESAT-6 LIKE PROTEIN ESXB;                                  1745:  1urf-A  4.4  3.0   63    81    6   MOLECULE: PROTEIN KINASE C-LIKE 1;                                   1746:  4gd3-A  4.4  3.3   59   179    3   MOLECULE: HYDROGENASE-1 SMALL CHAIN;                                 1747:  3owa-C  4.4  3.4   63   587   11   MOLECULE: ACYL-COA DEHYDROGENASE;                                    1748:  6tdv-D  4.4  5.0   71   186   11   MOLECULE: ATPTB1;                                                    1749:  5nx9-D  4.4  3.5   65   477   14   MOLECULE: ADENYLOSUCCINATE LYASE;                                    1750:  6tpi-A  4.4  2.6   71   380    6   MOLECULE: MUREIN HYDROLASE ACTIVATOR ENVC;                           1751:  7bou-A  4.4  6.7   75   525    7   MOLECULE: PORTAL PROTEIN;                                            1752:  8tsh-F  4.4  2.9   69   265   14   MOLECULE: ABC TRANSPORTER ATP-BINDING PROTEIN;                       1753:  6gej-M  4.4  3.6   64   688   14   MOLECULE: VACUOLAR PROTEIN SORTING-ASSOCIATED PROTEIN 72;            1754:  5n9j-G  4.4  1.8   51   163    4   MOLECULE: MEDIATOR OF RNA POLYMERASE II TRANSCRIPTION SUBUN          1755:  8ih5-A  4.4  3.2   60   689    8   MOLECULE: SYN-COPALYL DIPHOSPHATE SYNTHASE, CHLOROPLASTIC;           1756:  7wiu-B  4.4  5.2   75   576    8   MOLECULE: MYCOBACTIN IMPORT ATP-BINDING/PERMEASE PROTEIN IR          1757:  4hwi-B  4.4  3.5   68   177    4   MOLECULE: HEAT SHOCK COGNATE 71 KDA PROTEIN;                         1758:  6dlu-P  4.4  5.2   72   747    7   MOLECULE: DYNAMIN-1;                                                 1759:  7uq2-A  4.4  4.8   69    89    1   MOLECULE: VS.4;                                                      1760:  8c8g-A  4.4  4.7   78  1335    9   MOLECULE: PUTATIVE BOTULINUM-LIKE TOXIN WO;                          1761:  7eu9-A  4.4  3.5   65  1078    5   MOLECULE: CAS12I1 D647A MUTANT;                                      1762:  7awt-D  4.4  4.7   64   589    6   MOLECULE: NADH-QUINONE OXIDOREDUCTASE SUBUNIT B;                     1763:  7qj2-G  4.4  5.3   61   640    5   MOLECULE: GAMMA-TUBULIN COMPLEX COMPONENT 5;                         1764:  6ye4-A  4.4  4.1   79   236    8   MOLECULE: BIOPOLYMER TRANSPORT PROTEIN EXBB;                         1765:  5vyk-C  4.4  3.2   59   203    5   MOLECULE: CHIMERA PROTEIN OF BRS DOMAIN OF BRAF AND CC-SAM           1766:  3lut-B  4.4  9.0   80   390    6   MOLECULE: VOLTAGE-GATED POTASSIUM CHANNEL SUBUNIT BETA-2;            1767:  6xky-A  4.4  2.4   82   271    5   MOLECULE: FLAGELLIN;                                                 1768:  6uz2-A  4.4  3.9   82   570    9   MOLECULE: LIPID A EXPORT ATP-BINDING/PERMEASE PROTEIN MSBA;          1769:  3vr5-G  4.4  6.4   76   164    9   MOLECULE: V-TYPE SODIUM ATPASE CATALYTIC SUBUNIT A;                  1770:  6bbf-A  4.4  2.3   57   150    7   MOLECULE: CALCIUM RELEASE-ACTIVATED CALCIUM CHANNEL PROTEIN          1771:  6y92-B  4.4  3.6   78   178   10   MOLECULE: B-LYMPHOCYTE ANTIGEN CD20;                                 1772:  6jfm-B  4.4  2.7   81   422    9   MOLECULE: MITOFUSIN-2,MITOFUSIN-2;                                   1773:  7pmk-A  4.4  2.9   63   197   13   MOLECULE: DNA REPLICATION LICENSING FACTOR MCM2;                     1774:  2y44-A  4.4  1.9   71   184    6   MOLECULE: GLUTAMIC ACID/ALANINE-RICH PROTEIN;                        1775:  7pgi-B  4.4  4.4   73   146    7   MOLECULE: ION TRANSPORT PROTEIN;                                     1776:  7oia-Y  4.4  2.9   60   176    5   MOLECULE: 39S RIBOSOMAL PROTEIN L2, MITOCHONDRIAL;                   1777:  4jle-B  4.4  6.6   72   159    8   MOLECULE: PHIST;                                                     1778:  6bbf-C  4.4  2.3   57   150    7   MOLECULE: CALCIUM RELEASE-ACTIVATED CALCIUM CHANNEL PROTEIN          1779:  4g33-A  4.4  4.3   70   661   10   MOLECULE: 15S-LIPOXYGENASE;                                          1780:  5t4q-O  4.4  3.8   67    75    6   MOLECULE: ATP SYNTHASE SUBUNIT ALPHA;                                1781:  5dny-B  4.4  1.9   60   359    5   MOLECULE: DNA DOUBLE-STRAND BREAK REPAIR PROTEIN MRE11;              1782:  8th8-C  4.4  4.4   76   533    4   MOLECULE: DYNEIN REGULATORY COMPLEX PROTEIN 1/2 N-TERMINAL           1783:  7qh7-Y  4.4  2.9   60   175    5   MOLECULE: 39S RIBOSOMAL PROTEIN L2, MITOCHONDRIAL;                   1784:  5t4q-N  4.4  3.2   64    75    6   MOLECULE: ATP SYNTHASE SUBUNIT ALPHA;                                1785:  6xl0-B  4.4  2.3   81   271    9   MOLECULE: FLAGELLIN;                                                 1786:  2pyb-A  4.4  2.7   59   151    7   MOLECULE: NEUTROPHIL ACTIVATING PROTEIN;                             1787:  6xl0-M  4.4  2.3   81   271    9   MOLECULE: FLAGELLIN;                                                 1788:  6xl0-T  4.4  2.3   81   271    9   MOLECULE: FLAGELLIN;                                                 1789:  7sqj-B  4.4  2.9   85   546    5   MOLECULE: FLAGELLIN;                                                 1790:  6xl0-H  4.4  2.3   81   271    9   MOLECULE: FLAGELLIN;                                                 1791:  6xky-P  4.4  2.5   82   271    9   MOLECULE: FLAGELLIN;                                                 1792:  6xl0-P  4.4  2.3   81   271    9   MOLECULE: FLAGELLIN;                                                 1793:  7ylm-A  4.4  3.3   81   428    5   MOLECULE: STRUCTURAL MAINTENANCE OF CHROMOSOMES PROTEIN 5;           1794:  6xl0-I  4.4  2.3   81   271    9   MOLECULE: FLAGELLIN;                                                 1795:  8srm-C  4.4  5.1   73   205   10   MOLECULE: RB1-INDUCIBLE COILED-COIL PROTEIN 1;                       1796:  3i2w-A  4.4  5.7   80   270    8   MOLECULE: SYNDAPIN;                                                  1797:  6xl0-Q  4.4  2.3   81   271    9   MOLECULE: FLAGELLIN;                                                 1798:  5yqz-R  4.4  2.8   70   558    9   MOLECULE: GLUCAGON RECEPTOR,ENDOLYSIN,GLUCAGON RECEPTOR;             1799:  6z6e-C  4.4  3.6   62   101    6   MOLECULE: TERMINASE SMALL SUBUNIT;                                   1800:  4dx0-A  4.4  2.8   62   229   13   MOLECULE: 14-3-3-LIKE PROTEIN E;                                     1801:  3hkz-A  4.4 24.2   64   836    8   MOLECULE: DNA-DIRECTED RNA POLYMERASE SUBUNIT A';                    1802:  4e40-A  4.4  3.2   82   245   10   MOLECULE: PUTATIVE UNCHARACTERIZED PROTEIN;                          1803:  4up6-C  4.4  3.9   65    92   11   MOLECULE: DIACYLGLYCEROL KINASE;                                     1804:  7khw-A  4.4  3.3   72   174    7   MOLECULE: TRANSLOCON ESPA;                                           1805:  8ear-A  4.4  8.6   76  2389   12   MOLECULE: INOSITOL 1,4,5-TRISPHOSPHATE RECEPTOR TYPE 1;              1806:  5tj5-G  4.4  4.6   69   149    9   MOLECULE: V-TYPE PROTON ATPASE SUBUNIT A;                            1807:  7ux1-A  4.4  4.5   65   713   12   MOLECULE: MECHANOSENSITIVE CHANNEL MSCK;                             1808:  1hci-A  4.4  2.9   76   475    5   MOLECULE: ALPHA-ACTININ 2;                                           1809:  7nna-A  4.4  2.5   83   194   11   MOLECULE: KLEBICIN C ACTIVITY;                                       1810:  7yoj-A  4.4  2.5   61   867    7   MOLECULE: CASPI;                                                     1811:  1naf-A  4.4  1.9   54   124   17   MOLECULE: ADP-RIBOSYLATION FACTOR BINDING PROTEIN GGA1;              1812:  5j0l-D  4.4  4.3   72   119    7   MOLECULE: DESIGNED PROTEIN 3L6HC2_2;                                 1813:  8cbj-K  4.4  3.5   56    99    9   MOLECULE: 40S RIBOSOMAL PROTEIN S27-A;                               1814:  7s5c-H  4.4  2.4   54    63    7   MOLECULE: ENCB;                                                      1815:  1waf-A  4.4  3.0   77   903    4   MOLECULE: DNA POLYMERASE;                                            1816:  6dg6-E  4.4  1.6   53   100    9   MOLECULE: NEOLEUKIN-2/15;                                            1817:  3s4w-A  4.4  2.4   56  1206   11   MOLECULE: FANCONI ANEMIA GROUP I PROTEIN HOMOLOG;                    1818:  8p3q-H  4.4  2.1   63   177    5   MOLECULE: GLUTAMATE RECEPTOR 2;                                      1819:  7khw-k  4.4  3.4   73   174    7   MOLECULE: TRANSLOCON ESPA;                                           1820:  5ofo-A  4.4  2.8   64   686    6   MOLECULE: CHAPERONE PROTEIN CLPB,ATP-DEPENDENT CLP PROTEASE          1821:  5ijh-B  4.4  3.6   84   184    8   MOLECULE: XENOTROPIC AND POLYTROPIC RETROVIRUS RECEPTOR 1;           1822:  6zsh-A  4.4  3.8   69    92    7   MOLECULE: EH DOMAIN-BINDING PROTEIN 1;                               1823:  8hd0-E  4.4  3.2   81   380    6   MOLECULE: CELL DIVISION ATP-BINDING PROTEIN FTSE;                    1824:  6y92-A  4.4  3.7   79   178    3   MOLECULE: B-LYMPHOCYTE ANTIGEN CD20;                                 1825:  8cih-A  4.4  2.8   77   188   12   MOLECULE: CYCLIN-DEPENDENT KINASE 2-INTERACTING PROTEIN;             1826:  8sq0-A  4.4  5.2   81  1402   14   MOLECULE: METAL RESISTANCE PROTEIN YCF1;                             1827:  4brr-C  4.4  4.1   63    90   10   MOLECULE: DIACYLGLYCEROL KINASE;                                     1828:  3txs-B  4.4  2.5   66    90    3   MOLECULE: TERMINASE DNA PACKAGING ENZYME SMALL SUBUNIT;              1829:  2k48-A  4.4  2.1   68   107    1   MOLECULE: NUCLEOPROTEIN;                                             1830:  2wcd-F  4.4  3.1   80   285   11   MOLECULE: HEMOLYSIN E, CHROMOSOMAL;                                  1831:  1jm0-A  4.4  1.8   46    48    2   MOLECULE: PROTEIN (FOUR-HELIX BUNDLE MODEL);                         1832:  6wjc-A  4.4  2.6   61   445    7   MOLECULE: MUSCARINIC ACETYLCHOLINE RECEPTOR M1,ENDOLYSIN FU          1833:  8c5v-S  4.4  2.2   83   516    4   MOLECULE: CHEMOTAXIS PROTEIN CHEA;                                   1834:  4hwa-F  4.4  4.5   61   256    8   MOLECULE: SMALL-CONDUCTANCE MECHANOSENSITIVE CHANNEL;                1835:  4hw9-B  4.4  4.3   56   253    7   MOLECULE: MECHANOSENSITIVE CHANNEL MSCS;                             1836:  4hw9-G  4.4  4.4   55   253    7   MOLECULE: MECHANOSENSITIVE CHANNEL MSCS;                             1837:  4hw9-C  4.4  4.5   56   253    7   MOLECULE: MECHANOSENSITIVE CHANNEL MSCS;                             1838:  4hwa-A  4.4  4.4   61   256    8   MOLECULE: SMALL-CONDUCTANCE MECHANOSENSITIVE CHANNEL;                1839:  4hwa-D  4.4  4.5   61   256    8   MOLECULE: SMALL-CONDUCTANCE MECHANOSENSITIVE CHANNEL;                1840:  3din-A  4.4  6.7   66   816    9   MOLECULE: PROTEIN TRANSLOCASE SUBUNIT SECA;                          1841:  5aji-D  4.4  4.5   62   263    8   MOLECULE: SMALL-CONDUCTANCE MECHANOSENSITIVE CHANNEL;                1842:  4hwa-E  4.4  4.4   60   256    8   MOLECULE: SMALL-CONDUCTANCE MECHANOSENSITIVE CHANNEL;                1843:  4hwa-C  4.4  4.4   60   256    8   MOLECULE: SMALL-CONDUCTANCE MECHANOSENSITIVE CHANNEL;                1844:  6ds9-A  4.4  2.3   57    93    2   MOLECULE: DE NOVO DESIGNED THREE HELIX BUNDLE GRA3D;                 1845:  6fhj-A  4.4  3.5   58   979    9   MOLECULE: PROTEIN,PROTEIN;                                           1846:  4gwp-D  4.4  3.5   71   121    3   MOLECULE: MEDIATOR OF RNA POLYMERASE II TRANSCRIPTION SUBUN          1847:  2ic6-A  4.4  2.3   59    71    2   MOLECULE: NUCLEOCAPSID PROTEIN;                                      1848:  5c8f-A  4.4  2.6   50   274    8   MOLECULE: LIGHT-DEPENDENT TRANSCRIPTIONAL REGULATOR CARH;            1849:  6yvv-A  4.4  3.4   84   465    8   MOLECULE: STRUCTURAL MAINTENANCE OF CHROMOSOMES PROTEIN 2,S          1850:  4zh4-C  4.4  4.0   68  1340   13   MOLECULE: DNA-DIRECTED RNA POLYMERASE SUBUNIT ALPHA;                 1851:  4wwb-A  4.4  3.8   70   112    9   MOLECULE: NICKEL AND COBALT RESISTANCE PROTEIN CNRR;                 1852:  7pgi-E  4.4  4.8   75   147    7   MOLECULE: ION TRANSPORT PROTEIN;                                     1853:  8v2d-y  4.4  3.1   57   307    5   MOLECULE: O43_129 COMPONENT B;                                       1854:  6ud4-H  4.4  4.0   70   134    4   MOLECULE: GLUTAMATE RECEPTOR 2;                                      1855:  6nrb-5  4.4  5.0   75   127    9   MOLECULE: T-COMPLEX PROTEIN 1 SUBUNIT ALPHA;                         1856:  6rgv-A  4.4  2.9   80   406   13   MOLECULE: FLAGELLIN;                                                 1857:  6h2d-P  4.4  2.5   84   215    7   MOLECULE: AHLC;                                                      1858:  6ud4-F  4.4  4.0   70   134    4   MOLECULE: GLUTAMATE RECEPTOR 2;                                      1859:  5vot-E  4.4  2.2   60   169    7   MOLECULE: GLUTAMATE RECEPTOR 2;                                      1860:  7ux1-D  4.4  4.5   65   713   12   MOLECULE: MECHANOSENSITIVE CHANNEL MSCK;                             1861:  5ip0-C  4.4  3.9   70    90    9   MOLECULE: PHA GRANULE-ASSOCIATED PROTEIN;                            1862:  6woz-D  4.4  1.9   67   296    3   MOLECULE: RETICULOCYTE BINDING PROTEIN 2B;                           1863:  8cye-J  4.4  4.0   79   269   10   MOLECULE: FLAGELLIN;                                                 1864:  8fd6-A  4.4  2.8   67  2747    9   MOLECULE: CYTOPLASMIC DYNEIN 1 HEAVY CHAIN 1;                        1865:  8cvi-A  4.4  5.1   79   267   10   MOLECULE: FLAGELLIN;                                                 1866:  1zww-B  4.4  3.4   78   196    3   MOLECULE: SH3-CONTAINING GRB2-LIKE PROTEIN 2;                        1867:  6m6z-D  4.4  3.0   68   203   12   MOLECULE: TMH4C4;                                                    1868:  8cvi-B  4.4  3.5   75   267   13   MOLECULE: FLAGELLIN;                                                 1869:  7sn7-J  4.4  3.5   67   546    7   MOLECULE: FLAGELLIN;                                                 1870:  5w53-A  4.4  1.9   67   303    3   MOLECULE: RETICULOCYTE BINDING PROTEIN 2, PUTATIVE;                  1871:  6wn1-D  4.4  1.8   58   235    2   MOLECULE: RETICULOCYTE BINDING PROTEIN 2B;                           1872:  1zww-A  4.4  2.9   75   199    4   MOLECULE: SH3-CONTAINING GRB2-LIKE PROTEIN 2;                        1873:  3jc5-A  4.3  3.0   65   208   12   MOLECULE: DNA REPLICATION LICENSING FACTOR MCM2;                     1874:  8pm4-A  4.3  3.3   62   604   11   MOLECULE: TRANSPOSASE;                                               1875:  6orb-A  4.3  3.0   66  3352    3   MOLECULE: MIDASIN;                                                   1876:  8gmh-A  4.3  2.4   64   223    8   MOLECULE: LXG DOMAIN-CONTAINING PROTEIN;                             1877:  6ta5-D  4.3  2.8   61   345    8   MOLECULE: OUTER MEMBRANE PROTEIN OPRM;                               1878:  6gy6-A  4.3  2.5   71   365    3   MOLECULE: XAXA;                                                      1879:  8hf2-A  4.3  4.0   60   120    8   MOLECULE: PRA1 FAMILY PROTEIN;                                       1880:  6m31-A  4.3  4.4   64   280    3   MOLECULE: DIGERANYLGERANYLGLYCERYL PHOSPHATE SYNTHASE;               1881:  4ut1-A  4.3  3.1   68   551   13   MOLECULE: FLAGELLAR HOOK-ASSOCIATED PROTEIN;                         1882:  5oqm-n  4.3  3.4   64   136    3   MOLECULE: DNA-DIRECTED RNA POLYMERASE II SUBUNIT RPB1;               1883:  8ij1-D  4.3  2.7   56   627    4   MOLECULE: CULLIN-2;                                                  1884:  7zcm-B  4.3  3.8   58    66    7   MOLECULE: SENSORY RHODOPSIN-2;                                       1885:  7vyx-A  4.3  3.3   66  1211   11   MOLECULE: SELENOMETHIONINE (SEMET)-LABELED CAS12C1 D969A MU          1886:  7o3v-F  4.3  3.7   69   261    6   MOLECULE: TRWJ PROTEIN;                                              1887:  1wgz-A  4.3  2.7   64   510    8   MOLECULE: CARBOXYPEPTIDASE 1;                                        1888:  8bcs-A  4.3  1.9   47    49    2   MOLECULE: CC-HP1.0;                                                  1889:  7pbk-A  4.3  7.5   74   624    1   MOLECULE: DNA POLYMERASE I;                                          1890:  8hpo-E  4.3  3.4   73   255   14   MOLECULE: TRANSCRIPTIONAL REGULATORY PROTEIN UME1;                   1891:  8gxl-B  4.3  2.9   60   122    8   MOLECULE: SURP AND G-PATCH DOMAIN-CONTAINING PROTEIN 1;              1892:  7rz4-A  4.3  3.1   65   993    2   MOLECULE: GLUTAMATE RECEPTOR 2;                                      1893:  7wo9-A  4.3  4.5   72  1581    8   MOLECULE: NUCLEOPORIN NUP188;                                        1894:  3haj-A  4.3  2.7   78   286    9   MOLECULE: HUMAN PACSIN2 F-BAR;                                       1895:  6bbf-O  4.3  2.0   55   150   13   MOLECULE: CALCIUM RELEASE-ACTIVATED CALCIUM CHANNEL PROTEIN          1896:  1wp8-A  4.3  2.9   59    64   12   MOLECULE: FUSION GLYCOPROTEIN F0,FUSION GLYCOPROTEIN F0;             1897:  8fih-B  4.3  2.6   61   104    2   MOLECULE: 3HB05;                                                     1898:  6m3p-A  4.3  2.3   74   301    7   MOLECULE: ANKYRIN-3;                                                 1899:  3rrk-A  4.3  3.7   79   301   10   MOLECULE: V-TYPE ATPASE 116 KDA SUBUNIT;                             1900:  6wge-A  4.3  2.9   70   390    6   MOLECULE: STRUCTURAL MAINTENANCE OF CHROMOSOMES PROTEIN 1A;          1901:  1e79-G  4.3  2.3   67   263    4   MOLECULE: ATP SYNTHASE ALPHA CHAIN HEART ISOFORM;                    1902:  4yln-C  4.3  4.9   72  1341    7   MOLECULE: DNA-DIRECTED RNA POLYMERASE SUBUNIT ALPHA;                 1903:  7oie-Y  4.3  2.9   60   176    5   MOLECULE: 39S RIBOSOMAL PROTEIN L2, MITOCHONDRIAL;                   1904:  3lbx-B  4.3  2.9   65   182    5   MOLECULE: SPECTRIN ALPHA CHAIN, ERYTHROCYTE;                         1905:  8j0h-D  4.3  2.8   58   129    3   MOLECULE: UNCHARACTERIZED PROTEIN C4H3.06;                           1906:  7zgj-C  4.3  3.7   78   192    6   MOLECULE: COMPLEMENT C3 BETA CHAIN;                                  1907:  7oib-Y  4.3  2.9   60   176    5   MOLECULE: 39S RIBOSOMAL PROTEIN L2, MITOCHONDRIAL;                   1908:  2zbi-B  4.3  2.8   78   267    6   MOLECULE: FLAGELLIN HOMOLOG;                                         1909:  6ptn-a  4.3  3.3   66   208   12   MOLECULE: DNA POLYMERASE ALPHA-BINDING PROTEIN;                      1910:  4jlr-C  4.3  3.5   65   100    5   MOLECULE: MOTAVIZUMAB FAB HEAVY CHAIN;                               1911:  8e0m-K  4.3  2.7   58   169   14   MOLECULE: BGL15;                                                     1912:  6xky-Q  4.3  2.5   82   271    9   MOLECULE: FLAGELLIN;                                                 1913:  3oeo-A  4.3  7.0   74    91    7   MOLECULE: SPHEROPLAST PROTEIN Y;                                     1914:  4e3c-F  4.3  2.4   83   624    6   MOLECULE: INHIBITOR OF NUCLEAR FACTOR KAPPA-B KINASE SUBUNI          1915:  7oi9-Y  4.3  3.1   61   176    7   MOLECULE: 39S RIBOSOMAL PROTEIN L2, MITOCHONDRIAL;                   1916:  6vud-B  4.3  4.3   68   185    4   MOLECULE: RIBOSOME-RECYCLING FACTOR;                                 1917:  2h7o-A  4.3  8.7   78   270    8   MOLECULE: PROTEIN KINASE YPKA;                                       1918:  6n4q-A  4.3  8.9   75   261    5   MOLECULE: NAV1.7 VSD2-NAVAB CHIMERA;                                 1919:  6nyk-A  4.3  1.6   49    91    4   MOLECULE: DESIGN CONSTRUCT XAX_GGDQ;                                 1920:  1fpo-A  4.3  2.3   59   171    8   MOLECULE: CHAPERONE PROTEIN HSCB;                                    1921:  6o58-G  4.3  7.3   76   250    9   MOLECULE: CALCIUM UNIPORTER PROTEIN, MITOCHONDRIAL;                  1922:  7s7g-A  4.3  3.6   64   571    2   MOLECULE: VERY LONG-CHAIN SPECIFIC ACYL-COA DEHYDROGENASE,           1923:  5ls9-C  4.3  4.2   65   163   12   MOLECULE: FERRITIN, PUTATIVE;                                        1924:  8iwh-z  4.3  2.7   55    61    7   MOLECULE: PHOTOSYSTEM II PROTEIN D1;                                 1925:  5xa5-A  4.3  4.3   65   248    5   MOLECULE: ALPHA-CATENIN-LIKE PROTEIN HMP-1;                          1926:  8gae-C  4.3  2.8   75   259    9   MOLECULE: HEAT SHOCK PROTEIN HSP 90-BETA;                            1927:  7jw1-E  4.3  2.2   83   235   10   MOLECULE: CAPSID PROTEINS;                                           1928:  1ohh-G  4.3  4.2   64    94   16   MOLECULE: ATP SYNTHASE ALPHA CHAIN HEART ISOFORM,                    1929:  6dg6-A  4.3  1.7   53   100    9   MOLECULE: NEOLEUKIN-2/15;                                            1930:  6rwy-V  4.3  2.8   60    73    5   MOLECULE: INNER ROD PROTEIN;                                         1931:  5g54-A  4.3  2.2   65   269    2   MOLECULE: CHLORIDE PUMPING RHODOPSIN;                                1932:  7qru-F  4.3  2.6   53    91    8   MOLECULE: NA+/H+ ANTIPORTER SUBUNIT D;                               1933:  6dg6-F  4.3  1.5   51    97   10   MOLECULE: NEOLEUKIN-2/15;                                            1934:  6ijj-A  4.3  3.8   63   741    5   MOLECULE: PSAA;                                                      1935:  6tq4-A  4.3  2.9   66   297    6   MOLECULE: OREXIN RECEPTOR TYPE 1;                                    1936:  3txq-G  4.3  2.9   67    87    3   MOLECULE: TERMINASE DNA PACKAGING ENZYME SMALL SUBUNIT;              1937:  4uxz-A  4.3  4.1   65   117    8   MOLECULE: DIACYLGLYCEROL KINASE-DELTA 7;                             1938:  2wcd-L  4.3  3.1   80   285   11   MOLECULE: HEMOLYSIN E, CHROMOSOMAL;                                  1939:  4uxz-C  4.3  4.2   64    90    8   MOLECULE: DIACYLGLYCEROL KINASE-DELTA 7;                             1940:  5d57-C  4.3  4.2   64    92    8   MOLECULE: DIACYLGLYCEROL KINASE;                                     1941:  6ys8-G  4.3  2.6   54    60    7   MOLECULE: GLDM;                                                      1942:  4xwp-A  4.3  4.0   80   236    6   MOLECULE: ESX-1 SECRETION-ASSOCIATED PROTEIN ESPB;                   1943:  6xqn-B  4.3  5.7   80   151    9   MOLECULE: PROTEIN EMRE HOMOLOG, MITOCHONDRIAL-LIKE PROTEIN;          1944:  4hw9-F  4.3  4.5   56   253    7   MOLECULE: MECHANOSENSITIVE CHANNEL MSCS;                             1945:  4hw9-E  4.3  4.5   56   253    7   MOLECULE: MECHANOSENSITIVE CHANNEL MSCS;                             1946:  4hwa-B  4.3  4.4   60   256    8   MOLECULE: SMALL-CONDUCTANCE MECHANOSENSITIVE CHANNEL;                1947:  4hw9-D  4.3  4.5   56   253    7   MOLECULE: MECHANOSENSITIVE CHANNEL MSCS;                             1948:  4hwa-G  4.3  4.4   61   256    8   MOLECULE: SMALL-CONDUCTANCE MECHANOSENSITIVE CHANNEL;                1949:  7k7k-K  4.3  4.1   77   171    8   MOLECULE: TRANSLOCON ESPA;                                           1950:  2ld3-A  4.3  3.3   56    88    4   MOLECULE: MYOSIN VI;                                                 1951:  4l8i-A  4.3  4.4   66   114   11   MOLECULE: RSV EPITOPE SCAFFOLD FFL_005;                              1952:  7vrb-A  4.3  3.1   56   112    4   MOLECULE: SMARCA4 PROTEIN,PROTEIN SSXT;                              1953:  4um2-A  4.3  2.2   58   510   16   MOLECULE: TELOMERASE-BINDING PROTEIN EST1A;                          1954:  1yke-B  4.3  2.9   60   119    5   MOLECULE: RNA POLYMERASE II MEDIATOR COMPLEX PROTEIN MED7;           1955:  8evm-C  4.3  2.2   52   237   13   MOLECULE: CHLOROPHYLL DIMER PROTEIN DESIGNS, SPECIAL PAIR 3          1956:  5hfl-E  4.3  3.3   61    65    7   MOLECULE: ENVELOPE GLYCOPROTEIN,GP41 CHR REGION;                     1957:  6dfk-G  4.3  3.0   79   213    5   MOLECULE: SUBUNIT OF PROTEASEOME ACTIVATOR COMPLEX,PUTATIVE          1958:  4ke2-A  4.3  5.6   83   196    6   MOLECULE: TYPE I HYPERACTIVE ANTIFREEZE PROTEIN;                     1959:  5img-A  4.3  5.2   65   467    5   MOLECULE: DIPEPTIDASE;                                               1960:  7pgi-A  4.3  4.7   74   146    7   MOLECULE: ION TRANSPORT PROTEIN;                                     1961:  8d06-E  4.3  3.5   59    66    3   MOLECULE: HALC3_104;                                                 1962:  7emf-2  4.3  3.6   71   115    6   MOLECULE: MEDIATOR OF RNA POLYMERASE II TRANSCRIPTION SUBUN          1963:  7adp-E  4.3  3.2   57    92    9   MOLECULE: ER MEMBRANE PROTEIN COMPLEX SUBUNIT 1;                     1964:  5jje-B  4.3  3.0   54    63    7   MOLECULE: SENSORY RHODOPSIN-2;                                       1965:  6oqr-P  4.3  3.5   55    77    2   MOLECULE: ATP SYNTHASE SUBUNIT DELTA;                                1966:  6wnr-R  4.3  3.5   54    77    2   MOLECULE: ATP SYNTHASE SUBUNIT DELTA;                                1967:  7s6j-B  4.3  3.7   70  1073    6   MOLECULE: SPIKE GLYCOPROTEIN;                                        1968:  6d04-F  4.3  3.4   68   466   10   MOLECULE: TRANSFERRIN RECEPTOR PROTEIN 1;                            1969:  8w9a-B  4.3  3.3   84   276    7   MOLECULE: PHOSPHATIDYLINOSITOL 4,5-BISPHOSPHATE 3-KINASE CA          1970:  8cvi-N  4.3  2.8   82   268   11   MOLECULE: FLAGELLIN;                                                 1971:  4l2y-B  4.3  3.4   82   277    9   MOLECULE: PHOSPHATIDYLINOSITOL 4,5-BISPHOSPHATE 3-KINASE CA          1972:  1wp8-C  4.3  2.1   56    63   11   MOLECULE: FUSION GLYCOPROTEIN F0,FUSION GLYCOPROTEIN F0;             1973:  8cvi-c  4.3  2.9   82   269   11   MOLECULE: FLAGELLIN;                                                 1974:  5wjt-e  4.3  3.6   67   302   13   MOLECULE: FLAGELLIN;                                                 1975:  8cvi-F  4.3  2.8   82   268   11   MOLECULE: FLAGELLIN;                                                 1976:  8cvi-U  4.3  2.9   82   269   12   MOLECULE: FLAGELLIN;                                                 1977:  6x80-J  4.3  5.2   76   574    7   MOLECULE: FLAGELLIN A;                                               1978:  5ip0-K  4.3  3.9   70    90    9   MOLECULE: PHA GRANULE-ASSOCIATED PROTEIN;                            1979:  8v8v-B  4.3  2.6   71   144    3   MOLECULE: PHOSPHATIDYLINOSITOL 4,5-BISPHOSPHATE 3-KINASE CA          1980:  6jfl-A  4.3  2.7   67   413   10   MOLECULE: MITOFUSIN-2,CDNA FLJ57997, HIGHLY SIMILAR TO TRAN          1981:  3gi9-C  4.2  3.1   61   437    8   MOLECULE: 7F11 ANTI-APCT MONOCLONAL FAB LIGHT CHAIN;                 1982:  4nqj-A  4.2  2.6   64   177   11   MOLECULE: E3 UBIQUITIN-PROTEIN LIGASE TRIM69;                        1983:  3j83-A  4.2  3.4   83   276    6   MOLECULE: ESX-1 SECRETION-ASSOCIATED PROTEIN ESPB;                   1984:  5wjt-A  4.2  2.2   85   302    2   MOLECULE: FLAGELLIN;                                                 1985:  5iun-E  4.2  4.3   67   220    3   MOLECULE: SENSOR HISTIDINE KINASE DESK;                              1986:  2azj-A  4.2  4.8   59   276    3   MOLECULE: GERANYLGERANYL PYROPHOSPHATE SYNTHETASE;                   1987:  7jgs-C  4.2  3.6   53   614    4   MOLECULE: ORIGIN RECOGNITION COMPLEX SUBUNIT 2;                      1988:  8ivz-A  4.2  5.2   62   165    2   MOLECULE: TALIN-1;                                                   1989:  7mq1-B  4.2  2.2   54    83    6   MOLECULE: COPPER-SENSING TRANSCRIPTIONAL REPRESSOR CSOR;             1990:  6qm5-A  4.2  4.6   77   673    3   MOLECULE: PREDICTED PROTEIN;                                         1991:  6csm-D  4.2  2.6   63   277   11   MOLECULE: GTACR1;                                                    1992:  6cs2-C  4.2  3.9   69   893    6   MOLECULE: SPIKE GLYCOPROTEIN,FIBRITIN;                               1993:  7w1m-D  4.2  2.5   75   967    4   MOLECULE: STRUCTURAL MAINTENANCE OF CHROMOSOMES PROTEIN 1A;          1994:  4v19-2  4.2  2.9   60   178    5   MOLECULE: MITORIBOSOMAL PROTEIN BL27M, MRPL27;                       1995:  7qe7-I  4.2  2.3   70   742    7   MOLECULE: ANAPHASE-PROMOTING COMPLEX SUBUNIT 10;                     1996:  6s3k-A  4.2  3.5   62   573    3   MOLECULE: APC FAMILY PERMEASE;                                       1997:  7pl9-A  4.2  4.8   73   994    5   MOLECULE: RHODOPSIN;                                                 1998:  2spc-A  4.2  3.1   66   107   14   MOLECULE: SPECTRIN;                                                  1999:  6bx3-F  4.2  4.4   84   215    5   MOLECULE: HISTONE-LYSINE N-METHYLTRANSFERASE, H3 LYSINE-4 S          2000:  5oqj-1  4.2  3.3   59   491    3   MOLECULE: DNA-DIRECTED RNA POLYMERASE II SUBUNIT RPB1;               2001:  3iee-A  4.2  3.0   81   256    9   MOLECULE: PUTATIVE EXPORTED PROTEIN;                                 2002:  1zke-D  4.2  5.7   60    83    3   MOLECULE: HYPOTHETICAL PROTEIN HP1531;                               2003:  7c79-L  4.2  4.2   63   131   10   MOLECULE: RIBONUCLEASE MRP RNA SUBUNIT NME1;                         2004:  1pd3-A  4.2  2.4   47    54   13   MOLECULE: NONSTRUCTURAL PROTEIN NS2;                                 2005:  5jxl-A  4.2  2.8   65   851    5   MOLECULE: FLAGELLAR HOOK PROTEIN FLGE;                               2006:  8tlq-A  4.2  3.3   62  1283    6   MOLECULE: DNA POLYMERASE ZETA CATALYTIC SUBUNIT;                     2007:  7qhm-A  4.2  3.0   70   402    3   MOLECULE: CYTOCHROME BC1 COMPLEX RIESKE IRON-SULFUR SUBUNIT          2008:  7z0l-B  4.2  4.2   68   370    7   MOLECULE: INTERLEUKIN-6 RECEPTOR SUBUNIT BETA;                       2009:  7p2y-a  4.2  3.2   65   277    6   MOLECULE: ATP SYNTHASE SUBUNIT ALPHA;                                2010:  8oyv-A  4.2  3.0   63   182    6   MOLECULE: DE NOVO DESIGNED SOLUBLE CLAUDIN;                          2011:  6iiv-A  4.2  5.4   71   461    6   MOLECULE: SOLUBLE CYTOCHROME B562,THROMBOXANE A2 RECEPTOR,R          2012:  8fbi-A  4.2  2.5   69   274    7   MOLECULE: KWOCA_39;                                                  2013:  6pwc-R  4.2  4.2   75   323    4   MOLECULE: NEUROTENSIN RECEPTOR TYPE 1;                               2014:  7y8r-S  4.2  4.3   60   213   10   MOLECULE: HISTONE H3;                                                2015:  3cqx-C  4.2  2.6   59    81    7   MOLECULE: HEAT SHOCK COGNATE 71 KDA PROTEIN;                         2016:  4j9y-B  4.2  6.2   66    89   11   MOLECULE: SMALL CONDUCTANCE CALCIUM-ACTIVATED POTASSIUM CHA          2017:  3i9w-A  4.2  2.5   71   270    8   MOLECULE: SENSOR PROTEIN TORS;                                       2018:  5ual-I  4.2  4.5   73  1328    7   MOLECULE: DNA-DIRECTED RNA POLYMERASE SUBUNIT ALPHA;                 2019:  6xpf-A  4.2  4.4   61   291    3   MOLECULE: ZINC TRANSPORTER 8;                                        2020:  5vou-F  4.2  3.0   66   175    5   MOLECULE: GLUTAMATE RECEPTOR 2;                                      2021:  3frs-A  4.2  2.8   72   175   10   MOLECULE: UNCHARACTERIZED PROTEIN KIAA0174;                          2022:  6vhr-A  4.2  3.2   62    73   11   MOLECULE: PE FAMILY IMMUNOMODULATOR PE5;                             2023:  6egc-A  4.2  3.4   71   146   13   MOLECULE: SC_2L4HC2_23;                                              2024:  5mke-B  4.2  7.6   79   476    5   MOLECULE: POLYCYSTIN-2;                                              2025:  6pe5-R  4.2  2.5   62   369    5   MOLECULE: V-TYPE PROTON ATPASE SUBUNIT A, VACUOLAR ISOFORM;          2026:  6bbf-U  4.2  2.4   57   150    5   MOLECULE: CALCIUM RELEASE-ACTIVATED CALCIUM CHANNEL PROTEIN          2027:  6bbf-H  4.2  2.4   57   150    5   MOLECULE: CALCIUM RELEASE-ACTIVATED CALCIUM CHANNEL PROTEIN          2028:  3l8r-A  4.2  1.7   57   102    7   MOLECULE: PUTATIVE PTS SYSTEM, CELLOBIOSE-SPECIFIC IIA               2029:  5vot-H  4.2  3.1   64   175    5   MOLECULE: GLUTAMATE RECEPTOR 2;                                      2030:  4nx9-A  4.2  2.9   78   277   10   MOLECULE: FLAGELLIN;                                                 2031:  4uzy-A  4.2  2.6   64   624   13   MOLECULE: FLAGELLAR ASSOCIATED PROTEIN;                              2032:  7of0-Y  4.2  3.0   60   176    5   MOLECULE: 39S RIBOSOMAL PROTEIN L32, MITOCHONDRIAL;                  2033:  1kfd-A  4.2  2.7   56   560    5   MOLECULE: DNA POLYMERASE I KLENOW FRAGMENT;                          2034:  5vou-G  4.2  3.0   64   169    5   MOLECULE: GLUTAMATE RECEPTOR 2;                                      2035:  6mem-L  4.2  5.1   73   733    0   MOLECULE: CHLOROPHYLL A/B BINDING PROTEIN 1;                         2036:  8hf2-C  4.2  4.1   60   120    8   MOLECULE: PRA1 FAMILY PROTEIN;                                       2037:  7nz0-C  4.2  3.1   81   335    9   MOLECULE: CHROMOSOME PARTITION PROTEIN MUKB;                         2038:  6wm2-G  4.2  2.4   83   213    8   MOLECULE: V-TYPE PROTON ATPASE SUBUNIT E 1;                          2039:  6drj-A  4.2  6.8   77  1264    9   MOLECULE: TRANSIENT RECEPTOR POTENTIAL CATION CHANNEL, SUBF          2040:  8hf2-B  4.2  4.0   60   120    8   MOLECULE: PRA1 FAMILY PROTEIN;                                       2041:  8hf2-D  4.2  4.1   60   120    8   MOLECULE: PRA1 FAMILY PROTEIN;                                       2042:  4uyo-E  4.2  3.2   63   101   10   MOLECULE: DIACYLGLYCEROL KINASE-DELTA 7;                             2043:  7e84-A  4.2  5.1   75   460    7   MOLECULE: POTASSIUM VOLTAGE-GATED CHANNEL SUBFAMILY D MEMBE          2044:  6xz6-C  4.2  3.2   83   213   10   MOLECULE: GARP DOMAIN-CONTAINING PROTEIN;                            2045:  1xg2-B  4.2  2.2   58   151   10   MOLECULE: PECTINESTERASE 1;                                          2046:  5h2h-A  4.2  3.3   60   231    5   MOLECULE: BACTERIORHODOPSIN;                                         2047:  5b6z-A  4.2  3.3   60   231    5   MOLECULE: BACTERIORHODOPSIN;                                         2048:  5h2i-A  4.2  3.3   60   231    5   MOLECULE: BACTERIORHODOPSIN;                                         2049:  5h2p-A  4.2  3.3   60   231    5   MOLECULE: BACTERIORHODOPSIN;                                         2050:  5j7a-A  4.2  2.6   57   224    5   MOLECULE: BACTERIORHODOPSIN;                                         2051:  8ehx-A  4.2  4.2   59   631    3   MOLECULE: CSC1-LIKE PROTEIN 2;                                       2052:  5b6v-A  4.2  3.3   60   231    5   MOLECULE: BACTERIORHODOPSIN;                                         2053:  5h2o-A  4.2  3.3   60   231    5   MOLECULE: BACTERIORHODOPSIN;                                         2054:  5b6y-A  4.2  3.3   60   231    5   MOLECULE: BACTERIORHODOPSIN;                                         2055:  5b6x-A  4.2  3.3   60   231    5   MOLECULE: BACTERIORHODOPSIN;                                         2056:  5h2k-A  4.2  3.3   60   231    5   MOLECULE: BACTERIORHODOPSIN;                                         2057:  5b6w-A  4.2  3.3   60   231    5   MOLECULE: BACTERIORHODOPSIN;                                         2058:  5afq-B  4.2  4.6   82   444    7   MOLECULE: DNA-DIRECTED RNA POLYMERASE III SUBUNIT RPC3;              2059:  7f3t-A  4.2  2.1   67   330    6   MOLECULE: TRANSMEMBRANE PROTEIN 120A;                                2060:  3r84-B  4.2  3.6   66    80    6   MOLECULE: MEDIATOR OF RNA POLYMERASE II TRANSCRIPTION SUBUN          2061:  3sjb-D  4.2  2.5   60    72    5   MOLECULE: ATPASE GET3;                                               2062:  2rmk-B  4.2  3.0   67    81    6   MOLECULE: RAS-RELATED C3 BOTULINUM TOXIN SUBSTRATE 1;                2063:  7s5k-C  4.2  2.5   54    68    7   MOLECULE: ENCB;                                                      2064:  5m48-A  4.2  4.1   75   114    8   MOLECULE: REGULATOR OF TY1 TRANSPOSITION PROTEIN 103;                2065:  6vyd-A  4.2  4.8   65   304   11   MOLECULE: TERPENOID CYCLASE FGGS;                                    2066:  7vw1-B  4.2  2.0   50    92   10   MOLECULE: DUF305 DOMAIN-CONTAINING PROTEIN;                          2067:  7vw1-A  4.2  2.0   50    85   10   MOLECULE: DUF305 DOMAIN-CONTAINING PROTEIN;                          2068:  1ovu-A  4.2  1.8   46    48    2   MOLECULE: FOUR-HELIX BUNDLE MODEL DI-CO(II)-DF1-L13A (FORM           2069:  8dl7-A  4.2  4.9   75   467    9   MOLECULE: SOLUTE CARRIER FAMILY 40 MEMBER 1;                         2070:  2yf4-B  4.2  2.2   59   146    3   MOLECULE: MAZG-LIKE NUCLEOSIDE TRIPHOSPHATE PYROPHOSPHOHYDR          2071:  1vf7-E  4.2  2.3   58   252    9   MOLECULE: MULTIDRUG RESISTANCE PROTEIN MEXA;                         2072:  1skv-D  4.2  2.8   55    56    5   MOLECULE: HYPOTHETICAL 7.5 KDA PROTEIN;                              2073:  7zme-6  4.2  2.2   50   190    4   MOLECULE: NADH-UBIQUINONE OXIDOREDUCTASE CHAIN 1;                    2074:  5flm-A  4.2  6.2   64  1427    8   MOLECULE: DNA-DIRECTED RNA POLYMERASE;                               2075:  8akr-g  4.2  2.8   78   196    9   MOLECULE: CHLOROPLAST MEMBRANE-ASSOCIATED 30 KD PROTEIN;             2076:  3aqp-A  4.2  3.4   65   719    5   MOLECULE: PROBABLE SECDF PROTEIN-EXPORT MEMBRANE PROTEIN;            2077:  4brr-B  4.2  3.2   61    98   10   MOLECULE: DIACYLGLYCEROL KINASE;                                     2078:  4ckg-A  4.2  2.8   83   368   10   MOLECULE: ARF-GAP WITH COILED-COIL, ANK REPEAT AND PH DOMAI          2079:  7k7k-Y  4.2  4.1   77   171    8   MOLECULE: TRANSLOCON ESPA;                                           2080:  7k7k-E  4.2  4.1   77   171    8   MOLECULE: TRANSLOCON ESPA;                                           2081:  5aji-E  4.2  4.5   62   262    8   MOLECULE: SMALL-CONDUCTANCE MECHANOSENSITIVE CHANNEL;                2082:  7k7k-C  4.2  4.1   77   171    8   MOLECULE: TRANSLOCON ESPA;                                           2083:  7k7k-S  4.2  4.1   77   171    8   MOLECULE: TRANSLOCON ESPA;                                           2084:  7k7k-J  4.2  4.1   77   171    8   MOLECULE: TRANSLOCON ESPA;                                           2085:  7k7k-N  4.2  4.1   77   171    8   MOLECULE: TRANSLOCON ESPA;                                           2086:  7k7k-U  4.2  4.1   77   171    8   MOLECULE: TRANSLOCON ESPA;                                           2087:  5tqb-B  4.2  2.0   59   335    5   MOLECULE: 60S RIBOSOMAL PROTEIN L4-LIKE PROTEIN;                     2088:  2xq2-A  4.2  3.2   74   538    5   MOLECULE: SODIUM/GLUCOSE COTRANSPORTER;                              2089:  2yfa-A  4.2  4.3   85   236   12   MOLECULE: METHYL-ACCEPTING CHEMOTAXIS TRANSDUCER;                    2090:  6nbx-G  4.2  1.8   49   191    6   MOLECULE: NAD(P)H-QUINONE OXIDOREDUCTASE SUBUNIT 1;                  2091:  8ovx-Y  4.2  6.4   65   203    5   MOLECULE: INNER KINETOCHORE SUBUNIT MCM21;                           2092:  5wjt-B  4.2  2.2   85   302    2   MOLECULE: FLAGELLIN;                                                 2093:  5wjt-R  4.2  2.2   85   302    2   MOLECULE: FLAGELLIN;                                                 2094:  6m6z-C  4.2  2.8   67   203   12   MOLECULE: TMH4C4;                                                    2095:  6x80-Q  4.2  3.4   70   574    7   MOLECULE: FLAGELLIN A;                                               2096:  6cs1-C  4.2  3.7   69  1068    6   MOLECULE: SPIKE GLYCOPROTEIN,FIBRITIN;                               2097:  5wjt-K  4.2  2.2   85   302    2   MOLECULE: FLAGELLIN;                                                 2098:  5wjt-F  4.2  2.2   85   302    2   MOLECULE: FLAGELLIN;                                                 2099:  6jfl-C  4.2  2.7   67   412   10   MOLECULE: MITOFUSIN-2,CDNA FLJ57997, HIGHLY SIMILAR TO TRAN          2100:  8cvi-R  4.2  2.8   82   268   11   MOLECULE: FLAGELLIN;                                                 2101:  6rla-A  4.2  6.5   80  2750    5   MOLECULE: O6-ALKYLGUANINE-DNA ALKYLTRANSFERASE MUTANT,DYNC2          2102:  6rla-B  4.2  6.5   80  2750    5   MOLECULE: O6-ALKYLGUANINE-DNA ALKYLTRANSFERASE MUTANT,DYNC2          2103:  6acg-B  4.2  3.6   68  1065    6   MOLECULE: SPIKE GLYCOPROTEIN;                                        2104:  5x58-A  4.2  3.8   70  1054    7   MOLECULE: SPIKE GLYCOPROTEIN;                                        2105:  8cvi-d  4.2  2.8   82   269   12   MOLECULE: FLAGELLIN;                                                 2106:  5wjt-T  4.2  2.2   85   302    2   MOLECULE: FLAGELLIN;                                                 2107:  5wjt-S  4.2  2.2   85   302    2   MOLECULE: FLAGELLIN;                                                 2108:  5wjt-P  4.2  2.2   85   302    2   MOLECULE: FLAGELLIN;                                                 2109:  5wjt-N  4.2  2.2   85   302    2   MOLECULE: FLAGELLIN;                                                 2110:  5wjt-D  4.2  2.2   85   302    2   MOLECULE: FLAGELLIN;                                                 2111:  5wjt-f  4.2  2.2   85   302    2   MOLECULE: FLAGELLIN;                                                 2112:  8h0x-C  4.2  3.6   68  1039    4   MOLECULE: SPIKE GLYCOPROTEIN;                                        2113:  5wjt-W  4.2  2.2   85   302    2   MOLECULE: FLAGELLIN;                                                 2114:  2zbi-A  4.2  3.0   74   265    9   MOLECULE: FLAGELLIN HOMOLOG;                                         2115:  5wjt-O  4.2  2.2   85   302    2   MOLECULE: FLAGELLIN;                                                 2116:  5wjt-U  4.2  2.2   85   302    2   MOLECULE: FLAGELLIN;                                                 2117:  8etc-h  4.1  2.7   56   121   13   MOLECULE: RNA (2151-MER);                                            2118:  7nvr-n  4.1  3.7   67   132    6   MOLECULE: TFIIH BASAL TRANSCRIPTION FACTOR COMPLEX HELICASE          2119:  5x11-E  4.1  3.7   65   181   11   MOLECULE: DNA (28-MER);                                              2120:  8srm-B  4.1  4.1   73   235    8   MOLECULE: RB1-INDUCIBLE COILED-COIL PROTEIN 1;                       2121:  2p5t-E  4.1  4.1   57    95   14   MOLECULE: FRAGMENT OF PEZA HELIX-TURN-HELIX MOTIF;                   2122:  3jck-F  4.1  2.5   49   388   10   MOLECULE: 26S PROTEASOME REGULATORY SUBUNIT RPN3;                    2123:  4nb5-B  4.1  3.4   59   149    8   MOLECULE: DNA BINDING PROTEIN;                                       2124:  6w1s-X  4.1  3.2   68   123    6   MOLECULE: MEDIATOR OF RNA POLYMERASE II TRANSCRIPTION SUBUN          2125:  6a70-B  4.1  3.9   68   704    4   MOLECULE: POLYCYSTIN-2;                                              2126:  8bh8-A  4.1  4.5   69   523   10   MOLECULE: PCIF1_WW DOMAIN-CONTAINING PROTEIN;                        2127:  6fwr-A  4.1  3.8   61   699   13   MOLECULE: ATP-DEPENDENT DNA HELICASE DING;                           2128:  4nwp-D  4.1  4.5   50   149   12   MOLECULE: PUTATIVE UNCHARACTERIZED PROTEIN;                          2129:  6ep3-B  4.1  5.7   61   218    8   MOLECULE: LMO0651 PROTEIN;                                           2130:  4h54-A  4.1  2.9   61   274   11   MOLECULE: DIGUANYLATE CYCLASE YDEH;                                  2131:  8djk-A  4.1  4.2   63   245    3   MOLECULE: 3-HYDROXY-3-METHYLGLUTARYL-COENZYME A REDUCTASE;           2132:  3k1s-A  4.1  3.0   66   109    9   MOLECULE: PTS SYSTEM, CELLOBIOSE-SPECIFIC IIA COMPONENT;             2133:  5n9j-V  4.1  3.5   64    99    6   MOLECULE: MEDIATOR OF RNA POLYMERASE II TRANSCRIPTION SUBUN          2134:  4f52-E  4.1  4.3   72   523   10   MOLECULE: CULLIN-1;                                                  2135:  7tpr-A  4.1  4.4   73  1145   12   MOLECULE: SPIKE GLYCOPROTEIN;                                        2136:  3txs-C  4.1  2.9   66    91    8   MOLECULE: TERMINASE DNA PACKAGING ENZYME SMALL SUBUNIT;              2137:  6vbv-7  4.1  4.3   67   706   10   MOLECULE: BARDET-BIEDL SYNDROME 18 PROTEIN;                          2138:  4lun-U  4.1  3.1   66   311    8   MOLECULE: NONSENSE-MEDIATED MRNA DECAY PROTEIN 2;                    2139:  8k89-A  4.1  3.2   63    78   13   MOLECULE: NUCLEAR FACTOR INTERLEUKIN-3-REGULATED PROTEIN;            2140:  4zuz-B  4.1  2.7   58   858    7   MOLECULE: SIDC;                                                      2141:  2r9r-H  4.1  5.2   77   363    4   MOLECULE: VOLTAGE-GATED POTASSIUM CHANNEL SUBUNIT BETA-2;            2142:  3rgu-A  4.1  2.6   56    88    5   MOLECULE: FIMBRIAE-ASSOCIATED PROTEIN FAP1;                          2143:  5h46-A  4.1  2.5   57   149   12   MOLECULE: DNA PROTECTION DURING STARVATION PROTEIN;                  2144:  8jj5-D  4.1  3.1   76   254    7   MOLECULE: TETRASPANIN;                                               2145:  5ynx-A  4.1  2.5   62   117   13   MOLECULE: ALLERGEN DER F 21;                                         2146:  6atg-B  4.1  4.0   79   212    8   MOLECULE: HCG40889, ISOFORM CRA_B;                                   2147:  7z31-O  4.1  5.4   79   568    6   MOLECULE: DNA-DIRECTED RNA POLYMERASE III SUBUNIT RPC1;              2148:  3ehf-A  4.1  2.8   58   179   10   MOLECULE: SENSOR KINASE (YOCF PROTEIN);                              2149:  3nrx-B  4.1  2.5   61   118    7   MOLECULE: PROTEIN REGULATOR OF CYTOKINESIS 1;                        2150:  1ge9-A  4.1  5.3   71   184   11   MOLECULE: RIBOSOME RECYCLING FACTOR;                                 2151:  8cr2-C  4.1  4.3   68    97    4   MOLECULE: ATPASE ASNA1;                                              2152:  4h63-H  4.1  4.1   66   174    0   MOLECULE: MEDIATOR OF RNA POLYMERASE II TRANSCRIPTION SUBUN          2153:  6uuj-A  4.1  3.2   61    73   13   MOLECULE: PE FAMILY IMMUNOMODULATOR PE5;                             2154:  8p3z-F  4.1  2.4   62   177    6   MOLECULE: GLUTAMATE RECEPTOR 2;                                      2155:  6peq-F  4.1  2.5   67   134    3   MOLECULE: GLUTAMATE RECEPTOR 2;                                      2156:  3s84-A  4.1  3.9   69   239    7   MOLECULE: APOLIPOPROTEIN A-IV;                                       2157:  8p3y-H  4.1  2.4   62   177    6   MOLECULE: GLUTAMATE RECEPTOR 2;                                      2158:  2odu-A  4.1  2.7   74   217    8   MOLECULE: PLECTIN 1;                                                 2159:  3m9v-A  4.1  4.2   72   390   10   MOLECULE: FAD-DEPENDENT OXIDOREDUCTASE;                              2160:  8ppr-M  4.1  6.2   70   205    7   MOLECULE: KINETOCHORE-ASSOCIATED PROTEIN DSN1 HOMOLOG;               2161:  8c1r-F  4.1  2.4   62   178    6   MOLECULE: GLUTAMATE RECEPTOR 2;                                      2162:  6o7t-h  4.1  4.8   74   159    5   MOLECULE: V-TYPE PROTON ATPASE SUBUNIT A, VACUOLAR ISOFORM;          2163:  4g80-I  4.1  3.3   63   139    8   MOLECULE: VOLTAGE-SENSOR CONTAINING PHOSPHATASE;                     2164:  6tms-D  4.1  3.4   61    69    5   MOLECULE: A NOVEL DESIGNED PORE PROTEIN;                             2165:  6xl0-F  4.1  3.0   85   271    9   MOLECULE: FLAGELLIN;                                                 2166:  6xl0-S  4.1  3.0   86   271    9   MOLECULE: FLAGELLIN;                                                 2167:  6xl0-E  4.1  3.0   85   271    9   MOLECULE: FLAGELLIN;                                                 2168:  1m1j-A  4.1  3.2   76   192   11   MOLECULE: FIBRINOGEN ALPHA SUBUNIT;                                  2169:  6xl0-O  4.1  3.0   86   271    9   MOLECULE: FLAGELLIN;                                                 2170:  7pl9-B  4.1  3.3   60   994    7   MOLECULE: RHODOPSIN;                                                 2171:  6xl0-C  4.1  3.0   86   271    9   MOLECULE: FLAGELLIN;                                                 2172:  4ke2-C  4.1  5.9   83   196    7   MOLECULE: TYPE I HYPERACTIVE ANTIFREEZE PROTEIN;                     2173:  6xl0-D  4.1  3.0   86   271    9   MOLECULE: FLAGELLIN;                                                 2174:  6xl0-G  4.1  3.0   85   271    9   MOLECULE: FLAGELLIN;                                                 2175:  7vg4-A  4.1  2.7   77   204    6   MOLECULE: METHENYLTETRAHYDROFOLATE CYCLOHYDROLASE;                   2176:  6wg3-D  4.1  2.3   69   932   10   MOLECULE: STRUCTURAL MAINTENANCE OF CHROMOSOMES PROTEIN 1A;          2177:  1k04-A  4.1  3.4   66   142   11   MOLECULE: FOCAL ADHESION KINASE 1;                                   2178:  6myo-D  4.1  4.2   54   102    7   MOLECULE: SUCCINATE DEHYDROGENASE [UBIQUINONE] FLAVOPROTEIN          2179:  6xl0-R  4.1  3.0   86   271    9   MOLECULE: FLAGELLIN;                                                 2180:  6sih-F  4.1  3.3   80   488    5   MOLECULE: FLAGELLAR HOOK-ASSOCIATED PROTEIN 2;                       2181:  6xl0-J  4.1  3.0   86   271    9   MOLECULE: FLAGELLIN;                                                 2182:  6xl0-K  4.1  3.0   85   271    9   MOLECULE: FLAGELLIN;                                                 2183:  4jle-A  4.1  5.7   68   149    9   MOLECULE: PHIST;                                                     2184:  8hf2-E  4.1  4.0   60   120    8   MOLECULE: PRA1 FAMILY PROTEIN;                                       2185:  6xl0-L  4.1  3.0   86   271    9   MOLECULE: FLAGELLIN;                                                 2186:  6xl0-A  4.1  3.0   86   271    9   MOLECULE: FLAGELLIN;                                                 2187:  7rky-R  4.1  3.1   62   252    8   MOLECULE: GUANINE NUCLEOTIDE-BINDING PROTEIN G(I) SUBUNIT A          2188:  7tac-E  4.1  2.8   58   209    0   MOLECULE: REGULATORY PROTEIN NPR1;                                   2189:  8ioc-R  4.1  2.9   67   272    6   MOLECULE: GUANINE NUCLEOTIDE-BINDING PROTEIN G(I) SUBUNIT A          2190:  3ayf-A  4.1  6.8   85   754    2   MOLECULE: NITRIC OXIDE REDUCTASE;                                    2191:  5nl6-A  4.1  2.8   76   235    7   MOLECULE: CALPONIN DOMAIN FAMILY PROTEIN;                            2192:  7f3u-A  4.1  4.4   59   328    5   MOLECULE: TRANSMEMBRANE PROTEIN 120A;                                2193:  6yp7-z  4.1  2.9   56    62    7   MOLECULE: CHLOROPHYLL A-B BINDING PROTEIN 8, CHLOROPLASTIC;          2194:  7zqy-B  4.1  3.1   74   177    3   MOLECULE: DH DOMAIN-CONTAINING PROTEIN;                              2195:  1wp1-A  4.1  2.4   77   456    3   MOLECULE: OUTER MEMBRANE PROTEIN OPRM;                               2196:  1qoy-A  4.1  1.9   81   303   11   MOLECULE: HEMOLYSIN E;                                               2197:  8om1-L  4.1  2.4   53   607    4   MOLECULE: NADH-UBIQUINONE OXIDOREDUCTASE CHAIN 3;                    2198:  5y5x-G  4.1  2.5   80   209   13   MOLECULE: V-TYPE ATP SYNTHASE ALPHA CHAIN;                           2199:  5jld-A  4.1  2.8   52   559    4   MOLECULE: ARGINYL-TRNA SYNTHETASE, PUTATIVE;                         2200:  5f5p-B  4.1  3.3   77   171   10   MOLECULE: PROTEIN SHROOM2;                                           2201:  5t59-D  4.1  5.3   71   101    6   MOLECULE: KLLA0F02343P;                                              2202:  5kum-A  4.1  5.7   70   326    6   MOLECULE: ATP-SENSITIVE INWARD RECTIFIER POTASSIUM CHANNEL           2203:  5lb7-B  4.1  2.1   53    78   13   MOLECULE: KATANIN P80 WD40 REPEAT-CONTAINING SUBUNIT B1;             2204:  4whj-A  4.1  5.7   79   565    9   MOLECULE: INTERFERON-INDUCED GTP-BINDING PROTEIN MX2;                2205:  8akr-f  4.1  2.4   83   196   13   MOLECULE: CHLOROPLAST MEMBRANE-ASSOCIATED 30 KD PROTEIN;             2206:  4d2e-E  4.1  3.5   64    90    9   MOLECULE: DIACYLGLYCEROL KINASE;                                     2207:  5uhc-D  4.1  5.3   74  1265    3   MOLECULE: DNA-DIRECTED RNA POLYMERASE SUBUNIT ALPHA;                 2208:  2uwj-E  4.1  2.0   50    70    6   MOLECULE: TYPE III EXPORT PROTEIN PSCE;                              2209:  3ze5-C  4.1  3.7   63   104   10   MOLECULE: DIACYLGLYCEROL KINASE;                                     2210:  6v9s-A  4.1  2.6   61   503   11   MOLECULE: OREXIN RECEPTOR TYPE 1,GLGA GLYCOGEN SYNTHASE CHI          2211:  7s5k-I  4.1  2.3   53    68    8   MOLECULE: ENCB;                                                      2212:  1ovv-A  4.1  1.9   46    48    2   MOLECULE: FOUR-HELIX BUNDLE MODEL DI-CO(II)-DF1-L13A (FORM           2213:  8szz-x  4.1  2.3   58    70   10   MOLECULE: O32-ZL4 COMPONENT A;                                       2214:  8szz-T  4.1  2.2   58    70   10   MOLECULE: O32-ZL4 COMPONENT A;                                       2215:  8szz-w  4.1  2.2   58    70   10   MOLECULE: O32-ZL4 COMPONENT A;                                       2216:  8szz-u  4.1  2.3   58    70   10   MOLECULE: O32-ZL4 COMPONENT A;                                       2217:  8szz-Q  4.1  2.3   60    70   12   MOLECULE: O32-ZL4 COMPONENT A;                                       2218:  8szz-v  4.1  2.3   58    70   10   MOLECULE: O32-ZL4 COMPONENT A;                                       2219:  8szz-W  4.1  2.3   60    70   12   MOLECULE: O32-ZL4 COMPONENT A;                                       2220:  8szz-R  4.1  2.3   58    70   10   MOLECULE: O32-ZL4 COMPONENT A;                                       2221:  8szz-s  4.1  2.4   60    70   12   MOLECULE: O32-ZL4 COMPONENT A;                                       2222:  8szz-t  4.1  2.2   58    70   10   MOLECULE: O32-ZL4 COMPONENT A;                                       2223:  8szz-q  4.1  2.2   58    70   10   MOLECULE: O32-ZL4 COMPONENT A;                                       2224:  8szz-3  4.1  2.2   58    70   10   MOLECULE: O32-ZL4 COMPONENT A;                                       2225:  8szz-P  4.1  2.3   60    70   12   MOLECULE: O32-ZL4 COMPONENT A;                                       2226:  8szz-r  4.1  2.3   58    70   10   MOLECULE: O32-ZL4 COMPONENT A;                                       2227:  8szz-2  4.1  2.3   58    70   10   MOLECULE: O32-ZL4 COMPONENT A;                                       2228:  8szz-z  4.1  2.4   60    70   12   MOLECULE: O32-ZL4 COMPONENT A;                                       2229:  8szz-0  4.1  2.3   58    70   10   MOLECULE: O32-ZL4 COMPONENT A;                                       2230:  8szz-1  4.1  2.4   60    70   12   MOLECULE: O32-ZL4 COMPONENT A;                                       2231:  7rtu-A  4.1  3.7   80   395    5   MOLECULE: PROTEIN TWEETY HOMOLOG 2;                                  2232:  3sja-H  4.1  2.5   54    61    6   MOLECULE: ATPASE GET3;                                               2233:  4hb1-A  4.1  1.1   42    44   14   MOLECULE: DHP1;                                                      2234:  2m6u-A  4.1  3.8   54    82    9   MOLECULE: CHOLINE BINDING PROTEIN A;                                 2235:  7k7k-R  4.1  4.1   77   171    8   MOLECULE: TRANSLOCON ESPA;                                           2236:  7k7k-O  4.1  4.1   77   171    8   MOLECULE: TRANSLOCON ESPA;                                           2237:  7k7k-F  4.1  4.1   77   171    8   MOLECULE: TRANSLOCON ESPA;                                           2238:  7k7k-H  4.1  4.1   77   171    8   MOLECULE: TRANSLOCON ESPA;                                           2239:  7k7k-L  4.1  4.1   77   171    8   MOLECULE: TRANSLOCON ESPA;                                           2240:  6q6b-A  4.1  3.2   61   132    3   MOLECULE: CYTOSOLIC COPPER STORAGE PROTEIN;                          2241:  7k7k-I  4.1  4.1   77   171    8   MOLECULE: TRANSLOCON ESPA;                                           2242:  7k7k-Q  4.1  4.1   77   171    8   MOLECULE: TRANSLOCON ESPA;                                           2243:  7k7k-G  4.1  4.1   77   171    8   MOLECULE: TRANSLOCON ESPA;                                           2244:  7k7k-P  4.1  4.1   77   171    8   MOLECULE: TRANSLOCON ESPA;                                           2245:  4ayx-A  4.1  4.8   77   572    5   MOLECULE: ATP-BINDING CASSETTE SUB-FAMILY B MEMBER 10;               2246:  7k7k-W  4.1  4.1   77   171    8   MOLECULE: TRANSLOCON ESPA;                                           2247:  7k7k-M  4.1  4.1   77   171    8   MOLECULE: TRANSLOCON ESPA;                                           2248:  7k7k-X  4.1  4.1   77   171    8   MOLECULE: TRANSLOCON ESPA;                                           2249:  5tpt-A  4.1  4.3   73   197    4   MOLECULE: AMYLOID-LIKE PROTEIN 2;                                    2250:  2f1m-C  4.1  2.7   66   247    6   MOLECULE: ACRIFLAVINE RESISTANCE PROTEIN A;                          2251:  1jpx-A  4.1  2.5   53    59    9   MOLECULE: GP41 ENVELOPE PROTEIN;                                     2252:  2de0-X  4.1  5.0   68   460   10   MOLECULE: ALPHA-(1,6)-FUCOSYLTRANSFERASE;                            2253:  1w5c-Y  4.1 26.7   57   359    0   MOLECULE: PHOTOSYSTEM Q(B) PROTEIN 1;                                2254:  7zr0-C  4.1  3.2   76   266   12   MOLECULE: HEAT SHOCK PROTEIN HSP 90-BETA;                            2255:  5n9j-U  4.1  3.5   66   198    5   MOLECULE: MEDIATOR OF RNA POLYMERASE II TRANSCRIPTION SUBUN          2256:  7m96-B  4.1  3.9   55   166    5   MOLECULE: SIGMA INTRACELLULAR RECEPTOR 2;                            2257:  1z0p-A  4.1  2.9   56    73    7   MOLECULE: HYPOTHETICAL PROTEIN SPY1572;                              2258:  6o58-A  4.1  4.9   77   273    4   MOLECULE: CALCIUM UNIPORTER PROTEIN, MITOCHONDRIAL;                  2259:  5wjt-L  4.1  2.4   84   302    2   MOLECULE: FLAGELLIN;                                                 2260:  5wjt-b  4.1  2.4   84   302    2   MOLECULE: FLAGELLIN;                                                 2261:  5ip0-O  4.1  3.8   68    90    7   MOLECULE: PHA GRANULE-ASSOCIATED PROTEIN;                            2262:  5wjt-J  4.1  2.4   84   302    2   MOLECULE: FLAGELLIN;                                                 2263:  5x5b-B  4.1  3.8   70  1053    6   MOLECULE: SPIKE GLYCOPROTEIN;                                        2264:  5wjt-C  4.1  2.4   84   302    2   MOLECULE: FLAGELLIN;                                                 2265:  5wjt-H  4.1  2.4   84   302    2   MOLECULE: FLAGELLIN;                                                 2266:  6vfk-B  4.1  4.6   57   277    7   MOLECULE: T33_DN10B;                                                 2267:  5wjt-G  4.1  2.4   84   302    2   MOLECULE: FLAGELLIN;                                                 2268:  5wjt-X  4.1  2.4   84   302    2   MOLECULE: FLAGELLIN;                                                 2269:  8h0x-B  4.1  3.6   68  1039    4   MOLECULE: SPIKE GLYCOPROTEIN;                                        2270:  5yew-A  4.1  3.5   65   390    9   MOLECULE: MITOFUSIN-1,MITOFUSIN-1 FUSION PROTEIN;                    2271:  5wjt-Q  4.1  2.4   84   302    2   MOLECULE: FLAGELLIN;                                                 2272:  5wjt-d  4.1  2.4   84   302    2   MOLECULE: FLAGELLIN;                                                 2273:  6bpe-A  4.1  2.0   67   299    3   MOLECULE: RETICULOCYTE BINDING PROTEIN 2, PUTATIVE;                  2274:  5wjt-M  4.1  2.4   84   302    2   MOLECULE: FLAGELLIN;                                                 2275:  5ip0-E  4.1  6.6   72   107   10   MOLECULE: PHA GRANULE-ASSOCIATED PROTEIN;                            2276:  5wjt-I  4.1  2.4   84   302    2   MOLECULE: FLAGELLIN;                                                 2277:  5wjt-Z  4.1  2.4   84   302    2   MOLECULE: FLAGELLIN;                                                 2278:  1wp7-C  4.1  1.9   54    61    9   MOLECULE: FUSION PROTEIN;                                            2279:  5wjt-E  4.1  2.4   84   302    2   MOLECULE: FLAGELLIN;                                                 2280:  5wjt-V  4.1  2.4   84   302    2   MOLECULE: FLAGELLIN;                                                 2281:  8cvi-J  4.1  2.9   82   269   12   MOLECULE: FLAGELLIN;                                                 2282:  3jcf-A  4.0  2.8   74   349   12   MOLECULE: MAGNESIUM TRANSPORT PROTEIN CORA;                          2283:  5lnc-B  4.0  4.2   84   367   10   MOLECULE: VACUOLAR TRANSPORTER CHAPERONE 4,CORE HISTONE MAC          2284:  6r7f-E  4.0  4.6   80   311    8   MOLECULE: COP9 SIGNALOSOME COMPLEX SUBUNIT 1;                        2285:  8w8q-C  4.0  3.2   68   287    6   MOLECULE: SOLUBLE CYTOCHROME B562,PROBABLE G-PROTEIN COUPLE          2286:  7nh9-A  4.0  5.6   63   327    5   MOLECULE: CMAX PROTEIN;                                              2287:  5mx5-L  4.0  2.7   69   214    6   MOLECULE: PROTEASOME ACTIVATOR COMPLEX SUBUNIT 1;                    2288:  6xz6-A  4.0  3.3   81   213    7   MOLECULE: GARP DOMAIN-CONTAINING PROTEIN;                            2289:  8wcn-A  4.0  3.4   65   379    8   MOLECULE: DIGUANYLATE CYCLASE;                                       2290:  8age-H  4.0  4.4   65   133    6   MOLECULE: DOLICHYL-DIPHOSPHOOLIGOSACCHARIDE--PROTEIN                 2291:  7n15-A  4.0  9.2   74   520    4   MOLECULE: CYCLIC NUCLEOTIDE-GATED CATION CHANNEL;                    2292:  3aai-A  4.0  5.0   61    78    5   MOLECULE: COPPER HOMEOSTASIS OPERON REGULATORY PROTEIN;              2293:  6vxm-A  4.0  4.3   56   277    4   MOLECULE: MECHANOSENSITIVE ION CHANNEL PROTEIN 1, MITOCHOND          2294:  3vp8-B  4.0  1.9   50    80   16   MOLECULE: GENERAL TRANSCRIPTIONAL COREPRESSOR TUP1;                  2295:  6lcp-B  4.0  3.2   69   376    9   MOLECULE: PHOSPHOLIPID-TRANSPORTING ATPASE;                          2296:  1wa8-B  4.0  4.8   71    95   11   MOLECULE: ESAT-6 LIKE PROTEIN ESXB;                                  2297:  2zet-C  4.0  3.1   75   141   11   MOLECULE: RAS-RELATED PROTEIN RAB-27B;                               2298:  8egr-G  4.0  3.1   68   313    3   MOLECULE: GP15, RECEPTOR-BINDING PROTEIN, TAIL FIBER;                2299:  6wc3-B  4.0  2.6   61    94    3   MOLECULE: PROTEIN TRANSPORT PROTEIN TIP20;                           2300:  6zyd-A  4.0  4.7   59   323    5   MOLECULE: LOW CONDUCTANCE MECHANOSENSITIVE CHANNEL YNAI,LOW          2301:  3b8m-A  4.0  4.2   69   255   12   MOLECULE: FERRIC ENTEROBACTIN (ENTEROCHELIN) TRANSPORT;              2302:  7s9y-A  4.0  2.7   67   901    4   MOLECULE: CYTOCHROME C BIOGENESIS PROTEIN;                           2303:  8z1e-A  4.0  2.5   62   278    3   MOLECULE: UNCHARACTERIZED PROTEIN UL78;                              2304:  6r0z-I  4.0  2.2   49   103   10   MOLECULE: V-TYPE ATP SYNTHASE ALPHA CHAIN;                           2305:  8a3t-Q  4.0  3.2   75   623    7   MOLECULE: ANAPHASE-PROMOTING COMPLEX SUBUNIT CDC27;                  2306:  6tpn-A  4.0  5.3   64   510    9   MOLECULE: OREXIN RECEPTOR TYPE 2,GLGA GLYCOGEN SYNTHASE,ORE          2307:  7o4i-1  4.0  3.5   60   522   13   MOLECULE: GENERAL TRANSCRIPTION AND DNA REPAIR FACTOR IIH H          2308:  8j8j-A  4.0  2.2   59   290    5   MOLECULE: DECAPRENYL-PHOSPHATE PHOSPHORIBOSYLTRANSFERASE;            2309:  6tj1-C  4.0  3.0   63    74    5   MOLECULE: DE NOVO DESIGNED WSHC6;                                    2310:  2d1l-A  4.0  4.4   83   249   10   MOLECULE: METASTASIS SUPPRESSOR PROTEIN 1;                           2311:  7pi6-A  4.0  5.6   80   221    8   MOLECULE: 65 KDA INVARIANT SURFACE GLYCOPROTEIN;                     2312:  6bbf-S  4.0  2.0   55   150   13   MOLECULE: CALCIUM RELEASE-ACTIVATED CALCIUM CHANNEL PROTEIN          2313:  6tms-I  4.0  2.3   59    69    5   MOLECULE: A NOVEL DESIGNED PORE PROTEIN;                             2314:  7cg0-k  4.0  3.3   66   108    9   MOLECULE: FLAGELLAR MS RING L2;                                      2315:  8etj-h  4.0  2.7   56   121   13   MOLECULE: RNA (1758-MER);                                            2316:  6j9r-A  4.0  2.4   59   125    3   MOLECULE: BRAIN TUMOR PROTEIN;                                       2317:  4nb5-A  4.0  4.4   62   145    5   MOLECULE: DNA BINDING PROTEIN;                                       2318:  1nu7-D  4.0  5.6   75   282    3   MOLECULE: THROMBIN LIGHT CHAIN;                                      2319:  3sjc-C  4.0  2.2   48    54   13   MOLECULE: ATPASE GET3;                                               2320:  6nmi-C  4.0  2.9   64   408    2   MOLECULE: GENERAL TRANSCRIPTION AND DNA REPAIR FACTOR IIH H          2321:  3sjc-H  4.0  2.2   48    54   13   MOLECULE: ATPASE GET3;                                               2322:  5d57-E  4.0  3.1   61    89   11   MOLECULE: DIACYLGLYCEROL KINASE;                                     2323:  6ijz-A  4.0  3.5   67   683    6   MOLECULE: CALCIUM PERMEABLE STRESS-GATED CATION CHANNEL 1;           2324:  8bbe-D  4.0  2.5   54   648    9   MOLECULE: INTRAFLAGELLAR TRANSPORT PROTEIN 122 HOMOLOG;              2325:  4tql-B  4.0  4.0   76   235    9   MOLECULE: THREE HELIX BUNDLE;                                        2326:  6xtx-A  4.0  2.7   63   196    6   MOLECULE: DNA REPLICATION LICENSING FACTOR MCM2;                     2327:  3hah-A  4.0  2.5   78   269   12   MOLECULE: HUMAN PACSIN1 F-BAR;                                       2328:  6igk-A  4.0  3.6   73   471    4   MOLECULE: ENDOTHELIN RECEPTOR TYPE B,ENDOLYSIN,ENDOTHELIN R          2329:  5t4q-M  4.0  4.2   69    75    6   MOLECULE: ATP SYNTHASE SUBUNIT ALPHA;                                2330:  7zr6-C  4.0  2.5   72   277    8   MOLECULE: HEAT SHOCK PROTEIN HSP 90-BETA;                            2331:  2q0o-D  4.0  4.0   68    87    6   MOLECULE: PROBABLE TRANSCRIPTIONAL ACTIVATOR PROTEIN TRAR;           2332:  8ftx-A  4.0  2.6   60   214    7   MOLECULE: FLAGELLA BIOSYNTHESIS CHAPERONE FLGN, FLAGELLAR F          2333:  1wp7-A  4.0  2.2   55    64   13   MOLECULE: FUSION PROTEIN;                                            2334:  5uaj-C  4.0  4.2   68  1342   13   MOLECULE: DNA-DIRECTED RNA POLYMERASE SUBUNIT ALPHA;                 2335:  5wwl-M  4.0  5.1   69   214    7   MOLECULE: CENTROMERE PROTEIN MIS12;                                  2336:  5cwp-A  4.0  2.6   54   229    9   MOLECULE: DESIGNED HELICAL REPEAT PROTEIN;                           2337:  7emf-J  4.0  3.3   53   122   17   MOLECULE: MEDIATOR OF RNA POLYMERASE II TRANSCRIPTION SUBUN          2338:  6xl0-N  4.0  2.9   82   271   10   MOLECULE: FLAGELLIN;                                                 2339:  4i1m-A  4.0  4.2   62   285    8   MOLECULE: LEPB;                                                      2340:  2r9r-B  4.0  7.0   69   386    9   MOLECULE: VOLTAGE-GATED POTASSIUM CHANNEL SUBUNIT BETA-2;            2341:  4wd7-B  4.0  4.2   75   270    8   MOLECULE: BESTROPHIN DOMAIN PROTEIN;                                 2342:  5a6e-B  4.0  3.5   63    94    5   MOLECULE: S1-S4 DOMAIN OF POTASSIUM CHANNEL SUBFAMILY T MEM          2343:  6zkn-A  4.0  2.9   57   110    9   MOLECULE: NADH DEHYDROGENASE [UBIQUINONE] FLAVOPROTEIN 1,            2344:  4p1w-C  4.0  3.9   82   399   10   MOLECULE: ATG29;                                                     2345:  7nvr-b  4.0  2.8   55   136    5   MOLECULE: TFIIH BASAL TRANSCRIPTION FACTOR COMPLEX HELICASE          2346:  6xty-A  4.0  2.9   63   196    3   MOLECULE: DNA REPLICATION LICENSING FACTOR MCM2;                     2347:  6zkm-A  4.0  2.9   57   110    9   MOLECULE: NADH DEHYDROGENASE [UBIQUINONE] FLAVOPROTEIN 1,            2348:  2a01-B  4.0  3.9   74   243    8   MOLECULE: APOLIPOPROTEIN A-I;                                        2349:  4ke2-B  4.0  5.6   82   196    5   MOLECULE: TYPE I HYPERACTIVE ANTIFREEZE PROTEIN;                     2350:  6wdn-E  4.0  7.3   81   174   11   MOLECULE: CALCIUM UPTAKE PROTEIN 2, MITOCHONDRIAL;                   2351:  5jhf-C  4.0  5.0   80   404    9   MOLECULE: KLTH0D11660P;                                              2352:  5iy5-O  4.0  5.5   63   227   11   MOLECULE: CYTOCHROME C OXIDASE SUBUNIT 1;                            2353:  7k7k-a  4.0  3.8   80   171    3   MOLECULE: TRANSLOCON ESPA;                                           2354:  4wbd-A  4.0  6.4   79   540   11   MOLECULE: BSHC;                                                      2355:  7xnj-B  4.0  3.0   60    69    5   MOLECULE: STRESS RESPONSE FACILITATOR A, SRFA;                       2356:  3sja-G  4.0  2.4   51    61    2   MOLECULE: ATPASE GET3;                                               2357:  8rgt-L  4.0  2.2   54   606    4   MOLECULE: NADH DEHYDROGENASE [UBIQUINONE] IRON-SULFUR PROTE          2358:  3kdq-C  4.0  6.8   76   153    5   MOLECULE: UNCHARACTERIZED CONSERVED PROTEIN;                         2359:  5u0p-D  4.0  1.8   48    96    0   MOLECULE: MEDIATOR COMPLEX SUBUNIT 14;                               2360:  7kal-D  4.0  3.5   62   475    5   MOLECULE: PROTEIN TRANSPORT CHANNEL SEC61 COMPLEX, ALPHA SU          2361:  6s0k-Z  4.0  2.8   53    62    9   MOLECULE: TRNA-CCA;                                                  2362:  5h5v-C  4.0  3.0   71   356    4   MOLECULE: FLAGELLAR HOOK-ASSOCIATED PROTEIN 2;                       2363:  8c2s-L  4.0  2.2   54   606    4   MOLECULE: NADH-UBIQUINONE OXIDOREDUCTASE CHAIN 3;                    2364:  5nmo-B  4.0  5.0   76   136    8   MOLECULE: CHROMOSOME PARTITION PROTEIN SMC,CHROMOSOME PARTI          2365:  6bhp-A  4.0  2.7   80   170    8   MOLECULE: MEMBRANE PROTEIN;                                          2366:  6g8z-B  4.0  4.5   70   318   10   MOLECULE: VOLUME-REGULATED ANION CHANNEL SUBUNIT LRRC8A;             2367:  7xge-A  4.0  4.9   77   168   10   MOLECULE: BCL-XL AND MCL-1 DUAL BINDER 2;                            2368:  8q4h-C  4.0  3.6   58    89    2   MOLECULE: TETRACHLOROETHENE REDUCTIVE DEHALOGENASE;                  2369:  6rhz-A  4.0  3.8   64   739    5   MOLECULE: CHLOROPHYLL A-B BINDING PROTEIN, CHLOROPLASTIC;            2370:  4by9-L  4.0  3.1   69   366    6   MOLECULE: SSR26;                                                     2371:  8szz-o  4.0  2.4   60    70   12   MOLECULE: O32-ZL4 COMPONENT A;                                       2372:  8e0m-C  4.0  2.4   58   164   14   MOLECULE: BGL15;                                                     2373:  8szz-p  4.0  2.3   58    70   10   MOLECULE: O32-ZL4 COMPONENT A;                                       2374:  8szz-S  4.0  2.4   60    70   12   MOLECULE: O32-ZL4 COMPONENT A;                                       2375:  8szz-y  4.0  2.3   58    70   10   MOLECULE: O32-ZL4 COMPONENT A;                                       2376:  8szz-X  4.0  2.3   58    70   10   MOLECULE: O32-ZL4 COMPONENT A;                                       2377:  8szz-V  4.0  2.5   60    70   12   MOLECULE: O32-ZL4 COMPONENT A;                                       2378:  6vqc-f  4.0  2.7   59    84   10   MOLECULE: ATPASE H+-TRANSPORTING V1 SUBUNIT D;                       2379:  6grk-A  4.0  2.4   82   354    2   MOLECULE: AHLB;                                                      2380:  5eg1-B  4.0  4.1   82   576   12   MOLECULE: MICROCIN-J25 EXPORT ATP-BINDING/PERMEASE PROTEIN           2381:  1orq-C  4.0  3.4   65   223    3   MOLECULE: 6E1 FAB LIGHT CHAIN;                                       2382:  1u5p-A  4.0  2.5   68   211    7   MOLECULE: SPECTRIN ALPHA CHAIN, BRAIN;                               2383:  2w6g-G  4.0  3.1   79   140    6   MOLECULE: ATP SYNTHASE SUBUNIT ALPHA HEART ISOFORM, MITOCHO          2384:  5f7t-H  4.0  2.6   67   133   12   MOLECULE: TRIPARTITE MOTIF-CONTAINING PROTEIN 5,SERINE--TRN          2385:  3b2e-G  4.0  2.7   58    67    2   MOLECULE: ATPASE GET3;                                               2386:  8ovw-Q  4.0  5.6   65   258    5   MOLECULE: CENTROMERE-BINDING PROTEIN 1;                              2387:  6d80-I  4.0  6.8   67    81    0   MOLECULE: SAPOSIN A;                                                 2388:  5z7q-A  4.0  2.9   74   176    5   MOLECULE: FLAGELLIN;                                                 2389:  2z0v-A  4.0  2.4   73   223   11   MOLECULE: SH3-CONTAINING GRB2-LIKE PROTEIN 3;                        2390:  1ucu-A  4.0  3.8   67   494    3   MOLECULE: PHASE 1 FLAGELLIN;                                         2391:  8cqo-A  4.0  2.2   69   187    7   MOLECULE: LIPOPROTEIN, PUTATIVE;                                     2392:  3nmd-A  3.9  1.4   49    53   10   MOLECULE: CGMP DEPENDENT PROTEIN KINASE;                             2393:  7zw1-A  3.9  3.1   82   317    6   MOLECULE: PERIPHERIN-2;                                              2394:  6az6-A  3.9  5.3   52   209   17   MOLECULE: GNTR FAMILY TRANSCRIPTIONAL REGULATOR;                     2395:  8ugc-A  3.9  5.1   58   384    5   MOLECULE: FD15;                                                      2396:  7yqh-B  3.9  3.0   85  1066    6   MOLECULE: STRUCTURAL MAINTENANCE OF CHROMOSOMES PROTEIN 5;           2397:  8f7n-A  3.9  3.6   82   364   13   MOLECULE: METHYL-ACCEPTING CHEMOTAXIS PROTEIN;                       2398:  8k0b-A  3.9  5.2   61   689    7   MOLECULE: CALCIUM PERMEABLE STRESS-GATED CATION CHANNEL 1;           2399:  7dtl-A  3.9  2.9   52    61    8   MOLECULE: PSK;                                                       2400:  6ikn-A  3.9  3.4   80   305    5   MOLECULE: GROWTH ARREST-SPECIFIC PROTEIN 7;                          2401:  4akk-A  3.9  3.7   70   368   10   MOLECULE: NITRATE REGULATORY PROTEIN;                                2402:  3cf4-A  3.9  3.6   67   766   10   MOLECULE: ACETYL-COA DECARBOXYLASE/SYNTHASE ALPHA SUBUNIT;           2403:  5b52-B  3.9  2.3   55    60    9   MOLECULE: H-NS FAMILY PROTEIN MVAT;                                  2404:  8c8g-B  3.9  4.0   66  1195    6   MOLECULE: PUTATIVE BOTULINUM-LIKE TOXIN WO;                          2405:  7dgx-B  3.9  3.0   49    50   14   MOLECULE: CORONIN;                                                   2406:  6c5w-A  3.9  3.5   61   285   10   MOLECULE: CALCIUM UNIPORTER;                                         2407:  7ltq-A  3.9  1.9   63   256    6   MOLECULE: HISTIDINE KINASE;                                          2408:  7lby-A  3.9  3.1   58   755    7   MOLECULE: CELLULOSE SYNTHASE CATALYTIC SUBUNIT [UDP-FORMING          2409:  8owi-A  3.9  2.1   50    55    6   MOLECULE: CENTROMERE-ASSOCIATED PROTEIN E;                           2410:  3jac-A  3.9  3.8   75   918    0   MOLECULE: PIEZO-TYPE MECHANOSENSITIVE ION CHANNEL COMPONENT          2411:  1n2d-C  3.9  1.9   48    48    6   MOLECULE: MYOSIN LIGHT CHAIN;                                        2412:  6lw5-A  3.9  3.4   68   427    4   MOLECULE: SOLUBLE CYTOCHROME B562,N-FORMYL PEPTIDE RECEPTOR          2413:  6spb-Y  3.9  2.6   50    60    8   MOLECULE: 23S RIBOSOMAL RNA;                                         2414:  8j80-A  3.9  3.7   54   290   11   MOLECULE: ZINC TRANSPORTER 7;                                        2415:  6urt-A  3.9  4.4   62   331    8   MOLECULE: LOW CONDUCTANCE MECHANOSENSITIVE CHANNEL YNAI;             2416:  8fhc-B  3.9  5.0   61   215    7   MOLECULE: TRNA-(MS[2]IO[6]A)-HYDROXYLASE;                            2417:  2z5h-A  3.9  1.9   49    49    4   MOLECULE: GENERAL CONTROL PROTEIN GCN4 AND TROPOMYOSIN ALPH          2418:  7z12-A  3.9  2.5   65  1853    2   MOLECULE: PAM1.4, HEAVY CHAIN;                                       2419:  7w72-A  3.9  4.0   61   588    5   MOLECULE: PHOSPHATIDYLINOSITOL GLYCAN ANCHOR BIOSYNTHESIS C          2420:  2p7n-A  3.9  3.6   76   335    5   MOLECULE: PATHOGENICITY ISLAND 1 EFFECTOR PROTEIN;                   2421:  7rut-B  3.9  3.7   55   187    7   MOLECULE: CORRINOID ADENOSYLTRANSFERASE;                             2422:  7xp4-R  3.9  2.5   57   299    7   MOLECULE: GUANINE NUCLEOTIDE-BINDING PROTEIN G(T) SUBUNIT A          2423:  5yfp-B  3.9  4.5   78   927    6   MOLECULE: EXOCYST COMPLEX COMPONENT SEC3;                            2424:  6tpk-A  3.9  4.4   64   461    6   MOLECULE: OXYTOCIN RECEPTOR;                                         2425:  6wlw-T  3.9  3.6   65    85    9   MOLECULE: V-TYPE PROTON ATPASE 21 KDA PROTEOLIPID SUBUNIT;           2426:  2f43-G  3.9  3.7   73   124    5   MOLECULE: ATP SYNTHASE ALPHA CHAIN, MITOCHONDRIAL;                   2427:  4kis-A  3.9  2.1   51   315    4   MOLECULE: PUTATIVE INTEGRASE [BACTERIOPHAGE A118];                   2428:  8idb-D  3.9  6.0   81   295    7   MOLECULE: CELL DIVISION ATP-BINDING PROTEIN FTSE;                    2429:  7ubz-D  3.9  3.2   70   202    4   MOLECULE: ANKYRIN REPEAT DOMAIN-CONTAINING PROTEIN;                  2430:  6v7u-A  3.9  2.0   52    63    4   MOLECULE: QUORUM SENSING ANTI-ACTIVATOR PROTEIN AQS1;                2431:  8edv-A  3.9  1.8   48   333    8   MOLECULE: MITOGUARDIN HOMOLOG;                                       2432:  1tjl-A  3.9  3.3   73   145   10   MOLECULE: DNAK SUPPRESSOR PROTEIN;                                   2433:  5msj-a  3.9  3.1   82   209    9   MOLECULE: PROTEASOME ACTIVATOR COMPLEX SUBUNIT 1;                    2434:  4xwj-A  3.9  3.2   66   153    5   MOLECULE: REGULATOR OF SIGMA D;                                      2435:  5ebz-B  3.9  2.5   84   655    7   MOLECULE: INHIBITOR OF NUCLEAR FACTOR KAPPA-B KINASE SUBUNI          2436:  4j9z-B  3.9  6.2   68    97   10   MOLECULE: SMALL CONDUCTANCE CALCIUM-ACTIVATED POTASSIUM CHA          2437:  8eup-b  3.9  3.8   59   151    8   MOLECULE: RNA (1422-MER);                                            2438:  2hld-Y  3.9  2.7   70   200    3   MOLECULE: ATP SYNTHASE ALPHA CHAIN, MITOCHONDRIAL;                   2439:  5ynx-B  3.9  2.6   62   117   13   MOLECULE: ALLERGEN DER F 21;                                         2440:  6vi4-A  3.9  4.3   73   283    3   MOLECULE: KAPPA OPIOID RECEPTOR;                                     2441:  6m5u-B  3.9  3.6   59   670    7   MOLECULE: TRIPARTITE TERMINASE SUBUNIT 3;                            2442:  8keg-B  3.9  3.1   60   132    7   MOLECULE: NECK GP5;                                                  2443:  5mnt-A  3.9  3.7   73   420    8   MOLECULE: A2 MATURATION PROTEIN;                                     2444:  4gnk-E  3.9  5.6   84   235   10   MOLECULE: GUANINE NUCLEOTIDE-BINDING PROTEIN G(Q) SUBUNIT A          2445:  8keg-D  3.9  3.4   62   132    8   MOLECULE: NECK GP5;                                                  2446:  6rdq-7  3.9  5.8   73   176   11   MOLECULE: ASA-10: POLYTOMELLA F-ATP SYNTHASE ASSOCIATED SUB          2447:  3lf9-A  3.9  5.4   73   120    3   MOLECULE: 4E10_D0_1IS1A_001_C (T161);                                2448:  6rdr-7  3.9  5.8   73   176   11   MOLECULE: ASA-10: POLYTOMELLA F-ATP SYNTHASE ASSOCIATED SUB          2449:  5t59-A  3.9  4.6   67   114    6   MOLECULE: KLLA0F02343P;                                              2450:  5f3w-B  3.9  2.1   61   359    5   MOLECULE: DNA DOUBLE-STRAND BREAK REPAIR PROTEIN MRE11;              2451:  4x5t-A  3.9  4.2   62   308    5   MOLECULE: PROTON-GATED ION CHANNEL,GLRA1 PROTEIN,GLRA1 PROT          2452:  2x2e-A  3.9  4.3   61   341    8   MOLECULE: DYNAMIN-1;                                                 2453:  2v4d-C  3.9  2.9   62   327   11   MOLECULE: MULTIDRUG RESISTANCE PROTEIN MEXA;                         2454:  5mlc-9  3.9  5.7   69   107    6   MOLECULE: 23S RIBOSOMAL RNA, CHLOROPLASTIC;                          2455:  2hjd-D  3.9  4.4   66    90   14   MOLECULE: QUORUM-SENSING ANTIACTIVATOR;                              2456:  7ytj-A  3.9  4.7   66   119    6   MOLECULE: VACUOLAR TRANSPORTER CHAPERONE 4;                          2457:  3htk-C  3.9  3.1   63   254    3   MOLECULE: STRUCTURAL MAINTENANCE OF CHROMOSOMES PROTEIN 5;           2458:  7dtl-B  3.9  2.9   52    61    8   MOLECULE: PSK;                                                       2459:  7drt-B  3.9  4.1   72   496    6   MOLECULE: PROTEIN WNT-3A;                                            2460:  3rx6-A  3.9  2.4   71   187   11   MOLECULE: POLARITY SUPPRESSION PROTEIN;                              2461:  3sjq-D  3.9  2.3   61    78    8   MOLECULE: CALMODULIN;                                                2462:  5vr2-D  3.9  2.9   47    50    0   MOLECULE: MYOCILIN;                                                  2463:  6o6c-A  3.9  4.4   58  1446    3   MOLECULE: DNA-DIRECTED RNA POLYMERASE II SUBUNIT RPB1;               2464:  7m2w-G  3.9  5.4   84   712    2   MOLECULE: TUBULIN GAMMA CHAIN;                                       2465:  4d8m-A  3.9  3.4   71   585    6   MOLECULE: PESTICIDAL CRYSTAL PROTEIN CRY5BA;                         2466:  1i6z-A  3.9  2.1   63   135   10   MOLECULE: BAG-FAMILY MOLECULAR CHAPERONE REGULATOR-1;                2467:  6o7x-M  3.9  3.4   83   210   11   MOLECULE: V-TYPE PROTON ATPASE SUBUNIT C;                            2468:  6gcs-5  3.9  4.3   65   639    8   MOLECULE: 75-KDA PROTEIN (NUAM);                                     2469:  2jg7-A  3.9  3.8   56   509   14   MOLECULE: ANTIQUITIN;                                                2470:  8eav-C  3.9  3.9   74   159    0   MOLECULE: YAR027W OR YAR028W;                                        2471:  6vqh-f  3.9  3.0   60    84   10   MOLECULE: ATPASE H+-TRANSPORTING V1 SUBUNIT D;                       2472:  6vq8-f  3.9  3.0   60    84   10   MOLECULE: ATPASE H+-TRANSPORTING V1 SUBUNIT A;                       2473:  5tin-D  3.9  4.3   65   344    6   MOLECULE: GLYCINE RECEPTOR SUBUNIT ALPHA-3;                          2474:  4he8-A  3.9  2.6   55    92    5   MOLECULE: NADH-QUINONE OXIDOREDUCTASE SUBUNIT 7;                     2475:  3sjb-C  3.9  2.8   54    63    2   MOLECULE: ATPASE GET3;                                               2476:  5j1h-A  3.9  3.0   71   183    6   MOLECULE: PLECTIN,PLECTIN;                                           2477:  7q21-H  3.9  2.7   50   125    2   MOLECULE: CO-PURIFIED UNKNOWN TRANSMEMBRANE HELICES BUILT A          2478:  6nd4-P  3.9  3.3   57   359    7   MOLECULE: ETS RRNA;                                                  2479:  8tn1-A  3.9  2.6   66   147    5   MOLECULE: DE NOVO DESIGNED 4 HELIX BUNDLES;                          2480:  7khw-I  3.9  3.3   72   174    7   MOLECULE: TRANSLOCON ESPA;                                           2481:  4fye-A  3.9 19.4   81   725    7   MOLECULE: SIDF, INHIBITOR OF GROWTH FAMILY, MEMBER 3;                2482:  8ioi-B  3.9  3.8   70   181   10   MOLECULE: PADR FAMILY TRANSCRIPTIONAL REGULATOR;                     2483:  7rit-A  3.9  3.3   73   569    3   MOLECULE: ATP-DEPENDENT LIPID A-CORE FLIPPASE;                       2484:  4hw9-A  3.9  4.5   56   253    7   MOLECULE: MECHANOSENSITIVE CHANNEL MSCS;                             2485:  2elb-A  3.9  4.8   82   351    1   MOLECULE: ADAPTER PROTEIN CONTAINING PH DOMAIN, PTB DOMAIN           2486:  2jbs-C  3.9  3.4   67   400    6   MOLECULE: P-HYDROXYPHENYLACETATE HYDROXYLASE C2\:OXYGENASE           2487:  3g80-A  3.9  3.0   58    73    7   MOLECULE: PROTEIN B2;                                                2488:  2mme-A  3.9  3.1   64    83    5   MOLECULE: MXIH;                                                      2489:  6s7o-D  3.9  4.1   61   110    5   MOLECULE: DOLICHYL-DIPHOSPHOOLIGOSACCHARIDE--PROTEIN                 2490:  6z6e-A  3.9  3.3   63   104    5   MOLECULE: TERMINASE SMALL SUBUNIT;                                   2491:  6w08-B  3.9  3.6   67   356    6   MOLECULE: MOTILITY ASSOCIATED KILLING FACTOR E;                      2492:  5cwo-A  3.9  3.1   57   219   12   MOLECULE: DESIGNED HELICAL REPEAT PROTEIN;                           2493:  8v3b-a  3.9  3.0   57   416    5   MOLECULE: O43_129_+4 COMPONENT A;                                    2494:  4dvd-A  3.9  2.3   70   187   11   MOLECULE: POLARITY SUPPRESSION PROTEIN;                              2495:  5ibl-E  3.9  6.1   70   115    3   MOLECULE: HEMAGGLUTININ;                                             2496:  3spg-A  3.9  5.2   78   332   13   MOLECULE: INWARD-RECTIFIER K+ CHANNEL KIR2.2;                        2497:  8eat-A  3.9  3.8   71   130    4   MOLECULE: VACUOLAR ATPASE ASSEMBLY PROTEIN VMA22;                    2498:  5tqy-B  3.9  4.1   76   578    5   MOLECULE: INHIBITOR OF NUCLEAR FACTOR KAPPA-B KINASE SUBUNI          2499:  8d06-A  3.9  2.7   54    64    4   MOLECULE: HALC3_104;                                                 2500:  7lm2-B  3.9  3.4   81   169    9   MOLECULE: PHOSPHATIDYLINOSITOL 4,5-BISPHOSPHATE 3-KINASE CA          2501:  5yew-C  3.9  1.6   57   389    2   MOLECULE: MITOFUSIN-1,MITOFUSIN-1 FUSION PROTEIN;                    2502:  4l23-B  3.9  3.5   81   277    9   MOLECULE: PHOSPHATIDYLINOSITOL 4,5-BISPHOSPHATE 3-KINASE CA          2503:  5gnr-A  3.9  1.7   59   387    2   MOLECULE: MITOFUSIN-1;                                               2504:  6cnb-O  3.9  4.1   72   539    6   MOLECULE: DNA-DIRECTED RNA POLYMERASE III SUBUNIT RPC1;              2505:  5x58-C  3.9  4.5   73  1052   14   MOLECULE: SPIKE GLYCOPROTEIN;                                        2506:  8cvi-e  3.9  2.6   82   269    9   MOLECULE: FLAGELLIN;                                                 2507:  8vc1-A  3.8  2.7   67   391    6   MOLECULE: GUSTATORY RECEPTOR;                                        2508:  3din-E  3.8  4.2   59    65    3   MOLECULE: PROTEIN TRANSLOCASE SUBUNIT SECA;                          2509:  3e7k-H  3.8  1.4   50    56   10   MOLECULE: TRPM7 CHANNEL;                                             2510:  6qeq-A  3.8  5.0   63   111    6   MOLECULE: PCFF;                                                      2511:  6c70-A  3.8  6.1   73   388   10   MOLECULE: ODORANT RECEPTOR;                                          2512:  6wqz-A  3.8  6.0   66   536    6   MOLECULE: AUTOPHAGY-RELATED PROTEIN 9A;                              2513:  4zm1-B  3.8  6.5   83   238    8   MOLECULE: CHAIN LENGTH DETERMINANT PROTEIN;                          2514:  6bs9-A  3.8  2.2   55   130   11   MOLECULE: STAGE III SPORULATION PROTEIN AB;                          2515:  8tl7-A  3.8  5.7   57   694    9   MOLECULE: COMPUTATIONALLY DESIGNED PROTEIN;                          2516:  7wb4-b  3.8  4.5   55   636    9   MOLECULE: OUTER NUP133;                                              2517:  4hkr-A  3.8  3.7   72   165   10   MOLECULE: CALCIUM RELEASE-ACTIVATED CALCIUM CHANNEL PROTEIN          2518:  8ovw-U  3.8  3.2   58   184    0   MOLECULE: CENTROMERE-BINDING PROTEIN 1;                              2519:  8iff-A  3.8  3.0   63   868    8   MOLECULE: PHYTOCHROME A;                                             2520:  5mw9-H  3.8  1.7   50    57   10   MOLECULE: CENTROSOMIN;                                               2521:  5yi4-A  3.8  4.0   62   145    5   MOLECULE: DISRUPTED IN SCHIZOPHRENIA 1 HOMOLOG,NUCLEAR DIST          2522:  3dl8-E  3.8  4.0   58    65    3   MOLECULE: PROTEIN TRANSLOCASE SUBUNIT SECA;                          2523:  5ncn-B  3.8  4.8   66    76    2   MOLECULE: DBF2 KINASE ACTIVATOR PROTEIN MOB1;                        2524:  6y09-C  3.8  2.3   50    86    8   MOLECULE: RAS-RELATED PROTEIN RAB-33B;                               2525:  6xm1-D  3.8  2.6   61   181    7   MOLECULE: VPS45;                                                     2526:  4xvi-A  3.8  6.5   72   647    6   MOLECULE: DNA POLYMERASE NU;                                         2527:  4yv4-C  3.8  2.9   50    57    2   MOLECULE: SPINDLE ASSEMBLY ABNORMAL PROTEIN 5;                       2528:  8x2q-A  3.8  3.3   66   109    2   MOLECULE: ADENOMATOUS POLYPOSIS COLI PROTEIN;                        2529:  3h7x-E  3.8  3.4   50    53   10   MOLECULE: ADHESIN YADA;                                              2530:  7x07-A  3.8  2.0   68   633   10   MOLECULE: ATP-BINDING CASSETTE SUB-FAMILY D MEMBER 1;                2531:  5tgz-A  3.8  4.4   66   439    8   MOLECULE: CANNABINOID RECEPTOR 1,FLAVODOXIN,CANNABINOID REC          2532:  8bh1-D  3.8  1.5   50    85   12   MOLECULE: PROBABLE PEPTIDOGLYCAN GLYCOSYLTRANSFERASE FTSW;           2533:  8e1u-A  3.8  4.0   63  1126    5   MOLECULE: PPI-DEPENDENT PEPCK;                                       2534:  8pnu-A  3.8  4.7   64   155    5   MOLECULE: STYRENE OXIDE ISOMERASE;                                   2535:  8c29-z  3.8  2.4   51    62   10   MOLECULE: PHOTOSYSTEM II PROTEIN D1;                                 2536:  7vg4-B  3.8  3.8   63   206    6   MOLECULE: METHENYLTETRAHYDROFOLATE CYCLOHYDROLASE;                   2537:  6r9t-A  3.8  4.0   65  2185   11   MOLECULE: TALIN-1;                                                   2538:  1go4-G  3.8  3.3   53   100    8   MOLECULE: MITOTIC SPINDLE ASSEMBLY CHECKPOINT PROTEIN MAD2A          2539:  7ug2-A  3.8  1.9   48    49   10   MOLECULE: TRIPARTITE MOTIF-CONTAINING PROTEIN 75;                    2540:  5a5t-A  3.8  6.3   83   599   12   MOLECULE: EUKARYOTIC TRANSLATION INITIATION FACTOR 3 SUBUNI          2541:  3wmi-A  3.8  2.0   50    57    2   MOLECULE: EIAV GP45 WILD TYPE;                                       2542:  1au1-A  3.8  7.0   66   166    8   MOLECULE: INTERFERON-BETA;                                           2543:  6dfk-N  3.8  3.1   78   233    5   MOLECULE: SUBUNIT OF PROTEASEOME ACTIVATOR COMPLEX,PUTATIVE          2544:  3err-A  3.8  3.7   72   527   10   MOLECULE: FUSION PROTEIN OF MICROTUBULE BINDING DOMAIN FROM          2545:  5ebz-E  3.8  2.5   84   655    7   MOLECULE: INHIBITOR OF NUCLEAR FACTOR KAPPA-B KINASE SUBUNI          2546:  5a7d-Q  3.8  4.5   62   281    3   MOLECULE: PINS;                                                      2547:  6yel-A  3.8  2.4   58   115    7   MOLECULE: STROMAL INTERACTION MOLECULE 1;                            2548:  6ey0-B  3.8  2.9   64   148   16   MOLECULE: T9SS COMPONENT CYTOPLASMIC MEMBRANE PROTEIN PORM;          2549:  6r6b-G  3.8  6.5   71    85   15   MOLECULE: SURFACE PRESENTATION OF ANTIGENS PROTEIN SPAP;             2550:  5tin-E  3.8  3.8   61   339    5   MOLECULE: GLYCINE RECEPTOR SUBUNIT ALPHA-3;                          2551:  6rdf-7  3.8  5.2   72   176    7   MOLECULE: ASA-10: POLYTOMELLA F-ATP SYNTHASE ASSOCIATED SUB          2552:  5ir6-A  3.8  6.7   70   432    7   MOLECULE: BD-TYPE QUINOL OXIDASE SUBUNIT I;                          2553:  4wqt-X  3.8  2.8   59   154    7   MOLECULE: DNA-DIRECTED RNA POLYMERASE SUBUNIT ALPHA;                 2554:  6mbz-A  3.8  4.3   79   536   10   MOLECULE: SIGNAL TRANSDUCER AND ACTIVATOR OF TRANSCRIPTION           2555:  4ci6-B  3.8 16.0   65   576    9   MOLECULE: PROTEIN KINASE YOPO;                                       2556:  6bwj-B  3.8  7.5   71   617    6   MOLECULE: TRANSIENT RECEPTOR POTENTIAL CATION CHANNEL SUBFA          2557:  7udb-B  3.8  2.4   65   208    5   MOLECULE: SYNTAXIN-BINDING PROTEIN 1;                                2558:  3iuk-A  3.8  5.3   65   552    8   MOLECULE: UNCHARACTERIZED PROTEIN;                                   2559:  6mgv-A  3.8  4.5   68   634    6   MOLECULE: CALCIUM PERMEABLE STRESS-GATED CATION CHANNEL 1;           2560:  8piv-H  3.8  2.4   62   177    6   MOLECULE: GLUTAMATE RECEPTOR;                                        2561:  5kuk-A  3.8  5.7   76   326    5   MOLECULE: ATP-SENSITIVE INWARD RECTIFIER POTASSIUM CHANNEL           2562:  1w9r-A  3.8  2.8   62   119    3   MOLECULE: CHOLINE BINDING PROTEIN A;                                 2563:  8v2d-Q  3.8  3.0   52   307   13   MOLECULE: O43_129 COMPONENT B;                                       2564:  6kac-q  3.8  1.8   49   148    8   MOLECULE: PHOTOSYSTEM II PROTEIN D1;                                 2565:  3mxl-A  3.8  3.5   66   394    5   MOLECULE: NITROSOSYNTHASE;                                           2566:  5dm8-V  3.8  2.9   54    66   11   MOLECULE: 50S RIBOSOMAL PROTEIN L1;                                  2567:  1c17-M  3.8  6.2   67   142    6   MOLECULE: ATP SYNTHASE SUBUNIT C;                                    2568:  4x0j-B  3.8  2.9   83   262    8   MOLECULE: HAPTOGLOBIN-HEMOGLOBIN RECEPTOR;                           2569:  7phk-B  3.8  5.5   77   392    5   MOLECULE: POTASSIUM VOLTAGE-GATED CHANNEL, SHAW-RELATED SUB          2570:  6zni-O  3.8  3.7   64    82    5   MOLECULE: PROTEIN MXIH;                                              2571:  6zni-E  3.8  3.7   64    82    5   MOLECULE: PROTEIN MXIH;                                              2572:  5nnv-B  3.8  3.1   82   231    9   MOLECULE: CHROMOSOME PARTITION PROTEIN SMC,CHROMOSOME PARTI          2573:  6zni-H  3.8  3.7   64    82    5   MOLECULE: PROTEIN MXIH;                                              2574:  1k4t-A  3.8  4.6   70   565    7   MOLECULE: 5'-D(*AP*AP*AP*AP*AP*GP*AP*CP*TP*T)-3';                    2575:  7khw-d  3.8  3.4   73   174    7   MOLECULE: TRANSLOCON ESPA;                                           2576:  5fjd-B  3.8  2.8   60   111    5   MOLECULE: COPPER STORAGE PROTEIN 1;                                  2577:  2etd-A  3.8  4.1   74   141    3   MOLECULE: LEMA PROTEIN;                                              2578:  5vhx-E  3.8  1.6   64   179   11   MOLECULE: GLUTAMATE RECEPTOR 2,GERM CELL-SPECIFIC GENE 1-LI          2579:  6zni-P  3.8  3.7   64    82    5   MOLECULE: PROTEIN MXIH;                                              2580:  5yij-A  3.8  1.9   57   959    7   MOLECULE: SDEA;                                                      2581:  6xby-a  3.8  2.7   75   494   11   MOLECULE: V-TYPE PROTON ATPASE CATALYTIC SUBUNIT A;                  2582:  6peq-G  3.8  2.6   63   132    6   MOLECULE: GLUTAMATE RECEPTOR 2;                                      2583:  6ygi-A  3.8  3.4   64   148    6   MOLECULE: CAPSID PROTEIN,CAPSID PROTEIN;                             2584:  6ud8-G  3.8  2.8   64   132    6   MOLECULE: GLUTAMATE RECEPTOR 2;                                      2585:  5oxf-C  3.8  3.5   84   601    7   MOLECULE: GTP-BINDING PROTEIN;                                       2586:  1joy-A  3.8  3.6   57    67   11   MOLECULE: PROTEIN (ENVZ_ECOLI);                                      2587:  7phk-C  3.8  5.5   77   392    5   MOLECULE: POTASSIUM VOLTAGE-GATED CHANNEL, SHAW-RELATED SUB          2588:  6b2z-2  3.8  3.0   54    75    6   MOLECULE: ATP SYNTHASE SUBUNIT C, MITOCHONDRIAL;                     2589:  6x4s-B  3.8  4.4   72   151   13   MOLECULE: CALCIUM UNIPORTER PROTEIN,PROTEIN EMRE HOMOLOG,            2590:  7k7k-B  3.8  4.1   77   171    8   MOLECULE: TRANSLOCON ESPA;                                           2591:  7k7k-b  3.8  4.1   77   171    8   MOLECULE: TRANSLOCON ESPA;                                           2592:  2np0-A  3.8  7.0   83  1289    5   MOLECULE: BOTULINUM NEUROTOXIN TYPE B;                               2593:  4or2-B  3.8  3.2   57   366    9   MOLECULE: SOLUBLE CYTOCHROME B562, METABOTROPIC GLUTAMATE R          2594:  7khw-j  3.8  3.4   73   174    7   MOLECULE: TRANSLOCON ESPA;                                           2595:  7khw-c  3.8  3.3   72   174    7   MOLECULE: TRANSLOCON ESPA;                                           2596:  3mxl-B  3.8  3.5   68   370    4   MOLECULE: NITROSOSYNTHASE;                                           2597:  7phk-H  3.8  4.7   73   393    4   MOLECULE: POTASSIUM VOLTAGE-GATED CHANNEL, SHAW-RELATED SUB          2598:  8j7y-A  3.8  2.4   54   280    4   MOLECULE: ZINC TRANSPORTER 7;                                        2599:  2p61-A  3.8  2.6   57   114    4   MOLECULE: HYPOTHETICAL PROTEIN TM_1646;                              2600:  7pg8-T  3.8  4.4   67   130    7   MOLECULE: ANT05 H12 FAB FRAGMENT, LIGHT CHAIN;                       2601:  8j7y-B  3.8  2.4   54   280    4   MOLECULE: ZINC TRANSPORTER 7;                                        2602:  7pg8-Q  3.8  4.1   67   126    7   MOLECULE: ANT05 H12 FAB FRAGMENT, LIGHT CHAIN;                       2603:  7pg8-E  3.8  4.1   67   128    7   MOLECULE: ANT05 H12 FAB FRAGMENT, LIGHT CHAIN;                       2604:  2js5-A  3.8  2.8   60    71   12   MOLECULE: UNCHARACTERIZED PROTEIN;                                   2605:  6rfl-C  3.8  4.4   60   304    5   MOLECULE: DNA-DEPENDENT RNA POLYMERASE SUBUNIT RPO132;               2606:  4clv-A  3.8  4.5   72   142    7   MOLECULE: NICKEL-COBALT-CADMIUM RESISTANCE PROTEIN NCCX;             2607:  3txq-F  3.8  2.2   58    87    7   MOLECULE: TERMINASE DNA PACKAGING ENZYME SMALL SUBUNIT;              2608:  8akr-1  3.8  4.0   80   196    9   MOLECULE: CHLOROPLAST MEMBRANE-ASSOCIATED 30 KD PROTEIN;             2609:  7khw-T  3.8  3.4   73   174    7   MOLECULE: TRANSLOCON ESPA;                                           2610:  6dvu-A  3.8  1.5   47    51    4   MOLECULE: APOLIPOPROTEIN C-I;                                        2611:  5lj5-G  3.8  2.3   51    97    4   MOLECULE: U5 SNRNA (SMALL NUCLEAR RNA);                              2612:  7ymi-z  3.8  2.4   49    59   10   MOLECULE: PHOTOSYSTEM II PROTEIN D1 2;                               2613:  8akr-x  3.8  4.1   80   196    9   MOLECULE: CHLOROPLAST MEMBRANE-ASSOCIATED 30 KD PROTEIN;             2614:  6acj-B  3.8  3.5   68  1065    7   MOLECULE: SPIKE GLYCOPROTEIN;                                        2615:  1zv8-G  3.8  2.4   48    50    6   MOLECULE: E2 GLYCOPROTEIN;                                           2616:  6crx-A  3.8  4.5   74  1066   12   MOLECULE: SPIKE GLYCOPROTEIN,FIBRITIN;                               2617:  1zv8-C  3.8  2.0   46    46    7   MOLECULE: E2 GLYCOPROTEIN;                                           2618:  7sg4-B  3.8  3.6   70  1095    6   MOLECULE: SPIKE GLYCOPROTEIN;                                        2619:  6z6o-G  3.8  3.2   82   548    6   MOLECULE: HISTONE DEACETYLASE HDA1;                                  2620:  1zv8-K  3.8  2.4   48    50    6   MOLECULE: E2 GLYCOPROTEIN;                                           2621:  6nb6-A  3.8  3.7   70  1052    6   MOLECULE: SPIKE GLYCOPROTEIN;                                        2622:  1zv8-E  3.8  2.5   48    49    6   MOLECULE: E2 GLYCOPROTEIN;                                           2623:  6crw-C  3.8  4.2   72  1068   14   MOLECULE: SPIKE GLYCOPROTEIN,FIBRITIN;                               2624:  1x8z-A  3.7  5.4   71   151   10   MOLECULE: INVERTASE/PECTIN METHYLESTERASE INHIBITOR FAMILY           2625:  4cgb-B  3.7  1.4   45    45   16   MOLECULE: ECHINODERM MICROTUBULE-ASSOCIATED PROTEIN-LIKE 2;          2626:  1ifk-A  3.7  1.9   48    51    6   MOLECULE: INOVIRUS;                                                  2627:  3efg-A  3.7  2.8   49    51   18   MOLECULE: PROTEIN SLYX HOMOLOG;                                      2628:  3vn0-B  3.7  3.1   49    49    4   MOLECULE: VOLTAGE-GATED HYDROGEN CHANNEL 1;                          2629:  4m3l-A  3.7  2.1   50    60   10   MOLECULE: E3 UBIQUITIN-PROTEIN LIGASE TRIM63;                        2630:  3fga-D  3.7  1.9   47    47    6   MOLECULE: SERINE/THREONINE-PROTEIN PHOSPHATASE 2A 65 KDA RE          2631:  2x7r-C  3.7  1.6   50    58    8   MOLECULE: TRANSMEMBRANE PROTEIN GP41;                                2632:  6vrb-C  3.7  5.7   59   229   10   MOLECULE: RNA (52-MER);                                              2633:  8jhu-A  3.7  3.3   56   811   11   MOLECULE: LEGIONELLA PNEUMOPHILA EFFECTOR PROTEIN SIDI;              2634:  7jtk-I  3.7  5.6   67   483    7   MOLECULE: FLAGELLAR RADIAL SPOKE PROTEIN 1;                          2635:  6ocf-B  3.7  2.9   48    49    0   MOLECULE: TUBULINYL-TYR CARBOXYPEPTIDASE 1;                          2636:  6cko-C  3.7  2.6   48    50    8   MOLECULE: PROTEIN AF-10;                                             2637:  6m5r-B  3.7  3.9   62   689    6   MOLECULE: TRIPARTITE TERMINASE SUBUNIT 3;                            2638:  6dfp-A  3.7  4.5   85   361   11   MOLECULE: VCA0883;                                                   2639:  6fpr-B  3.7  2.5   50    58   16   MOLECULE: SIGNAL RECOGNITION PARTICLE RECEPTOR FTSY;                 2640:  7tpg-B  3.7  5.9   56   403    5   MOLECULE: PUTATIVE CELL SURFACE POLYSACCHARIDE POLYMERASE/L          2641:  4xyp-A  3.7  2.5   56    70    4   MOLECULE: FUSION PROTEIN;                                            2642:  8ewg-A  3.7  4.0   61  1017   11   MOLECULE: CRISPR-ASSOCIATED ENDONUCLEASE CAS9;                       2643:  8bw9-B  3.7  3.7   56   288    7   MOLECULE: PROTEIN AVEUGLE;                                           2644:  8dtp-A  3.7  3.6   56   432    5   MOLECULE: DNAB-LIKE REPLICATIVE HELICASE;                            2645:  4aqr-D  3.7  1.7   50    57    6   MOLECULE: CALMODULIN-7;                                              2646:  7uic-b  3.7  2.2   53    65    2   MOLECULE: MEDIATOR OF RNA POLYMERASE II TRANSCRIPTION SUBUN          2647:  7vcf-C  3.7  3.3   66   217    6   MOLECULE: TIC214;                                                    2648:  4x3n-A  3.7  3.4   66   259    6   MOLECULE: CALCIUM-REGULATED ACTIN-BUNDLING PROTEIN;                  2649:  6fes-A  3.7  5.8   56   365    5   MOLECULE: D12_BRIC2, A SYNTHETIC PROTEIN,D12_BRIC2, A SYNTH          2650:  7f9l-A  3.7  3.6   59   162    7   MOLECULE: RIFIN;                                                     2651:  3zd0-A  3.7  3.4   65    85    6   MOLECULE: P7 PROTEIN;                                                2652:  6yj6-A  3.7  4.9   56   819    5   MOLECULE: TRANSCRIPTION FACTOR TAU 131 KDA SUBUNIT;                  2653:  6c6l-O  3.7  4.0   60    76    5   MOLECULE: V-TYPE PROTON ATPASE SUBUNIT C';                           2654:  5v2c-b  3.7  5.8   77   506    8   MOLECULE: PHOTOSYSTEM II PROTEIN D1;                                 2655:  7wjt-B  3.7  2.7   50    60    4   MOLECULE: ISOFORM 2 OF TRANSMEMBRANE PROTEIN 266;                    2656:  8b8d-D  3.7  2.7   50   103    6   MOLECULE: PHOSPHOPROTEIN;                                            2657:  8p50-A  3.7  4.7   74  1574    3   MOLECULE: TOXIN PROTEIN;                                             2658:  6od2-A  3.7  1.9   48    53    4   MOLECULE: SPINDLE POLE BODY COMPONENT SPC42;                         2659:  7nyw-A  3.7  3.0   67   685    6   MOLECULE: CHROMOSOME PARTITION PROTEIN MUKB;                         2660:  7ml0-1  3.7  2.7   58   367   12   MOLECULE: DNA-DIRECTED RNA POLYMERASE SUBUNIT;                       2661:  8dfu-0  3.7  2.6   59    84    7   MOLECULE: PILIN PROTEIN;                                             2662:  8q85-U  3.7  1.3   50    95   12   MOLECULE: KINETOCHORE PROTEIN NDC80;                                 2663:  5vox-R  3.7  3.8   68   212    6   MOLECULE: V-TYPE PROTON ATPASE CATALYTIC SUBUNIT A,V-TYPE P          2664:  8i3e-C  3.7  2.7   49    56   10   MOLECULE: ELKS/RAB6-INTERACTING/CAST FAMILY MEMBER 1;                2665:  7c79-D  3.7  4.1   58   251    7   MOLECULE: RIBONUCLEASE MRP RNA SUBUNIT NME1;                         2666:  4gwp-C  3.7  5.2   63   184    8   MOLECULE: MEDIATOR OF RNA POLYMERASE II TRANSCRIPTION SUBUN          2667:  7b6h-A  3.7  2.4   61   614    7   MOLECULE: TRAFFICKING PROTEIN PARTICLE COMPLEX SUBUNIT 11;           2668:  4pac-A  3.7  3.6   61   153    7   MOLECULE: HISTIDINE-CONTAINING PHOSPHOTRANSFER PROTEIN 2;            2669:  5xns-A  3.7  4.8   77   398    9   MOLECULE: CHROMOSOME PARTITION PROTEIN SMC;                          2670:  3a8n-A  3.7  2.5   66   230    5   MOLECULE: T-LYMPHOMA INVASION AND METASTASIS-INDUCING                2671:  8eav-k  3.7  4.1   71   145    0   MOLECULE: YAR027W OR YAR028W;                                        2672:  6s7t-F  3.7  3.7   56   250   11   MOLECULE: DOLICHYL-DIPHOSPHOOLIGOSACCHARIDE--PROTEIN                 2673:  6rwa-B  3.7  2.9   83  2361    5   MOLECULE: TCDA4;                                                     2674:  7qpg-X  3.7  3.2   68   779    7   MOLECULE: PROTEIN ZWILCH HOMOLOG;                                    2675:  6g9l-A  3.7  4.9   71   718    1   MOLECULE: VOLUME-REGULATED ANION CHANNEL SUBUNIT LRRC8A;             2676:  5n5e-c  3.7  5.0   60    98    7   MOLECULE: PFC_05175;                                                 2677:  7pgh-F  3.7  3.5   68   133   12   MOLECULE: ION TRANSPORT PROTEIN,VOLTAGE-GATED SODIUM CHANNE          2678:  6xjv-M  3.7  4.4   72   270    7   MOLECULE: CALCIUM UNIPORTER PROTEIN, MITOCHONDRIAL;                  2679:  5n5e-K  3.7  4.9   60    97    7   MOLECULE: PFC_05175;                                                 2680:  6ysl-E  3.7  3.3   72   255    4   MOLECULE: MOTILITY PROTEIN A;                                        2681:  5n5e-O  3.7  4.9   60    97    7   MOLECULE: PFC_05175;                                                 2682:  5n5e-Q  3.7  4.9   60    98    7   MOLECULE: PFC_05175;                                                 2683:  6bu5-A  3.7  4.6   64   398    6   MOLECULE: DIVALENT METAL CATION TRANSPORTER MNTH;                    2684:  3fd9-B  3.7  3.0   68   239    9   MOLECULE: UNCHARACTERIZED PROTEIN;                                   2685:  5sy1-A  3.7  6.3   80   582    4   MOLECULE: CALMODULIN;                                                2686:  4uxz-E  3.7  3.2   61   100   11   MOLECULE: DIACYLGLYCEROL KINASE-DELTA 7;                             2687:  6uuj-J  3.7  3.1   62    71   10   MOLECULE: PE FAMILY IMMUNOMODULATOR PE5;                             2688:  6xky-B  3.7  2.4   82   271    7   MOLECULE: FLAGELLIN;                                                 2689:  4xy3-A  3.7  3.3   79   243    6   MOLECULE: ESX-1 SECRETION-ASSOCIATED PROTEIN ESPB;                   2690:  6xky-N  3.7  2.4   82   271    7   MOLECULE: FLAGELLIN;                                                 2691:  5n5e-M  3.7  4.9   60    98    7   MOLECULE: PFC_05175;                                                 2692:  4zh3-C  3.7  5.5   74  1340    8   MOLECULE: DNA-DIRECTED RNA POLYMERASE SUBUNIT ALPHA;                 2693:  7nd2-B  3.7  2.9   74   330    9   MOLECULE: PROTEIN PHOSPHATASE 1 REGULATORY SUBUNIT 21;               2694:  7s9z-A  3.7  3.5   75   890   12   MOLECULE: CYTOCHROME C BIOGENESIS PROTEIN;                           2695:  1h2s-B  3.7  3.3   51    60   10   MOLECULE: SENSORY RHODOPSIN II;                                      2696:  8j0k-A  3.7  3.7   64   213    6   MOLECULE: TRANSCRIPTION FACTOR AP-2-ALPHA;                           2697:  6s1k-A  3.7  4.1   67   383   16   MOLECULE: CHEMOTAXIS PROTEIN CHEA;                                   2698:  8tn6-A  3.7  3.0   71   147    7   MOLECULE: DE NOVO DESIGNED PROTEIN;                                  2699:  8g9k-B  3.7  2.9   51   201    8   MOLECULE: THR2;                                                      2700:  7khr-H  3.7  2.7   82   212   12   MOLECULE: V-TYPE PROTON ATPASE CATALYTIC SUBUNIT A;                  2701:  6zni-A  3.7  3.7   64    82    5   MOLECULE: PROTEIN MXIH;                                              2702:  6zni-T  3.7  3.7   64    82    5   MOLECULE: PROTEIN MXIH;                                              2703:  8ap9-H  3.7  3.9   51   161    8   MOLECULE: ATP SYNTHASE GAMMA SUBUNIT;                                2704:  6blj-A  3.7  3.9   79   473    9   MOLECULE: SERINE-TRNA LIGASE;                                        2705:  1o20-A  3.7 10.5   78   414   10   MOLECULE: GAMMA-GLUTAMYL PHOSPHATE REDUCTASE;                        2706:  8p3q-F  3.7  2.1   63   178    5   MOLECULE: GLUTAMATE RECEPTOR 2;                                      2707:  3nkz-A  3.7  2.5   53    97    9   MOLECULE: FLAGELLAR PROTEIN FLIT;                                    2708:  6zkf-L  3.7  2.4   55   606    7   MOLECULE: NADH DEHYDROGENASE [UBIQUINONE] FLAVOPROTEIN 1,            2709:  7phk-D  3.7  5.3   76   392    5   MOLECULE: POTASSIUM VOLTAGE-GATED CHANNEL, SHAW-RELATED SUB          2710:  7ad3-A  3.7  4.1   72   298    4   MOLECULE: PHEROMONE ALPHA FACTOR RECEPTOR;                           2711:  5nmo-A  3.7  2.9   77   162    8   MOLECULE: CHROMOSOME PARTITION PROTEIN SMC,CHROMOSOME PARTI          2712:  6ezn-B  3.7  3.0   59   110    5   MOLECULE: DOLICHYL-DIPHOSPHOOLIGOSACCHARIDE--PROTEIN                 2713:  8srq-C  3.7  4.8   74   205   12   MOLECULE: RB1-INDUCIBLE COILED-COIL PROTEIN 1;                       2714:  6ud4-G  3.7  3.6   66   132    8   MOLECULE: GLUTAMATE RECEPTOR 2;                                      2715:  6n4n-F  3.7  3.4   51   191   10   MOLECULE: NS3 PROTEASE;                                              2716:  7xnj-D  3.7  3.1   59    68    2   MOLECULE: STRESS RESPONSE FACILITATOR A, SRFA;                       2717:  4h9o-C  3.7  2.8   56   205   13   MOLECULE: HISTONE H3.3;                                              2718:  5cqs-D  3.7  3.1   65   324    8   MOLECULE: ELONGATOR COMPLEX PROTEIN 1;                               2719:  2itb-A  3.7  3.4   57   199    9   MOLECULE: TRNA-(MS(2)IO(6)A)-HYDROXYLASE, PUTATIVE;                  2720:  8jia-E  3.7  2.6   82   285   12   MOLECULE: CELL DIVISION ATP-BINDING PROTEIN FTSE;                    2721:  1k34-A  3.7  2.5   52    60   10   MOLECULE: TRANSMEMBRANE GLYCOPROTEIN GP41;                           2722:  6cfw-C  3.7  3.1   64   114    2   MOLECULE: MONOVALENT CATION/H+ ANTIPORTER SUBUNIT D;                 2723:  6s7t-H  3.7  3.1   59   112   14   MOLECULE: DOLICHYL-DIPHOSPHOOLIGOSACCHARIDE--PROTEIN                 2724:  5nnv-C  3.7  3.1   82   237   10   MOLECULE: CHROMOSOME PARTITION PROTEIN SMC,CHROMOSOME PARTI          2725:  6n51-B  3.7  2.4   58   793    2   MOLECULE: METABOTROPIC GLUTAMATE RECEPTOR 5;                         2726:  4rsi-A  3.7  5.0   75   282    4   MOLECULE: STRUCTURAL MAINTENANCE OF CHROMOSOMES PROTEIN 2;           2727:  6elu-G  3.7  3.6   61   168    5   MOLECULE: SERUM RESISTANCE ASSOCIATED; VSG PROTEIN;                  2728:  6rn5-A  3.7  6.7   67   283   10   MOLECULE: CHAD DOMAIN PROTEIN;                                       2729:  3oeh-P  3.7  2.4   82   246    9   MOLECULE: ATP SYNTHASE SUBUNIT ALPHA;                                2730:  1rq0-A  3.7  3.2   59   319   12   MOLECULE: PEPTIDE CHAIN RELEASE FACTOR 1;                            2731:  7fda-M  3.7  2.7   85   218    8   MOLECULE: YEAST VACUOLAR ATPASE A SUBUNIT;                           2732:  5u70-A  3.7  3.2   69   901    6   MOLECULE: POTASSIUM CHANNEL SUBFAMILY T MEMBER 1;                    2733:  6jsi-I  3.7  5.3   62    71    2   MOLECULE: FATTY ACID SYNTHASE SUBUNIT BETA;                          2734:  6crv-A  3.7  4.3   71   881   13   MOLECULE: SPIKE GLYCOPROTEIN,FIBRITIN;                               2735:  3k8v-B  3.7  2.9   69   320    7   MOLECULE: FLAGELLIN HOMOLOG;                                         2736:  5x58-B  3.7  3.8   70  1053    7   MOLECULE: SPIKE GLYCOPROTEIN;                                        2737:  8h13-B  3.7  3.8   69   931    6   MOLECULE: SPIKE GLYCOPROTEIN;                                        2738:  7nz0-B  3.7  3.2   80  1467   13   MOLECULE: CHROMOSOME PARTITION PROTEIN MUKB;                         2739:  5xlr-A  3.7  3.9   72  1022    7   MOLECULE: SPIKE GLYCOPROTEIN;                                        2740:  6crw-B  3.7  3.5   69  1068    6   MOLECULE: SPIKE GLYCOPROTEIN,FIBRITIN;                               2741:  5wd8-A  3.6  3.0   57    90   11   MOLECULE: UNCHARACTERIZED PROTEIN;                                   2742:  6c6r-A  3.6  5.0   62   451    6   MOLECULE: SQUALENE MONOOXYGENASE;                                    2743:  6lea-C  3.6  3.4   69   126    7   MOLECULE: FLAGELLAR SECRETION CHAPERONE FLIS;                        2744:  5j1f-A  3.6  2.7   61   188    8   MOLECULE: PLECTIN,PLECTIN;                                           2745:  3ci9-B  3.6  1.8   45    45    7   MOLECULE: HEAT SHOCK FACTOR-BINDING PROTEIN 1;                       2746:  3ezu-A  3.6  3.0   58   336   10   MOLECULE: GGDEF DOMAIN PROTEIN;                                      2747:  4g6d-B  3.6  4.4   65   198    3   MOLECULE: RNA POLYMERASE SIGMA FACTOR RPOD;                          2748:  3f1i-H  3.6  3.6   55    98    5   MOLECULE: HEPATOCYTE GROWTH FACTOR-REGULATED TYROSINE KINAS          2749:  8fbj-A  3.6  2.0   66   307    3   MOLECULE: KWOCA_60;                                                  2750:  7lid-A  3.6  3.2   65   381    6   MOLECULE: MHOR5;                                                     2751:  7vxs-m  3.6  2.4   48   175    6   MOLECULE: NADH DEHYDROGENASE [UBIQUINONE] IRON-SULFUR PROTE          2752:  6db1-A  3.6  2.8   64   137    3   MOLECULE: PUTATIVE METHYL-ACCEPTING CHEMOTAXIS PROTEIN;              2753:  4yxw-G  3.6  2.8   63   213    2   MOLECULE: ATP SYNTHASE SUBUNIT ALPHA, MITOCHONDRIAL;                 2754:  5zle-A  3.6  6.9   62   225    8   MOLECULE: CYTOCHROME B REDUCTASE 1;                                  2755:  6xr1-A  3.6  4.3   56   513   11   MOLECULE: DTOR_9X57R;                                                2756:  5a7d-R  3.6  2.6   61   311    7   MOLECULE: PINS;                                                      2757:  5x3q-A  3.6  3.9   59   313    5   MOLECULE: ENVELOPE GLYCOPROTEIN;                                     2758:  2xco-A  3.6  5.6   75   636    4   MOLECULE: DNA GYRASE SUBUNIT B, DNA GYRASE SUBUNIT A;                2759:  1ifl-A  3.6  2.1   50    53   10   MOLECULE: INOVIRUS;                                                  2760:  7x29-A  3.6  3.6   68   991    9   MOLECULE: SPIKE GLYCOPROTEIN;                                        2761:  6gyb-C  3.6  4.9   58   205   14   MOLECULE: VIRB7;                                                     2762:  4tt0-B  3.6  3.5   59   131    7   MOLECULE: DENEDDYLASE;                                               2763:  8g0l-B  3.6  3.2   69   925   12   MOLECULE: N-ALPHA-ACETYLTRANSFERASE 20;                              2764:  5d60-B  3.6  1.9   50    61    8   MOLECULE: PUTATIVE TRANSCRIPTION FACTOR;                             2765:  3zc0-B  3.6  2.9   57   191    2   MOLECULE: AFTRAX;                                                    2766:  7ogt-A  3.6  2.8   86   688    7   MOLECULE: STRUCTURAL MAINTENANCE OF CHROMOSOMES PROTEIN 1;           2767:  7qe5-A  3.6  6.6   62   616   15   MOLECULE: SIALIC ACID TRAP TRANSPORTER PERMEASE PROTEIN SIA          2768:  6klb-A  3.6  4.8   69  1118    9   MOLECULE: LBCAS12A;                                                  2769:  8b6l-P  3.6  3.7   56   602   11   MOLECULE: PROTEIN TRANSPORT PROTEIN SEC61 SUBUNIT ALPHA ISO          2770:  2btp-A  3.6  5.2   50   248   10   MOLECULE: 14-3-3 PROTEIN TAU;                                        2771:  8etc-F  3.6  3.8   58   218    5   MOLECULE: RNA (2151-MER);                                            2772:  2jx0-A  3.6  4.5   69   131    7   MOLECULE: ARF GTPASE-ACTIVATING PROTEIN GIT1;                        2773:  7e40-D  3.6  3.3   78   345    9   MOLECULE: PROTEIN PHOSPHATE STARVATION RESPONSE 2;                   2774:  6nr8-3  3.6  4.6   78   132    6   MOLECULE: PREFOLDIN SUBUNIT 1;                                       2775:  4dl0-K  3.6  2.5   50    97    8   MOLECULE: V-TYPE PROTON ATPASE SUBUNIT C;                            2776:  6hd5-t  3.6  2.9   66   838    6   MOLECULE: N-TERMINAL ACETYLTRANSFERASE A COMPLEX SUBUNIT NA          2777:  5jbr-A  3.6  4.7   60   149   10   MOLECULE: UNCHARACTERIZED PROTEIN BCAV_2135;                         2778:  5oqj-W  3.6  5.6   65   258    5   MOLECULE: DNA-DIRECTED RNA POLYMERASE II SUBUNIT RPB1;               2779:  8hnc-A  3.6  5.6   74   570    4   MOLECULE: SOLUTE CARRIER ORGANIC ANION TRANSPORTER FAMILY M          2780:  5yr0-B  3.6  1.5   44    44    2   MOLECULE: BECLIN-1;                                                  2781:  7dkh-I  3.6  3.2   62   914    3   MOLECULE: RNA POLYMERASE-ASSOCIATED PROTEIN CTR9;                    2782:  4y66-D  3.6  4.3   63   208   10   MOLECULE: MND1;                                                      2783:  4r4l-C  3.6  2.3   48    49    6   MOLECULE: CGMP-DEPENDENT PROTEIN KINASE 1;                           2784:  7p2p-C  3.6  5.6   62   174    3   MOLECULE: SIGNAL PEPTIDASE COMPLEX CATALYTIC SUBUNIT SEC11A          2785:  5al6-A  3.6  1.5   44    44   11   MOLECULE: ANASTRAL SPINDLE 2;                                        2786:  6ugm-X  3.6  2.7   63    73    8   MOLECULE: HISTONE H3;                                                2787:  7d2e-D  3.6  2.9   50    63   10   MOLECULE: LIPRIN-ALPHA-2;                                            2788:  3he5-A  3.6  2.6   46    47    2   MOLECULE: SYNZIP1;                                                   2789:  3vou-A  3.6  6.1   64   139    6   MOLECULE: ION TRANSPORT 2 DOMAIN PROTEIN, VOLTAGE-GATED SOD          2790:  2e87-A  3.6  4.8   76   356   11   MOLECULE: HYPOTHETICAL PROTEIN PH1320;                               2791:  6dfk-H  3.6  3.1   80   216    5   MOLECULE: SUBUNIT OF PROTEASEOME ACTIVATOR COMPLEX,PUTATIVE          2792:  1x03-A  3.6  2.4   73   210   14   MOLECULE: SH3-CONTAINING GRB2-LIKE PROTEIN 2;                        2793:  2izp-A  3.6  3.2   82   272    5   MOLECULE: PUTATIVE MEMBRANE ANTIGEN;                                 2794:  6y07-A  3.6  3.6   69   154    1   MOLECULE: SOHAIR;                                                    2795:  6pe5-Q  3.6  2.4   62   369    6   MOLECULE: V-TYPE PROTON ATPASE SUBUNIT A, VACUOLAR ISOFORM;          2796:  1gqe-A  3.6  3.9   67   362    6   MOLECULE: RELEASE FACTOR 2;                                          2797:  6em5-h  3.6  4.4   60   119    7   MOLECULE: 5.8S RIBOSOMAL RNA;                                        2798:  5n5e-Z  3.6  4.9   60    98    7   MOLECULE: PFC_05175;                                                 2799:  5n5e-L  3.6  4.8   59    97    7   MOLECULE: PFC_05175;                                                 2800:  5n5e-X  3.6  4.9   60    97    7   MOLECULE: PFC_05175;                                                 2801:  5iea-D  3.6  3.7   65   118    8   MOLECULE: TRIPARTITE MOTIF-CONTAINING PROTEIN 5, SERINE--TR          2802:  6dk7-D  3.6  2.8   57   225   11   MOLECULE: RETS (REGULATOR OF EXOPOLYSACCHARIDE AND TYPE III          2803:  5tw1-D  3.6  5.2   78  1248    8   MOLECULE: RNA POLYMERASE-BINDING PROTEIN RBPA;                       2804:  5a7d-L  3.6  3.6   69   284   12   MOLECULE: PINS;                                                      2805:  5ijp-A  3.6  4.0   79   178    9   MOLECULE: PUTATIVE UNCHARACTERIZED PROTEIN;                          2806:  6aki-P  3.6  2.5   53   150    6   MOLECULE: CALCIUM RELEASE-ACTIVATED CALCIUM CHANNEL PROTEIN          2807:  6gol-A  3.6  2.7   57    65    9   MOLECULE: HEMAGGLUTININ TRI-STALK;                                   2808:  5n5e-V  3.6  4.9   60    97    7   MOLECULE: PFC_05175;                                                 2809:  8ow0-Y  3.6  6.0   65   223   14   MOLECULE: C0N3 DNA;                                                  2810:  5n5e-H  3.6  4.9   60    97    7   MOLECULE: PFC_05175;                                                 2811:  5n5e-D  3.6  4.9   60    97    7   MOLECULE: PFC_05175;                                                 2812:  2nnw-A  3.6  3.5   74   350   12   MOLECULE: NOP5/NOP56 RELATED PROTEIN;                                2813:  7sn7-c  3.6  2.8   85   546    4   MOLECULE: FLAGELLIN;                                                 2814:  6djb-B  3.6  3.7   73   725    3   MOLECULE: VOLUME-REGULATED ANION CHANNEL SUBUNIT LRRC8A;             2815:  1sks-A  3.6  8.2   68   681    7   MOLECULE: 5'-                                                        2816:  1h6g-A  3.6  3.0   60   255    8   MOLECULE: ALPHA-1 CATENIN;                                           2817:  5x5f-A  3.6  4.2   66  1140    9   MOLECULE: S PROTEIN;                                                 2818:  7sn7-E  3.6  2.9   85   546    5   MOLECULE: FLAGELLIN;                                                 2819:  8pr3-B  3.6  3.9   53   105   11   MOLECULE: C-JUN-AMINO-TERMINAL KINASE-INTERACTING PROTEIN 3          2820:  8e7s-o  3.6  4.5   61   269    8   MOLECULE: CYTOCHROME B-C1 COMPLEX SUBUNIT 1, MITOCHONDRIAL;          2821:  6vp9-B  3.6  2.8   62   914    8   MOLECULE: N-ALPHA-ACETYLTRANSFERASE 20;                              2822:  5m4y-A  3.6  3.1   71   177    4   MOLECULE: PROTEIN SSO2;                                              2823:  8tdj-A  3.6  5.5   84   467   10   MOLECULE: MECHANOSENSITIVE ION CHANNEL PROTEIN 10;                   2824:  6wki-A  3.6  4.8   60   266    7   MOLECULE: PENTALENENE SYNTHASE;                                      2825:  7yvb-A  3.6  3.8   64   471    5   MOLECULE: FMRFAMIDE-GATED NA+ CHANNEL;                               2826:  7qhm-J  3.6  5.3   73   102   12   MOLECULE: CYTOCHROME BC1 COMPLEX RIESKE IRON-SULFUR SUBUNIT          2827:  7f6j-C  3.6  3.0   71   106   10   MOLECULE: RAS-RELATED PROTEIN RAB-7A;                                2828:  1d8s-G  3.6  2.9   82   214    0   MOLECULE: F1 ATPASE (ALPHA SUBUNIT);                                 2829:  6rdd-7  3.6  5.7   70   176    9   MOLECULE: ASA-10: POLYTOMELLA F-ATP SYNTHASE ASSOCIATED SUB          2830:  6dt0-A  3.6  7.1   75   236   12   MOLECULE: MITOCHONDRIAL CALCIUM UNIPORTER;                           2831:  3nry-A  3.6  2.7   64   125    5   MOLECULE: PROTEIN REGULATOR OF CYTOKINESIS 1;                        2832:  7xlb-A  3.6  3.3   74   336    5   MOLECULE: PANNEXIN-2;                                                2833:  6wca-A  3.6  3.5   62   367    8   MOLECULE: ENDOSOMAL/LYSOSOMAL POTASSIUM CHANNEL TMEM175;             2834:  6w1w-A  3.6  3.5   59   348   10   MOLECULE: MOTILITY-ASSOCIATED KILLING FACTOR MAKB;                   2835:  6wca-B  3.6  3.5   62   367    8   MOLECULE: ENDOSOMAL/LYSOSOMAL POTASSIUM CHANNEL TMEM175;             2836:  6fnd-A  3.6  2.8   52   423   10   MOLECULE: APICAL COMPLEX LYSINE METHYLTRANSFERASE;                   2837:  6m04-A  3.6  4.4   77   714    8   MOLECULE: VOLUME-REGULATED ANION CHANNEL SUBUNIT LRRC8D;             2838:  6jp8-E  3.6  4.1   58   169    2   MOLECULE: VOLTAGE-DEPENDENT CALCIUM CHANNEL GAMMA-1 SUBUNIT          2839:  7nj1-A  3.6  4.6   60  1448    5   MOLECULE: SEPARIN;                                                   2840:  6nrc-3  3.6  5.6   79   132    8   MOLECULE: PREFOLDIN SUBUNIT 1;                                       2841:  6dk7-B  3.6  2.5   54   224    9   MOLECULE: RETS (REGULATOR OF EXOPOLYSACCHARIDE AND TYPE III          2842:  7zw1-B  3.6  3.3   77   316    5   MOLECULE: PERIPHERIN-2;                                              2843:  2wcd-K  3.6  3.1   80   285   11   MOLECULE: HEMOLYSIN E, CHROMOSOMAL;                                  2844:  6ud8-E  3.6  2.5   62   132    6   MOLECULE: GLUTAMATE RECEPTOR 2;                                      2845:  7cmg-A  3.6  4.8   73   487    4   MOLECULE: POR SECRETION SYSTEM PROTEIN PORM/GLDM;                    2846:  6vli-A  3.6  2.3   53   154    2   MOLECULE: REGULATORY PROTEIN CII;                                    2847:  2qyp-B  3.6  6.4   64    81    8   MOLECULE: PROACTIVATOR POLYPEPTIDE;                                  2848:  5mil-A  3.6  3.4   54   157    2   MOLECULE: DUTPASE FAMILY PROTEIN;                                    2849:  6pfv-T  3.6  3.0   71   164    7   MOLECULE: AMFC PROTEIN;                                              2850:  7sau-F  3.6  3.1   56    71    4   MOLECULE: GLDM;                                                      2851:  7xnj-C  3.6  3.0   57    62    2   MOLECULE: STRESS RESPONSE FACILITATOR A, SRFA;                       2852:  7f3u-B  3.6  4.4   59   328    5   MOLECULE: TRANSMEMBRANE PROTEIN 120A;                                2853:  6t8d-X  3.6  2.7   84   355    7   MOLECULE: MAKB;                                                      2854:  4brb-B  3.6  3.2   63    99   10   MOLECULE: DIACYLGLYCEROL KINASE;                                     2855:  6gdi-A  3.6  3.7   82  1182   11   MOLECULE: MULTIDRUG RESISTANCE PROTEIN 1A;                           2856:  4bpd-B  3.6  3.2   63    99   10   MOLECULE: DIACYLGLYCEROL KINASE;                                     2857:  5fej-B  3.6  2.7   51   140    8   MOLECULE: COPM;                                                      2858:  8gxm-A  3.6  2.6   64   119    8   MOLECULE: SURP AND G-PATCH DOMAIN-CONTAINING PROTEIN 1;              2859:  7s5c-I  3.6  2.1   48    63    4   MOLECULE: ENCB;                                                      2860:  6w2w-A  3.6  3.6   61   224   10   MOLECULE: JUNCTION 24 DHR14-DHR18;                                   2861:  6rd4-7  3.6  5.6   71   176    8   MOLECULE: ASA-10: POLYTOMELLA F-ATP SYNTHASE ASSOCIATED SUB          2862:  1hx1-B  3.6  4.5   70   112    6   MOLECULE: HEAT SHOCK 70 KDA PROTEIN 8;                               2863:  3jcu-z  3.6  2.9   55    61    9   MOLECULE: PHOTOSYSTEM II PROTEIN D1;                                 2864:  5u1d-B  3.6  3.3   79   552    9   MOLECULE: ANTIGEN PEPTIDE TRANSPORTER 1;                             2865:  7loh-C  3.6  4.7   68   158   12   MOLECULE: TRANSMEMBRANE PROTEIN GP41;                                2866:  6vme-N  3.6  2.4   53    67   11   MOLECULE: TUMOR SUSCEPTIBILITY GENE 101 PROTEIN;                     2867:  8akr-R  3.6  4.0   80   196    9   MOLECULE: CHLOROPLAST MEMBRANE-ASSOCIATED 30 KD PROTEIN;             2868:  1s0g-A  3.6  3.2   50  1288   10   MOLECULE: BOTULINUM NEUROTOXIN TYPE B;                               2869:  7y6t-B  3.6  3.4   64  1224    5   MOLECULE: SPIKE GLYCOPROTEIN;                                        2870:  6nb6-B  3.6  3.7   70  1026    6   MOLECULE: SPIKE GLYCOPROTEIN;                                        2871:  6x45-B  3.6  2.9   49    53   12   MOLECULE: SPIKE PROTEIN S2';                                         2872:  8gub-B  3.6  3.4   82   276    6   MOLECULE: PHOSPHATIDYLINOSITOL 4,5-BISPHOSPHATE 3-KINASE CA          2873:  8cvi-H  3.6  3.5   69   268   10   MOLECULE: FLAGELLIN;                                                 2874:  6x45-A  3.6  1.7   48    51    4   MOLECULE: SPIKE PROTEIN S2';                                         2875:  8fcy-A  3.6  3.2   64  2864    5   MOLECULE: CYTOPLASMIC DYNEIN 1 HEAVY CHAIN 1;                        2876:  5nug-B  3.6  2.9   63  2920    6   MOLECULE: CYTOPLASMIC DYNEIN 1 HEAVY CHAIN 1;                        2877:  5j65-A  3.5  3.8   79   402    6   MOLECULE: PESTICIDAL CRYSTAL PROTEIN CRY6AA;                         2878:  6ks0-A  3.5  2.5   63   286    3   MOLECULE: ADIPONECTIN RECEPTOR PROTEIN 1;                            2879:  3hrn-A  3.5  2.0   50    63    6   MOLECULE: TRANSIENT RECEPTOR POTENTIAL (TRP) CHANNEL                 2880:  7bin-Q  3.5  3.6   68   133    7   MOLECULE: FLAGELLAR BIOSYNTHETIC PROTEIN FLIP;                       2881:  7jmn-P  3.5  3.8   65   810    8   MOLECULE: MEDIATOR OF RNA POLYMERASE II TRANSCRIPTION SUBUN          2882:  8eoi-K  3.5  3.2   63  1116   13   MOLECULE: ER MEMBRANE PROTEIN COMPLEX SUBUNIT 1;                     2883:  7loi-A  3.5  4.4   68   175   10   MOLECULE: TRANSMEMBRANE PROTEIN GP41;                                2884:  1y9b-A  3.5  2.4   54    81    9   MOLECULE: CONSERVED HYPOTHETICAL PROTEIN;                            2885:  7wdk-B  3.5  2.6   61   348   13   MOLECULE: PHOSPHOLIPASE D;                                           2886:  8ap7-a  3.5  2.8   67   231    0   MOLECULE: ATP SYNTHASE SUBUNIT A;                                    2887:  4i1l-A  3.5  1.9   50    62   12   MOLECULE: FORKHEAD BOX PROTEIN P3;                                   2888:  7dgu-A  3.5  2.6   46    97    9   MOLECULE: A1;                                                        2889:  1gd2-E  3.5  1.8   50    65    0   MOLECULE: DNA (5'-                                                   2890:  5vpc-B  3.5  1.8   50    68    4   MOLECULE: PROTEIN FOSB;                                              2891:  8hoy-A  3.5  3.6   60  1006    5   MOLECULE: DNA POLYMERASE;                                            2892:  6k2j-B  3.5  3.0   48    83    8   MOLECULE: UPF0335 PROTEIN CCNA_03428;                                2893:  3rip-A  3.5  4.5   68   568    3   MOLECULE: GAMMA-TUBULIN COMPLEX COMPONENT 4;                         2894:  2i7u-A  3.5  3.5   53    62   11   MOLECULE: FOUR-ALPHA-HELIX BUNDLE;                                   2895:  1szi-A  3.5  2.5   63   194    3   MOLECULE: MANNOSE-6-PHOSPHATE RECEPTOR BINDING PROTEIN 1;            2896:  8tvh-A  3.5  2.9   51    86    8   MOLECULE: 4G5 LIGHT CHAIN;                                           2897:  6irr-A  3.5  3.5   60   133    7   MOLECULE: DISRUPTED IN SCHIZOPHRENIA 1 HOMOLOG,CYCLIC AMP-D          2898:  4v1a-o  3.5  1.8   49    94   12   MOLECULE: MITORIBOSOMAL PROTEIN ML37, MRPL37;                        2899:  3va9-A  3.5  2.8   60   127    3   MOLECULE: SENSOR HISTIDINE KINASE;                                   2900:  4gx2-B  3.5  3.0   61   548    5   MOLECULE: TRKA DOMAIN PROTEIN;                                       2901:  6ucv-J  3.5  2.1   48    55   13   MOLECULE: MITOCHONDRIAL IMPORT RECEPTOR SUBUNIT TOM40;               2902:  7bst-A  3.5  4.2   76   384   11   MOLECULE: TYPE I RESTRICTION ENZYME R PROTEIN;                       2903:  7eqb-A  3.5  1.7   50    68    6   MOLECULE: KINESIN-LIKE PROTEIN;                                      2904:  7qdr-B  3.5  3.2   51  1212    8   MOLECULE: HELICASE SKI2W;                                            2905:  8ap7-l  3.5  2.2   50    65    4   MOLECULE: ATP SYNTHASE SUBUNIT A;                                    2906:  5mw9-A  3.5  2.3   50    66   12   MOLECULE: CENTROSOMIN;                                               2907:  8iq4-R  3.5  2.4   63   274    5   MOLECULE: PROSTAGLANDIN F2-ALPHA RECEPTOR;                           2908:  3cl3-D  3.5  2.3   50    59    6   MOLECULE: ORF K13;                                                   2909:  4wj1-A  3.5  3.6   80   286    5   MOLECULE: ANTIGEN MTB48, MYCOBACTERIAL PROTEIN;                      2910:  4pxj-C  3.5  2.9   50    61    2   MOLECULE: C-JUN-AMINO-TERMINAL KINASE-INTERACTING PROTEIN 3          2911:  6wxu-B  3.5  2.8   65   273    9   MOLECULE: DUAL OXIDASE 1;                                            2912:  3wuv-Q  3.5  5.1   54    62   11   MOLECULE: CENTROSOMAL PROTEIN OF 55 KDA;                             2913:  7w91-A  3.5  1.9   48    53    4   MOLECULE: CENTROSOMAL PROTEIN OF 63 KDA;                             2914:  8eki-A  3.5  3.4   73   170    8   MOLECULE: PROTEIN TRANSPORT PROTEIN SEC20;                           2915:  8hk0-B  3.5  4.4   61   379   13   MOLECULE: DEHYDROGENASE;                                             2916:  5z7g-C  3.5  1.2   43    43    7   MOLECULE: TAX1-BINDING PROTEIN 1;                                    2917:  7nad-5  3.5  2.5   53   123    6   MOLECULE: 25S RRNA;                                                  2918:  5oqm-i  3.5  2.5   54   136    7   MOLECULE: DNA-DIRECTED RNA POLYMERASE II SUBUNIT RPB1;               2919:  5dol-B  3.5  3.0   50    62    8   MOLECULE: INITIATION-CONTROL PROTEIN YABA;                           2920:  8ixk-E  3.5  3.6   59   109   12   MOLECULE: ATTACHMENT PROTEIN G3P;                                    2921:  7dwb-A  3.5  4.6   78   421    5   MOLECULE: PANNEXIN-1;                                                2922:  8afz-A  3.5  2.0   68   381    7   MOLECULE: SORTING NEXIN-1;                                           2923:  3lt6-F  3.5  1.9   50    63    4   MOLECULE: ADHESIN YADA;                                              2924:  5mnt-B  3.5  3.9   71   421   14   MOLECULE: A2 MATURATION PROTEIN;                                     2925:  5afr-A  3.5  3.7   61   332    7   MOLECULE: DYNEIN HEAVY CHAIN, CYTOPLASMIC;                           2926:  2pfd-A  3.5  3.5   63   540    3   MOLECULE: FORMIMIDOYLTRANSFERASE-CYCLODEAMINASE;                     2927:  7wvt-A  3.5  8.3   64   364    8   MOLECULE: PHOSPHATIDYLINOSITOL TRANSFER PROTEIN CSR1;                2928:  8fck-H  3.5  2.2   50   218    6   MOLECULE: HAUS AUGMIN-LIKE COMPLEX SUBUNIT 1;                        2929:  7ofq-b  3.5  5.6   52   213    6   MOLECULE: ARCHAELLIN;                                                2930:  2qfa-B  3.5  3.3   52    62    4   MOLECULE: BACULOVIRAL IAP REPEAT-CONTAINING PROTEIN 5;               2931:  8i03-B  3.5  3.8   71   639    6   MOLECULE: PAIRED AMPHIPATHIC HELIX PROTEIN PST1;                     2932:  8b9z-K  3.5  3.7   55    96   13   MOLECULE: NADH-UBIQUINONE OXIDOREDUCTASE CHAIN 3;                    2933:  8wzb-D  3.5  1.8   46    46   11   MOLECULE: DPY30 DOMAIN CONTAINING 2;                                 2934:  4gkg-A  3.5  2.7   48    52    8   MOLECULE: C4-DICARBOXYLATE TRANSPORT SENSOR PROTEIN DCTB;            2935:  5ipx-A  3.5  3.7   69   282   10   MOLECULE: ORF49 PROTEIN;                                             2936:  2yev-B  3.5  5.9   72   319    3   MOLECULE: CYTOCHROME C OXIDASE POLYPEPTIDE I+III;                    2937:  3brt-D  3.5  2.3   50    62    4   MOLECULE: INHIBITOR OF NUCLEAR FACTOR KAPPA-B KINASE SUBUNI          2938:  1i5p-A  3.5  2.6   62   633    5   MOLECULE: PESTICIDIAL CRYSTAL PROTEIN CRY2AA;                        2939:  6soz-A  3.5  4.1   82   323    9   MOLECULE: ESAG6, SUBUNIT OF HETERODIMERIC TRANSFERRIN RECEP          2940:  8buy-B  3.5  6.8   76   126    3   MOLECULE: GRANULE ASSOCIATED RAC AND RHOG EFFECTOR PROTEIN           2941:  3g9g-A  3.5  5.1   86   250    6   MOLECULE: SUPPRESSOR OF YEAST PROFILIN DELETION;                     2942:  6y9a-A  3.5  3.1   75   169   11   MOLECULE: B-LYMPHOCYTE ANTIGEN CD20;                                 2943:  8txr-h  3.5  3.1   51    59    4   MOLECULE: EXODEOXYRIBONUCLEASE 7 LARGE SUBUNIT;                      2944:  6ree-7  3.5  5.1   66   176    6   MOLECULE: ASA-10: POLYTOMELLA F-ATP SYNTHASE ASSOCIATED SUB          2945:  6ref-7  3.5  5.1   66   176    6   MOLECULE: ASA-10: POLYTOMELLA F-ATP SYNTHASE ASSOCIATED SUB          2946:  5m4y-E  3.5  3.1   71   181    4   MOLECULE: PROTEIN SSO2;                                              2947:  6b85-A  3.5  2.8   71   215    7   MOLECULE: TMHC4_R;                                                   2948:  1oed-C  3.5  2.9   55   127    9   MOLECULE: ACETYLCHOLINE RECEPTOR PROTEIN, ALPHA CHAIN;               2949:  4xng-A  3.5  2.9   72   140    6   MOLECULE: UNCHARACTERIZED PROTEIN MG218.1;                           2950:  5kk2-H  3.5  2.8   59   168   10   MOLECULE: GLUTAMATE RECEPTOR 2;                                      2951:  5o60-Z  3.5  2.3   48    64    8   MOLECULE: 50S RIBOSOMAL PROTEIN BL37;                                2952:  2efk-A  3.5  4.7   84   273    6   MOLECULE: CDC42-INTERACTING PROTEIN 4;                               2953:  7sn7-M  3.5  2.9   85   546    5   MOLECULE: FLAGELLIN;                                                 2954:  4xc6-A  3.5  3.6   67  1067   16   MOLECULE: ISOBUTYRYL-COA MUTASE FUSED;                               2955:  3pxi-C  3.5  5.3   62   711   10   MOLECULE: ADAPTER PROTEIN MECA 1;                                    2956:  2wmm-A  3.5  3.2   59   162    5   MOLECULE: CHROMOSOME PARTITION PROTEIN MUKB;                         2957:  7r1e-A  3.5  5.6   70   626    7   MOLECULE: PESTICIDAL CRYSTAL PROTEIN CRY11BA;                        2958:  3zni-A  3.5  6.1   64   390    8   MOLECULE: E3 UBIQUITIN-PROTEIN LIGASE CBL-B;                         2959:  6neq-O  3.5  2.7   51   173    6   MOLECULE: 28S RIBOSOMAL RNA, MITOCHONDRIAL;                          2960:  5lbm-D  3.5  3.2   59    90    5   MOLECULE: TRANSCRIPTIONAL REPRESSOR FRMR;                            2961:  8th8-A  3.5  5.7   70   290    3   MOLECULE: DYNEIN REGULATORY COMPLEX PROTEIN 1/2 N-TERMINAL           2962:  6yvu-B  3.5  3.6   84  1191    8   MOLECULE: STRUCTURAL MAINTENANCE OF CHROMOSOMES PROTEIN 2,S          2963:  5ys9-A  3.5  2.7   71   692    7   MOLECULE: ACYL-COENZYME A OXIDASE 3;                                 2964:  6re2-7  3.5  6.0   70   176    7   MOLECULE: ASA-10: POLYTOMELLA F-ATP SYNTHASE ASSOCIATED SUB          2965:  1ehk-A  3.5  3.5   64   544    3   MOLECULE: BA3-TYPE CYTOCHROME-C OXIDASE;                             2966:  3hq2-B  3.5  3.6   61   497    7   MOLECULE: BACILLUS SUBTILIS M32 CARBOXYPEPTIDASE;                    2967:  6wcc-A  3.5  3.5   62   367    8   MOLECULE: ENDOSOMAL/LYSOSOMAL POTASSIUM CHANNEL TMEM175;             2968:  6wcc-B  3.5  3.8   63   367    8   MOLECULE: ENDOSOMAL/LYSOSOMAL POTASSIUM CHANNEL TMEM175;             2969:  6me8-A  3.5  2.7   44   449    9   MOLECULE: SOLUBLE CYTOCHROME B562,MELATONIN RECEPTOR TYPE 1          2970:  2bdp-A  3.5  6.7   58   580    9   MOLECULE: DNA (5'-D(*GP*CP*AP*TP*GP*AP*TP*GP*C)-3');                 2971:  5ic0-A  3.5  3.0   65   465    8   MOLECULE: TALIN-1;                                                   2972:  5up9-B  3.5  3.7   66   174    8   MOLECULE: FERRITIN HEAVY CHAIN;                                      2973:  4fla-B  3.5  3.3   69   124    9   MOLECULE: REGULATION OF NUCLEAR PRE-MRNA DOMAIN-CONTAINING           2974:  7m68-A  3.5  4.6   82  1390   12   MOLECULE: METAL RESISTANCE PROTEIN YCF1;                             2975:  7y6b-D  3.5  2.3   51    72    8   MOLECULE: ESCE/YSCE/SSAE FAMILY TYPE III SECRETION SYSTEM N          2976:  6o7u-f  3.5  3.3   53    61    4   MOLECULE: V-TYPE PROTON ATPASE SUBUNIT A, GOLGI ISOFORM;             2977:  7xnj-A  3.5  3.1   60    67    2   MOLECULE: STRESS RESPONSE FACILITATOR A, SRFA;                       2978:  5vgz-d  3.5  2.3   49   257    2   MOLECULE: 26S PROTEASOME REGULATORY SUBUNIT 7;                       2979:  7p34-A  3.5  3.3   84   391    5   MOLECULE: PEPTIDE ANTIBIOTIC TRANSPORTER SBMA;                       2980:  7oca-E  3.5  3.3   73   158    4   MOLECULE: GLUTAMATE RECEPTOR 1;                                      2981:  7fde-M  3.5  2.9   82   218    9   MOLECULE: V-TYPE PROTON ATPASE SUBUNIT C;                            2982:  7s5k-D  3.5  2.2   48    64    4   MOLECULE: ENCB;                                                      2983:  3iox-A  3.5  2.8   71   489    6   MOLECULE: AGI/II;                                                    2984:  7p34-B  3.5  3.3   84   391    5   MOLECULE: PEPTIDE ANTIBIOTIC TRANSPORTER SBMA;                       2985:  4ons-A  3.5  3.4   63   230    8   MOLECULE: CATENIN ALPHA-2;                                           2986:  6d7w-C  3.5  4.3   77   269    6   MOLECULE: MITOCHONDRIAL CALCIUM UNIPORTER;                           2987:  6re0-7  3.5  6.0   74   176    7   MOLECULE: ASA-10: POLYTOMELLA F-ATP SYNTHASE ASSOCIATED SUB          2988:  6re8-7  3.5  6.0   74   176    7   MOLECULE: ASA-10: POLYTOMELLA F-ATP SYNTHASE ASSOCIATED SUB          2989:  6rdz-7  3.5  6.0   74   176    7   MOLECULE: ASA-10: POLYTOMELLA F-ATP SYNTHASE ASSOCIATED SUB          2990:  6rdi-7  3.5  5.7   71   176   10   MOLECULE: ASA-10: POLYTOMELLA F-ATP SYNTHASE ASSOCIATED SUB          2991:  2fic-B  3.5  3.1   74   201    7   MOLECULE: BRIDGING INTEGRATOR 1;                                     2992:  6rd9-7  3.5  6.0   70   176    7   MOLECULE: ASA-10: POLYTOMELLA F-ATP SYNTHASE ASSOCIATED SUB          2993:  6rdc-7  3.5  5.7   72   176    8   MOLECULE: ASA-10: POLYTOMELLA F-ATP SYNTHASE ASSOCIATED SUB          2994:  6rdh-7  3.5  5.7   71   176   10   MOLECULE: ASA-10: POLYTOMELLA F-ATP SYNTHASE ASSOCIATED SUB          2995:  6re9-7  3.5  6.0   74   176    7   MOLECULE: ASA-10: POLYTOMELLA F-ATP SYNTHASE ASSOCIATED SUB          2996:  6hwh-A  3.5  3.0   67   380   12   MOLECULE: UBIQUINOL-CYTOCHROME C REDUCTASE IRON-SULFUR SUBU          2997:  7phk-G  3.5  4.7   73   393    4   MOLECULE: POTASSIUM VOLTAGE-GATED CHANNEL, SHAW-RELATED SUB          2998:  3whl-H  3.5  2.3   53    91    9   MOLECULE: PROTEASOME-ACTIVATING NUCLEOTIDASE, 26S PROTEASE           2999:  4qn1-A  3.5  3.8   75   372    7   MOLECULE: E3 UBIQUITIN-PROTEIN LIGASE SHPRH;                         3000:  5dwk-D  3.5  2.8   51    87    2   MOLECULE: DIACYLGLYCEROL KINASE;                                     3001:  7s5k-J  3.5  2.2   48    64    4   MOLECULE: ENCB;                                                      3002:  5l0g-D  3.5  2.9   61   170    8   MOLECULE: VINCULIN;                                                  3003:  7s5k-A  3.5  2.1   48    64    4   MOLECULE: ENCB;                                                      3004:  6cs1-A  3.5  3.7   70  1066    6   MOLECULE: SPIKE GLYCOPROTEIN,FIBRITIN;                               3005:  8h14-B  3.5  3.7   68  1029    4   MOLECULE: SPIKE GLYCOPROTEIN;                                        3006:  7akj-B  3.5  3.7   69  1084    4   MOLECULE: SPIKE GLYCOPROTEIN;                                        3007:  8ts9-B  3.5  3.3   82   272    6   MOLECULE: PHOSPHATIDYLINOSITOL 4,5-BISPHOSPHATE 3-KINASE CA          3008:  8h13-C  3.5  3.8   69   931    6   MOLECULE: SPIKE GLYCOPROTEIN;                                        3009:  5j1i-A  3.4  2.6   67   346    7   MOLECULE: PLECTIN;                                                   3010:  4xng-D  3.4  3.3   75   144    7   MOLECULE: UNCHARACTERIZED PROTEIN MG218.1;                           3011:  8j2g-A  3.4  3.6   60   325    5   MOLECULE: PROTON-COUPLED ZINC ANTIPORTER SLC30A1;                    3012:  1wt6-B  3.4  2.1   50    67    8   MOLECULE: MYOTONIN-PROTEIN KINASE;                                   3013:  8umy-D  3.4  3.6   42   329    5   MOLECULE: CHROMOSOME TRANSMISSION FIDELITY PROTEIN 18 HOMOL          3014:  5xmk-G  3.4  3.3   47    54    9   MOLECULE: VACUOLAR PROTEIN SORTING-ASSOCIATED PROTEIN 4;             3015:  8bt6-A  3.4  5.1   65  2633    6   MOLECULE: PUTATIVE ANONYMOUS ANTIGEN-1;                              3016:  3m0d-A  3.4  2.1   50    63    4   MOLECULE: TNF RECEPTOR-ASSOCIATED FACTOR 2;                          3017:  1dh3-A  3.4  2.9   50    55    4   MOLECULE: DNA (5'-                                                   3018:  6gvw-B  3.4  3.6   60   262    8   MOLECULE: BRCA1-A COMPLEX SUBUNIT ABRAXAS 1;                         3019:  8bhw-A  3.4  5.4   80   332    5   MOLECULE: ECA POLYSACCHARIDE CHAIN LENGTH MODULATION PROTEI          3020:  2oev-A  3.4  3.9   72   697    8   MOLECULE: PROGRAMMED CELL DEATH 6-INTERACTING PROTEIN;               3021:  1m1j-D  3.4  2.8   76   194    4   MOLECULE: FIBRINOGEN ALPHA SUBUNIT;                                  3022:  5gna-B  3.4  2.9   49    56    2   MOLECULE: FLAGELLAR PROTEIN FLIT;                                    3023:  7ydq-A  3.4  5.1   58   597    5   MOLECULE: NUCLEOSIDE TRANSPORTER 1,GREEN FLUORESCENT PROTEI          3024:  7njt-d  3.4  3.3   50    61   10   MOLECULE: ATP SYNTHASE SUBUNIT C;                                    3025:  3rgw-L  3.4  4.6   71   602    3   MOLECULE: MEMBRANE-BOUND HYDROGENASE (NIFE) LARGE SUBUNIT H          3026:  1sjj-A  3.4  3.5   71   863   13   MOLECULE: ACTININ;                                                   3027:  7sfy-B  3.4  2.2   44    44    7   MOLECULE: PROTEIN MIS18-ALPHA;                                       3028:  7ura-A  3.4  5.0   68   432   12   MOLECULE: ISOFORM 2 OF PROTEIN-SERINE O-PALMITOLEOYLTRANSFE          3029:  3p01-A  3.4  2.9   50   178    2   MOLECULE: TWO-COMPONENT RESPONSE REGULATOR;                          3030:  7x29-B  3.4  3.5   67  1190    9   MOLECULE: SPIKE GLYCOPROTEIN;                                        3031:  6akm-A  3.4  2.2   46    47    9   MOLECULE: SUPPRESSOR OF IKBKE 1;                                     3032:  4n78-E  3.4  2.2   50    67    6   MOLECULE: CYTOPLASMIC FMR1-INTERACTING PROTEIN 1;                    3033:  7etm-A  3.4  7.3   79   498    5   MOLECULE: PORTAL PROTEIN;                                            3034:  7v2c-d  3.4  3.3   60   175    3   MOLECULE: NADH DEHYDROGENASE [UBIQUINONE] FLAVOPROTEIN 1,            3035:  5doq-B  3.4  4.2   64   330    9   MOLECULE: BD-TYPE QUINOL OXIDASE SUBUNIT I;                          3036:  1ysm-A  3.4  4.6   50    55    2   MOLECULE: CALCYCLIN-BINDING PROTEIN;                                 3037:  8szh-A  3.4  3.4   62   836    6   MOLECULE: EXTRACELLULAR CALCIUM-SENSING RECEPTOR;                    3038:  7vs4-C  3.4  7.0   71   374    4   MOLECULE: SITE-SPECIFIC DNA-METHYLTRANSFERASE (ADENINE-SPEC          3039:  3qh9-A  3.4  2.1   50    66    2   MOLECULE: LIPRIN-BETA-2;                                             3040:  3m0d-C  3.4  2.3   50    63   14   MOLECULE: TNF RECEPTOR-ASSOCIATED FACTOR 2;                          3041:  4dvy-P  3.4  2.7   63   656   10   MOLECULE: CYTOTOXICITY-ASSOCIATED IMMUNODOMINANT ANTIGEN;            3042:  6c0f-w  3.4  5.6   58    70    0   MOLECULE: SACCHAROMYCES CEREVISIAE S288C 35S PRE-RIBOSOMAL           3043:  2y1v-A  3.4  4.4   58   604    2   MOLECULE: CELL WALL SURFACE ANCHOR FAMILY PROTEIN;                   3044:  5vjx-H  3.4  3.5   48    51    2   MOLECULE: CLOCK-INTERACTING PACEMAKER;                               3045:  2ba2-A  3.4  3.3   50    81    6   MOLECULE: HYPOTHETICAL UPF0134 PROTEIN MPN010;                       3046:  6b5b-F  3.4  3.4   58    68   10   MOLECULE: BACULOVIRAL IAP REPEAT-CONTAINING PROTEIN 1E;              3047:  6vqi-I  3.4  2.5   50    64    2   MOLECULE: V-TYPE PROTON ATPASE SUBUNIT C 1;                          3048:  3o0z-D  3.4  1.4   50   160   10   MOLECULE: RHO-ASSOCIATED PROTEIN KINASE 1;                           3049:  2ehw-C  3.4  2.6   53   117    9   MOLECULE: HYPOTHETICAL PROTEIN TTHB059;                              3050:  6yhs-U  3.4  2.6   48    61   10   MOLECULE: 23S RIBOSOMAL RNA;                                         3051:  3mud-C  3.4  1.1   41    41    5   MOLECULE: DNA REPAIR PROTEIN XRCC4,TROPOMYOSIN ALPHA-1 CHAI          3052:  2m67-A  3.4  4.1   68    81    9   MOLECULE: MERF;                                                      3053:  1jek-A  3.4  0.9   40    40    3   MOLECULE: ENV POLYPROTEIN;                                           3054:  5hzi-A  3.4  3.4   80   476    9   MOLECULE: INTERSECTIN-1,NPH1-1,INTERSECTIN-1;                        3055:  2oqm-A  3.4  2.9   61   180   10   MOLECULE: HYPOTHETICAL PROTEIN;                                      3056:  4i5s-B  3.4  3.5   66   395    2   MOLECULE: PUTATIVE HISTIDINE KINASE COVS; VICK-LIKE PROTEIN          3057:  5h5v-A  3.4  3.1   63   352    6   MOLECULE: FLAGELLAR HOOK-ASSOCIATED PROTEIN 2;                       3058:  8pbz-A  3.4  3.8   72  1003   11   MOLECULE: MGP-OPERON PROTEIN 3;                                      3059:  7ewp-A  3.4  2.4   60   577    7   MOLECULE: PROBABLE G-PROTEIN COUPLED RECEPTOR 158;                   3060:  7c4o-A  3.4  2.4   43    48    7   MOLECULE: TRANSCRIPTION FACTOR HES-1;                                3061:  2yf3-C  3.4  2.8   57   153    7   MOLECULE: MAZG-LIKE NUCLEOSIDE TRIPHOSPHATE PYROPHOSPHOHYDR          3062:  7ekn-F  3.4  1.6   42    42    7   MOLECULE: IPEP;                                                      3063:  4u5a-B  3.4  5.4   73   184    4   MOLECULE: SPOROZOITE MICRONEME PROTEIN ESSENTIAL FOR CELL T          3064:  5ebz-L  3.4  2.7   83   655    7   MOLECULE: INHIBITOR OF NUCLEAR FACTOR KAPPA-B KINASE SUBUNI          3065:  6qhd-B  3.4  4.5   79   532    6   MOLECULE: SIGNAL TRANSDUCER AND ACTIVATOR OF TRANSCRIPTION           3066:  6xf1-C  3.4  3.3   75   224   11   MOLECULE: NESPRIN-2;                                                 3067:  6wg3-A  3.4 21.6   66   561    6   MOLECULE: STRUCTURAL MAINTENANCE OF CHROMOSOMES PROTEIN 1A;          3068:  6tms-L  3.4  3.5   61    69   11   MOLECULE: A NOVEL DESIGNED PORE PROTEIN;                             3069:  6xpd-A  3.4  4.2   71   303   11   MOLECULE: ZINC TRANSPORTER 8;                                        3070:  6bbf-N  3.4  2.0   55   150   13   MOLECULE: CALCIUM RELEASE-ACTIVATED CALCIUM CHANNEL PROTEIN          3071:  7f02-C  3.4  4.9   73   231    1   MOLECULE: CYTOCHROME C BIOGENESIS ATP-BINDING EXPORT PROTEI          3072:  3t6g-B  3.4  4.0   66   134    6   MOLECULE: SH2 DOMAIN-CONTAINING PROTEIN 3C;                          3073:  3cqz-A  3.4  6.1   67  1349    4   MOLECULE: DNA-DIRECTED RNA POLYMERASE II SUBUNIT RPB1;               3074:  6kos-A  3.4  2.6   65   101    2   MOLECULE: SUWA (SUPER WA20);                                         3075:  4dnd-A  3.4  3.0   58    95   14   MOLECULE: SYNTAXIN-10;                                               3076:  8j7u-B  3.4  2.7   57   286    7   MOLECULE: ZINC TRANSPORTER 7;                                        3077:  7uqi-G  3.4 10.5   82   197   12   MOLECULE: ATPASE HISTONE CHAPERONE YTA7;                             3078:  6rdl-7  3.4  5.7   72   176    8   MOLECULE: ASA-10: POLYTOMELLA F-ATP SYNTHASE ASSOCIATED SUB          3079:  5flz-A  3.4  4.8   73   575    5   MOLECULE: SPINDLE POLE BODY COMPONENT SPC97;                         3080:  7m2w-F  3.4  3.1   78   674    4   MOLECULE: TUBULIN GAMMA CHAIN;                                       3081:  2dnx-A  3.4  2.6   67   130   13   MOLECULE: SYNTAXIN-12;                                               3082:  6o7v-a  3.4  4.1   71   625    6   MOLECULE: V-TYPE PROTON ATPASE SUBUNIT D;                            3083:  1uur-A  3.4  2.4   64   461    9   MOLECULE: STATA PROTEIN;                                             3084:  6ar7-A  3.4  4.4   59   205   10   MOLECULE: UNCHARACTERIZED PROTEIN;                                   3085:  2ib0-A  3.4  2.8   58   142    7   MOLECULE: CONSERVED HYPOTHETICAL ALANINE RICH PROTEIN;               3086:  6jzr-a  3.4  3.4   70   247    4   MOLECULE: FLAGELLAR BASAL-BODY ROD PROTEIN FLGG;                     3087:  3kdq-B  3.4  6.9   70   152    6   MOLECULE: UNCHARACTERIZED CONSERVED PROTEIN;                         3088:  5ww3-A  3.4  3.9   64   161    8   MOLECULE: PUTATIVE STARVATION-INDUCED DNA PROTECTING PROTEI          3089:  5xhi-A  3.4  3.5   65   174    8   MOLECULE: FERRITIN, MIDDLE SUBUNIT;                                  3090:  5tin-C  3.4  4.1   62   339    5   MOLECULE: GLYCINE RECEPTOR SUBUNIT ALPHA-3;                          3091:  4x5m-B  3.4  2.5   52    93    6   MOLECULE: UNCHARACTERIZED PROTEIN;                                   3092:  1lih-A  3.4  3.0   71   160    3   MOLECULE: ASPARTATE RECEPTOR;                                        3093:  3i9y-A  3.4  2.5   79   229    9   MOLECULE: SENSOR PROTEIN;                                            3094:  4z9g-B  3.4  3.0   64   415    8   MOLECULE: CORTICOTROPIN-RELEASING FACTOR RECEPTOR 1,LYSOZYM          3095:  5cd4-I  3.4  3.2   53   494    9   MOLECULE: CRISPR SYSTEM CASCADE SUBUNIT CASE;                        3096:  6zkt-Z  3.4  2.8   60   171    8   MOLECULE: NADH DEHYDROGENASE [UBIQUINONE] FLAVOPROTEIN 1,            3097:  2bcc-C  3.4  3.4   54   379   15   MOLECULE: UBIQUINOL CYTOCHROME C OXIDOREDUCTASE;                     3098:  7r41-p  3.4  2.8   60   171    8   MOLECULE: NADH-UBIQUINONE OXIDOREDUCTASE CHAIN 3;                    3099:  3r9v-B  3.4  5.2   77   163    3   MOLECULE: INVASIN IPAD;                                              3100:  7wu5-R  3.4  2.5   61   286    3   MOLECULE: GUANINE NUCLEOTIDE-BINDING PROTEIN G(I) SUBUNIT A          3101:  6ucb-G  3.4  4.0   67   132    7   MOLECULE: GLUTAMATE RECEPTOR 2;                                      3102:  8vjn-A  3.4  3.5   50    52   14   MOLECULE: ENCAPSULIN NANOCOMPARTMENT CARGO PROTEIN ENCD;             3103:  7tap-O  3.4  3.3   55    69    7   MOLECULE: V-TYPE PROTON ATPASE SUBUNIT C';                           3104:  2gta-A  3.4  3.3   58    98    5   MOLECULE: HYPOTHETICAL PROTEIN YPJD;                                 3105:  6rdk-7  3.4  5.7   72   176    8   MOLECULE: ASA-10: POLYTOMELLA F-ATP SYNTHASE ASSOCIATED SUB          3106:  6ret-7  3.4  6.0   71   176   10   MOLECULE: ASA-10: POLYTOMELLA F-ATP SYNTHASE ASSOCIATED SUB          3107:  6res-7  3.4  6.0   71   176   10   MOLECULE: ASA-10: POLYTOMELLA F-ATP SYNTHASE ASSOCIATED SUB          3108:  7y3f-2  3.4  3.7   67   302    0   MOLECULE: PHOTOSYSTEM I P700 CHLOROPHYLL A APOPROTEIN A1;            3109:  6rep-7  3.4  5.7   72   176    8   MOLECULE: ASA-10: POLYTOMELLA F-ATP SYNTHASE ASSOCIATED SUB          3110:  5an6-A  3.4  4.4   60   123    8   MOLECULE: CRISPR-ASSOCIATED PROTEIN, CSM2 FAMILY;                    3111:  4rkm-A  3.4  1.9   45   659   11   MOLECULE: MCCA;                                                      3112:  5ofp-A  3.4  3.5   80   571    6   MOLECULE: MICROCIN-J25 EXPORT ATP-BINDING/PERMEASE PROTEIN           3113:  8u29-C  3.4  3.7   70  1082    7   MOLECULE: PRD-0038 SPIKE GLYCOPROTEIN;                               3114:  7zh1-C  3.4  3.7   68  1020    4   MOLECULE: SPIKE GLYCOPROTEIN,FIBRITIN;                               3115:  6x80-E  3.4  3.3   63   574    3   MOLECULE: FLAGELLIN A;                                               3116:  6wtv-A  3.4  4.0   66   299    2   MOLECULE: RETICULOCYTE BINDING PROTEIN 2B;                           3117:  8u29-A  3.4  3.7   70  1082    7   MOLECULE: PRD-0038 SPIKE GLYCOPROTEIN;                               3118:  5gom-B  3.4  2.9   71   404    4   MOLECULE: MITOFUSIN-1;                                               3119:  6wty-G  3.4  2.5   75   290    5   MOLECULE: RETICULOCYTE BINDING PROTEIN 2B;                           3120:  5zvm-C  3.4  2.2   50    67    6   MOLECULE: SPIKE GLYCOPROTEIN;                                        3121:  6wtu-G  3.4  4.7   71   297    6   MOLECULE: RETICULOCYTE BINDING PROTEIN 2B;                           3122:  1y4m-B  3.3  1.9   47    53    4   MOLECULE: HERV-FRD_6P24.1 PROVIRUS ANCESTRAL ENV POLYPROTEI          3123:  6its-A  3.3  2.1   60   147   10   MOLECULE: METHYL-ACCEPTING CHEMOTAXIS SENSORY TRANSDUCER;            3124:  5dfz-A  3.3  2.9   49   343    6   MOLECULE: VACUOLAR PROTEIN SORTING-ASSOCIATED PROTEIN 38;            3125:  3b5n-C  3.3  2.2   50    70   12   MOLECULE: SYNAPTOBREVIN HOMOLOG 1;                                   3126:  2xeq-C  3.3  4.1   60   241    2   MOLECULE: PAT1 HOMOLOG 1,;                                           3127:  1fbm-A  3.3  1.8   45    46    2   MOLECULE: PROTEIN (CARTILAGE OLIGOMERIC MATRIX PROTEIN);             3128:  7jhj-R  3.3  2.4   57   277   11   MOLECULE: GUANINE NUCLEOTIDE-BINDING PROTEIN G(I) SUBUNIT A          3129:  2k73-A  3.3  5.5   68   183    3   MOLECULE: DISULFIDE BOND FORMATION PROTEIN B;                        3130:  5wp4-A  3.3  4.0   52   487   12   MOLECULE: PHOSPHOETHANOLAMINE N-METHYLTRANSFERASE 1;                 3131:  5jvp-B  3.3  3.4   57    90    5   MOLECULE: CHIMERA PROTEIN OF CENTROMERE-ASSOCIATED PROTEIN           3132:  5we2-A  3.3  3.7   66   229    6   MOLECULE: PROTECTION OF TELOMERES PROTEIN POZ1;                      3133:  7vpx-J  3.3  2.0   47    54    4   MOLECULE: SPLICING FACTOR 3A SUBUNIT 2;                              3134:  8au0-B  3.3  1.4   41    41   10   MOLECULE: SUN DOMAIN-CONTAINING PROTEIN 1;                           3135:  6ec8-A  3.3  4.5   82   803    7   MOLECULE: LANTIBIOTIC DEHYDRATASE DOMAIN PROTEIN;                    3136:  8php-A  3.3  3.5   68   325    7   MOLECULE: PHAGE PORTAL PROTEIN;                                      3137:  7yyg-A  3.3  2.6   44   275    9   MOLECULE: PUTATIVE OUTER PROTEIN N;                                  3138:  8eki-C  3.3  3.7   57   701   12   MOLECULE: PROTEIN TRANSPORT PROTEIN SEC20;                           3139:  6nf1-A  3.3  3.6   78   550    5   MOLECULE: PROTO-ONCOGENE VAV;                                        3140:  3ub0-D  3.3  3.1   58   194    9   MOLECULE: NON-STRUCTURAL PROTEIN 6, NSP6,;                           3141:  7wb4-I  3.3  2.5   65   796    3   MOLECULE: OUTER NUP133;                                              3142:  5n0c-A  3.3  6.0   66  1293    5   MOLECULE: TETANUS TOXIN;                                             3143:  3jbz-A  3.3  3.5   53   960   11   MOLECULE: SERINE/THREONINE-PROTEIN KINASE MTOR;                      3144:  7x0a-B  3.3  4.6   67   429    4   MOLECULE: T-COMPLEX PROTEIN 1 SUBUNIT ALPHA;                         3145:  4xvx-B  3.3  5.6   65   373   11   MOLECULE: ACYL-[ACYL-CARRIER-PROTEIN] DEHYDROGENASE MBTN;            3146:  7yfu-A  3.3  2.1   44    44    7   MOLECULE: PROTEIN FANTOM;                                            3147:  6s18-A  3.3  2.2   60   143    3   MOLECULE: AROMATIC ACID CHEMORECEPTOR;                               3148:  2kes-A  3.3  1.9   46    48   13   MOLECULE: SYNPHILIN-1;                                               3149:  6n63-A  3.3  3.6   71   141    0   MOLECULE: ENCAPSULIN CARGO PROTEIN;                                  3150:  7xgr-A  3.3  4.7   57   546    2   MOLECULE: GEM-ASSOCIATED PROTEIN 5;                                  3151:  7bin-V  3.3  2.6   61   133    7   MOLECULE: FLAGELLAR BIOSYNTHETIC PROTEIN FLIP;                       3152:  5hud-E  3.3  2.3   51    84    6   MOLECULE: 3-DEOXY-D-ARABINO-HEPTULOSONATE 7-PHOSPHATE (DAHP          3153:  8qt5-A  3.3  2.4   60   248    5   MOLECULE: 14-3-3-LIKE PROTEIN G-BOX FACTOR 14 LAMBDA,PROTEI          3154:  1upg-B  3.3  3.9   64    99   11   MOLECULE: TRANSCRIPTIONAL REPRESSOR TRAM;                            3155:  4e61-A  3.3  3.1   57    91    7   MOLECULE: PROTEIN BIM1;                                              3156:  6n6s-D  3.3  2.2   50    69   10   MOLECULE: TNFAIP3-INTERACTING PROTEIN 1;                             3157:  7yxx-A  3.3  5.7   60  1754    7   MOLECULE: PROBABLE UBIQUITIN CARBOXYL-TERMINAL HYDROLASE FA          3158:  8gyw-A  3.3  4.0   61   380   10   MOLECULE: CHOLINE/ETHANOLAMINEPHOSPHOTRANSFERASE 1;                  3159:  7b9f-D  3.3  3.8   64   485    6   MOLECULE: ECCE5;                                                     3160:  7obq-u  3.3  6.4   67   441    9   MOLECULE: SRP RNA;                                                   3161:  6gmh-Q  3.3  4.7   51   884    0   MOLECULE: RPB1;                                                      3162:  6xz4-A  3.3  2.4   62   304   11   MOLECULE: TALIN ROD DOMAIN-CONTAINING PROTEIN 1;                     3163:  8soi-C  3.3  4.3   73   205    7   MOLECULE: RB1-INDUCIBLE COILED-COIL PROTEIN 1;                       3164:  6un9-A  3.3  1.7   50    69   12   MOLECULE: UNCHARACTERIZED PROTEIN;                                   3165:  7mvx-A  3.3  2.5   47  1645    9   MOLECULE: NUCLEOPORIN NUP188;                                        3166:  4fz4-A  3.3  3.0   59   154    8   MOLECULE: UNCHARACTERIZED PROTEIN CONSERVED IN BACTERIA;             3167:  5mmj-t  3.3  3.2   62   107    2   MOLECULE: 50S RIBOSOMAL PROTEIN L31;                                 3168:  7vzg-a  3.3  2.8   71   858    8   MOLECULE: PSCA;                                                      3169:  8hir-A  3.3  4.7   57   918    5   MOLECULE: POTASSIUM CHANNEL SUBFAMILY T MEMBER 1;                    3170:  6otn-B  3.3  2.8   50    67    8   MOLECULE: TROPOMYOSIN ALPHA-3 CHAIN;                                 3171:  1lj2-B  3.3  5.7   53   110    4   MOLECULE: NONSTRUCTURAL RNA-BINDING PROTEIN 34;                      3172:  7edp-B  3.3  1.5   40    40   10   MOLECULE: HISTONE-LYSINE N-METHYLTRANSFERASE, H3 LYSINE-79           3173:  7qj0-L  3.3  3.4   53   566    6   MOLECULE: MITOTIC-SPINDLE ORGANIZING PROTEIN 1;                      3174:  6qrj-A  3.3  2.9   56   463   11   MOLECULE: HYBRID KINASE;                                             3175:  4eyy-R  3.3  3.8   51    59    6   MOLECULE: ICMR;                                                      3176:  4l2w-D  3.3  2.3   50    68   12   MOLECULE: RHO-ASSOCIATED PROTEIN KINASE 1;                           3177:  2ocy-A  3.3  2.1   50   149   12   MOLECULE: RAB GUANINE NUCLEOTIDE EXCHANGE FACTOR SEC2;               3178:  3m9b-A  3.3  1.4   44   186   23   MOLECULE: PROTEASOME-ASSOCIATED ATPASE;                              3179:  8g52-A  3.3  2.9   42   279    7   MOLECULE: TPR_REGION DOMAIN-CONTAINING PROTEIN;                      3180:  7kzn-C  3.3  2.8   52   965    4   MOLECULE: HEAVY CHAIN ALPHA;                                         3181:  3kdw-A  3.3  4.6   66   206    5   MOLECULE: PUTATIVE SUGAR BINDING PROTEIN;                            3182:  4aur-A  3.3  4.2   82   564   11   MOLECULE: LEOA;                                                      3183:  4it4-B  3.3  3.5   56   210    7   MOLECULE: CG17282;                                                   3184:  7b93-L  3.3  2.5   51   607    6   MOLECULE: NADH-UBIQUINONE OXIDOREDUCTASE CHAIN 3;                    3185:  5l25-A  3.3  4.1   63   399    6   MOLECULE: BORON TRANSPORTER 1;                                       3186:  4w8j-A  3.3  3.1   63  1017    5   MOLECULE: PESTICIDAL CRYSTAL PROTEIN CRY1AC;                         3187:  5v8k-A  3.3  3.4   62   600    3   MOLECULE: P800 REACTION CENTER CORE PROTEIN;                         3188:  8f5p-D  3.3  3.8   66   612    3   MOLECULE: NET DOMAIN-CONTAINING PROTEIN;                             3189:  1r0d-A  3.3  3.9   76   194    9   MOLECULE: HUNTINGTIN INTERACTING PROTEIN 12;                         3190:  4bem-J  3.3  5.7   77   182    6   MOLECULE: F1FO ATPASE C2 SUBUNIT;                                    3191:  5a3f-C  3.3  7.4   72   700    3   MOLECULE: DYNAMIN 3;                                                 3192:  2xra-A  3.3  3.0   70   203    7   MOLECULE: TRANSMEMBRANE PROTEIN GP41;                                3193:  3fks-G  3.3  2.9   70   268    4   MOLECULE: ATP SYNTHASE SUBUNIT ALPHA, MITOCHONDRIAL;                 3194:  2oex-A  3.3  4.0   74   342    3   MOLECULE: PROGRAMMED CELL DEATH 6-INTERACTING PROTEIN;               3195:  8txr-f  3.3  3.3   50    59    4   MOLECULE: EXODEOXYRIBONUCLEASE 7 LARGE SUBUNIT;                      3196:  7zw6-D  3.3  3.2   73   793   12   MOLECULE: SLR0869 PROTEIN;                                           3197:  6ixr-A  3.3  2.6   70   388    6   MOLECULE: MYOSIN-2;                                                  3198:  4nq0-A  3.3  2.9   51   254   14   MOLECULE: HAT1-INTERACTING FACTOR 1;                                 3199:  4xng-B  3.3  3.0   71   137    6   MOLECULE: UNCHARACTERIZED PROTEIN MG218.1;                           3200:  4xng-C  3.3  2.9   68   138    6   MOLECULE: UNCHARACTERIZED PROTEIN MG218.1;                           3201:  3swh-A  3.3  2.6   62   295    6   MOLECULE: PROTEIN UNC-13 HOMOLOG A;                                  3202:  6bbf-E  3.3  2.0   55   150   13   MOLECULE: CALCIUM RELEASE-ACTIVATED CALCIUM CHANNEL PROTEIN          3203:  5n5e-J  3.3  4.9   60    98    7   MOLECULE: PFC_05175;                                                 3204:  8p3s-H  3.3  2.7   63   177    6   MOLECULE: GLUTAMATE RECEPTOR 2;                                      3205:  8tnb-C  3.3  5.7   69   147    9   MOLECULE: DE NOVO DESIGNED PROTEIN;                                  3206:  5lsk-A  3.3  6.2   74   199   11   MOLECULE: PROTEIN MIS12 HOMOLOG;                                     3207:  5n5e-B  3.3  4.9   60    98    7   MOLECULE: PFC_05175;                                                 3208:  3fd9-C  3.3  3.1   68   239    9   MOLECULE: UNCHARACTERIZED PROTEIN;                                   3209:  6mhy-A  3.3  3.8   60   177    2   MOLECULE: GAP JUNCTION ALPHA-8 PROTEIN;                              3210:  3afe-A  3.3  4.2   64   384    5   MOLECULE: HYDROXYLASE, PUTATIVE;                                     3211:  6dfk-M  3.3  3.3   76   213    7   MOLECULE: SUBUNIT OF PROTEASEOME ACTIVATOR COMPLEX,PUTATIVE          3212:  5n5e-d  3.3  4.9   60    97    7   MOLECULE: PFC_05175;                                                 3213:  7o4i-W  3.3  5.1   68   312    9   MOLECULE: GENERAL TRANSCRIPTION AND DNA REPAIR FACTOR IIH H          3214:  3cfo-A  3.3  3.0   77   906    5   MOLECULE: DNA POLYMERASE;                                            3215:  7qyd-B  3.3  5.7   70   630    7   MOLECULE: PESTICIDAL CRYSTAL PROTEIN CRY11BA;                        3216:  1qvx-A  3.3  3.5   66   134    8   MOLECULE: FOCAL ADHESION KINASE 1;                                   3217:  7b5n-H  3.3  3.1   61   423    5   MOLECULE: CULLIN-1;                                                  3218:  4rsi-B  3.3  3.2   85   386    4   MOLECULE: STRUCTURAL MAINTENANCE OF CHROMOSOMES PROTEIN 2;           3219:  6wtd-O  3.3  4.1   65    74    3   MOLECULE: ATP SYNTHASE SUBUNIT 9, MITOCHONDRIAL;                     3220:  5tj5-A  3.3  3.3   76   570    9   MOLECULE: V-TYPE PROTON ATPASE SUBUNIT A;                            3221:  6j9r-B  3.3  2.3   64   126    3   MOLECULE: BRAIN TUMOR PROTEIN;                                       3222:  6hum-F  3.3  5.4   56   611    0   MOLECULE: NAD(P)H-QUINONE OXIDOREDUCTASE SUBUNIT 1;                  3223:  3rko-B  3.3  2.6   52   612    8   MOLECULE: NADH-QUINONE OXIDOREDUCTASE SUBUNIT L;                     3224:  4p3g-A  3.3  2.8   62   219    8   MOLECULE: SIGNAL RECOGNITION PARTICLE SUBUNIT SRP68;                 3225:  5arm-A  3.3  2.3   57   127    4   MOLECULE: CSP3;                                                      3226:  5ywb-B  3.3  4.9   60  1322    5   MOLECULE: ATP-SENSITIVE INWARD RECTIFIER POTASSIUM CHANNEL           3227:  2jaa-A  3.3  5.2   76   167    3   MOLECULE: INVASIN IPAD;                                              3228:  6c26-2  3.3  6.7   62   108    6   MOLECULE: DOLICHYL-DIPHOSPHOOLIGOSACCHARIDE--PROTEIN                 3229:  6zni-B  3.3  3.7   63    82    8   MOLECULE: PROTEIN MXIH;                                              3230:  6zni-F  3.3  3.7   63    82    8   MOLECULE: PROTEIN MXIH;                                              3231:  6zni-S  3.3  3.7   63    82    8   MOLECULE: PROTEIN MXIH;                                              3232:  6rec-7  3.3  5.7   72   176    8   MOLECULE: ASA-10: POLYTOMELLA F-ATP SYNTHASE ASSOCIATED SUB          3233:  4h8s-C  3.3  2.6   80   382    8   MOLECULE: DCC-INTERACTING PROTEIN 13-BETA;                           3234:  7k7k-Z  3.3  4.1   77   171    8   MOLECULE: TRANSLOCON ESPA;                                           3235:  7k7k-V  3.3  4.1   77   171    8   MOLECULE: TRANSLOCON ESPA;                                           3236:  7k7k-T  3.3  4.1   77   171    8   MOLECULE: TRANSLOCON ESPA;                                           3237:  7k7k-D  3.3  4.1   77   171    8   MOLECULE: TRANSLOCON ESPA;                                           3238:  7k7k-A  3.3  4.1   77   171    8   MOLECULE: TRANSLOCON ESPA;                                           3239:  6sqw-A  3.3  3.7   57   114    5   MOLECULE: DCTP PYROPHOSPHATASE 1;                                    3240:  3csx-B  3.3  3.2   58    71   17   MOLECULE: PUTATIVE UNCHARACTERIZED PROTEIN;                          3241:  1ser-B  3.3  5.2   69   421    9   MOLECULE: TRNASER;                                                   3242:  6reb-7  3.3  5.7   72   176    8   MOLECULE: ASA-10: POLYTOMELLA F-ATP SYNTHASE ASSOCIATED SUB          3243:  6uh5-X  3.3  3.0   62    73    2   MOLECULE: HISTONE H3;                                                3244:  3pdy-B  3.3  2.9   74   205    9   MOLECULE: PLECTIN;                                                   3245:  6lp3-A  3.3  4.8   73   118   11   MOLECULE: UNCHARACTERIZED PROTEIN YMR124W;                           3246:  6qlf-Z  3.3  3.4   50   140    8   MOLECULE: INNER KINETOCHORE SUBUNIT IML3;                            3247:  6zni-C  3.3  3.7   63    82    8   MOLECULE: PROTEIN MXIH;                                              3248:  8eau-f  3.3  3.2   54    69    7   MOLECULE: V-TYPE PROTON ATPASE SUBUNIT A, VACUOLAR ISOFORM;          3249:  6zni-N  3.3  3.7   63    82    8   MOLECULE: PROTEIN MXIH;                                              3250:  7sg4-A  3.3  3.7   70  1095    6   MOLECULE: SPIKE GLYCOPROTEIN;                                        3251:  5zvm-A  3.3  3.0   50    69    2   MOLECULE: SPIKE GLYCOPROTEIN;                                        3252:  1ztm-A  3.3  2.5   54   416   11   MOLECULE: FUSION GLYCOPROTEIN;                                       3253:  5wjt-c  3.3  2.9   83   302   13   MOLECULE: FLAGELLIN;                                                 3254:  8dyu-A  3.3  3.8   70  2562   10   MOLECULE: CYTOPLASMIC DYNEIN 1 HEAVY CHAIN 1;                        3255:  6cs2-A  3.3  3.6   69   891    6   MOLECULE: SPIKE GLYCOPROTEIN,FIBRITIN;                               3256:  2c08-A  3.3  3.2   78   204    6   MOLECULE: SH3-CONTAINING GRB2-LIKE PROTEIN 2;                        3257:  7sg4-C  3.3  3.7   70  1095    6   MOLECULE: SPIKE GLYCOPROTEIN;                                        3258:  8u29-B  3.3  3.7   70  1082    7   MOLECULE: PRD-0038 SPIKE GLYCOPROTEIN;                               3259:  8fa1-B  3.3  1.9   50    71    6   MOLECULE: FERRITIN, DPS FAMILY PROTEIN AND SPIKE PROTEIN S2          3260:  7jh5-B  3.3  3.6   66   279    9   MOLECULE: CO-LOCKR: DE NOVO DESIGNED PROTEIN SWITCH;                 3261:  6wv9-A  3.2  4.6   63   411    6   MOLECULE: VITAMIN K EPOXIDE REDUCTASE-LIKE PROTEIN, TERMINI          3262:  7k36-D  3.2  2.2   50    74    6   MOLECULE: SERINE/THREONINE-PROTEIN PHOSPHATASE 2A 65 KDA RE          3263:  1oxz-A  3.2  3.7   68   132    6   MOLECULE: ADP-RIBOSYLATION FACTOR BINDING PROTEIN GGA1;              3264:  5cwt-A  3.2  1.5   41    41   12   MOLECULE: NUCLEOPORIN NUP57;                                         3265:  1vcs-A  3.2  3.7   59   102    8   MOLECULE: VESICLE TRANSPORT THROUGH INTERACTION WITH T-              3266:  2oqq-A  3.2  2.5   42    42    7   MOLECULE: TRANSCRIPTION FACTOR HY5;                                  3267:  8j01-A  3.2  3.1   62   354    8   MOLECULE: POTASSIUM VOLTAGE-GATED CHANNEL SUBFAMILY KQT MEM          3268:  4x01-C  3.2  2.9   46    50    7   MOLECULE: DNA BINDING CTP1;                                          3269:  3e98-A  3.2  4.8   56   178    7   MOLECULE: GAF DOMAIN OF UNKNOWN FUNCTION;                            3270:  4gdo-A  3.2  2.2   42    42   10   MOLECULE: PLECTIN;                                                   3271:  4axz-A  3.2  3.0   69   218    6   MOLECULE: PUTATIVE ANTIGEN P35;                                      3272:  6tdv-E  3.2  3.2   57    96    4   MOLECULE: ATPTB1;                                                    3273:  3jv3-A  3.2  3.7   79   278    8   MOLECULE: INTERSECTIN-1;                                             3274:  4pas-B  3.2  1.0   39    39    5   MOLECULE: GAMMA-AMINOBUTYRIC ACID TYPE B RECEPTOR SUBUNIT 1          3275:  5xiu-A  3.2  3.8   45    47    7   MOLECULE: E3 UBIQUITIN-PROTEIN LIGASE RNF168;                        3276:  5f5t-C  3.2  4.1   53    64    4   MOLECULE: PUTATIVE UNCHARACTERIZED PROTEIN;                          3277:  6w1s-W  3.2  2.9   56   118    7   MOLECULE: MEDIATOR OF RNA POLYMERASE II TRANSCRIPTION SUBUN          3278:  2xqh-A  3.2  2.5   52   258    8   MOLECULE: IMMUNOGLOBULIN-BINDING PROTEIN EIBD;                       3279:  7wae-A  3.2  3.4   50   877   10   MOLECULE: CYANOPHYCIN SYNTHASE;                                      3280:  7bw0-R  3.2  4.6   70   280   11   MOLECULE: SOLUBLE CYTOCHROME B562,G-PROTEIN COUPLED BILE AC          3281:  8fnu-A  3.2  3.9   56   796    5   MOLECULE: KAP NTPASE DOMAIN-CONTAINING PROTEIN;                      3282:  3rk3-E  3.2  2.9   47    50    2   MOLECULE: VAMP2;                                                     3283:  6zmq-A  3.2  2.2   60   589    8   MOLECULE: CYTOCHROME C-TYPE BIOGENESIS PROTEIN CCMF;                 3284:  5eof-A  3.2  1.9   50    74    4   MOLECULE: OPTINEURIN;                                                3285:  7a48-B  3.2  1.9   40    40   15   MOLECULE: NANOBODY 49;                                               3286:  1fou-A  3.2  2.9   70   257    4   MOLECULE: UPPER COLLAR PROTEIN;                                      3287:  6lp3-C  3.2  1.6   43    43    9   MOLECULE: UNCHARACTERIZED PROTEIN YMR124W;                           3288:  4k1p-B  3.2  2.8   85   346    5   MOLECULE: NHEA;                                                      3289:  6o9l-3  3.2  3.4   63   309   10   MOLECULE: DNA-DIRECTED RNA POLYMERASE II SUBUNIT RPB1;               3290:  7qtt-Y  3.2  2.5   49   306    4   MOLECULE: SPLICING FACTOR 3B SUBUNIT 3;                              3291:  8bef-u  3.2  3.0   50    55    4   MOLECULE: NADH-UBIQUINONE OXIDOREDUCTASE CHAIN 3;                    3292:  7o97-A  3.2  2.4   50    73    0   MOLECULE: HYPOTHETICAL PROTEIN UY81_C0065G0003 FROM CANDIDA          3293:  6yip-A  3.2  2.4   50    71   14   MOLECULE: KINESIN-LIKE PROTEIN KIF20A;                               3294:  5xpd-A  3.2  2.6   52   269    8   MOLECULE: SUGAR TRANSPORTER;                                         3295:  6hu9-c  3.2  3.4   64   269    3   MOLECULE: CYTOCHROME B-C1 COMPLEX SUBUNIT 1, MITOCHONDRIAL;          3296:  8h9d-A  3.2  4.1   63  1114   10   MOLECULE: CAS12A;                                                    3297:  2c5i-T  3.2  3.2   62    94   10   MOLECULE: T-SNARE AFFECTING A LATE GOLGI COMPARTMENT                 3298:  7bjs-A  3.2  1.6   50    69    2   MOLECULE: KINESIN HEAVY CHAIN;                                       3299:  2mpn-A  3.2  4.0   54    68    7   MOLECULE: INNER MEMBRANE PROTEIN YGAP;                               3300:  5j4i-A  3.2  2.5   57   437    7   MOLECULE: ARGININE/AGMATINE ANTIPORTER;                              3301:  2oku-A  3.2  3.9   59   122   14   MOLECULE: ACYL-COA DEHYDROGENASE FAMILY PROTEIN;                     3302:  6vls-D  3.2  4.3   54   963   15   MOLECULE: MALTOSE/MALTODEXTRIN-BINDING PERIPLASMIC PROTEIN,          3303:  2gzh-B  3.2  2.7   51    57   10   MOLECULE: RAS-RELATED PROTEIN RAB-11A;                               3304:  3wvo-A  3.2  3.6   57   543   11   MOLECULE: CRISPR-ASSOCIATED PROTEIN, CSE1 FAMILY;                    3305:  4nqf-B  3.2  3.6   69   151    7   MOLECULE: NUCLEOTIDYLTRANSFERASE;                                    3306:  3rkl-A  3.2  2.5   52    80    6   MOLECULE: STIV-A81;                                                  3307:  7ar9-K  3.2  2.9   55   104    9   MOLECULE: ND3;                                                       3308:  7stw-A  3.2  3.8   61   170    7   MOLECULE: DNA PROTECTION DURING STARVATION PROTEIN;                  3309:  8jw0-b  3.2  5.1   68   617    3   MOLECULE: PHOTOSYSTEM I PSAA;                                        3310:  6ksa-B  3.2  2.6   65   611    5   MOLECULE: ACYL-COA DEHYDROGENASE;                                    3311:  8q85-c  3.2  2.6   50    64   14   MOLECULE: KINETOCHORE PROTEIN NDC80;                                 3312:  8ptx-A  3.2  3.4   75  1215    7   MOLECULE: ELONGATOR COMPLEX PROTEIN 1;                               3313:  6snr-A  3.2  3.9   68   402    3   MOLECULE: LIPID II:GLYCINE GLYCYLTRANSFERASE;                        3314:  8fuq-A  3.2  4.4   47    51    6   MOLECULE: PHOSPHOPROTEIN;                                            3315:  8txr-e  3.2  3.3   51    61    4   MOLECULE: EXODEOXYRIBONUCLEASE 7 LARGE SUBUNIT;                      3316:  8txr-n  3.2  2.8   48    59    4   MOLECULE: EXODEOXYRIBONUCLEASE 7 LARGE SUBUNIT;                      3317:  1r5i-D  3.2  4.5   56   214    4   MOLECULE: HLA CLASS II HISTOCOMPATIBILITY ANTIGEN, DR                3318:  8txr-d  3.2  3.1   51    60    4   MOLECULE: EXODEOXYRIBONUCLEASE 7 LARGE SUBUNIT;                      3319:  7pvk-A  3.2  2.7   51   513    8   MOLECULE: RESPONSE REGULATOR;                                        3320:  8txr-l  3.2  3.2   50    59    4   MOLECULE: EXODEOXYRIBONUCLEASE 7 LARGE SUBUNIT;                      3321:  7zw6-B  3.2  3.2   73   793   12   MOLECULE: SLR0869 PROTEIN;                                           3322:  7ssp-E  3.2  3.2   63   165    6   MOLECULE: UBIQUINONE BIOSYNTHESIS PROTEIN COQ9, MITOCHONDRI          3323:  4q5v-A  3.2  3.7   60   865   13   MOLECULE: DNA POLYMERASE ALPHA CATALYTIC SUBUNIT;                    3324:  3q0k-D  3.2  4.7   81   289    6   MOLECULE: PROTEIN KINASE C AND CASEIN KINASE SUBSTRATE IN N          3325:  6dfk-C  3.2  3.0   79   222    5   MOLECULE: SUBUNIT OF PROTEASEOME ACTIVATOR COMPLEX,PUTATIVE          3326:  6yf5-B  3.2  3.6   55    83   11   MOLECULE: PRELAMIN-A/C,MICROTUBULE-ASSOCIATED PROTEIN RP/EB          3327:  6yf5-D  3.2  3.3   54    82   11   MOLECULE: PRELAMIN-A/C,MICROTUBULE-ASSOCIATED PROTEIN RP/EB          3328:  7nvz-W  3.2 13.5   70   202    7   MOLECULE: TFIIH BASAL TRANSCRIPTION FACTOR COMPLEX HELICASE          3329:  4i0u-B  3.2  3.5   72   348   13   MOLECULE: MAGNESIUM TRANSPORT PROTEIN CORA;                          3330:  7qhm-V  3.2  3.3   53   135    9   MOLECULE: CYTOCHROME BC1 COMPLEX RIESKE IRON-SULFUR SUBUNIT          3331:  7yn1-B  3.2  2.1   52   348    8   MOLECULE: CCBD;                                                      3332:  2iiu-A  3.2  2.9   53   208    4   MOLECULE: HYPOTHETICAL PROTEIN;                                      3333:  7enj-D  3.2  2.8   56   158    5   MOLECULE: MEDIATOR OF RNA POLYMERASE II TRANSCRIPTION SUBUN          3334:  7vg4-E  3.2  4.3   73   196    4   MOLECULE: METHENYLTETRAHYDROFOLATE CYCLOHYDROLASE;                   3335:  3sog-A  3.2  4.4   79   197    4   MOLECULE: AMPHIPHYSIN;                                               3336:  1uru-A  3.2  2.9   80   217    3   MOLECULE: AMPHIPHYSIN;                                               3337:  6nyk-B  3.2  2.5   56    90    5   MOLECULE: DESIGN CONSTRUCT XAX_GGDQ;                                 3338:  1qjb-B  3.2  3.7   62   232    6   MOLECULE: 14-3-3 PROTEIN ZETA/DELTA;                                 3339:  1i1r-B  3.2  1.9   57   167    9   MOLECULE: INTERLEUKIN-6 RECEPTOR BETA CHAIN;                         3340:  6zni-M  3.2  3.7   63    82    6   MOLECULE: PROTEIN MXIH;                                              3341:  4d10-A  3.2  2.5   50   419    4   MOLECULE: COP9 SIGNALOSOME COMPLEX SUBUNIT 1;                        3342:  6nbq-G  3.2  2.0   49   191   10   MOLECULE: NAD(P)H-QUINONE OXIDOREDUCTASE SUBUNIT H;                  3343:  6ww2-R  3.2  2.2   43   425   12   MOLECULE: ANTI-BRIL FAB HEAVY CHAIN;                                 3344:  1jnv-Z  3.2  2.7   83   273    0   MOLECULE: ATP SYNTHASE ALPHA CHAIN;                                  3345:  6xt9-I  3.2  3.2   82   767    9   MOLECULE: BARDET-BIEDL SYNDROME 1 PROTEIN;                           3346:  8dql-A  3.2  3.3   56   257   11   MOLECULE: SECRETION SYSTEM PROTEIN;                                  3347:  6t5a-A  3.2  5.0   58    96    5   MOLECULE: TEGUMENT PROTEIN UL51;                                     3348:  6r6n-A  3.2  3.0   54   111    7   MOLECULE: SMALL SOLUBLE CYT C;                                       3349:  3gm1-B  3.2  2.6   64   141    8   MOLECULE: PROTEIN TYROSINE KINASE 2 BETA;                            3350:  6iol-L  3.2  3.9   63   335   11   MOLECULE: MULTIDRUG RESISTANCE PROTEIN MEXA;                         3351:  7tao-C  3.2  3.0   63   198   10   MOLECULE: V-TYPE PROTON ATPASE SUBUNIT C';                           3352:  6yj4-V  3.2  3.6   50   126    2   MOLECULE: NADH-UBIQUINONE OXIDOREDUCTASE CHAIN 3;                    3353:  2iak-A  3.2  2.9   74   196    8   MOLECULE: BULLOUS PEMPHIGOID ANTIGEN 1, ISOFORM 5;                   3354:  5l0w-A  3.2  3.1   60   162    8   MOLECULE: SEC72;                                                     3355:  4s0f-A  3.2  3.1   74   565    4   MOLECULE: ABC-TYPE BACTERIOCIN TRANSPORTER;                          3356:  3bkk-A  3.2  3.6   60   587    7   MOLECULE: ANGIOTENSIN-CONVERTING ENZYME, SOMATIC ISOFORM;            3357:  7q21-K  3.2  1.9   49    63    4   MOLECULE: CO-PURIFIED UNKNOWN TRANSMEMBRANE HELICES BUILT A          3358:  6u7h-C  3.2  3.5   66   965    9   MOLECULE: SPIKE GLYCOPROTEIN;                                        3359:  6u7h-A  3.2  3.5   66   965    9   MOLECULE: SPIKE GLYCOPROTEIN;                                        3360:  8fa1-A  3.2  1.9   50    71    6   MOLECULE: FERRITIN, DPS FAMILY PROTEIN AND SPIKE PROTEIN S2          3361:  8cvi-f  3.2  5.1   71   269    8   MOLECULE: FLAGELLIN;                                                 3362:  7sob-K  3.2  3.7   70  1025    6   MOLECULE: SPIKE GLYCOPROTEIN;                                        3363:  4akg-B  3.2  6.7   84  2650   10   MOLECULE: GLUTATHIONE S-TRANSFERASE CLASS-MU 26 KDA ISOZYME          3364:  5gof-A  3.2  3.8   67   390    6   MOLECULE: MITOFUSIN-1;                                               3365:  4cfi-A  3.2  2.9   62   258    8   MOLECULE: FLAGELLIN;                                                 3366:  7zh5-C  3.2  3.6   68   962    6   MOLECULE: SPIKE GLYCOPROTEIN,FIBRITIN;                               3367:  6bfu-C  3.2  3.9   68   964   13   MOLECULE: SPIKE PROTEIN;                                             3368:  6bfu-B  3.2  3.9   68   964   13   MOLECULE: SPIKE PROTEIN;                                             3369:  7rzu-A  3.2  2.9   50    71    8   MOLECULE: SARS-COV-2 HR1 A942S LINKED TO A SCAFFOLD,SPIKE P          3370:  5zvm-B  3.2  2.0   50    76    8   MOLECULE: SPIKE GLYCOPROTEIN;                                        3371:  1mof-A  3.1  1.9   46    53    9   MOLECULE: MOLONEY MURINE LEUKEMIA VIRUS P15;                         3372:  1usd-A  3.1  2.1   41    41   12   MOLECULE: VASODILATOR-STIMULATED PHOSPHOPROTEIN;                     3373:  5jvu-A  3.1  2.1   52    76    6   MOLECULE: CHIMERA PROTEIN OF KINESIN HEAVY CHAIN AND MICROT          3374:  6oeg-N  3.1  5.2   49   162    6   MOLECULE: TYPE IV SECRETION SYSTEM APPARATUS PROTEIN CAGX;           3375:  7v7b-A  3.1  2.8   57  1110    7   MOLECULE: DDB1- AND CUL4-ASSOCIATED FACTOR 1;                        3376:  8fn4-3  3.1  5.0   56   480    5   MOLECULE: RNA-EDITING SUBSTRATE-BINDING COMPLEX PROTEIN 1 (          3377:  8ppr-Z  3.1  1.1   39    39   10   MOLECULE: KINETOCHORE-ASSOCIATED PROTEIN DSN1 HOMOLOG;               3378:  7dgw-A  3.1  3.3   44    94   11   MOLECULE: A2;                                                        3379:  8i3e-B  3.1  2.0   50   135   12   MOLECULE: ELKS/RAB6-INTERACTING/CAST FAMILY MEMBER 1;                3380:  4ll7-A  3.1  3.1   50    94    8   MOLECULE: SWI5-DEPENDENT HO EXPRESSION PROTEIN 3;                    3381:  3bni-A  3.1  3.9   48   174    8   MOLECULE: PUTATIVE TETR-FAMILY TRANSCRIPTIONAL REGULATOR;            3382:  5h11-A  3.1  4.1   63   494    3   MOLECULE: UNCHARACTERIZED PROTEIN;                                   3383:  2wit-A  3.1  6.8   67   532    9   MOLECULE: GLYCINE BETAINE TRANSPORTER BETP;                          3384:  6mpb-A  3.1  3.0   63   447   11   MOLECULE: NEUTRAL AMINO ACID TRANSPORTER B(0);                       3385:  8jhq-A  3.1  4.8   67   446    6   MOLECULE: SPHINGOSINE-1-PHOSPHATE TRANSPORTER SPNS2,GLGA GL          3386:  7qe7-H  3.1  4.8   53    58    8   MOLECULE: ANAPHASE-PROMOTING COMPLEX SUBUNIT 10;                     3387:  6d42-A  3.1  1.4   39    39    3   MOLECULE: INTERMEDIATE CONDUCTANCE CALCIUM-ACTIVATED POTASS          3388:  7y88-A  3.1  7.0   73   354    8   MOLECULE: PUTATIVE GLUTAMATE DEHYDROGENASE/LEUCINE DEHYDROG          3389:  7oci-C  3.1  4.1   59   119    5   MOLECULE: DOLICHYL-DIPHOSPHOOLIGOSACCHARIDE--PROTEIN                 3390:  7r5s-S  3.1  4.7   47   120    4   MOLECULE: CENTROMERE PROTEIN H;                                      3391:  8bd7-A  3.1  3.3   50   539    2   MOLECULE: IFT88;                                                     3392:  5j8v-A  3.1  4.1   63  3398    5   MOLECULE: RYANODINE RECEPTOR 1;                                      3393:  7xzj-G  3.1  2.3   56    73    9   MOLECULE: CTAP3;                                                     3394:  3m62-A  3.1  2.4   65   955    5   MOLECULE: UBIQUITIN CONJUGATION FACTOR E4;                           3395:  8sjj-A  3.1  2.3   50    74    4   MOLECULE: SEPTIN-14;                                                 3396:  3wvz-A  3.1  5.9   60   192   10   MOLECULE: PROTEIN HIKESHI;                                           3397:  4qlb-D  3.1  3.4   62   651    3   MOLECULE: PROBABLE GLYCOGEN [STARCH] SYNTHASE;                       3398:  3w5m-A  3.1  5.0   51  1030    8   MOLECULE: PUTATIVE RHAMNOSIDASE;                                     3399:  8q7h-A  3.1  3.5   53  1403    4   MOLECULE: CULLIN-9;                                                  3400:  5wst-A  3.1  2.5   50    71   10   MOLECULE: UNCONVENTIONAL MYOSIN-VIIA;                                3401:  8hij-A  3.1  4.3   55   499    4   MOLECULE: BRIL-SLC19A1 CHIMERA;                                      3402:  7xpt-A  3.1  5.1   59   375   14   MOLECULE: TRANSGLYCOSYLSE;                                           3403:  6h3z-A  3.1  3.9   53   208    9   MOLECULE: CCR4-NOT TRANSCRIPTION COMPLEX SUBUNIT 1;                  3404:  4y66-C  3.1  2.1   50   197   10   MOLECULE: MND1;                                                      3405:  3afl-A  3.1  5.6   53   766    6   MOLECULE: OLIGO ALGINATE LYASE;                                      3406:  5i9e-H  3.1  2.0   44    44    5   MOLECULE: ACTIN-RELATED PROTEIN 4;                                   3407:  8bh1-A  3.1  3.1   57   348    7   MOLECULE: PROBABLE PEPTIDOGLYCAN GLYCOSYLTRANSFERASE FTSW;           3408:  6sih-A  3.1  4.1   83   488    4   MOLECULE: FLAGELLAR HOOK-ASSOCIATED PROTEIN 2;                       3409:  8j22-C  3.1  2.7   63   285    8   MOLECULE: GUANINE NUCLEOTIDE-BINDING PROTEIN G(I)/G(S)/G(T)          3410:  7w1m-B  3.1  3.6   85   528    7   MOLECULE: STRUCTURAL MAINTENANCE OF CHROMOSOMES PROTEIN 1A;          3411:  3o1j-A  3.1  3.0   81   273    9   MOLECULE: SENSOR PROTEIN TORS;                                       3412:  5dku-B  3.1  4.5   63   581    5   MOLECULE: PREX DNA POLYMERASE;                                       3413:  5fiy-A  3.1  2.3   50    78    2   MOLECULE: PRKC APOPTOSIS WT1 REGULATOR PROTEIN;                      3414:  5jx1-A  3.1  3.1   51    64    6   MOLECULE: CHIMERA PROTEIN OF KINESIN-LIKE PROTEIN KIF3A AND          3415:  6zyx-C  3.1  3.6   64   278   11   MOLECULE: DYNEIN HEAVY CHAIN, OUTER ARM PROTEIN;                     3416:  8ag6-B  3.1  7.0   80   975   11   MOLECULE: DNA MISMATCH REPAIR PROTEIN MSH2;                          3417:  8oyy-A  3.1  5.8   67   233    6   MOLECULE: DE NOVO DESIGNED SOLUBLE GPCR-LIKE PROTEIN;                3418:  2xcq-A  3.1  5.4   75   652    4   MOLECULE: DNA GYRASE SUBUNIT B, DNA GYRASE SUBUNIT A;                3419:  2bl2-A  3.1  4.5   68   156    3   MOLECULE: V-TYPE SODIUM ATP SYNTHASE SUBUNIT K;                      3420:  4wid-A  3.1  4.9   78   353    8   MOLECULE: RHUL123;                                                   3421:  8tn6-C  3.1  5.8   71   147    8   MOLECULE: DE NOVO DESIGNED PROTEIN;                                  3422:  6zl1-C  3.1  2.1   56   122    5   MOLECULE: ALBUMIN;                                                   3423:  7zw6-C  3.1  3.2   75   793   12   MOLECULE: SLR0869 PROTEIN;                                           3424:  6ziu-a  3.1  3.4   76   226    5   MOLECULE: ATP SYNTHASE PROTEIN 8;                                    3425:  7mit-E  3.1  4.3   70  1440    9   MOLECULE: ATP-SENSITIVE INWARD RECTIFIER POTASSIUM CHANNEL           3426:  6g8z-C  3.1  3.6   76   318    4   MOLECULE: VOLUME-REGULATED ANION CHANNEL SUBUNIT LRRC8A;             3427:  6uqf-A  3.1  8.2   74   531    7   MOLECULE: POTASSIUM/SODIUM HYPERPOLARIZATION-ACTIVATED CYCL          3428:  4u83-A  3.1  3.3   68   371    4   MOLECULE: ACYL-COA DEHYDROGENASE;                                    3429:  8q0p-B  3.1  2.5   60   230    7   MOLECULE: VARIANT SURFACE GLYCOPROTEIN MITAT 1.21;                   3430:  4bul-A  3.1  5.6   76   674    4   MOLECULE: DNA GYRASE SUBUNIT B, DNA GYRASE SUBUNIT A;                3431:  7cuj-A  3.1  4.1   50   258    4   MOLECULE: COILED-COIL QUANTITATIVELY-ENRICHED PROTEIN 1;             3432:  6n4n-C  3.1  2.3   50   184    6   MOLECULE: NS3 PROTEASE;                                              3433:  4xa6-C  3.1  2.1   54   116    9   MOLECULE: GP7-MYH7(1777-1855)-EB1 CHIMERA PROTEIN;                   3434:  5awi-A  3.1  5.4   52   106   13   MOLECULE: SOLUBLE CYTOCHROME B562;                                   3435:  7n5g-B  3.1  2.9   56   371    5   MOLECULE: MECHANOSENSITIVE ION CHANNEL FLYCATCHER1;                  3436:  5u6o-C  3.1  7.5   70   482    7   MOLECULE: POTASSIUM/SODIUM HYPERPOLARIZATION-ACTIVATED CYCL          3437:  5m0i-A  3.1  4.3   67   225    7   MOLECULE: SWI5-DEPENDENT HO EXPRESSION PROTEIN 2;                    3438:  6iko-A  3.1  3.5   81   303    5   MOLECULE: GROWTH ARREST-SPECIFIC PROTEIN 7;                          3439:  3ux4-A  3.1  1.7   48   180    6   MOLECULE: ACID-ACTIVATED UREA CHANNEL;                               3440:  6umm-C  3.1  4.3   68   452   10   MOLECULE: ESX-3 SECRETION SYSTEM PROTEIN ECCE3;                      3441:  4r0g-A  3.1  3.4   48   253    4   MOLECULE: UNCHARACTERIZED PROTEIN;                                   3442:  6vhr-B  3.1  3.2   78   164    8   MOLECULE: PE FAMILY IMMUNOMODULATOR PE5;                             3443:  1f6f-A  3.1  2.5   63   183   10   MOLECULE: PLACENTAL LACTOGEN;                                        3444:  2lch-A  3.1  2.5   54   113   11   MOLECULE: PROTEIN OR38;                                              3445:  1ovr-A  3.1  2.1   43    48   16   MOLECULE: FOUR-HELIX BUNDLE MODEL DI-MN(II)-DF1-L13;                 3446:  3geh-A  3.1  3.0   69   443    4   MOLECULE: TRNA MODIFICATION GTPASE MNME;                             3447:  7d0i-J  3.1  2.5   44   424    2   MOLECULE: AUTOPHAGY-RELATED PROTEIN 9;                               3448:  3qf4-A  3.1  3.8   75   572    3   MOLECULE: ABC TRANSPORTER, ATP-BINDING PROTEIN;                      3449:  7rit-B  3.1  3.3   73   569    3   MOLECULE: ATP-DEPENDENT LIPID A-CORE FLIPPASE;                       3450:  2nnu-A  3.1  1.7   52   201    2   MOLECULE: REGULATORY PROTEIN E2;                                     3451:  5tgw-A  3.1  4.0   58   109    5   MOLECULE: PS1;                                                       3452:  3ce2-A  3.1  3.3   53   595    9   MOLECULE: PUTATIVE PEPTIDASE;                                        3453:  6uun-R  3.1  2.0   55   354    7   MOLECULE: GUANINE NUCLEOTIDE-BINDING PROTEIN G(S) SUBUNIT A          3454:  6e67-B  3.1  2.3   57   476    4   MOLECULE: BETA-2 ADRENERGIC RECEPTOR,ENDOLYSIN,GUANINE NUCL          3455:  7n5d-A  3.1  3.1   61   386    5   MOLECULE: MECHANOSENSITIVE ION CHANNEL FLYCATCHER1;                  3456:  3whl-F  3.1  3.2   56    90    9   MOLECULE: PROTEASOME-ACTIVATING NUCLEOTIDASE, 26S PROTEASE           3457:  6nxm-A  3.1  2.3   51    92    2   MOLECULE: DESIGN CONSTRUCT XAA_GVDQ;                                 3458:  3dyj-A  3.1  3.1   65   317    9   MOLECULE: TALIN-1;                                                   3459:  7r5v-U  3.1  1.8   49   165    8   MOLECULE: CENTROMERE PROTEIN H;                                      3460:  3apz-A  3.1  3.9   53   299    6   MOLECULE: GERANYL DIPHOSPHATE SYNTHASE;                              3461:  1r42-A  3.1  6.0   65   597    6   MOLECULE: ANGIOTENSIN I CONVERTING ENZYME 2;                         3462:  6z16-G  3.1  2.4   55   104    9   MOLECULE: MULTISUBUNIT NA+/H+ ANTIPORTER, A SUBUNIT;                 3463:  6zit-R  3.1  2.8   43    57    5   MOLECULE: ATP SYNTHASE PROTEIN 8;                                    3464:  3zci-A  3.1  2.9   64   191   11   MOLECULE: CAG PATHOGENICITY ISLAND PROTEIN (CAG18);                  3465:  5szs-A  3.1  3.9   66  1177    6   MOLECULE: SPIKE GLYCOPROTEIN;                                        3466:  5gns-A  3.1  3.5   71   377    1   MOLECULE: MITOFUSIN-1;                                               3467:  6jy0-G  3.1  3.3   67   410    9   MOLECULE: FLAGELLIN;                                                 3468:  2d4c-A  3.1  4.8   64   224   14   MOLECULE: SH3-CONTAINING GRB2-LIKE PROTEIN 2;                        3469:  6acj-C  3.1  4.3   65  1057    8   MOLECULE: SPIKE GLYCOPROTEIN;                                        3470:  6gow-D  3.1  2.9   61   256   10   MOLECULE: FLAGELLIN;                                                 3471:  2d4c-D  3.1  4.6   66   217   11   MOLECULE: SH3-CONTAINING GRB2-LIKE PROTEIN 2;                        3472:  8px9-A  3.1  2.2   63   557    6   MOLECULE: MICROCIN-J25 EXPORT ATP-BINDING/PERMEASE PROTEIN           3473:  1y4m-A  3.0  2.3   47    53    4   MOLECULE: HERV-FRD_6P24.1 PROVIRUS ANCESTRAL ENV POLYPROTEI          3474:  8dft-A  3.0  3.9   61   112    5   MOLECULE: PILIN PROTEIN;                                             3475:  1s1c-Y  3.0  2.4   50    70    8   MOLECULE: TRANSFORMING PROTEIN RHOA;                                 3476:  8q85-X  3.0  2.4   49    68    8   MOLECULE: KINETOCHORE PROTEIN NDC80;                                 3477:  3iv1-A  3.0  2.8   50    78    2   MOLECULE: TUMOR SUSCEPTIBILITY GENE 101 PROTEIN;                     3478:  7lzh-A  3.0  5.6   68   799    3   MOLECULE: GLUTAMATE RECEPTOR 3.4;                                    3479:  5oge-G  3.0  3.5   58   306   10   MOLECULE: GDP-MANNOSE TRANSPORTER 1;                                 3480:  7nsb-7  3.0  6.5   54   531    2   MOLECULE: VACUOLAR IMPORT AND DEGRADATION PROTEIN 30;                3481:  6odm-B  3.0  2.6   46    48   11   MOLECULE: MAJOR CAPSID PROTEIN;                                      3482:  6yj4-L  3.0  2.6   52   655    6   MOLECULE: NADH-UBIQUINONE OXIDOREDUCTASE CHAIN 3;                    3483:  2l35-A  3.0  3.1   53    62   11   MOLECULE: DAP12-NKG2C_TM;                                            3484:  2c0s-A  3.0  2.7   46    64    7   MOLECULE: CONSERVED DOMAIN PROTEIN;                                  3485:  8r5i-B  3.0  2.6   43    43    7   MOLECULE: CORE PROTEIN A10;                                          3486:  3eo8-A  3.0  4.3   50   219   12   MOLECULE: BLUB-LIKE FLAVOPROTEIN;                                    3487:  8ts6-A  3.0  5.4   52   500    4   MOLECULE: PORTAL PROTEIN;                                            3488:  2ooc-B  3.0  3.8   60   105    5   MOLECULE: HISTIDINE PHOSPHOTRANSFERASE;                              3489:  5ibw-C  3.0  2.7   42    43    5   MOLECULE: CALCIUM-BINDING EF-HAND DOMAIN-CONTAINING PROTEIN          3490:  8c8q-I  3.0  3.3   48    55    0   MOLECULE: CYTOCHROME C OXIDASE SUBUNIT 1;                            3491:  6wgv-A  3.0  4.7   51   173    4   MOLECULE: CORRINOID ADENOSYLTRANSFERASE;                             3492:  6nyi-A  3.0  2.3   57    97    2   MOLECULE: DESIGN CONSTRUCT XXA;                                      3493:  6fpd-A  3.0  2.6   67   191   10   MOLECULE: PROTEIN AB21;                                              3494:  8wag-B  3.0  3.4   56   235   14   MOLECULE: PROTEIN CHUP1, CHLOROPLASTIC;                              3495:  5oc0-A  3.0  2.9   68   174    6   MOLECULE: CYTOCHROME B561;                                           3496:  6irw-A  3.0  6.5   58   495    3   MOLECULE: PHOSPHORYLATED CTD-INTERACTING FACTOR 1;                   3497:  6u8y-b  3.0  2.5   51    79    6   MOLECULE: MONOVALENT CATION/H+ ANTIPORTER SUBUNIT E;                 3498:  8tek-N  3.0  3.1   50   187    8   MOLECULE: DYNEIN REGULATORY COMPLEX PROTEIN 1/2 N-TERMINAL           3499:  2qqy-A  3.0  3.4   62   139   10   MOLECULE: SIGMA B OPERON;                                            3500:  7mi8-A  3.0  3.2   52   868    6   MOLECULE: FUSION PROTEIN OF DYNEIN AND ENDOLYSIN;                    3501:  8fxf-A  3.0  2.6   50    78   18   MOLECULE: E3 UBIQUITIN-PROTEIN LIGASE TRIM56;                        3502:  6jho-A  3.0  3.6   67   200    3   MOLECULE: CAG PATHOGENICITY ISLAND PROTEIN (CAG6);                   3503:  6eyu-A  3.0  7.7   61   232    8   MOLECULE: BACTERIORHODOPSIN;                                         3504:  7woo-D  3.0  5.4   74  1398    9   MOLECULE: NUCLEOPORIN NIC96;                                         3505:  4wy4-C  3.0  1.7   50    78    2   MOLECULE: VESICLE-ASSOCIATED MEMBRANE PROTEIN 8;                     3506:  6cfz-C  3.0  1.8   50    79   16   MOLECULE: ASK1;                                                      3507:  7u8g-A  3.0  4.2   62   260    8   MOLECULE: EGFP, CYTOCHROME B-245 HEAVY CHAIN CHIMERA;                3508:  5hda-A  3.0  2.0   50   121    0   MOLECULE: ZINC FINGER MYND DOMAIN-CONTAINING PROTEIN 11;             3509:  5xau-C  3.0  3.0   50    70    6   MOLECULE: LAMININ SUBUNIT ALPHA-5;                                   3510:  1bhb-A  3.0  2.6   51    67    6   MOLECULE: BACTERIORHODOPSIN;                                         3511:  8ab6-Q  3.0  4.8   57    71    5   MOLECULE: CYTOCHROME B;                                              3512:  7emf-D  3.0  2.1   55   158   11   MOLECULE: MEDIATOR OF RNA POLYMERASE II TRANSCRIPTION SUBUN          3513:  8teu-A  3.0  2.5   56   722    9   MOLECULE: LARGE TEGUMENT PROTEIN DENEDDYLASE;                        3514:  6jx7-A  3.0  3.9   62  1245    5   MOLECULE: FELINE INFECTIOUS PERITONITIS VIRUS SPIKE PROTEIN          3515:  3trt-A  3.0  2.9   50    75    6   MOLECULE: VIMENTIN;                                                  3516:  6iak-F  3.0  1.8   50    71    8   MOLECULE: UNCHARACTERIZED PROTEIN;                                   3517:  8uao-A  3.0  2.0   64   226    9   MOLECULE: DPHF18;                                                    3518:  6kg7-A  3.0  3.9   55  1817    4   MOLECULE: PIEZO-TYPE MECHANOSENSITIVE ION CHANNEL COMPONENT          3519:  7bvf-A  3.0  3.4   55  1087    7   MOLECULE: PROBABLE ARABINOSYLTRANSFERASE B;                          3520:  6rgo-C  3.0  1.7   38    38    8   MOLECULE: AUTOPHAGY-RELATED PROTEIN 21;                              3521:  7zmg-d  3.0  2.9   59   101    2   MOLECULE: NADH-UBIQUINONE OXIDOREDUCTASE CHAIN 1;                    3522:  1a92-A  3.0  5.4   48    50    4   MOLECULE: DELTA ANTIGEN;                                             3523:  7byl-A  3.0  3.9   63   354    6   MOLECULE: GREEN FLUORESCENT PROTEIN,POTASSIUM VOLTAGE-GATED          3524:  7w5m-A  3.0  3.4   49   227   10   MOLECULE: TETRATRICOPEPTIDE REPEAT (TPR)-LIKE SUPERFAMILY P          3525:  6cpu-A  3.0  6.1   54   526    7   MOLECULE: PHOSPHODIESTERASE;                                         3526:  6teq-A  3.0  2.3   48   418    2   MOLECULE: GALACTOKINASE;                                             3527:  8grn-A  3.0  4.1   61   716    8   MOLECULE: PROTEIN OSCA1;                                             3528:  7ryq-B  3.0  3.5   52   527    6   MOLECULE: KIF-BINDING PROTEIN;                                       3529:  8q85-b  3.0  2.3   50    70   10   MOLECULE: KINETOCHORE PROTEIN NDC80;                                 3530:  2np9-A  3.0  4.5   53   423    2   MOLECULE: DPGC;                                                      3531:  3b4r-A  3.0  2.8   53   218   11   MOLECULE: PUTATIVE ZINC METALLOPROTEASE MJ0392;                      3532:  4m1p-A  3.0  4.1   60    96    5   MOLECULE: COPPER-SENSITIVE OPERON REPRESSOR (CSOR);                  3533:  3wmi-B  3.0  0.7   37    37   11   MOLECULE: EIAV GP45 WILD TYPE;                                       3534:  5djn-C  3.0  2.5   50    81    8   MOLECULE: KINESIN-LIKE PROTEIN;                                      3535:  1ku9-A  3.0  4.0   60   151    7   MOLECULE: HYPOTHETICAL PROTEIN MJ223;                                3536:  7w0y-k  3.0  3.1   53    98    9   MOLECULE: NADH DEHYDROGENASE [UBIQUINONE] FLAVOPROTEIN 1,            3537:  8izl-B  3.0  1.5   49    69   12   MOLECULE: PHOSPHOPROTEIN;                                            3538:  5an3-B  3.0  3.0   44   136   11   MOLECULE: SGT1;                                                      3539:  6zka-K  3.0  3.0   52    98   10   MOLECULE: NADH-UBIQUINONE OXIDOREDUCTASE CHAIN 3;                    3540:  6bbf-L  3.0  2.0   55   150   13   MOLECULE: CALCIUM RELEASE-ACTIVATED CALCIUM CHANNEL PROTEIN          3541:  8tl7-S  3.0  6.7   61   694    8   MOLECULE: COMPUTATIONALLY DESIGNED PROTEIN;                          3542:  7pv7-A  3.0  2.8   51   513    8   MOLECULE: RESPONSE REGULATOR;                                        3543:  7pvk-B  3.0  2.6   51   513    8   MOLECULE: RESPONSE REGULATOR;                                        3544:  6h2f-B  3.0  3.5   80   344    6   MOLECULE: AHLB;                                                      3545:  8txr-k  3.0  3.0   49    60    8   MOLECULE: EXODEOXYRIBONUCLEASE 7 LARGE SUBUNIT;                      3546:  6ziq-a  3.0  3.4   77   226    5   MOLECULE: ATP SYNTHASE PROTEIN 8;                                    3547:  7eeb-D  3.0  3.1   58   242    9   MOLECULE: ENHANCED GREEN FLUORESCENT PROTEIN,CATION CHANNEL          3548:  6nz3-B  3.0  2.1   49    91    2   MOLECULE: DESIGN CONSTRUCT XAA_GGHN;                                 3549:  2o5j-D  3.0 25.5   61  1264    7   MOLECULE: 5'-D(P*CP*CP*CP*TP*GP*TP*CP*TP*GP*GP*CP*GP*TP*TP*          3550:  7sn7-C  3.0  2.9   85   546    5   MOLECULE: FLAGELLIN;                                                 3551:  7enj-G  3.0  2.7   60   161    5   MOLECULE: MEDIATOR OF RNA POLYMERASE II TRANSCRIPTION SUBUN          3552:  6o0i-A  3.0  3.1   55    98    5   MOLECULE: DESIGN CONSTRUCT XAA;                                      3553:  4ons-C  3.0  2.7   61   205    5   MOLECULE: CATENIN ALPHA-2;                                           3554:  7emf-G  3.0  2.7   60   161    5   MOLECULE: MEDIATOR OF RNA POLYMERASE II TRANSCRIPTION SUBUN          3555:  1k05-B  3.0  2.7   64   142    8   MOLECULE: FOCAL ADHESION KINASE 1;                                   3556:  6o0i-C  3.0  3.1   55    98    5   MOLECULE: DESIGN CONSTRUCT XAA;                                      3557:  3i2w-B  3.0  4.8   80   285    8   MOLECULE: SYNDAPIN;                                                  3558:  1yc9-A  3.0  2.6   79   411    8   MOLECULE: MULTIDRUG RESISTANCE PROTEIN;                              3559:  6l3t-A  3.0  2.8   62   201    2   MOLECULE: GAP JUNCTION GAMMA-3 PROTEIN;                              3560:  4by1-A  3.0  4.3   60  1427    3   MOLECULE: DNA-DIRECTED RNA POLYMERASE II SUBUNIT RPB1;               3561:  6xbo-A  3.0  3.8   62   319    5   MOLECULE: CMP-SIALIC ACID TRANSPORTER;                               3562:  8d08-C  3.0  4.3   53    66   11   MOLECULE: HALC4_135;                                                 3563:  6s7t-C  3.0  4.6   62    78    8   MOLECULE: DOLICHYL-DIPHOSPHOOLIGOSACCHARIDE--PROTEIN                 3564:  1t7p-A  3.0  5.9   66   662    6   MOLECULE: DNA (5'-D(P*GP*CP*CP*AP*GP*TP*GP*CP*CP*AP*2DA)-            3565:  7n5d-B  3.0  2.9   55   371    5   MOLECULE: MECHANOSENSITIVE ION CHANNEL FLYCATCHER1;                  3566:  5w5h-A  3.0  2.6   53   451    4   MOLECULE: INTERFERON-INDUCED PROTEIN WITH TETRATRICOPEPTIDE          3567:  1occ-C  3.0  3.1   65   261    3   MOLECULE: CYTOCHROME C OXIDASE;                                      3568:  5fil-W  3.0  4.0   73   217    5   MOLECULE: ATP SYNTHASE SUBUNIT ALPHA, MITOCHONDRIAL;                 3569:  4zsv-A  3.0  2.0   48   294   10   MOLECULE: UNCHARACTERIZED PROTEIN;                                   3570:  8csr-U  3.0  2.6   50   174   14   MOLECULE: 28S RIBOSOMAL PROTEIN S34, MITOCHONDRIAL;                  3571:  3c7n-A  3.0  2.9   54   648    4   MOLECULE: HEAT SHOCK PROTEIN HOMOLOG SSE1;                           3572:  2hyd-A  3.0  4.1   79   578    3   MOLECULE: ABC TRANSPORTER HOMOLOG;                                   3573:  5v4s-A  3.0  3.5   68   387    7   MOLECULE: TRANSPORTER, CATION CHANNEL FAMILY / CYCLIC NUCLE          3574:  3anw-A  3.0  2.0   53   171    4   MOLECULE: PUTATIVE UNCHARACTERIZED PROTEIN;                          3575:  7y8t-B  3.0  5.4   47   680    6   MOLECULE: CHAT DOMAIN PROTEIN;                                       3576:  6jx7-B  3.0  3.9   62  1245    5   MOLECULE: FELINE INFECTIOUS PERITONITIS VIRUS SPIKE PROTEIN          3577:  8h11-A  3.0  3.7   70   979    6   MOLECULE: SPIKE GLYCOPROTEIN;                                        3578:  2z0v-B  3.0  2.9   78   221    9   MOLECULE: SH3-CONTAINING GRB2-LIKE PROTEIN 3;                        3579:  2d4c-C  3.0  2.7   73   226   15   MOLECULE: SH3-CONTAINING GRB2-LIKE PROTEIN 2;                        3580:  3qmz-B  3.0  3.6   65  2136    2   MOLECULE: CYTOPLASMIC DYNEIN HEAVY CHAIN;                            3581:  6jx7-C  3.0  3.9   62  1245    5   MOLECULE: FELINE INFECTIOUS PERITONITIS VIRUS SPIKE PROTEIN          3582:  3kyj-A  2.9  3.6   58   129    7   MOLECULE: PUTATIVE HISTIDINE PROTEIN KINASE;                         3583:  7uxc-R  2.9  2.1   44   107    7   MOLECULE: SERINE/THREONINE-PROTEIN KINASE MTOR;                      3584:  7n8x-A  2.9  3.5   50   691    8   MOLECULE: CHONDROITIN SULFATE PROTEOGLYCAN 4;                        3585:  8av6-H  2.9  2.6   42    98   12   MOLECULE: RUVB-LIKE HELICASE;                                        3586:  5yep-B  2.9  2.6   54   133    6   MOLECULE: TOXIN-ANTITOXIN SYSTEM ANTIDOTE MNT FAMILY;                3587:  5toh-B  2.9  4.5   50    70    8   MOLECULE: POLYMERASE COFACTOR VP35;                                  3588:  7zl9-A  2.9  6.9   49   398   14   MOLECULE: HYDROXYCARBOXYLIC ACID RECEPTOR 2,SOLUBLE CYTOCHR          3589:  8eau-e  2.9  4.7   53    72    4   MOLECULE: V-TYPE PROTON ATPASE SUBUNIT A, VACUOLAR ISOFORM;          3590:  5mq4-C  2.9  2.9   50   117   16   MOLECULE: PROTEIN KINASE C-BINDING PROTEIN 1;                        3591:  2pnv-A  2.9  1.7   39    39    8   MOLECULE: SMALL CONDUCTANCE CALCIUM-ACTIVATED POTASSIUM              3592:  5b2g-C  2.9  3.4   70   350    7   MOLECULE: ENDOLYSIN,CLAUDIN-4;                                       3593:  2jee-A  2.9  2.0   50    78    8   MOLECULE: CELL DIVISION PROTEIN ZAPB;                                3594:  8sdi-A  2.9  2.5   50    80    8   MOLECULE: TRIPARTITE MOTIF-CONTAINING PROTEIN 45;                    3595:  8wtc-B  2.9  7.1   45   100    7   MOLECULE: PROTEIN-ARGININE KINASE;                                   3596:  1vp7-B  2.9  2.8   52    77    2   MOLECULE: EXODEOXYRIBONUCLEASE VII SMALL SUBUNIT;                    3597:  6bpz-A  2.9  5.9   59   900   12   MOLECULE: PIEZO-TYPE MECHANOSENSITIVE ION CHANNEL COMPONENT          3598:  5o01-A  2.9  2.8   44   254   14   MOLECULE: BKLC (BACTERIAL KINESIN-LIGHT CHAIN-LIKE);                 3599:  6nps-A  2.9  4.0   57   968   14   MOLECULE: AXYAGU115A;                                                3600:  7p5v-B  2.9  4.5   72   732    7   MOLECULE: VOLUME-REGULATED ANION CHANNEL SUBUNIT LRRC8A;             3601:  7t5p-B  2.9  3.0   45   419   11   MOLECULE: SUMO-INTERACTING MOTIF-CONTAINING PROTEIN 1;               3602:  7c86-A  2.9  4.2   57   296    7   MOLECULE: SENSORY OPSIN A,CHANNELRHODOPSIN (CHR) CHIMERA BE          3603:
[truncated: 1,200,000 more chars]
